# Reduced modeling of signal transduction – a modular approach

**DOI:** 10.1186/1471-2105-8-336

**Published:** 2007-09-13

**Authors:** Markus Koschorreck, Holger Conzelmann, Sybille Ebert, Michael Ederer, Ernst Dieter Gilles

**Affiliations:** 1 Max Planck Institute for Dynamics of Complex Technical Systems, Sandtorstr. 1, 39106 Magdeburg, Germany

## Abstract

**Background:**

Combinatorial complexity is a challenging problem in detailed and mechanistic mathematical modeling of signal transduction. This subject has been discussed intensively and a lot of progress has been made within the last few years. A software tool (BioNetGen) was developed which allows an automatic rule-based set-up of mechanistic model equations. In many cases these models can be reduced by an exact domain-oriented lumping technique. However, the resulting models can still consist of a very large number of differential equations.

**Results:**

We introduce a new reduction technique, which allows building modularized and highly reduced models. Compared to existing approaches further reduction of signal transduction networks is possible. The method also provides a new modularization criterion, which allows to dissect the model into smaller modules that are called layers and can be modeled independently. Hallmarks of the approach are conservation relations within each layer and connection of layers by signal flows instead of mass flows. The reduced model can be formulated directly without previous generation of detailed model equations. It can be understood and interpreted intuitively, as model variables are macroscopic quantities that are converted by rates following simple kinetics. The proposed technique is applicable without using complex mathematical tools and even without detailed knowledge of the mathematical background. However, we provide a detailed mathematical analysis to show performance and limitations of the method. For physiologically relevant parameter domains the transient as well as the stationary errors caused by the reduction are negligible.

**Conclusion:**

The new layer based reduced modeling method allows building modularized and strongly reduced models of signal transduction networks. Reduced model equations can be directly formulated and are intuitively interpretable. Additionally, the method provides very good approximations especially for macroscopic variables. It can be combined with existing reduction methods without any difficulties.

## Background

### Modeling of signaling pathways

Systems biology aims at a holistic understanding of cellular processes. Mathematical models that integrate the current state of knowledge are analyzed to understand system properties that are not apparent from the characteristics of their components.

Different approaches exist to model and analyze signal transduction systems. Qualitative modeling uses solely structural information about the network and performs no quantitative statements. Examples for qualitative modeling techniques applied to signal transduction pathways are Petri nets [1], interaction graphs and Boolean networks [2]. Qualitative models can be analyzed by computational studies and structural analysis. As an example, independent signaling paths and feedback circuits can be obtained from an interaction graph using logical elementary modes [2]. The concept of T-invariants in Petri nets allows to identify self contained subnets that are active under a given input situation [1]. With both techniques it is possible to decompose a signaling network into smaller functional units. Qualitative analysis is especially helpful if structural information but relatively little quantitative information about the system is known. Quantitative modeling explicitly considers the quantitative nature of physical systems. Processes on the molecular level exhibit stochastic behavior. When only a low number of a specific molecule is present, stochastic modeling techniques should be used. The chemical master equations [3] describe the stochastic dynamics of chemical systems in a quantitative way. However, they are difficult to analyze and simulate. If concentrations are high enough, the system dynamics shows deterministic behavior. Thus, in many cases a simplified approach neglecting the stochastic nature of the dynamics can be used.

Inside the cell, signals propagate in time and space. Therefore, partial differential equations should be used to describe them exactly. This continuous spatial behavior can often be modeled by assuming fast distribution of all species inside a cellular compartment and transport reactions between the compartments. This leads to model equations in the form of ordinary differential equations (ODEs). If processes are modeled on a molecular level mass action kinetics are a good description of the chemical processes and are frequently used. This view is the basis for the work presented here and also used by many others [4-13].

The more information about the system is available, the more powerful quantitative modeling techniques become, as prediction accuracy of models grows.

### Combinatorial variety

Modeling with ODEs is challenged by biological reality. In signal transduction, association and modification of a relatively small number of different molecules usually give rise to an enormous amount of possible protein complexes [14]. This problem of combinatorial complexity has been tackled in different ways. In 1998, a stochastic modeling tool called STOCHSIM was developed to circumvent the problem of combinatorial complexity in bacterial chemotaxis [15,16]. This algorithm is especially suited for systems with a very low number of molecules where stochastic effects may play an important role. Up to now there is research on stochastic simulations of combinatorial systems.

Many deterministic models of large signaling networks that have been published within the last years neglect the combinatorial variety of protein complexes. Their focus is on small subsets of the occurring reactions and complexes [4-11]. It was shown that such reduced model structures may provide a good approximation to a model accounting for all feasible protein complexes and reactions [17]. However, the main difficulty is to decide which reactions and complexes can be neglected and which are essential. The suited reduced model structure highly depends on the kinetic parameters of the reaction network [17]. Even in very simple examples an apparently reasonable assumption may lead to significant approximation errors [18].

An alternative approach was suggested by Blinov et al. [19], who introduced the software tool BioNetGen. This program translates a rule-based model formulation into a complete ordinary diffierential equation (ODE) model accounting for all feasible reactions and species. Additionally, a graphical rule-based representation of signaling networks has been introduced [20,21]. BioNetGen was used to generate complete mechanistic models of early events in Fc*ε*RI [12] and EGF signaling [13]. In both cases only a very limited number of components and binding domains are included. Nevertheless, more than three hundred ODEs are required in the case of Fc*ε*RI signaling and over one thousand for EGF signaling. A detailed model of a complete signaling cascade easily can grow to millions or even billions of equations as shown below for the insulin signaling pathway. Recently, a novel approach to tackle this combinatorial explosion of model equations has been presented [22], formally extended and generalized [18]. The approach bases on the view that the fundamental elements of signal transduction are domains instead of molecular species [23]. The conventional mechanistic description of all feasible multi-protein complexes is replaced by a macroscopic one, where the focus is on the state of domains like levels of occupancy or degrees of phosphorylation. For many realistic systems this method allows a very strong reduction of the complete combinatorial models. The approach proposed by Borisov et al. [22] identifies reduction opportunities based on site dependencies that are determined from rules, not from the expanded network. The main deficiency of that method is that it considers only modularity within a single multi-site molecule and does not consider modularity of the reaction network as a whole.

The exact domain-oriented lumping technique proposed by Conzelmann et al. [18] guarantees an accurate description of macroscopic quantities. It is also applicable to whole reaction networks. However, a prerequisite for using this method is availability of the detailed model equations. The resulting models most often still consist of a very large number of ODEs which makes them difficult to handle and to analyze. In the following the insulin signaling pathway will serve as a realistic example to exemplify combinatorial variety. Afterwards, we introduce a new approximative reduced modeling method and show its efficiency by mathematical analysis and applying it to insulin signaling models of varying levels of detail.

### Insulin signaling and combinatorial variety

The insulin signaling system is of high medical interest and therefore well studied [24-27]. Insulin regulates cellular glucose uptake [24,28] and has a major impact on metabolism [26,29]. It promotes synthesis and storage of glycogen, proteins and lipids and negatively regulates their degradation. Additionally, it negatively regulates secretion of sugars, amino acids and fatty acids [25]. Insulin is also involved in gene expression [30], cell survival and differentiation.

Defects in the insulin signaling system give rise to insulin resistance, obesity and type II diabetes mellitus [31-33]. There are intense efforts to improve therapies of these maladies [34-37].

In a simplified form the insulin signaling system will serve as demonstration object for combinatorial complexity. The insulin receptor is a transmembrane protein that is constitutively dimerized [38].

**Figure 1:**
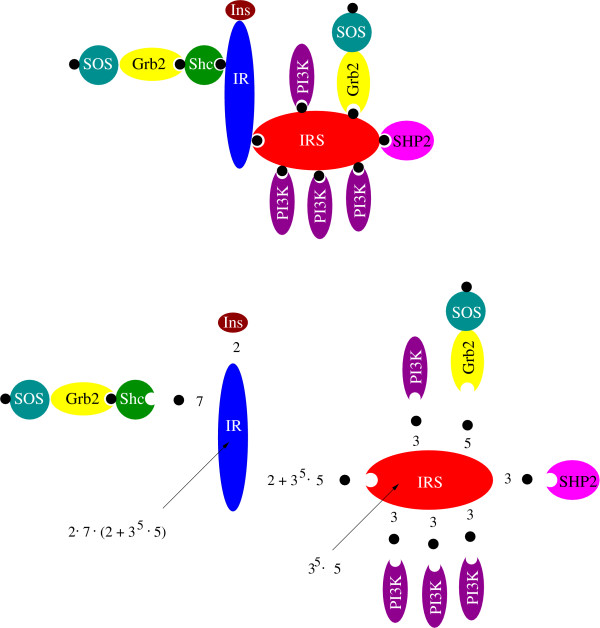
**Combinatorial complexity**. The full combinatorial complexity of the described parts of insulin signaling is demonstrated. The insulin receptor can bind Insulin, Shc and IRS. IRS can bind four PI3K molecules, SHP2 and Grb2. Grb2 can bind SOS and phosphorylated SOS. This results in 3^5^·5 = 1215 different complexes with IRS. For Shc and insulin binding to the receptor monomer there are seven and two possibilities, respectively. Altogether there are *n *= 2·7·(3^5^·5 + 2) = 17038 different complexes of the receptor monomer. As the receptor is a dimer (*k *= 2) this setup allows (n+k−1k)
 MathType@MTEF@5@5@+=feaafiart1ev1aaatCvAUfKttLearuWrP9MDH5MBPbIqV92AaeXatLxBI9gBaebbnrfifHhDYfgasaacH8akY=wiFfYdH8Gipec8Eeeu0xXdbba9frFj0=OqFfea0dXdd9vqai=hGuQ8kuc9pgc9s8qqaq=dirpe0xb9q8qiLsFr0=vr0=vr0dc8meaabaqaciaacaGaaeqabaqabeGadaaakeaadaqadaqaauaabeqaceaaaeaacqWGUbGBcqGHRaWkcqWGRbWAcqGHsislcqaIXaqmaeaacqWGRbWAaaaacaGLOaGaayzkaaaaaa@3524@ = (170392)
 MathType@MTEF@5@5@+=feaafiart1ev1aaatCvAUfKttLearuWrP9MDH5MBPbIqV92AaeXatLxBI9gBaebbnrfifHhDYfgasaacH8akY=wiFfYdH8Gipec8Eeeu0xXdbba9frFj0=OqFfea0dXdd9vqai=hGuQ8kuc9pgc9s8qqaq=dirpe0xb9q8qiLsFr0=vr0=vr0dc8meaabaqaciaacaGaaeqabaqabeGadaaakeaadaqadaqaauaabeqaceaaaeaacqaIXaqmcqaI3aWncqaIWaamcqaIZaWmcqaI5aqoaeaacqaIYaGmaaaacaGLOaGaayzkaaaaaa@3402@ = 145, 155, 241 ≈ 1.5·10^8 ^different combinations. Free species contribute another 1215 + 10 + 1 + 1 + 1 = 1228 possible species: 1215 for the free IRS complexes, 10 for all combinations of Shc, Grb2 and SOS and one for insulin, PI3K and SHP2 each.

However, first we analyze complexity on a virtual monomer. The receptor monomer (which consists of an *α*- and a *β*- chain) can bind an insulin molecule and has binding sites for IRS and Shc. Both sites become phosphorylated before effector binding [25]. Shc becomes phosphorylated and binds Grb2. Grb2 can bind SOS, which in turn can be phosphorylated. IRS has four binding sites for PI3K (in fact it has at least nine binding sites for PI3K; each p85 subunit of PI3K occupies two binding sites), one for Grb2 and one for SHP2 [39]. All these binding sites can be phosphorylated. The number of feasible molecular species in this network can be calculated as exemplified in Figure 1. In total there are 145,156,469 possible species. The fact that the insulin receptor and IRS (in fact there exist several different IRS molecules) can be phosphorylated on several regulatory sites and have additional binding partners [25,40,41] is not considered. This accounts for an dramatic further increase of complexity. Even without these additional processes the barriers for detailed modeling are tremendous, as one differential equation is required for the balance of each species. Several other signaling systems show comparable combinatorial complexity.

## Results and discussion

### Introduction of the layer based approach

The two most important mathematical tools to tackle the enormous complexity of models describing biological reaction networks are model reduction and modularization techniques. Here, we introduce a new systematic approach which allows to create considerably reduced models of signaling networks and also provides a new modularization criterion. This new modularization criterion suggests to separate molecular processes (bindings and post-translational modifications) depending on the types of interactions between them.

The basic idea is that three different types of interactions between two processes in signal transduction networks exist. The first interaction type is called all-or-none interaction. In this case, there exists a causal relationship between the two processes, which means that one process must occur before the second process. Most frequently, this kind of interaction is between binding site phosphorylation and effector binding. These two processes can be divided into four molecular events (phosphorylation, dephosphorylation, binding, dissociation). The effector can only bind if the binding site is phosphorylated and dephosphorylation is only possible in the absence of effector. This means that phosphorylation is required for binding and dissociation is required for dephosphorylation.

The second interaction type is called graded interaction. There, two processes influence each other mutually, as it is the case for ligand binding and autophosphorylation of a receptor. This means that kinetic parameters for one process are influenced by the other process. Note that this allows unidirectional interactions, where the first process influences the second, while the second does not influence the first, and bidirectional interactions, where both processes influence each other.

**Figure 2:**
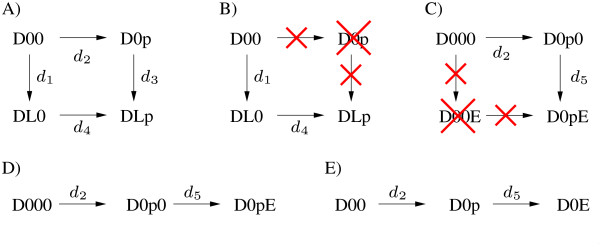
**Graded and all-or-none interactions**. **A) **The reaction cycle of ligand binding and phosphorylation consists of four species that are connected by four reactions. This is a general property of graded interactions. **B) **The processes are coupled via an all-or-none interaction. Therefore the species *DOp *and all corresponding rates do not exist. **C) **All-or-none interaction between binding site phosphorylation and effector binding. The species *DOOE *does not exist. **D) **The reaction cycle degenerates to a reaction chain which is a hallmark of all-or-none interactions. **E) **In this case, nomenclature can be simplified as the two sites on the receptor are essentially one.

The third type are non-interacting, independent processes. They do not influence each other directly. Therefore this type represents in fact no interaction. The concept of all-or-none interactions and graded interactions is demonstrated in Figure 2.

According to our new modularization criterion, no graded interactions are allowed between different modules. Processes of different modules, which we call layers, interact only via all-or-none interactions. Interestingly, the so defined layers only exchange information. The number of molecules in each layer stays constant when no synthesis or degradation is considered. This is a main difference compared to metabolic pathways where mass flows occur and represents the characteristic signal flow within a signal transduction network. Considering the fact that in signaling an extra-cellular signaling molecule causes transmission of a signal to the nucleus of a cell without passing the cell membrane, this difference is most obvious. The signals that are exchanged between layers correspond to a very restricted number of macroscopic variables like levels of occupancy or phosphorylation as they are used by Borisov et al. [22] and Conzelmann et al. [18].

We also contribute a new aspect to the discussion about modularization of biological networks and the optimal criterion for modularization (see also [42-45]). Modularization in our new approach is tightly linked to model reduction. In fact, modularization sets the preconditions to directly postulate the reduced model, without preceding generation of detailed model equations.

In detailed modeling binding or modification events are represented by a huge number of reactions, since the involved proteins can exist in a high number of feasible configurations. The sum of a certain subset of these reaction rates defines a gross reaction rate of binding or modification. The sum of a certain subset of species corresponds to macroscopic quantities as degrees of phosphorylation or occupancy. In the following we show that in most frequent biological scenarios gross rate kinetics can be formulated using macroscopic variables and have a quite simple structure. Mathematical analysis of detailed and reduced models shows that the dynamics of essential macroscopic quantities is highly preserved in most cases. We give qualitative and also some quantitative information about the approximation quality for varying kinetic parameters. A basic advantageous feature of models that are created with the layer based approach presented here is intuitive interpretability. Additionally, preceding generation of detailed model equations is not necessary, as reduced models can be built directly by an intuitive procedure, for which a step by step procedure is given. There exists also a mathematical formalism to derive the reduced model equations. Thus, one can access model generation intuitively or by a mathematical formalism. Since both approaches are equivalent, understanding of the mathematical part is not necessary to create reduced models.

The approach combines qualitative and quantitative system descriptions. The first, qualitative step identifies processes and their interactions to define modules. Inside these modules, processes are described highly reduced using quantitative techniques. All methods of quantitative system analysis then can be applied to the model.

A general problem in modeling of signal transduction is availability of experimental data. Especially kinetic data is difficult to measure. Obviously, the layer based approach has also to face this problem. However, the same kinetic parameters can be taken for the macroscopic quantities that are states of the model as in detailed mechanistic modeling. Therefore, the problem does not become worse when using the reduced modeling technique.

The layer based method can also be combined with the exact domain-oriented lumping technique [18]. As a motivation Table 1, which is discussed later on, shows how many equations are needed to describe the insulin signaling system. Detailed kinetic modeling, exact domain-oriented lumping [18], layer based model reduction and a combination of the reduction techniques are compared in several scenarios.

**Table 1:** Necessary equations for modeling the insulin signaling system

Scenario	Detailed modeling	Exact lumping	Layer based reduction	Combination
1	145 156 468	145 156 468	214	214
2	145 156 468	212	214	56

In two scenarios layer based reduction, domain-oriented lumping and their combination are compared with respect to the number of necessary differential equations. The insulin signaling system is used as example system. Insulin concentration is assumed to be constant. The two binding sites on the receptor for Shc and IRS in each case are assumed to be equivalent. **1)**: All binding site phosphorylations and corresponding bindings are all-or-none interactions. All binding sites on the same molecule can perform graded interactions. **2)**: All binding site phosphorylations and corresponding bindings are all-or-none interactions. The phosphorylation state of binding sites does not influence phosphorylation of other binding sites on the same molecule. See Additional files 3, 4, 5, 6, 7, 8, for model equations and further explanations.

The approach does not have to be used rigorously throughout the model. Subsystems can also be modeled in the detailed formalism, which simplifies integration of existing models.

### A simple example system

**Figure 3:**
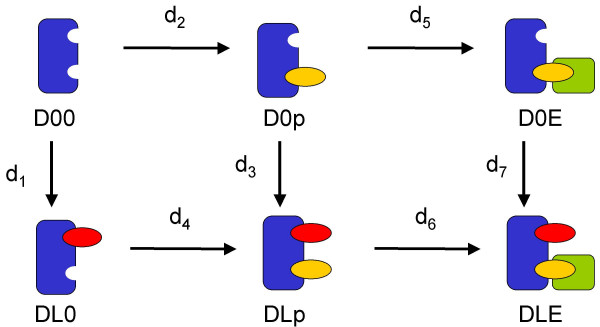
**Visualization of the detailed kinetic model**. All possible reactions of the detailed model for the small example system are shown. The model equations are shown in Equation 1.

In the following the reduction method is presented by introducing general principles, which are exemplified considering a strongly simplified model of insulin receptor signaling (see Figure 3). Afterwards, we will also discuss more complicated examples. The simple insulin model is characterized by the assumption that the receptor only binds one insulin molecule (*L*). Additionally, we only consider one additional binding site, which undergoes autophosphorylation. Afterwards an effector molecule *E *(e.g. IRS) can bind to the receptor. Binding of *E *protects the binding site from dephosphorylation. We do not consider synthesis or degradation of any molecular species. A detailed model of these basic processes comprises eight molecular species and can be described with five ODEs due to the assumption of constant insulin concentration *L *and conservation relations for the receptor *D *and the effector *E*. The reaction network with all occurring reactions is visualized in Figure 3.

d1=k1⋅L⋅DOO−k−1⋅DLOd2=k2⋅DOO−k−2⋅DOpd3=k3⋅L⋅DOp−k−3⋅DLpd4=k4⋅DLO−k−4⋅DLpd5=k5⋅DOp⋅E−k−5⋅DOEd6=k6⋅DLp⋅E−k−6⋅DLEd7=k7⋅L⋅DOE−k−7⋅DLE
 MathType@MTEF@5@5@+=feaafiart1ev1aaatCvAUfKttLearuWrP9MDH5MBPbIqV92AaeXatLxBI9gBaebbnrfifHhDYfgasaacH8akY=wiFfYdH8Gipec8Eeeu0xXdbba9frFj0=OqFfea0dXdd9vqai=hGuQ8kuc9pgc9s8qqaq=dirpe0xb9q8qiLsFr0=vr0=vr0dc8meaabaqaciaacaGaaeqabaqabeGadaaakeaafaqaaeWbbaaaaeaacqWGKbazdaWgaaWcbaGaeGymaedabeaakiabg2da9iabdUgaRnaaBaaaleaacqaIXaqmaeqaaOGaeyyXICTaemitaWKaeyyXICTaemiraqKaem4ta8Kaem4ta8KaeyOeI0Iaem4AaS2aaSbaaSqaaiabgkHiTiabigdaXaqabaGccqGHflY1cqWGebarcqWGmbatcqWGpbWtaeaacqWGKbazdaWgaaWcbaGaeGOmaidabeaakiabg2da9iabdUgaRnaaBaaaleaacqaIYaGmaeqaaOGaeyyXICTaemiraqKaem4ta8Kaem4ta8KaeyOeI0Iaem4AaS2aaSbaaSqaaiabgkHiTiabikdaYaqabaGccqGHflY1cqWGebarcqWGpbWtcqWGWbaCaeaacqWGKbazdaWgaaWcbaGaeG4mamdabeaakiabg2da9iabdUgaRnaaBaaaleaacqaIZaWmaeqaaOGaeyyXICTaemitaWKaeyyXICTaemiraqKaem4ta8KaemiCaaNaeyOeI0Iaem4AaS2aaSbaaSqaaiabgkHiTiabiodaZaqabaGccqGHflY1cqWGebarcqWGmbatcqWGWbaCaeaacqWGKbazdaWgaaWcbaGaeGinaqdabeaakiabg2da9iabdUgaRnaaBaaaleaacqaI0aanaeqaaOGaeyyXICTaemiraqKaemitaWKaem4ta8KaeyOeI0Iaem4AaS2aaSbaaSqaaiabgkHiTiabisda0aqabaGccqGHflY1cqWGebarcqWGmbatcqWGWbaCaeaacqWGKbazdaWgaaWcbaGaeGynaudabeaakiabg2da9iabdUgaRnaaBaaaleaacqaI1aqnaeqaaOGaeyyXICTaemiraqKaem4ta8KaemiCaaNaeyyXICTaemyrauKaeyOeI0Iaem4AaS2aaSbaaSqaaiabgkHiTiabiwda1aqabaGccqGHflY1cqWGebarcqWGpbWtcqWGfbqraeaacqWGKbazdaWgaaWcbaGaeGOnaydabeaakiabg2da9iabdUgaRnaaBaaaleaacqaI2aGnaeqaaOGaeyyXICTaemiraqKaemitaWKaemiCaaNaeyyXICTaemyrauKaeyOeI0Iaem4AaS2aaSbaaSqaaiabgkHiTiabiAda2aqabaGccqGHflY1cqWGebarcqWGmbatcqWGfbqraeaacqWGKbazdaWgaaWcbaGaeG4naCdabeaakiabg2da9iabdUgaRnaaBaaaleaacqaI3aWnaeqaaOGaeyyXICTaemitaWKaeyyXICTaemiraqKaem4ta8KaemyrauKaeyOeI0Iaem4AaS2aaSbaaSqaaiabgkHiTiabiEda3aqabaGccqGHflY1cqWGebarcqWGmbatcqWGfbqraaaaaa@D732@

ddtDOO=−d1−d2ddtDLO=d1−d4ddtDOp=d2−d3−d5ddtDOE=d5−d7ddtDLE=d6+d7
 MathType@MTEF@5@5@+=feaafiart1ev1aaatCvAUfKttLearuWrP9MDH5MBPbIqV92AaeXatLxBI9gBaebbnrfifHhDYfgasaacH8akY=wiFfYdH8Gipec8Eeeu0xXdbba9frFj0=OqFfea0dXdd9vqai=hGuQ8kuc9pgc9s8qqaq=dirpe0xb9q8qiLsFr0=vr0=vr0dc8meaabaqaciaacaGaaeqabaqabeGadaaakeaafaqaaeqbbaaaaeaadaWcaaqaaiabdsgaKbqaaiabdsgaKjabdsha0baacqWGebarcqWGpbWtcqWGpbWtcqGH9aqpcqGHsislcqWGKbazdaWgaaWcbaGaeGymaedabeaakiabgkHiTiabdsgaKnaaBaaaleaacqaIYaGmaeqaaaGcbaWaaSaaaeaacqWGKbazaeaacqWGKbazcqWG0baDaaGaemiraqKaemitaWKaem4ta8Kaeyypa0Jaemizaq2aaSbaaSqaaiabigdaXaqabaGccqGHsislcqWGKbazdaWgaaWcbaGaeGinaqdabeaaaOqaamaalaaabaGaemizaqgabaGaemizaqMaemiDaqhaaiabdseaejabd+eapjabdchaWjabg2da9iabdsgaKnaaBaaaleaacqaIYaGmaeqaaOGaeyOeI0Iaemizaq2aaSbaaSqaaiabiodaZaqabaGccqGHsislcqWGKbazdaWgaaWcbaGaeGynaudabeaaaOqaamaalaaabaGaemizaqgabaGaemizaqMaemiDaqhaaiabdseaejabd+eapjabdweafjabg2da9iabdsgaKnaaBaaaleaacqaI1aqnaeqaaOGaeyOeI0Iaemizaq2aaSbaaSqaaiabiEda3aqabaaakeaadaWcaaqaaiabdsgaKbqaaiabdsgaKjabdsha0baacqWGebarcqWGmbatcqWGfbqrcqGH9aqpcqWGKbazdaWgaaWcbaGaeGOnaydabeaakiabgUcaRiabdsgaKnaaBaaaleaacqaI3aWnaeqaaaaaaaa@7937@

DLp=D0−DOO−DLO−DOp−DOE−DLEE=E0−DOE−DLE
 MathType@MTEF@5@5@+=feaafiart1ev1aaatCvAUfKttLearuWrP9MDH5MBPbIqV92AaeXatLxBI9gBaebbnrfifHhDYfgasaacH8akY=wiFfYdH8Gipec8Eeeu0xXdbba9frFj0=OqFfea0dXdd9vqai=hGuQ8kuc9pgc9s8qqaq=dirpe0xb9q8qiLsFr0=vr0=vr0dc8meaabaqaciaacaGaaeqabaqabeGadaaakeaafaqadeGabaaabaGaemiraqKaemitaWKaemiCaaNaeyypa0Jaemiraq0aaSbaaSqaaiabicdaWaqabaGccqGHsislcqWGebarcqWGpbWtcqWGpbWtcqGHsislcqWGebarcqWGmbatcqWGpbWtcqGHsislcqWGebarcqWGpbWtcqWGWbaCcqGHsislcqWGebarcqWGpbWtcqWGfbqrcqGHsislcqWGebarcqWGmbatcqWGfbqraeaacqWGfbqrcqGH9aqpcqWGfbqrdaWgaaWcbaGaeGimaadabeaakiabgkHiTiabdseaejabd+eapjabdweafjabgkHiTiabdseaejabdYeamjabdweafbaaaaa@55D5@

*D*_0 _and *E*_0 _are the total concentrations of receptor *D *and effector *E*. We denote all receptor states of the *detailed *model by a leading *D*, while later on those of the *reduced *models will be denoted by a leading *R*. In the following we will always assume that detailed kinetic models, which build the base for our consideration of gross rates, are formulated using the law of mass action, which is a frequently used assumption.

### Definitions

#### a) Molecules and complexes

In order to provide a complete and general description of our method, we introduce a formal nomenclature. Due to its generality this nomenclature might appear cumbersome. A simplified nomenclature can be used in most cases because many examples only comprise a small subset of all formally possible cases. In our examples we consistently use a simplified denotation.

Consider a general signaling protein *R*, that provides a number of distinct sites. In the general case each of these sites *i *can be modified from a state *m*_*i, a *_(e.g. not modified) to other states *m*_*i, b *_(e.g. phosphorylated). We denote the molecule with a certain configuration of domain modifications as *D*_*R*_[*m*_1_...*m*_*n*_]. We distinguish between the molecule *D*_*R*_[*m*_1_...*m*_*n*_] and its concentration *D*_*R*_*m*_1_...*m*_*n*_. In a second step we also consider binding of other molecules to a certain binding site of *R*. Such a molecule might be *D*_*E*_[*m*_1_...*m*_*k*_], which also provides a number of sites. One of them can bind to *R*. The result of this association is a complex consisting of *R *and *E*. This shall be denoted as {*D*_*R*_[*m*_1_...*E*...*m*_*n*_], *D*_*E*_[*m*_1_...*R*...*m*_*k*_]}.

The general rule for representation of complexes is as follows. All molecules within a complex are listed comma separated within curly brackets. On each occupied binding site the name of the binding partner is indicated. *A *complex of the molecule *R *binding *E *and *F *would be denoted as {*D*_*R*_[*m*_1_...*E*...*F*...*m*_*n*_], *D*_*E*_[*m*_1_...*R*...*m*_*k*_], *D*_*F*_[*m*_1_...*R*...*m*_*q*_]}. If the same molecule occurs more often than once within the complex, indices have to be used. A graph-oriented molecule representation is suited to solve the problem of correct denotation in difficult cases.

Since our method often works with lumped states comprising a number of molecular species, we introduce the symbol 'X', which is a replacement character for each possible state of a binding site. An 'X' within the site definitions of the molecules indicates all possible modifications on one site. A sequence of three dots following 'X' or before 'X' indicates that there may be additional sites and abbreviates a sequence of 'X'. A sequence of dots between site configurations other than 'X' indicates other sites with distinct configurations (not 'X'). *D*_*R*_[*X*...*P*] for example is a lumped state describing all molecules *R *with a phosphorylated site, no matter in which state all other sites are. Finally, one has to distinguish between *D*_*R *_[*X*...*p*]with a lowercase letter and *D*_*R*_[*X*...*P*] with a capital letter. While *D*_*R*_[*X*...*P*] comprises completely all species with a phosphorylated site, *D*_*R*_[*X*...*p*] only includes those phosphorylated species which do not have bound any other molecule at this site. Thus, if a phosphorylated site cannot bind any other molecule it necessarily is *D*_*R*_[*X*...*p*] = *D*_*R*_[*X*...*P*].

#### b) Rules and reactions

According to Blinov et al. [19], rules are a possibility to condense the description of a reaction system. They correspond to macroscopic chemical reaction equations. Each rule represents a set of chemical reaction equations that have common properties. Using the nomenclature defined above such a reaction rule describing a modification can be written as

*D*_*R*_[*X*...0....*X*] ⇋ *D*_*R*_[*X*....*p*...*X*],

and a binding reaction as

*D*_*R*_[*X*...*p*....*X*] + *D*_*E*_[*X*...*0*...*X*] ⇋ {*D*_*R*_[*X*...*E*....*X*], *D*_*E*_[*X*...*R*...*X*]}.

These rules shall be interpreted as a set of elementary reactions, which all can be modeled using the mass action law. If we consider the simple example defined above a possible reaction rule is

*D*[0, *X*] + *L *⇋ *D*[*L, X*],

which defines all three elementary reactions describing *L *binding to the receptor (*d*_1_, *d*_3 _and *d*_7 _in Equation 1 and Figure 3). Observe that certain sets of elementary reactions may have different representations as reaction rules. As an example, the two rules

D[X,0]⇋D[X,p]D[X,0]⇋D[X,P],
 MathType@MTEF@5@5@+=feaafiart1ev1aaatCvAUfKttLearuWrP9MDH5MBPbIqV92AaeXatLxBI9gBaebbnrfifHhDYfgasaacH8akY=wiFfYdH8Gipec8Eeeu0xXdbba9frFj0=OqFfea0dXdd9vqai=hGuQ8kuc9pgc9s8qqaq=dirpe0xb9q8qiLsFr0=vr0=vr0dc8meaabaqaciaacaGaaeqabaqabeGadaaakeaafaqaaeGabaaabaGaemiraqKaei4waSLaemiwaGLaeiilaWIaeGimaaJaeiyxa01efv3ySLgznfgDOfdaryqr1ngBPrginfgDObYtUvgaiqaacqWFlhYXcqWGebarcqGGBbWwcqWGybawcqGGSaalcqWGWbaCcqGGDbqxaeaacqWGebarcqGGBbWwcqWGybawcqGGSaalcqaIWaamcqGGDbqxcqWFlhYXcqWGebarcqGGBbWwcqWGybawcqGGSaalcqWGqbaucqGGDbqxcqGGSaalaaaaaa@56A7@

are equivalent, since both describe the reactions *d*_2 _and *d*_4 _in Equation 1 and Figure 3. Reaction rules are a short way to formulate the complete and detailed stoichiometry of a reaction network. A short list of reaction rules may correspond to a very long list of possible complexes and elementary reaction steps and thus to large number of model equations.

Our goal is to formulate reduced order models in terms of macroscopic chemical species (denoted by a leading R) and macroscopic gross reactions (denoted by r) converting them. The phosphorylation of a binding site may be described in a reduced manner by the gross reaction

*R*_*R*_*X*..0...*X *⇋ *R*_*R*_*X*...*P*...*X*,

with the rate

*r *= *r*(*R*_*R*_*X*...0...*X*, *R*_*R*_*X*...*P*...*X*).

The corresponding submodel consists of the two ODEs for the macroscopic variables *R*_*R*_*X*...0...*X *and *R*_*R*_*X*...*P*...*X *and thus is much smaller than the model defined by the corresponding reaction rule. The crucial point in model reduction is the derivation of suited gross reaction rates *r*_*j *_in dependence of the concentrations of the macroscopic species. In this work we suggest a method for deriving approximative expressions for gross reaction rates.

#### c) Different types of interactions

As introduced earlier, there exist three structurally different types of interactions. Now we discuss the three types by means of our example (Figure 3). In this example we consider three processes, namely ligand binding/dissociation, receptor phosphorylation/dephosphorylation and effector binding/dissociation. First, we focus on ligand binding and phosphorylation, which we describe in a separate reaction network. These processes form a reaction cycle that is shown in Figure 2A. By choosing the kinetic parameters of the occurring four reactions one determines if and how the two processes interact. Note that a process, e.g. phosphorylation, includes two molecular events, e.g. phosphorylation or dephosphorylation. The same holds for a binding process which consists of the events binding and dissociation.

We start considering the special case of non-interacting processes. If the kinetic parameters describing the reactions *d*_1 _and *d*_3 _are identical and the parameters of *d*_2 _and *d*_4 _are also identical, the two processes – in this case phosphorylation and binding of *L *– are completely independent. Hence, there is no interaction between the two processes. If this condition is not fulfilled, the two processes interact. Characteristics of this interaction can vary in a wide range depending on the corresponding parameters. Due to this great variability we call these interactions *graded *interactions, which form the first important type of interaction. A hallmark of a graded interaction between two processes is that both processes can proceed independently. However, they can influence their kinetic properties mutually.

A structurally different type of interaction is given if there is a causal relationship between the two processes, which means that one processes must occur before the second process. In many cases this probably will only be an approximation to the real interaction. However, it allows to strongly simplify the model. One can assume that receptor phosphorylation only occurs if ligand is bound to the receptor and the species *DOp *never occurs. The reaction network, which is shown in Figure 2B, now is reduced to a reaction chain. In this case *all *receptors follow the remaining reaction path and *none *the deleted one. Hence, we call this type of interaction *all-or-none *interaction. In most examples the assumption that phosphorylation and ligand binding interact in this way will only be a rough approximation. However, if we consider phosphorylation and effector binding the importance of all-or-none interactions becomes apparent. This case is depicted in Figure 2C, where *DOOO *is the unphosphorylated receptor without bound effector *E*. Phosphorylation is indicated by a *p *on the second position, binding of *E *by an *E *on third position. Note, that the species *DOOE *represents species without phosphorylation but with bound *E*. However, phosphorylation is an essential precondition for effector binding and effector binding prevents dephosphorylation due to steric reasons. Therefore, as indicated in Figure 2C, the species *DOOE *and the rates related to it do not exist in this case. Interactions, where both processes can only occur under certain preconditions are denoted as all-or-none interactions. A hallmark of all-or-none interactions is that reaction cycles degenerate to reaction chains due to non-existing species. Most of the occurring all-or-none interactions are interactions between phosphorylations and associated bindings. The reaction scheme in this case can be simplified by omitting non-existing components (Figure 2D) and the notation can be simplified as indicated in Figure 2E and introduced before. This fully simplified notation will be used from now on. Note that all-or-none interactions can be interpreted as a limit case of graded interactions, where certain parameters (e.g. the rate constants for *d*_2 _and *d*_3 _in Figure 2B) are set to zero and therefore reaction cycles degenerate to reaction chains. Analogously non-interacting processes realize another limit case of graded interactions, where there are equality constraints on parameters. However, we define both limit cases not to be graded interactions, since they allow model reduction.

#### d) Modularization: layers

We define that all binding and modification events coupled by graded interactions form a module which we call a layer. Note that the definition of layers is according to interactions of processes and not according to molecules. Roughly speaking, a layer contains processes, not molecules. However, layers are often dominated by the reactions of a single molecule.

**Figure 4:**
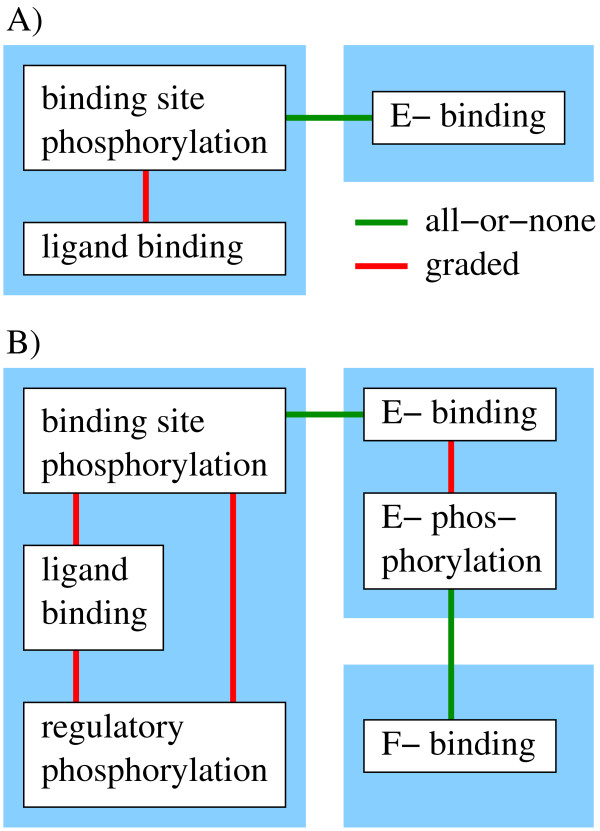
**Interactions define layers**. Processes (white boxes) are coupled by graded (red lines) and all-or-non interactions (green lines). All processes that are coupled by graded interactions are merged into the same module, which is called layer (blue boxes). Therefore, layers are only connected by all-or-none interactions. **A) **A small example system that is discussed in detail. Occurring processes are ligand binding, binding site phosphorylation and effector binding. The reaction scheme is shown in Figure 5. **B) **A larger example system that is discussed in Additional file 4. There, the successive arrangements of layers can be seen.

Diffierent layers are only coupled by all-or-none interactions. The definition of layers and resulting modularization is demonstrated for two example systems in Figure 4. Both example systems are discussed in the article. In the larger system (Figure 4B), binding of an effector molecule influences its phosphorylation through a graded interaction. Binding site phosphorylation and effector binding interact via all-or-none interactions. Note that we also allow all-or-none interactions occurring within layers if the processes that undergo this interaction are linked indirectly by graded interactions.

An important feature of this modularization is that it only depends on the definition of interactions. The introduction of additional graded interactions between processes of different layers leads to disruption of the modular structure. In most cases these layers now form one larger common layer.

The terminus 'layer' results from the typical structure of the modules. As described, usually binding of a signaling molecule and its modifications (usually phosphorylation) form a layer. There is also the possibility that a layer comprises only one process, typically a binding process, as can be seen for *E*-binding in Figure 4A and *F*-binding in Figure 4B. One can imagine the successive layers of signaling that are traversed while the signal propagates through the pathway.

The layers are interconnected by signal flows. This means that information about macroscopic quantities of a layer is exchanged with other layers (signal flow). No mass flows cross layer boundaries. This means, that no reaction exists that transports substance from one layer to the other. Therefore, in the absence of protein synthesis or degradation, the sum of concentrations of all species for each molecule remains constant within the layers. Within layers there are mass flows defined by reaction equations and corresponding rates as in detailed modeling.

Altogether, layer based reduced modeling and modularization results in a highly structured reduced model that is characterized by mass conservation within layers and signal flow between layers.

### Description of the reduction method

First, we consider the more academic case that all processes within a reaction network either do not interact with each other or provide an all-or-none interaction. Afterwards we will consider general networks also including graded interactions. General considerations are illustrated using the simple example defined above.

#### a) Networks without graded interactions

**General considerations: **We assume that within a reaction network all occurring processes do not interact, except domain phosphorylation and subsequent effector bindings that are characterized by all-or-none interactions. Phosphorylation usually can be considered as an essential precondition for binding an effector protein. The reverse modification (dephosphorylation) is prevented by binding of this molecule. Both conditions have to hold, otherwise the interaction between phosphorylation and binding will be graded.

We dissect the pathway into layers as defined above. Since the whole network does not contain any graded interaction, each process can be described in a separate layer. Interestingly, the network has another nice property. All rates *d*_*i *_that describe one of the occurring processes, e.g. ligand binding, are parametrized by the same kinetic constants in the detailed model. The sum of all corresponding rates *d*_*i *_defines a gross rate *r*_*tot *_(Equation 8), which in this case can be interpreted as macroscopic mass action kinetics.

rtot=∑i∈Igrossdi=kon⋅RRX...p...X⋅REX...0...X−koff⋅{RRX...E...X,REX...R...X}
 MathType@MTEF@5@5@+=feaafiart1ev1aaatCvAUfKttLearuWrP9MDH5MBPbIqV92AaeXatLxBI9gBaebbnrfifHhDYfgasaacH8akY=wiFfYdH8Gipec8Eeeu0xXdbba9frFj0=OqFfea0dXdd9vqai=hGuQ8kuc9pgc9s8qqaq=dirpe0xb9q8qiLsFr0=vr0=vr0dc8meaabaqaciaacaGaaeqabaqabeGadaaakeaacqWGYbGCdaWgaaWcbaGaemiDaqNaem4Ba8MaemiDaqhabeaakiabg2da9maaqafabaGaemizaq2aaSbaaSqaaiabdMgaPbqabaGccqGH9aqpcqWGRbWAdaWgaaWcbaGaem4Ba8MaemOBa4gabeaakiabgwSixlabdkfasnaaBaaaleaacqWGsbGuaeqaaOGaemiwaGLaeiOla4IaeiOla4IaeiOla4IaemiCaaNaeiOla4IaeiOla4IaeiOla4IaemiwaGLaeyyXICTaemOuai1aaSbaaSqaaiabdweafbqabaGccqWGybawcqGGUaGlcqGGUaGlcqGGUaGlcqaIWaamcqGGUaGlcqGGUaGlcqGGUaGlcqWGybawaSqaaiabdMgaPjabgIGiolabdMeajnaaBaaameaacqWGNbWzcqWGYbGCcqWGVbWBcqWGZbWCcqWGZbWCaeqaaaWcbeqdcqGHris5aOGaeyOeI0Iaem4AaS2aaSbaaSqaaiabd+gaVjabdAgaMjabdAgaMbqabaGccqGHflY1cqGG7bWEcqWGsbGudaWgaaWcbaGaemOuaifabeaakiabdIfayjabc6caUiabc6caUiabc6caUiabdweafjabc6caUiabc6caUiabc6caUiabdIfayjabcYcaSiabdkfasnaaBaaaleaacqWGfbqraeqaaOGaemiwaGLaeiOla4IaeiOla4IaeiOla4IaemOuaiLaeiOla4IaeiOla4IaeiOla4IaemiwaGLaeiyFa0haaa@886B@

Herein, *I*_*gross *_is the set of all reactions describing the binding of *R *and *E*. Observe that the lumped states of the model are capital letter species *R*_*R *_[*X*...*P*...*X*]. They occur in gross reactions of phosphorylation events. Lowercase species *R*_*R*_[*X*...*p*...*X*] occur in gross reactions of binding events. No ODE is required for lowercase species, as they are defined by conservation relations (Equation 9) between the sum of all phosphorylated binding sites *R*_*R*_*X*...*P*...*X *and the sum of all occupied binding sites {*R*_*R*_*X*...*E*...*X*, *R*_*E*_*X*...*R*...*X*}.

*R*_*R*_*X*...*p*...*X *= *R*_*R*_*X*...*P*...*X *- {*R*_*R*_*X*...*E*...*X*, *R*_*E*_*X*...*R*...*X*}

Obviously, the same considerations can be made concerning modification processes. So, it is only necessary to balance capital letter species.

Gross rates of bindings as well as modifications can be formulated using the mass action formalism, as the parameters of all single elementary processes are equal. This is also discussed by Borisov et al. [22] from another point of view. The resulting model in this special case also corresponds to what the application of the domain-oriented approach provides [18]. Hence, all approaches are exact and consistent in this special case.

**Example: **The example (Figure 3) comprises the three processes ligand binding, binding site phosphorylation and subsequent effector binding. We assume that all reactions describing ligand binding are parametrized by the same kinetic constants. The same holds true for all phosphorylation and all effector binding reactions. Structurally, the system can be dissected into three different layers.

Two binding layers describing ligand and effector binding, and one modification layer describing receptor phosphorylation. Ligand binding can be described by the reaction rule

*D*[0, *X*] + *L *⇋ *D*[*L, X*],

which can be expanded to the reaction rates *d*_1_, *d*_3 _and *d*_7_. Since all of these reactions are parametrized by the same kinetic parameters we combine them all to one single gross reaction

*ROX *+ *L *⇋ *RLX*

with the gross reaction rate

r1=d1+d3+d7=k1⋅(DOO+DOp+DOE︸=ROX)⋅L−k−1⋅(DLO+DLp+DOE︸=RLX).
 MathType@MTEF@5@5@+=feaafiart1ev1aaatCvAUfKttLearuWrP9MDH5MBPbIqV92AaeXatLxBI9gBaebbnrfifHhDYfgasaacH8akY=wiFfYdH8Gipec8Eeeu0xXdbba9frFj0=OqFfea0dXdd9vqai=hGuQ8kuc9pgc9s8qqaq=dirpe0xb9q8qiLsFr0=vr0=vr0dc8meaabaqaciaacaGaaeqabaqabeGadaaakeaacqWGYbGCdaWgaaWcbaGaeGymaedabeaakiabg2da9iabdsgaKnaaBaaaleaacqaIXaqmaeqaaOGaey4kaSIaemizaq2aaSbaaSqaaiabiodaZaqabaGccqGHRaWkcqWGKbazdaWgaaWcbaGaeG4naCdabeaakiabg2da9iabdUgaRnaaBaaaleaacqaIXaqmaeqaaOGaeyyXICTaeiikaGYaaGbaaeaacqWGebarcqWGpbWtcqWGpbWtcqGHRaWkcqWGebarcqWGpbWtcqWGWbaCcqGHRaWkcqWGebarcqWGpbWtcqWGfbqraSqaaiabg2da9iabdkfasjabd+eapjabdIfaybGccaGL44pacqGGPaqkcqGHflY1cqWGmbatcqGHsislcqWGRbWAdaWgaaWcbaGaeyOeI0IaeGymaedabeaakiabgwSixlabcIcaOmaayaaabaGaemiraqKaemitaWKaem4ta8Kaey4kaSIaemiraqKaemitaWKaemiCaaNaey4kaSIaemiraqKaem4ta8KaemyrauealeaacqGH9aqpcqWGsbGucqWGmbatcqWGybawaOGaayjo+dGaeiykaKIaeiOla4caaa@72E1@

The gross reaction

*RX*0 ⇋ *RXP*

describes binding site phosphorylation and corresponds to the reaction rule

*D*[*X*, *O*] ⇋ *D*[*X*, *p*]

which can be expanded to the reaction rates *d*_2 _and *d*_4_.

The corresponding gross reaction rate is given by

r2=k2(DOO+DLO︸=RXO)−k−2(DOp+DLp︸=RXp),
 MathType@MTEF@5@5@+=feaafiart1ev1aaatCvAUfKttLearuWrP9MDH5MBPbIqV92AaeXatLxBI9gBaebbnrfifHhDYfgasaacH8akY=wiFfYdH8Gipec8Eeeu0xXdbba9frFj0=OqFfea0dXdd9vqai=hGuQ8kuc9pgc9s8qqaq=dirpe0xb9q8qiLsFr0=vr0=vr0dc8meaabaqaciaacaGaaeqabaqabeGadaaakeaacqWGYbGCdaWgaaWcbaGaeGOmaidabeaakiabg2da9iabdUgaRnaaBaaaleaacqaIYaGmaeqaaOGaeiikaGYaaGbaaeaacqWGebarcqWGpbWtcqWGpbWtcqGHRaWkcqWGebarcqWGmbatcqWGpbWtaSqaaiabg2da9iabdkfasjabdIfayjabd+eapbGccaGL44pacqGGPaqkcqGHsislcqWGRbWAdaWgaaWcbaGaeyOeI0IaeGOmaidabeaakiabcIcaOmaayaaabaGaemiraqKaem4ta8KaemiCaaNaey4kaSIaemiraqKaemitaWKaemiCaahaleaacqGH9aqpcqWGsbGucqWGybawcqWGWbaCaOGaayjo+dGaeiykaKIaeiilaWcaaa@5893@

in which the lowercase letter concentration *RXp *occurs. However, the macroscopic variable we want to describe is *RXP*. Hence, one has to express *RXp *using macroscopic variables, which in this case is relatively simple. One can use the conservation relation

*RXp *= *RXP *- *RXE*.

The gross reaction

*RXp *+ *E *⇋ *RXE*

describes effector binding and corresponds to the rule

*D*[*X, p*] + *E *⇋ *D*[*X*, *E*],

which can be expanded to the reaction rates *d*_5 _and *d*_6_. The gross reaction rate is

r3=kE⋅(DOp+DLp︸RXp)−k−E⋅(DOE+DLE︸RXE).
 MathType@MTEF@5@5@+=feaafiart1ev1aaatCvAUfKttLearuWrP9MDH5MBPbIqV92AaeXatLxBI9gBaebbnrfifHhDYfgasaacH8akY=wiFfYdH8Gipec8Eeeu0xXdbba9frFj0=OqFfea0dXdd9vqai=hGuQ8kuc9pgc9s8qqaq=dirpe0xb9q8qiLsFr0=vr0=vr0dc8meaabaqaciaacaGaaeqabaqabeGadaaakeaacqWGYbGCdaWgaaWcbaGaeG4mamdabeaakiabg2da9iabdUgaRnaaBaaaleaacqWGfbqraeqaaOGaeyyXICTaeiikaGYaaGbaaeaacqWGebarcqWGpbWtcqWGWbaCcqGHRaWkcqWGebarcqWGmbatcqWGWbaCaSqaaiabdkfasjabdIfayjabdchaWbGccaGL44pacqGGPaqkcqGHsislcqWGRbWAdaWgaaWcbaGaeyOeI0IaemyraueabeaakiabgwSixlabcIcaOmaayaaabaGaemiraqKaem4ta8KaemyrauKaey4kaSIaemiraqKaemitaWKaemyrauealeaacqWGsbGucqWGybawcqWGfbqraOGaayjo+dGaeiykaKIaeiOla4caaa@5B27@

Note that *k*_*E *_= *k*_5 _and *k*_-*E *_= *k*-5. The sum of phosphorylated binding sites *RXP *and the sum of occupied binding sites *RXE *are exchanged between the layers describing phosphorylation and binding. So, *RXp *can be computed in both layers using Equation 16. Using all possible conservation relations

ROX=R0−RLXRXO=R0−RXPE=E0−RXERXp=RXP−RXE
 MathType@MTEF@5@5@+=feaafiart1ev1aaatCvAUfKttLearuWrP9MDH5MBPbIqV92AaeXatLxBI9gBaebbnrfifHhDYfgasaacH8akY=wiFfYdH8Gipec8Eeeu0xXdbba9frFj0=OqFfea0dXdd9vqai=hGuQ8kuc9pgc9s8qqaq=dirpe0xb9q8qiLsFr0=vr0=vr0dc8meaabaqaciaacaGaaeqabaqabeGadaaakeaafaqadeabbaaaaeaacqWGsbGucqWGpbWtcqWGybawcqGH9aqpcqWGsbGudaWgaaWcbaGaeGimaadabeaakiabgkHiTiabdkfasjabdYeamjabdIfaybqaaiabdkfasjabdIfayjabd+eapjabg2da9iabdkfasnaaBaaaleaacqaIWaamaeqaaOGaeyOeI0IaemOuaiLaemiwaGLaemiuaafabaGaemyrauKaeyypa0Jaemyrau0aaSbaaSqaaiabicdaWaqabaGccqGHsislcqWGsbGucqWGybawcqWGfbqraeaacqWGsbGucqWGybawcqWGWbaCcqGH9aqpcqWGsbGucqWGybawcqWGqbaucqGHsislcqWGsbGucqWGybawcqWGfbqraaaaaa@58F7@

with *R*_0 _and *E*_0 _as total concentrations of *R *and *E*, one can eliminate several variables and express all of them using the macroscopic levels of occupancy *RLX*, *RXP *and *RXE*. Remember that ligand concentration *L *is assumed to be constant. Hence, the complete reaction network can be described by only three ODEs.

ddtRLX=r1ddtRXP=r2ddtRXE=r3
 MathType@MTEF@5@5@+=feaafiart1ev1aaatCvAUfKttLearuWrP9MDH5MBPbIqV92AaeXatLxBI9gBaebbnrfifHhDYfgasaacH8akY=wiFfYdH8Gipec8Eeeu0xXdbba9frFj0=OqFfea0dXdd9vqai=hGuQ8kuc9pgc9s8qqaq=dirpe0xb9q8qiLsFr0=vr0=vr0dc8meaabaqaciaacaGaaeqabaqabeGadaaakeaafaqaaeWabaaabaWaaSaaaeaacqWGKbazaeaacqWGKbazcqWG0baDaaGaemOuaiLaemitaWKaemiwaGLaeyypa0JaemOCai3aaSbaaSqaaiabigdaXaqabaaakeaadaWcaaqaaiabdsgaKbqaaiabdsgaKjabdsha0baacqWGsbGucqWGybawcqWGqbaucqGH9aqpcqWGYbGCdaWgaaWcbaGaeGOmaidabeaaaOqaamaalaaabaGaemizaqgabaGaemizaqMaemiDaqhaaiabdkfasjabdIfayjabdweafjabg2da9iabdkhaYnaaBaaaleaacqaIZaWmaeqaaaaaaaa@4E79@

Note that instead of *RXP *or *RXE *we could also choose *RXp *as a state variable of the reduced system. However, as degrees of phosphorylation (e.g. *RXP*) and levels of occupancy (e.g. *RXE*) are usually of interest and correspond to experimental readouts, we generally choose uppercase species as state variables. This has the additional advantage that then there is a conservation relation for each molecule that is not degraded or synthesized within each layer (see Equation 20).

#### b) Networks including all kind of interactions

To introduce the general reduced modeling concept, where all kinds of interactions are allowed, we start with an example to illustrate the main features.

**Example: **Again we consider the simplified insulin model introduced above. Now, we assume that ligand binding unidirectionally influences receptor phosphorylation which in turn is an essential precondition for effector binding. Ligand binding and effector binding do not interact directly. From this it follows that the reaction rates *d*_1_, *d*_3 _and *d*_7 _are all parametrized by the same kinetic rate constants. The same holds true for the reaction rates *d*_5 _and *d*_6_.

**Figure 5:**
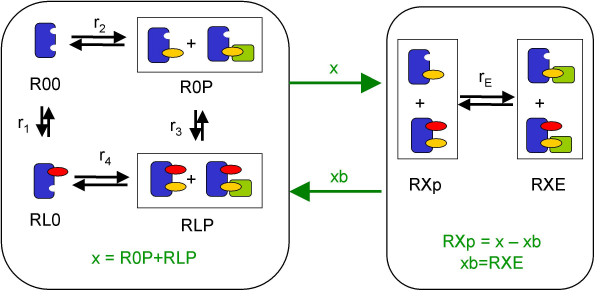
**Visualization of the reduced kinetic model**. In this small example system the left module is the receptor layer which includes ligand binding and phosphorylation of the binding site on the receptor. The right module is the effector layer, where binding of *E *to phosphorylated binding sites on *R *is described. The equations for this system are shown in Equation 26. Note that we could also compute *RXp *in the receptor layer and transfer it to the effector layer instead of *x*. The choice to choose *x *or *RXp *as a transfer variable is up to the modeler. We, however, always transfer *x*, as then macroscopic quantities that correspond to experimental readouts are transfered between the layers.

In the reduced model there are two layers. The receptor layer describes ligand binding and receptor phosphorylation, the effector layer effector binding (Figures 4A and 5). There is a graded interaction within the receptor layer and an all-or-none interaction between the layers. In this simple case there are no graded interactions in the effector layer. The states of the reduced model generally represent lumped states, i.e. they correspond to sums of micro-states or in special cases to single micro-states. In our example *ROO *and *RLO *correspond to micro-states. The lumped states *ROP *and *RLP *are pools for all species which are phosphorylated with no regard to *effector *binding. *RXp *and *RXE *are pools for species with no regard to *ligand *binding.

ROO=DOORLO=DLOROP=DOp+DOERLP=DLp+DLERXp=DOp+DLpRXE=DOE+DLE
 MathType@MTEF@5@5@+=feaafiart1ev1aaatCvAUfKttLearuWrP9MDH5MBPbIqV92AaeXatLxBI9gBaebbnrfifHhDYfgasaacH8akY=wiFfYdH8Gipec8Eeeu0xXdbba9frFj0=OqFfea0dXdd9vqai=hGuQ8kuc9pgc9s8qqaq=dirpe0xb9q8qiLsFr0=vr0=vr0dc8meaabaqaciaacaGaaeqabaqabeGadaaakeaafaqaaeGbdaaaaeaacqWGsbGucqWGpbWtcqWGpbWtaeaacqGH9aqpaeaacqWGebarcqWGpbWtcqWGpbWtaeaacqWGsbGucqWGmbatcqWGpbWtaeaacqGH9aqpaeaacqWGebarcqWGmbatcqWGpbWtaeaacqWGsbGucqWGpbWtcqWGqbauaeaacqGH9aqpaeaacqWGebarcqWGpbWtcqWGWbaCcqGHRaWkcqWGebarcqWGpbWtcqWGfbqraeaacqWGsbGucqWGmbatcqWGqbauaeaacqGH9aqpaeaacqWGebarcqWGmbatcqWGWbaCcqGHRaWkcqWGebarcqWGmbatcqWGfbqraeaacqWGsbGucqWGybawcqWGWbaCaeaacqGH9aqpaeaacqWGebarcqWGpbWtcqWGWbaCcqGHRaWkcqWGebarcqWGmbatcqWGWbaCaeaacqWGsbGucqWGybawcqWGfbqraeaacqGH9aqpaeaacqWGebarcqWGpbWtcqWGfbqrcqGHRaWkcqWGebarcqWGmbatcqWGfbqraaaaaa@6DF6@

Observe, that these six equations are linearly dependent. *RXp *or *RXE *can be expressed by using a conservation relation

*x *= *ROP *+ *RLP *= *RXp *+ *RXE *= *RXP*.

The connection between the two layers, i.e. the information exchange, is given by *x *= *RXP *(Equation 23) and *xb *= *RXE*. The sum of phosphorylated binding sites (*x*) is passed to the effector layer, the sum of occupied binding sites (*xb*) is passed to the receptor layer.

If we compare the reactions of the reduced model (Figure 5) and the reactions of the detailed model (Figure 3), we find

r1=d1r2=d2r3=d3+d7r4=d4rE=d5+d6.
 MathType@MTEF@5@5@+=feaafiart1ev1aaatCvAUfKttLearuWrP9MDH5MBPbIqV92AaeXatLxBI9gBaebbnrfifHhDYfgasaacH8akY=wiFfYdH8Gipec8Eeeu0xXdbba9frFj0=OqFfea0dXdd9vqai=hGuQ8kuc9pgc9s8qqaq=dirpe0xb9q8qiLsFr0=vr0=vr0dc8meaabaqaciaacaGaaeqabaqabeGadaaakeaafaqaaeGadaaabaGaemOCai3aaSbaaSqaaiabigdaXaqabaGccqGH9aqpcqWGKbazdaWgaaWcbaGaeGymaedabeaaaOqaaiabdkhaYnaaBaaaleaacqaIYaGmaeqaaOGaeyypa0Jaemizaq2aaSbaaSqaaiabikdaYaqabaaakeaacqWGYbGCdaWgaaWcbaGaeG4mamdabeaakiabg2da9iabdsgaKnaaBaaaleaacqaIZaWmaeqaaOGaey4kaSIaemizaq2aaSbaaSqaaiabiEda3aqabaaakeaacqWGYbGCdaWgaaWcbaGaeGinaqdabeaakiabg2da9iabdsgaKnaaBaaaleaacqaI0aanaeqaaaGcbaGaemOCai3aaSbaaSqaaiabdweafbqabaGccqGH9aqpcqWGKbazdaWgaaWcbaGaeGynaudabeaakiabgUcaRiabdsgaKnaaBaaaleaacqaI2aGnaeqaaOGaeiOla4cabaaaaaaa@52FD@

As already mentioned, the reaction rates that are merged together (*d*_3 _and *d*_7 _as well as *d*_5 _and *d*_6_) have the same kinetic rate constants.

Our model shall provide equations for all variables that are given in Equation 22. Hence, all the *D*-variables occurring in the gross reaction rates have to be replaced by expressions only comprising *R*-variables.*r*_1_, *r*_3 _and *r*_*E *_can be written using the reduced *R*-states defined above. However, for an exact formulation of the rates *r*_2 _and *r*_4 _one requires the micro-states *DOp *and *DLp*. Due to the linear dependence of Equations 22 an exact reconstruction of these micro-states is not possible. They only can be approximated by

DOp≈ROP−ROPx⋅RXE=x−xbx⋅ROPDLp≈RLP−RLPx⋅RXE=x−xbx⋅RLP,
 MathType@MTEF@5@5@+=feaafiart1ev1aaatCvAUfKttLearuWrP9MDH5MBPbIqV92AaeXatLxBI9gBaebbnrfifHhDYfgasaacH8akY=wiFfYdH8Gipec8Eeeu0xXdbba9frFj0=OqFfea0dXdd9vqai=hGuQ8kuc9pgc9s8qqaq=dirpe0xb9q8qiLsFr0=vr0=vr0dc8meaabaqaciaacaGaaeqabaqabeGadaaakeaafaqabeGabaaabaGaemiraqKaem4ta8KaemiCaaNaeyisISRaemOuaiLaem4ta8KaemiuaaLaeyOeI0YaaSaaaeaacqWGsbGucqWGpbWtcqWGqbauaeaacqWG4baEaaGaeyyXICTaemOuaiLaemiwaGLaemyrauKaeyypa0ZaaSaaaeaacqWG4baEcqGHsislcqWG4baEcqWGIbGyaeaacqWG4baEaaGaeyyXICTaemOuaiLaem4ta8KaemiuaafabaGaemiraqKaemitaWKaemiCaaNaeyisISRaemOuaiLaemitaWKaemiuaaLaeyOeI0YaaSaaaeaacqWGsbGucqWGmbatcqWGqbauaeaacqWG4baEaaGaeyyXICTaemOuaiLaemiwaGLaemyrauKaeyypa0ZaaSaaaeaacqWG4baEcqGHsislcqWG4baEcqWGIbGyaeaacqWG4baEaaGaeyyXICTaemOuaiLaemitaWKaemiuaaLaeiilaWcaaaaa@718F@

with x−xbx=cI
 MathType@MTEF@5@5@+=feaafiart1ev1aaatCvAUfKttLearuWrP9MDH5MBPbIqV92AaeXatLxBI9gBaebbnrfifHhDYfgasaacH8akY=wiFfYdH8Gipec8Eeeu0xXdbba9frFj0=OqFfea0dXdd9vqai=hGuQ8kuc9pgc9s8qqaq=dirpe0xb9q8qiLsFr0=vr0=vr0dc8meaabaqaciaacaGaaeqabaqabeGadaaakeaadaWcaaqaaiabdIha4jabgkHiTiabdIha4jabdkgaIbqaaiabdIha4baacqGH9aqpcqWGJbWydaWgaaWcbaGaemysaKeabeaaaaa@36FD@ (see below, Equation 29). The factor *c*_*I *_is the fraction of unoccupied sites from all phosphorylated sites (bound and unoccupied). Note that *xb *= *RXE*. Thus the rates *r*_2 _and *r*_4 _read as in Equation 26a. As occupied binding sites cannot be dephosphorylated, the sum of occupied binding sites (*xb*) is to be divided on *RLP *and *ROP *for dephosphorylation of those species. This is done according to their relative amount to ensure that only the fraction of *RLP *and *ROP *with unoccupied binding site can become dephosphorylated. Model equations for the reduced system are as follows.

r1=k1⋅L⋅ROO−k−1⋅RLOr2=k2⋅ROO−k−2⋅x−xbx⋅ROPr3=k1⋅L⋅ROP−k−1⋅RLPr4=k4⋅RLO−k−4⋅x−xbx⋅RLPrE=kE⋅RXp⋅E−k−E⋅RXEROO=R0−RLO−ROP−RLP
 MathType@MTEF@5@5@+=feaafiart1ev1aaatCvAUfKttLearuWrP9MDH5MBPbIqV92AaeXatLxBI9gBaebbnrfifHhDYfgasaacH8akY=wiFfYdH8Gipec8Eeeu0xXdbba9frFj0=OqFfea0dXdd9vqai=hGuQ8kuc9pgc9s8qqaq=dirpe0xb9q8qiLsFr0=vr0=vr0dc8meaabaqaciaacaGaaeqabaqabeGadaaakeaafaqaaeGbbaaaaeaacqWGYbGCdaWgaaWcbaGaeGymaedabeaakiabg2da9iabdUgaRnaaBaaaleaacqaIXaqmaeqaaOGaeyyXICTaemitaWKaeyyXICTaemOuaiLaem4ta8Kaem4ta8KaeyOeI0Iaem4AaS2aaSbaaSqaaiabgkHiTiabigdaXaqabaGccqGHflY1cqWGsbGucqWGmbatcqWGpbWtaeaacqWGYbGCdaWgaaWcbaGaeGOmaidabeaakiabg2da9iabdUgaRnaaBaaaleaacqaIYaGmaeqaaOGaeyyXICTaemOuaiLaem4ta8Kaem4ta8KaeyOeI0Iaem4AaS2aaSbaaSqaaiabgkHiTiabikdaYaqabaGccqGHflY1daWcaaqaaiabdIha4jabgkHiTiabdIha4jabdkgaIbqaaiabdIha4baacqGHflY1cqWGsbGucqWGpbWtcqWGqbauaeaacqWGYbGCdaWgaaWcbaGaeG4mamdabeaakiabg2da9iabdUgaRnaaBaaaleaacqaIXaqmaeqaaOGaeyyXICTaemitaWKaeyyXICTaemOuaiLaem4ta8KaemiuaaLaeyOeI0Iaem4AaS2aaSbaaSqaaiabgkHiTiabigdaXaqabaGccqGHflY1cqWGsbGucqWGmbatcqWGqbauaeaacqWGYbGCdaWgaaWcbaGaeGinaqdabeaakiabg2da9iabdUgaRnaaBaaaleaacqaI0aanaeqaaOGaeyyXICTaemOuaiLaemitaWKaem4ta8KaeyOeI0Iaem4AaS2aaSbaaSqaaiabgkHiTiabisda0aqabaGccqGHflY1daWcaaqaaiabdIha4jabgkHiTiabdIha4jabdkgaIbqaaiabdIha4baacqGHflY1cqWGsbGucqWGmbatcqWGqbauaeaacqWGYbGCdaWgaaWcbaGaemyraueabeaakiabg2da9iabdUgaRnaaBaaaleaacqWGfbqraeqaaOGaeyyXICTaemOuaiLaemiwaGLaemiCaaNaeyyXICTaemyrauKaeyOeI0Iaem4AaS2aaSbaaSqaaiabgkHiTiabdweafbqabaGccqGHflY1cqWGsbGucqWGybawcqWGfbqraeaacqWGsbGucqWGpbWtcqWGpbWtcqGH9aqpcqWGsbGudaWgaaWcbaGaeGimaadabeaakiabgkHiTiabdkfasjabdYeamjabd+eapjabgkHiTiabdkfasjabd+eapjabdcfaqjabgkHiTiabdkfasjabdYeamjabdcfaqbaaaaa@CBDD@

     E=E0−RXE     x=ROP+RLP   xb=RXERXp=x−xb
 MathType@MTEF@5@5@+=feaafiart1ev1aaatCvAUfKttLearuWrP9MDH5MBPbIqV92AaeXatLxBI9gBaebbnrfifHhDYfgasaacH8akY=wiFfYdH8Gipec8Eeeu0xXdbba9frFj0=OqFfea0dXdd9vqai=hGuQ8kuc9pgc9s8qqaq=dirpe0xb9q8qiLsFr0=vr0=vr0dc8meaabaqaciaacaGaaeqabaqabeGadaaakeaafaqadeabbaaaaeaacqWGfbqrcqGH9aqpcqWGfbqrdaWgaaWcbaGaeGimaadabeaakiabgkHiTiabdkfasjabdIfayjabdweafbqaaiabdIha4jabg2da9iabdkfasjabd+eapjabdcfaqjabgUcaRiabdkfasjabdYeamjabdcfaqbqaaiabdIha4jabdkgaIjabg2da9iabdkfasjabdIfayjabdweafbqaaiabdkfasjabdIfayjabdchaWjabg2da9iabdIha4jabgkHiTiabdIha4jabdkgaIbaaaaa@5110@

ddtRLO=r1−r4ddtROP=r2−r3ddtRLP=r3+r4ddtRXE=rE
 MathType@MTEF@5@5@+=feaafiart1ev1aaatCvAUfKttLearuWrP9MDH5MBPbIqV92AaeXatLxBI9gBaebbnrfifHhDYfgasaacH8akY=wiFfYdH8Gipec8Eeeu0xXdbba9frFj0=OqFfea0dXdd9vqai=hGuQ8kuc9pgc9s8qqaq=dirpe0xb9q8qiLsFr0=vr0=vr0dc8meaabaqaciaacaGaaeqabaqabeGadaaakeaafaqaaeabbaaaaeaadaWcaaqaaiabdsgaKbqaaiabdsgaKjabdsha0baacqWGsbGucqWGmbatcqWGpbWtcqGH9aqpcqWGYbGCdaWgaaWcbaGaeGymaedabeaakiabgkHiTiabdkhaYnaaBaaaleaacqaI0aanaeqaaaGcbaWaaSaaaeaacqWGKbazaeaacqWGKbazcqWG0baDaaGaemOuaiLaem4ta8KaemiuaaLaeyypa0JaemOCai3aaSbaaSqaaiabikdaYaqabaGccqGHsislcqWGYbGCdaWgaaWcbaGaeG4mamdabeaaaOqaamaalaaabaGaemizaqgabaGaemizaqMaemiDaqhaaiabdkfasjabdYeamjabdcfaqjabg2da9iabdkhaYnaaBaaaleaacqaIZaWmaeqaaOGaey4kaSIaemOCai3aaSbaaSqaaiabisda0aqabaaakeaadaWcaaqaaiabdsgaKbqaaiabdsgaKjabdsha0baacqWGsbGucqWGybawcqWGfbqrcqGH9aqpcqWGYbGCdaWgaaWcbaGaemyraueabeaaaaaaaa@6432@

The approximation bases on the assumption that ligand and effector binding are completely independent.

Accordingly, the calculus of probability suggests that the ratio equation

DOpDLp=DOEDLE⇔DOp⋅DLE−DLp⋅DOE=0
 MathType@MTEF@5@5@+=feaafiart1ev1aaatCvAUfKttLearuWrP9MDH5MBPbIqV92AaeXatLxBI9gBaebbnrfifHhDYfgasaacH8akY=wiFfYdH8Gipec8Eeeu0xXdbba9frFj0=OqFfea0dXdd9vqai=hGuQ8kuc9pgc9s8qqaq=dirpe0xb9q8qiLsFr0=vr0=vr0dc8meaabaqaciaacaGaaeqabaqabeGadaaakeaadaWcaaqaaiabdseaejabd+eapjabdchaWbqaaiabdseaejabdYeamjabdchaWbaacqGH9aqpdaWcaaqaaiabdseaejabd+eapjabdweafbqaaiabdseaejabdYeamjabdweafbaacqGHuhY2cqWGebarcqWGpbWtcqWGWbaCcqGHflY1cqWGebarcqWGmbatcqWGfbqrcqGHsislcqWGebarcqWGmbatcqWGWbaCcqGHflY1cqWGebarcqWGpbWtcqWGfbqrcqGH9aqpcqaIWaamaaa@533B@

is fulfilled [22]. If one uses the five linear independent Equations 22 and this ratio equation it is possible to solve the equations for all micro-states.

DOO=ROODLO=RLODOp=ROP−ROPROP+RLP⋅RXEDLp=RLP−RLPROP+RLP⋅RXEDOE=ROPROP+RLP⋅RXEDLE=RLPROP+RLP⋅RXE
 MathType@MTEF@5@5@+=feaafiart1ev1aaatCvAUfKttLearuWrP9MDH5MBPbIqV92AaeXatLxBI9gBaebbnrfifHhDYfgasaacH8akY=wiFfYdH8Gipec8Eeeu0xXdbba9frFj0=OqFfea0dXdd9vqai=hGuQ8kuc9pgc9s8qqaq=dirpe0xb9q8qiLsFr0=vr0=vr0dc8meaabaqaciaacaGaaeqabaqabeGadaaakeaafaqadeGbbaaaaeaacqWGebarcqWGpbWtcqWGpbWtcqGH9aqpcqWGsbGucqWGpbWtcqWGpbWtaeaacqWGebarcqWGmbatcqWGpbWtcqGH9aqpcqWGsbGucqWGmbatcqWGpbWtaeaacqWGebarcqWGpbWtcqWGWbaCcqGH9aqpcqWGsbGucqWGpbWtcqWGqbaucqGHsisldaWcaaqaaiabdkfasjabd+eapjabdcfaqbqaaiabdkfasjabd+eapjabdcfaqjabgUcaRiabdkfasjabdYeamjabdcfaqbaacqGHflY1cqWGsbGucqWGybawcqWGfbqraeaacqWGebarcqWGmbatcqWGWbaCcqGH9aqpcqWGsbGucqWGmbatcqWGqbaucqGHsisldaWcaaqaaiabdkfasjabdYeamjabdcfaqbqaaiabdkfasjabd+eapjabdcfaqjabgUcaRiabdkfasjabdYeamjabdcfaqbaacqGHflY1cqWGsbGucqWGybawcqWGfbqraeaacqWGebarcqWGpbWtcqWGfbqrcqGH9aqpdaWcaaqaaiabdkfasjabd+eapjabdcfaqbqaaiabdkfasjabd+eapjabdcfaqjabgUcaRiabdkfasjabdYeamjabdcfaqbaacqGHflY1cqWGsbGucqWGybawcqWGfbqraeaacqWGebarcqWGmbatcqWGfbqrcqGH9aqpdaWcaaqaaiabdkfasjabdYeamjabdcfaqbqaaiabdkfasjabd+eapjabdcfaqjabgUcaRiabdkfasjabdYeamjabdcfaqbaacqGHflY1cqWGsbGucqWGybawcqWGfbqraaaaaa@9BB5@

**Figure 6:**
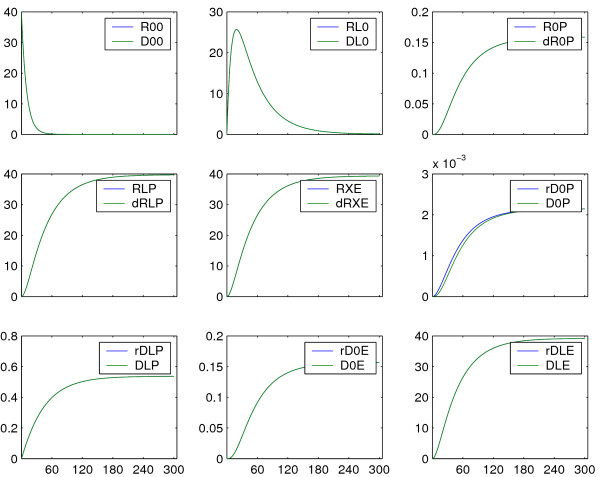
**Simulation results: comparison reduced and detailed model**. For parameter values from literature (Table 2), the deviations of the lumped states from the corresponding sums of states of the detailed model (Equation 22) are negligible. Reconstitution of states of the detailed model is possible with high accuracy. The axis of abscissae is given in *s*, the axis of ordinates in *nM*.

Hence, it is possible to reconstruct all micro-states of the detailed model. The accuracy of this reconstruction highly depends on the validity of the ratio equation (Equation 27). Observe, that an erroneous ratio equation may not have similar strong impact on the accuracy of the reduced model states, like e.g. *RXE*. This depends on the systems ability to damp disturbances, since an erroneous Equation 25 can be interpreted as a disturbance. Simulation with parameter values from literature (Table 2) shows that the deviations of the lumped states from the corresponding sums of states of the detailed model (Equation 22) are negligible. This holds also for variation of each parameter in a wide range (not shown). Reconstitution of states of the detailed model (Equation 28) also provides a high accuracy. Simulation results are shown in Figure 6.

**Table 2:** Kinetic parameters and initial conditions for the small example system

Parameter	Literature value	Unit	Source
*k*_1_	0.001	*nM*^-1^*s*^-1^	[52]
*k*_-1_	4·10^-4^	*s*^-1^	[52]
*k*_2_	0	*s*^-1^	ass.
*k*_-2_	0.00385	*s*^-1^	[51]
*k*_4_	0.0231	*s*^-1^	[50]
*k*_-4_	0.00385	*s*^-1^	[51]
*k*_5_	0.033	*nM*^-1^*s*^-1^	[53]
*k*_-5_	0.113	*s*^-1^	[53]

Initial conditions were 40 *nM *for *DOO*, 250 *nM *for E and 10^-20 ^*nM *for all other species of the detailed model. Initial conditions for the reduced model were chosen consistently (Equations 22 and 28). The insulin concentration is set to 100 *nM*. This is a typical concentration for insulin stimulation. Assumptions are: *k*_1 _= *k*_3 _= *k*_7_, *k*_-1 _= *k*_-3 _= *k*_-7_, *k*_5 _= *k*_6 _= *k*_*E *_and *k*_-5 _= *k*_-6 _= *k*_*-E*_.

**Summary: **Only one variable has actually been eliminated by model reduction, but the example serves to illustrate the main elements of the method. These are:

• Two kinds of real interactions between two processes exist: all-or-none interactions and graded interactions. The third possibility is that these two processes do not interact.

• Modularization is achieved by analyzing interactions between processes. No graded interactions are allowed between modules which are called layers. Layers are only connected by all-or-none interactions.

• Each layer is modeled independently of the others. Gross reactions are formulated that correspond to reactions of macrostates.

• Dephosphorylation reactions of binding sites need special attention, as approximation of lowercase species is necessary. All other reactions can be formulated as in detailed mechanistic modeling, however, using also macroscopic species.

• Concentrations of macroscopic species (or sums of them) are transfered between the layers. Between layers there is only signal flow, but no mass flow.

• Combinatorial complexity is decreased, as all-or-none interactions between layers reduce the number of binding events and bound species by introducing a macroscopic description of the processes.

• Reduced model equations can be obtained directly without previous generation of detailed mechanistic model equations.

• Model equations can be understood and interpreted intuitively. Model variables are macroscopic quantities that are converted by rates following simple kinetics.

**General considerations: **In reaction networks including graded interactions the formulation of gross reaction rates as defined above becomes more difficult than only with all-or-none interactions. We again start by dissecting the whole network into layers. Processes coupled by graded interactions are merged into one layer. All binding and modification processes within a layer must be directly or indirectly linked by graded interactions, while the different layers are only coupled by all-or-none interactions. The coupled layers only exchange information about macroscopic variables, like phosphorylation degrees and levels of occupancy.

Now we assume that the processes of each layer form an isolated network and formulate a detailed kinetic model of solely these processes. Since processes from other layers are neglected in these considerations combinatorial variety is highly reduced. Each state of the submodel can be interpreted as a sum of states of the complete model, and each single reaction in the submodel represents a number of reactions in the complete network. Interestingly, all reactions of the complete network corresponding to a certain reaction in the subnetwork are parameterized by the same kinetic parameters. The reason for this can be found in the definition of layers. Since there are no graded interactions between layers, alterations in other layers do not change the kinetic properties inside the considered layer.

Observe, that we define the reactions of the isolated partial network as gross reactions. We stated earlier that if all reactions forming a gross reaction have the same kinetic parameters it can be formulated using the law of mass action. However, one has to accommodate these rates by including mass conservation relations to eliminate lowercase species from the description (as in Equation 9). As processes within a layer can be modeled separately each layer has to fulfill certain conservation relations. The receptor layer e.g. is characterized by a conservation relation for *R*. Due to these conservation relations within each layer the gross reactions for phosphorylations have to be formulated with capital letter concentrations like *RX*...*P*...*X*, which also comprise all phosphorylated species with additionally bound effectors. Gross rate kinetics are nevertheless characterized by lowercase concentrations. However, a mass conservation relation as Equation 9 does usually not allow to replace the lowercase concentrations by macroscopic variables. This results from the presence of graded interactions within the layers. If binding site phosphorylation is influenced by other processes of the same layer, e.g. ligand binding, more than one gross rate is needed to describe binding site phosphorylation. Therefore, the mass conservation relation as Equation 9 does only provide information about the sum of concentrations of species that may be dephosphorylated. However, we can formulate approximative equations

*R*_*R*_*M*_1_...*p*...*M*_*n *_≈ *c*_*I*_·*R*_*R*_*M*_1_....*P*....*M*_*n*_,   0 ≤ *c*_*I *_≤ 1

where *c*_*I *_is a correction term that is the fraction of phosphorylated binding sites that is unoccupied.

cI=RRX...P...X−{RRX...E...X,REX...R...X}RRX...P...X=RRX...p...XRRX...P...X=xi−xibxi
 MathType@MTEF@5@5@+=feaafiart1ev1aaatCvAUfKttLearuWrP9MDH5MBPbIqV92AaeXatLxBI9gBaebbnrfifHhDYfgasaacH8akY=wiFfYdH8Gipec8Eeeu0xXdbba9frFj0=OqFfea0dXdd9vqai=hGuQ8kuc9pgc9s8qqaq=dirpe0xb9q8qiLsFr0=vr0=vr0dc8meaabaqaciaacaGaaeqabaqabeGadaaakeaafaqadeGabaaabaGaem4yam2aaSbaaSqaaiabdMeajbqabaGccqGH9aqpdaWcaaqaaiabdkfasnaaBaaaleaacqWGsbGuaeqaaOGaemiwaGLaeiOla4IaeiOla4IaeiOla4IaemiuaaLaeiOla4IaeiOla4IaeiOla4IaemiwaGLaeyOeI0Iaei4EaSNaemOuai1aaSbaaSqaaiabdkfasbqabaGccqWGybawcqGGUaGlcqGGUaGlcqGGUaGlcqWGfbqrcqGGUaGlcqGGUaGlcqGGUaGlcqWGybawcqGGSaalcqWGsbGudaWgaaWcbaGaemyraueabeaakiabdIfayjabc6caUiabc6caUiabc6caUiabdkfasjabc6caUiabc6caUiabc6caUiabdIfayjabc2ha9bqaaiabdkfasnaaBaaaleaacqWGsbGuaeqaaOGaemiwaGLaeiOla4IaeiOla4IaeiOla4IaemiuaaLaeiOla4IaeiOla4IaeiOla4IaemiwaGfaaaqaaiabg2da9maalaaabaGaemOuai1aaSbaaSqaaiabdkfasbqabaGccqWGybawcqGGUaGlcqGGUaGlcqGGUaGlcqWGWbaCcqGGUaGlcqGGUaGlcqGGUaGlcqWGybawaeaacqWGsbGudaWgaaWcbaGaemOuaifabeaakiabdIfayjabc6caUiabc6caUiabc6caUiabdcfaqjabc6caUiabc6caUiabc6caUiabdIfaybaacqGH9aqpdaWcaaqaaiabdIha4naaBaaaleaacqWGPbqAaeqaaOGaeyOeI0IaemiEaG3aaSbaaSqaaiabdMgaPbqabaGccqWGIbGyaeaacqWG4baEdaWgaaWcbaGaemyAaKgabeaaaaaaaaaa@87DE@

It is assumed that this fraction is identical for all species *R*_*R*_[*M*_1_....*P*....*M*_*n*_]. Note that the factor *c*_*I *_is time dependent as the fraction of phosphorylated binding sites that is unoccupied may change over time. A detailed discussion of approximation quality will be given in the mathematical background section. The considered gross rates for the phosphorylation of *R *can be formulated using the law of mass action, if the resulting rate equations are accommodated using correction terms *c*_*I *_(Equations 29 and 30).

#### Consistent initial values

When comparing the reduced and detailed models the initial conditions have to be chosen that transformation equations hold for the starting point. This is guaranteed for the example system (Figure 5) if Equation 27 holds. If there is no detailed model, choice of consistent initial values also is of great importance. Without caring about this problem simulation can end up with negative concentrations, e.g. if initial concentrations satisfy *RXE *> *ROP *+ *RLP*. Effector binding requires previous receptor phosphorylation and the receptor cannot be dephosphorylated while an effector is bound. Therefore, the number of phosphorylated receptors always has to be greater than or equal to the number of receptor-effector complexes. In general, for each connection between layers there is one inequality constraint, e.g. *RXE *≤ *ROP *+ *RLP*. Ignoring them results in physically infeasible systems. So there are two general possibilities to avoid infeasible systems. The first is to start with initial conditions for the detailed model and transform them into initial conditions for the reduced model (Equation 22). The other possibility is to ensure that the inequality constraints hold for each connection between layers.

Another aspect of choosing proper initial conditions arises from approximation of lowercase species (Equation 29). As there is division by *R*_*R*_*X*...*P*...*X *to calculate *c*_*I *_(Equation 30), all *R*_*R*_*X*...*P*...*X*, where *P *represents phosphorylation of a binding site, have to be larger than zero. However, if *R*_*R*_*X*...*P*...*X *equals zero, *R*_*R*_*X*...*p*...*X *also equals zero. This results in an 0/0 case, for which the limit is one. As this may be problematic during model simulation we recommend to choose initial conditions for all *R*_*R*_*X*...*P*...*X *larger than zero. Values very close to zero can be taken, if zero is the desired initial condition.

### Mathematical background

In this section we analyze the presented reduced modeling method from a mathematical point of view. First, we introduce some general mathematical considerations about model reduction. Afterwards we will show that the layer based reduction method also fits into this general procedure. This will help us to evaluate the method and make statements about approximation errors.

#### General considerations

The layer based approach allows to directly generate reduced model equations, a step by step procedure for this is given after the mathematical background. However, in common model reduction techniques the starting point is a detailed mechanistic model of the form

x→˙=f→(x→,u→),y→=h→(x→).
 MathType@MTEF@5@5@+=feaafiart1ev1aaatCvAUfKttLearuWrP9MDH5MBPbIqV92AaeXatLxBI9gBaebbnrfifHhDYfgasaacH8akY=wiFfYdH8Gipec8Eeeu0xXdbba9frFj0=OqFfea0dXdd9vqai=hGuQ8kuc9pgc9s8qqaq=dirpe0xb9q8qiLsFr0=vr0=vr0dc8meaabaqaciaacaGaaeqabaqabeGadaaakeaafaqabeqacaaabaGafmiEaGNbaSGbaiaacqGH9aqpcuWGMbGzgaWcaiabcIcaOiqbdIha4zaalaGaeiilaWIafmyDauNbaSaacqGGPaqkcqGGSaalaeaacuWG5bqEgaWcaiabg2da9iqbdIgaOzaalaGaeiikaGIafmiEaGNbaSaacqGGPaqkaaGaeiOla4caaa@3F5A@

Herein x→
 MathType@MTEF@5@5@+=feaafiart1ev1aaatCvAUfKttLearuWrP9MDH5MBPbIqV92AaeXatLxBI9gBaebbnrfifHhDYfgasaacH8akY=wiFfYdH8Gipec8Eeeu0xXdbba9frFj0=OqFfea0dXdd9vqai=hGuQ8kuc9pgc9s8qqaq=dirpe0xb9q8qiLsFr0=vr0=vr0dc8meaabaqaciaacaGaaeqabaqabeGadaaakeaacuWG4baEgaWcaaaa@2E37@ ∈ **R**^*n *^denotes the state vector, u→
 MathType@MTEF@5@5@+=feaafiart1ev1aaatCvAUfKttLearuWrP9MDH5MBPbIqV92AaeXatLxBI9gBaebbnrfifHhDYfgasaacH8akY=wiFfYdH8Gipec8Eeeu0xXdbba9frFj0=OqFfea0dXdd9vqai=hGuQ8kuc9pgc9s8qqaq=dirpe0xb9q8qiLsFr0=vr0=vr0dc8meaabaqaciaacaGaaeqabaqabeGadaaakeaacuWG1bqDgaWcaaaa@2E31@ ∈ **R**^*m *^the system inputs and y→
 MathType@MTEF@5@5@+=feaafiart1ev1aaatCvAUfKttLearuWrP9MDH5MBPbIqV92AaeXatLxBI9gBaebbnrfifHhDYfgasaacH8akY=wiFfYdH8Gipec8Eeeu0xXdbba9frFj0=OqFfea0dXdd9vqai=hGuQ8kuc9pgc9s8qqaq=dirpe0xb9q8qiLsFr0=vr0=vr0dc8meaabaqaciaacaGaaeqabaqabeGadaaakeaacuWG5bqEgaWcaaaa@2E39@ ∈ **R**^*q *^the system outputs. Now, the objective is to find another mathematical representation of the dynamic model which allows to approximately describe the output variables by a reduced state vector. In order to achieve this reduction one has to transform the original dynamic system to new coordinates z→=φ→(x→)
 MathType@MTEF@5@5@+=feaafiart1ev1aaatCvAUfKttLearuWrP9MDH5MBPbIqV92AaeXatLxBI9gBaebbnrfifHhDYfgasaacH8akY=wiFfYdH8Gipec8Eeeu0xXdbba9frFj0=OqFfea0dXdd9vqai=hGuQ8kuc9pgc9s8qqaq=dirpe0xb9q8qiLsFr0=vr0=vr0dc8meaabaqaciaacaGaaeqabaqabeGadaaakeaacuWG6bGEgaWcaiabg2da9GGaciqb=z8aMzaalaGaeiikaGIafmiEaGNbaSaacqGGPaqkaaa@3450@. First, we require that this generally nonlinear transformation is a diffeomorphism, which means that the function φ→
 MathType@MTEF@5@5@+=feaafiart1ev1aaatCvAUfKttLearuWrP9MDH5MBPbIqV92AaeXatLxBI9gBaebbnrfifHhDYfgasaacH8akY=wiFfYdH8Gipec8Eeeu0xXdbba9frFj0=OqFfea0dXdd9vqai=hGuQ8kuc9pgc9s8qqaq=dirpe0xb9q8qiLsFr0=vr0=vr0dc8meaabaqaciaacaGaaeqabaqabeGadaaakeaaiiGacuWFgpGzgaWcaaaa@2E7E@ is invertible and smooth. Additionally, we require that the transformation separates the states that have a strong impact on the output variables from the states that only have little influence on them. The first part of the transformed state z→
 MathType@MTEF@5@5@+=feaafiart1ev1aaatCvAUfKttLearuWrP9MDH5MBPbIqV92AaeXatLxBI9gBaebbnrfifHhDYfgasaacH8akY=wiFfYdH8Gipec8Eeeu0xXdbba9frFj0=OqFfea0dXdd9vqai=hGuQ8kuc9pgc9s8qqaq=dirpe0xb9q8qiLsFr0=vr0=vr0dc8meaabaqaciaacaGaaeqabaqabeGadaaakeaacuWG6bGEgaWcaaaa@2E3B@ shall be the states of the reduced model z→k
 MathType@MTEF@5@5@+=feaafiart1ev1aaatCvAUfKttLearuWrP9MDH5MBPbIqV92AaeXatLxBI9gBaebbnrfifHhDYfgasaacH8akY=wiFfYdH8Gipec8Eeeu0xXdbba9frFj0=OqFfea0dXdd9vqai=hGuQ8kuc9pgc9s8qqaq=dirpe0xb9q8qiLsFr0=vr0=vr0dc8meaabaqaciaacaGaaeqabaqabeGadaaakeaacuWG6bGEgaWcamaaBaaaleaacqWGRbWAaeqaaaaa@2FC6@. The other set of states z→n
 MathType@MTEF@5@5@+=feaafiart1ev1aaatCvAUfKttLearuWrP9MDH5MBPbIqV92AaeXatLxBI9gBaebbnrfifHhDYfgasaacH8akY=wiFfYdH8Gipec8Eeeu0xXdbba9frFj0=OqFfea0dXdd9vqai=hGuQ8kuc9pgc9s8qqaq=dirpe0xb9q8qiLsFr0=vr0=vr0dc8meaabaqaciaacaGaaeqabaqabeGadaaakeaacuWG6bGEgaWcamaaBaaaleaacqWGUbGBaeqaaaaa@2FCC@ shall be eliminated in the reduced model. Hence, the transformed system can be written as

z→˙=[z→˙kz→˙n]=[g→1(z→k,z→n,u→)g→2(z→k,z→n,u→)].y→∗=h→∗(z→k)≈y→=h→(x→)
 MathType@MTEF@5@5@+=feaafiart1ev1aaatCvAUfKttLearuWrP9MDH5MBPbIqV92AaeXatLxBI9gBaebbnrfifHhDYfgasaacH8akY=wiFfYdH8Gipec8Eeeu0xXdbba9frFj0=OqFfea0dXdd9vqai=hGuQ8kuc9pgc9s8qqaq=dirpe0xb9q8qiLsFr0=vr0=vr0dc8meaabaqaciaacaGaaeqabaqabeGadaaakeaafaqabeGabaaabaGafmOEaONbaSGbaiaacqGH9aqpdaWadaqaauaabeqaceaaaeaacuWG6bGEgaWcgaGaamaaBaaaleaacqWGRbWAaeqaaaGcbaGafmOEaONbaSGbaiaadaWgaaWcbaGaemOBa4gabeaaaaaakiaawUfacaGLDbaacqGH9aqpdaWadaqaauaabeqaceaaaeaacuWGNbWzgaWcamaaBaaaleaacqaIXaqmaeqaaOGaeiikaGIafmOEaONbaSaadaWgaaWcbaGaem4AaSgabeaakiabcYcaSiqbdQha6zaalaWaaSbaaSqaaiabd6gaUbqabaGccqGGSaalcuWG1bqDgaWcaiabcMcaPaqaaiqbdEgaNzaalaWaaSbaaSqaaiabikdaYaqabaGccqGGOaakcuWG6bGEgaWcamaaBaaaleaacqWGRbWAaeqaaOGaeiilaWIafmOEaONbaSaadaWgaaWcbaGaemOBa4gabeaakiabcYcaSiqbdwha1zaalaGaeiykaKcaaaGaay5waiaaw2faaiabc6caUaqaaiqbdMha5zaalaWaaWbaaSqabeaacqGHxiIkaaGccqGH9aqpcuWGObaAgaWcamaaCaaaleqabaGaey4fIOcaaOGaeiikaGIafmOEaONbaSaadaWgaaWcbaGaem4AaSgabeaakiabcMcaPiabgIKi7kqbdMha5zaalaGaeyypa0JafmiAaGMbaSaacqGGOaakcuWG4baEgaWcaiabcMcaPaaaaaa@6B52@

If g→1
 MathType@MTEF@5@5@+=feaafiart1ev1aaatCvAUfKttLearuWrP9MDH5MBPbIqV92AaeXatLxBI9gBaebbnrfifHhDYfgasaacH8akY=wiFfYdH8Gipec8Eeeu0xXdbba9frFj0=OqFfea0dXdd9vqai=hGuQ8kuc9pgc9s8qqaq=dirpe0xb9q8qiLsFr0=vr0=vr0dc8meaabaqaciaacaGaaeqabaqabeGadaaakeaacuWGNbWzgaWcamaaBaaaleaacqaIXaqmaeqaaaaa@2F31@ and h→*
 MathType@MTEF@5@5@+=feaafiart1ev1aaatCvAUfKttLearuWrP9MDH5MBPbIqV92AaeXatLxBI9gBaebbnrfifHhDYfgasaacH8akY=wiFfYdH8Gipec8Eeeu0xXdbba9frFj0=OqFfea0dXdd9vqai=hGuQ8kuc9pgc9s8qqaq=dirpe0xb9q8qiLsFr0=vr0=vr0dc8meaabaqaciaacaGaaeqabaqabeGadaaakeaacuWGObaAgaWcamaaCaaaleqabaGaeiOkaOcaaaaa@2F20@ only depend on z→k
 MathType@MTEF@5@5@+=feaafiart1ev1aaatCvAUfKttLearuWrP9MDH5MBPbIqV92AaeXatLxBI9gBaebbnrfifHhDYfgasaacH8akY=wiFfYdH8Gipec8Eeeu0xXdbba9frFj0=OqFfea0dXdd9vqai=hGuQ8kuc9pgc9s8qqaq=dirpe0xb9q8qiLsFr0=vr0=vr0dc8meaabaqaciaacaGaaeqabaqabeGadaaakeaacuWG6bGEgaWcamaaBaaaleaacqWGRbWAaeqaaaaa@2FC6@ and not on z→n
 MathType@MTEF@5@5@+=feaafiart1ev1aaatCvAUfKttLearuWrP9MDH5MBPbIqV92AaeXatLxBI9gBaebbnrfifHhDYfgasaacH8akY=wiFfYdH8Gipec8Eeeu0xXdbba9frFj0=OqFfea0dXdd9vqai=hGuQ8kuc9pgc9s8qqaq=dirpe0xb9q8qiLsFr0=vr0=vr0dc8meaabaqaciaacaGaaeqabaqabeGadaaakeaacuWG6bGEgaWcamaaBaaaleaacqWGUbGBaeqaaaaa@2FCC@ the equations for z→n
 MathType@MTEF@5@5@+=feaafiart1ev1aaatCvAUfKttLearuWrP9MDH5MBPbIqV92AaeXatLxBI9gBaebbnrfifHhDYfgasaacH8akY=wiFfYdH8Gipec8Eeeu0xXdbba9frFj0=OqFfea0dXdd9vqai=hGuQ8kuc9pgc9s8qqaq=dirpe0xb9q8qiLsFr0=vr0=vr0dc8meaabaqaciaacaGaaeqabaqabeGadaaakeaacuWG6bGEgaWcamaaBaaaleaacqWGUbGBaeqaaaaa@2FCC@ can simply be omitted. Then it is guaranteed that the reduced model has exactly the same input/output behavior as the complete model. However, such an exact reduction is only possible in a restricted number of cases [18,22]. Generally, the vector field g→1
 MathType@MTEF@5@5@+=feaafiart1ev1aaatCvAUfKttLearuWrP9MDH5MBPbIqV92AaeXatLxBI9gBaebbnrfifHhDYfgasaacH8akY=wiFfYdH8Gipec8Eeeu0xXdbba9frFj0=OqFfea0dXdd9vqai=hGuQ8kuc9pgc9s8qqaq=dirpe0xb9q8qiLsFr0=vr0=vr0dc8meaabaqaciaacaGaaeqabaqabeGadaaakeaacuWGNbWzgaWcamaaBaaaleaacqaIXaqmaeqaaaaa@2F31@ also will depend on z→n
 MathType@MTEF@5@5@+=feaafiart1ev1aaatCvAUfKttLearuWrP9MDH5MBPbIqV92AaeXatLxBI9gBaebbnrfifHhDYfgasaacH8akY=wiFfYdH8Gipec8Eeeu0xXdbba9frFj0=OqFfea0dXdd9vqai=hGuQ8kuc9pgc9s8qqaq=dirpe0xb9q8qiLsFr0=vr0=vr0dc8meaabaqaciaacaGaaeqabaqabeGadaaakeaacuWG6bGEgaWcamaaBaaaleaacqWGUbGBaeqaaaaa@2FCC@. Since the dynamics of the states z→k
 MathType@MTEF@5@5@+=feaafiart1ev1aaatCvAUfKttLearuWrP9MDH5MBPbIqV92AaeXatLxBI9gBaebbnrfifHhDYfgasaacH8akY=wiFfYdH8Gipec8Eeeu0xXdbba9frFj0=OqFfea0dXdd9vqai=hGuQ8kuc9pgc9s8qqaq=dirpe0xb9q8qiLsFr0=vr0=vr0dc8meaabaqaciaacaGaaeqabaqabeGadaaakeaacuWG6bGEgaWcamaaBaaaleaacqWGRbWAaeqaaaaa@2FC6@ is still influenced by the states z→n
 MathType@MTEF@5@5@+=feaafiart1ev1aaatCvAUfKttLearuWrP9MDH5MBPbIqV92AaeXatLxBI9gBaebbnrfifHhDYfgasaacH8akY=wiFfYdH8Gipec8Eeeu0xXdbba9frFj0=OqFfea0dXdd9vqai=hGuQ8kuc9pgc9s8qqaq=dirpe0xb9q8qiLsFr0=vr0=vr0dc8meaabaqaciaacaGaaeqabaqabeGadaaakeaacuWG6bGEgaWcamaaBaaaleaacqWGUbGBaeqaaaaa@2FCC@, these have to be approximated by the states z→k
 MathType@MTEF@5@5@+=feaafiart1ev1aaatCvAUfKttLearuWrP9MDH5MBPbIqV92AaeXatLxBI9gBaebbnrfifHhDYfgasaacH8akY=wiFfYdH8Gipec8Eeeu0xXdbba9frFj0=OqFfea0dXdd9vqai=hGuQ8kuc9pgc9s8qqaq=dirpe0xb9q8qiLsFr0=vr0=vr0dc8meaabaqaciaacaGaaeqabaqabeGadaaakeaacuWG6bGEgaWcamaaBaaaleaacqWGRbWAaeqaaaaa@2FC6@. If such an approximation z→n≈ψ→(z→k)
 MathType@MTEF@5@5@+=feaafiart1ev1aaatCvAUfKttLearuWrP9MDH5MBPbIqV92AaeXatLxBI9gBaebbnrfifHhDYfgasaacH8akY=wiFfYdH8Gipec8Eeeu0xXdbba9frFj0=OqFfea0dXdd9vqai=hGuQ8kuc9pgc9s8qqaq=dirpe0xb9q8qiLsFr0=vr0=vr0dc8meaabaqaciaacaGaaeqabaqabeGadaaakeaacuWG6bGEgaWcamaaBaaaleaacqWGUbGBaeqaaOGaeyisISlcciGaf8hYdKNbaSaacqGGOaakcuWG6bGEgaWcamaaBaaaleaacqWGRbWAaeqaaOGaeiykaKcaaa@3844@ is found the reduced model can be written as

z→˙k=g→1(z→k,ψ→(z→k),u→)y→∗=h→∗(z→k).
 MathType@MTEF@5@5@+=feaafiart1ev1aaatCvAUfKttLearuWrP9MDH5MBPbIqV92AaeXatLxBI9gBaebbnrfifHhDYfgasaacH8akY=wiFfYdH8Gipec8Eeeu0xXdbba9frFj0=OqFfea0dXdd9vqai=hGuQ8kuc9pgc9s8qqaq=dirpe0xb9q8qiLsFr0=vr0=vr0dc8meaabaqaciaacaGaaeqabaqabeGadaaakeaafaqaaeGabaaabaGafmOEaONbaSGbaiaadaWgaaWcbaGaem4AaSgabeaakiabg2da9iqbdEgaNzaalaWaaSbaaSqaaiabigdaXaqabaGccqGGOaakcuWG6bGEgaWcamaaBaaaleaacqWGRbWAaeqaaOGaeiilaWccciGaf8hYdKNbaSaacqGGOaakcuWG6bGEgaWcamaaBaaaleaacqWGRbWAaeqaaOGaeiykaKIaeiilaWIafmyDauNbaSaacqGGPaqkaeaacuWG5bqEgaWcamaaCaaaleqabaGaey4fIOcaaOGaeyypa0JafmiAaGMbaSaadaahaaWcbeqaaiabgEHiQaaakiabcIcaOiqbdQha6zaalaWaaSbaaSqaaiabdUgaRbqabaGccqGGPaqkcqGGUaGlaaaaaa@4E55@

#### The relevant states

Now, we show that the layer based reduction method fits into the previously introduced general pattern of model reduction. Hence, we first have to define the two set of states z→k
 MathType@MTEF@5@5@+=feaafiart1ev1aaatCvAUfKttLearuWrP9MDH5MBPbIqV92AaeXatLxBI9gBaebbnrfifHhDYfgasaacH8akY=wiFfYdH8Gipec8Eeeu0xXdbba9frFj0=OqFfea0dXdd9vqai=hGuQ8kuc9pgc9s8qqaq=dirpe0xb9q8qiLsFr0=vr0=vr0dc8meaabaqaciaacaGaaeqabaqabeGadaaakeaacuWG6bGEgaWcamaaBaaaleaacqWGRbWAaeqaaaaa@2FC6@ and z→n
 MathType@MTEF@5@5@+=feaafiart1ev1aaatCvAUfKttLearuWrP9MDH5MBPbIqV92AaeXatLxBI9gBaebbnrfifHhDYfgasaacH8akY=wiFfYdH8Gipec8Eeeu0xXdbba9frFj0=OqFfea0dXdd9vqai=hGuQ8kuc9pgc9s8qqaq=dirpe0xb9q8qiLsFr0=vr0=vr0dc8meaabaqaciaacaGaaeqabaqabeGadaaakeaacuWG6bGEgaWcamaaBaaaleaacqWGUbGBaeqaaaaa@2FCC@ implying the transformation φ→(x→)
 MathType@MTEF@5@5@+=feaafiart1ev1aaatCvAUfKttLearuWrP9MDH5MBPbIqV92AaeXatLxBI9gBaebbnrfifHhDYfgasaacH8akY=wiFfYdH8Gipec8Eeeu0xXdbba9frFj0=OqFfea0dXdd9vqai=hGuQ8kuc9pgc9s8qqaq=dirpe0xb9q8qiLsFr0=vr0=vr0dc8meaabaqaciaacaGaaeqabaqabeGadaaakeaaiiGacuWFgpGzgaWcaiabcIcaOiqbdIha4zaalaGaeiykaKcaaa@31BB@. Second, we have to define algebraic equations that can be used to approximate the states z→n
 MathType@MTEF@5@5@+=feaafiart1ev1aaatCvAUfKttLearuWrP9MDH5MBPbIqV92AaeXatLxBI9gBaebbnrfifHhDYfgasaacH8akY=wiFfYdH8Gipec8Eeeu0xXdbba9frFj0=OqFfea0dXdd9vqai=hGuQ8kuc9pgc9s8qqaq=dirpe0xb9q8qiLsFr0=vr0=vr0dc8meaabaqaciaacaGaaeqabaqabeGadaaakeaacuWG6bGEgaWcamaaBaaaleaacqWGUbGBaeqaaaaa@2FCC@. The definition of the states z→k
 MathType@MTEF@5@5@+=feaafiart1ev1aaatCvAUfKttLearuWrP9MDH5MBPbIqV92AaeXatLxBI9gBaebbnrfifHhDYfgasaacH8akY=wiFfYdH8Gipec8Eeeu0xXdbba9frFj0=OqFfea0dXdd9vqai=hGuQ8kuc9pgc9s8qqaq=dirpe0xb9q8qiLsFr0=vr0=vr0dc8meaabaqaciaacaGaaeqabaqabeGadaaakeaacuWG6bGEgaWcamaaBaaaleaacqWGRbWAaeqaaaaa@2FC6@ is quite simple, since the layer based reduction method makes clear statements about the states of the reduced model (see step by step procedure). The state vector of the reduced model z→k
 MathType@MTEF@5@5@+=feaafiart1ev1aaatCvAUfKttLearuWrP9MDH5MBPbIqV92AaeXatLxBI9gBaebbnrfifHhDYfgasaacH8akY=wiFfYdH8Gipec8Eeeu0xXdbba9frFj0=OqFfea0dXdd9vqai=hGuQ8kuc9pgc9s8qqaq=dirpe0xb9q8qiLsFr0=vr0=vr0dc8meaabaqaciaacaGaaeqabaqabeGadaaakeaacuWG6bGEgaWcamaaBaaaleaacqWGRbWAaeqaaaaa@2FC6@ comprises all states of the reduced model that are defined by an ODE.

If we consider the example shown in Figure 5, the vector z→k
 MathType@MTEF@5@5@+=feaafiart1ev1aaatCvAUfKttLearuWrP9MDH5MBPbIqV92AaeXatLxBI9gBaebbnrfifHhDYfgasaacH8akY=wiFfYdH8Gipec8Eeeu0xXdbba9frFj0=OqFfea0dXdd9vqai=hGuQ8kuc9pgc9s8qqaq=dirpe0xb9q8qiLsFr0=vr0=vr0dc8meaabaqaciaacaGaaeqabaqabeGadaaakeaacuWG6bGEgaWcamaaBaaaleaacqWGRbWAaeqaaaaa@2FC6@ includes the states *RLO, ROP, RLP *and *RXE *(Equation 26). Importantly, this definition of z→k
 MathType@MTEF@5@5@+=feaafiart1ev1aaatCvAUfKttLearuWrP9MDH5MBPbIqV92AaeXatLxBI9gBaebbnrfifHhDYfgasaacH8akY=wiFfYdH8Gipec8Eeeu0xXdbba9frFj0=OqFfea0dXdd9vqai=hGuQ8kuc9pgc9s8qqaq=dirpe0xb9q8qiLsFr0=vr0=vr0dc8meaabaqaciaacaGaaeqabaqabeGadaaakeaacuWG6bGEgaWcamaaBaaaleaacqWGRbWAaeqaaaaa@2FC6@ suggests a linear transformation. The states z→n
 MathType@MTEF@5@5@+=feaafiart1ev1aaatCvAUfKttLearuWrP9MDH5MBPbIqV92AaeXatLxBI9gBaebbnrfifHhDYfgasaacH8akY=wiFfYdH8Gipec8Eeeu0xXdbba9frFj0=OqFfea0dXdd9vqai=hGuQ8kuc9pgc9s8qqaq=dirpe0xb9q8qiLsFr0=vr0=vr0dc8meaabaqaciaacaGaaeqabaqabeGadaaakeaacuWG6bGEgaWcamaaBaaaleaacqWGUbGBaeqaaaaa@2FCC@ (in the example system: one state *z*_*n*_) have to be chosen such that the resulting transformation z→=φ→(x→)
 MathType@MTEF@5@5@+=feaafiart1ev1aaatCvAUfKttLearuWrP9MDH5MBPbIqV92AaeXatLxBI9gBaebbnrfifHhDYfgasaacH8akY=wiFfYdH8Gipec8Eeeu0xXdbba9frFj0=OqFfea0dXdd9vqai=hGuQ8kuc9pgc9s8qqaq=dirpe0xb9q8qiLsFr0=vr0=vr0dc8meaabaqaciaacaGaaeqabaqabeGadaaakeaacuWG6bGEgaWcaiabg2da9GGaciqb=z8aMzaalaGaeiikaGIafmiEaGNbaSaacqGGPaqkaaa@3450@ is invertible. Theoretically, there exist an infinite number of possibilities to choose z→n
 MathType@MTEF@5@5@+=feaafiart1ev1aaatCvAUfKttLearuWrP9MDH5MBPbIqV92AaeXatLxBI9gBaebbnrfifHhDYfgasaacH8akY=wiFfYdH8Gipec8Eeeu0xXdbba9frFj0=OqFfea0dXdd9vqai=hGuQ8kuc9pgc9s8qqaq=dirpe0xb9q8qiLsFr0=vr0=vr0dc8meaabaqaciaacaGaaeqabaqabeGadaaakeaacuWG6bGEgaWcamaaBaaaleaacqWGUbGBaeqaaaaa@2FCC@. The layer based reduction method does not make any direct statement about how to choose z→n
 MathType@MTEF@5@5@+=feaafiart1ev1aaatCvAUfKttLearuWrP9MDH5MBPbIqV92AaeXatLxBI9gBaebbnrfifHhDYfgasaacH8akY=wiFfYdH8Gipec8Eeeu0xXdbba9frFj0=OqFfea0dXdd9vqai=hGuQ8kuc9pgc9s8qqaq=dirpe0xb9q8qiLsFr0=vr0=vr0dc8meaabaqaciaacaGaaeqabaqabeGadaaakeaacuWG6bGEgaWcamaaBaaaleaacqWGUbGBaeqaaaaa@2FCC@. The reason is that the additional states z→n
 MathType@MTEF@5@5@+=feaafiart1ev1aaatCvAUfKttLearuWrP9MDH5MBPbIqV92AaeXatLxBI9gBaebbnrfifHhDYfgasaacH8akY=wiFfYdH8Gipec8Eeeu0xXdbba9frFj0=OqFfea0dXdd9vqai=hGuQ8kuc9pgc9s8qqaq=dirpe0xb9q8qiLsFr0=vr0=vr0dc8meaabaqaciaacaGaaeqabaqabeGadaaakeaacuWG6bGEgaWcamaaBaaaleaacqWGUbGBaeqaaaaa@2FCC@ can be chosen freely, as long as the transformation is invertible. Their choice does not affect the shape of the reduced model equations. This can be proven by some simple considerations. Let us assume that we have chosen a certain z→n
 MathType@MTEF@5@5@+=feaafiart1ev1aaatCvAUfKttLearuWrP9MDH5MBPbIqV92AaeXatLxBI9gBaebbnrfifHhDYfgasaacH8akY=wiFfYdH8Gipec8Eeeu0xXdbba9frFj0=OqFfea0dXdd9vqai=hGuQ8kuc9pgc9s8qqaq=dirpe0xb9q8qiLsFr0=vr0=vr0dc8meaabaqaciaacaGaaeqabaqabeGadaaakeaacuWG6bGEgaWcamaaBaaaleaacqWGUbGBaeqaaaaa@2FCC@ and have a model like shown in Equation 32. Additionally, we also have some algebraic equations for the approximation step z→n−ψ→(z→k)
 MathType@MTEF@5@5@+=feaafiart1ev1aaatCvAUfKttLearuWrP9MDH5MBPbIqV92AaeXatLxBI9gBaebbnrfifHhDYfgasaacH8akY=wiFfYdH8Gipec8Eeeu0xXdbba9frFj0=OqFfea0dXdd9vqai=hGuQ8kuc9pgc9s8qqaq=dirpe0xb9q8qiLsFr0=vr0=vr0dc8meaabaqaciaacaGaaeqabaqabeGadaaakeaacuWG6bGEgaWcamaaBaaaleaacqWGUbGBaeqaaOGaeyOeI0ccciGaf8hYdKNbaSaacqGGOaakcuWG6bGEgaWcamaaBaaaleaacqWGRbWAaeqaaOGaeiykaKcaaa@3780@ and consecutively reduced model equations z→˙k=g→1(z→k,ψ→(z→k))
 MathType@MTEF@5@5@+=feaafiart1ev1aaatCvAUfKttLearuWrP9MDH5MBPbIqV92AaeXatLxBI9gBaebbnrfifHhDYfgasaacH8akY=wiFfYdH8Gipec8Eeeu0xXdbba9frFj0=OqFfea0dXdd9vqai=hGuQ8kuc9pgc9s8qqaq=dirpe0xb9q8qiLsFr0=vr0=vr0dc8meaabaqaciaacaGaaeqabaqabeGadaaakeaacuWG6bGEgaWcgaGaamaaBaaaleaacqWGRbWAaeqaaOGaeyypa0Jafm4zaCMbaSaadaWgaaWcbaGaeGymaedabeaakiabcIcaOiqbdQha6zaalaWaaSbaaSqaaiabdUgaRbqabaGccqGGSaaliiGacuWFipqEgaWcaiabcIcaOiqbdQha6zaalaWaaSbaaSqaaiabdUgaRbqabaGccqGGPaqkcqGGPaqkaaa@3FE0@. Now the question is whether the reduced model still has the same structure if we choose another representation of z→n
 MathType@MTEF@5@5@+=feaafiart1ev1aaatCvAUfKttLearuWrP9MDH5MBPbIqV92AaeXatLxBI9gBaebbnrfifHhDYfgasaacH8akY=wiFfYdH8Gipec8Eeeu0xXdbba9frFj0=OqFfea0dXdd9vqai=hGuQ8kuc9pgc9s8qqaq=dirpe0xb9q8qiLsFr0=vr0=vr0dc8meaabaqaciaacaGaaeqabaqabeGadaaakeaacuWG6bGEgaWcamaaBaaaleaacqWGUbGBaeqaaaaa@2FCC@, namely z→n
 MathType@MTEF@5@5@+=feaafiart1ev1aaatCvAUfKttLearuWrP9MDH5MBPbIqV92AaeXatLxBI9gBaebbnrfifHhDYfgasaacH8akY=wiFfYdH8Gipec8Eeeu0xXdbba9frFj0=OqFfea0dXdd9vqai=hGuQ8kuc9pgc9s8qqaq=dirpe0xb9q8qiLsFr0=vr0=vr0dc8meaabaqaciaacaGaaeqabaqabeGadaaakeaacuWG6bGEgaWcamaaBaaaleaacqWGUbGBaeqaaaaa@2FCC@. This can be realized by using another linear transformation

[z˜→kz˜→n]=T⋅[z→kz→n]whereT=[I0T1T2]andT−1=[I0−T2−1T1T2−1],
 MathType@MTEF@5@5@+=feaafiart1ev1aaatCvAUfKttLearuWrP9MDH5MBPbIqV92AaeXatLxBI9gBaebbnrfifHhDYfgasaacH8akY=wiFfYdH8Gipec8Eeeu0xXdbba9frFj0=OqFfea0dXdd9vqai=hGuQ8kuc9pgc9s8qqaq=dirpe0xb9q8qiLsFr0=vr0=vr0dc8meaabaqaciaacaGaaeqabaqabeGadaaakeaafaqabeGabaaabaqbaeqabeWaaaqaamaadmaabaqbaeqabiqaaaqaaiqbdQha6zaaiyaalaWaaSbaaSqaaiabdUgaRbqabaaakeaacuWG6bGEgaacgaWcamaaBaaaleaacqWGUbGBaeqaaaaaaOGaay5waiaaw2faaiabg2da9iabdsfaujabgwSixpaadmaabaqbaeqabiqaaaqaaiqbdQha6zaalaWaaSbaaSqaaiabdUgaRbqabaaakeaacuWG6bGEgaWcamaaBaaaleaacqWGUbGBaeqaaaaaaOGaay5waiaaw2faaaqaaiabbEha3jabbIgaOjabbwgaLjabbkhaYjabbwgaLbqaaiabdsfaujabg2da9maadmaabaqbaeqabiGaaaqaaiabdMeajbqaaiabicdaWaqaaiabdsfaunaaBaaaleaacqaIXaqmaeqaaaGcbaGaemivaq1aaSbaaSqaaiabikdaYaqabaaaaaGccaGLBbGaayzxaaaaaaqaauaabeqabiaaaeaacqqGHbqycqqGUbGBcqqGKbazaeaacqWGubavdaahaaWcbeqaaiabgkHiTiabigdaXaaakiabg2da9maadmaabaqbaeqabiGaaaqaaiabdMeajbqaaiabicdaWaqaaiabgkHiTiabdsfaunaaDaaaleaacqaIYaGmaeaacqGHsislcqaIXaqmaaGccqWGubavdaWgaaWcbaGaeGymaedabeaaaOqaaiabdsfaunaaDaaaleaacqaIYaGmaeaacqGHsislcqaIXaqmaaaaaaGccaGLBbGaayzxaaGaeiilaWcaaaaaaaa@6CBA@

which transforms the states z→n
 MathType@MTEF@5@5@+=feaafiart1ev1aaatCvAUfKttLearuWrP9MDH5MBPbIqV92AaeXatLxBI9gBaebbnrfifHhDYfgasaacH8akY=wiFfYdH8Gipec8Eeeu0xXdbba9frFj0=OqFfea0dXdd9vqai=hGuQ8kuc9pgc9s8qqaq=dirpe0xb9q8qiLsFr0=vr0=vr0dc8meaabaqaciaacaGaaeqabaqabeGadaaakeaacuWG6bGEgaWcamaaBaaaleaacqWGUbGBaeqaaaaa@2FCC@ of Equation 32 to new states z→n
 MathType@MTEF@5@5@+=feaafiart1ev1aaatCvAUfKttLearuWrP9MDH5MBPbIqV92AaeXatLxBI9gBaebbnrfifHhDYfgasaacH8akY=wiFfYdH8Gipec8Eeeu0xXdbba9frFj0=OqFfea0dXdd9vqai=hGuQ8kuc9pgc9s8qqaq=dirpe0xb9q8qiLsFr0=vr0=vr0dc8meaabaqaciaacaGaaeqabaqabeGadaaakeaacuWG6bGEgaWcamaaBaaaleaacqWGUbGBaeqaaaaa@2FCC@. Since *I *is the identity matrix, z˜→k=z→k
 MathType@MTEF@5@5@+=feaafiart1ev1aaatCvAUfKttLearuWrP9MDH5MBPbIqV92AaeXatLxBI9gBaebbnrfifHhDYfgasaacH8akY=wiFfYdH8Gipec8Eeeu0xXdbba9frFj0=OqFfea0dXdd9vqai=hGuQ8kuc9pgc9s8qqaq=dirpe0xb9q8qiLsFr0=vr0=vr0dc8meaabaqaciaacaGaaeqabaqabeGadaaakeaacuWG6bGEgaacgaWcamaaBaaaleaacqWGRbWAaeqaaOGaeyypa0JafmOEaONbaSaadaWgaaWcbaGaem4AaSgabeaaaaa@33FE@.

In the transformed system the structure of the ODEs for z→k
 MathType@MTEF@5@5@+=feaafiart1ev1aaatCvAUfKttLearuWrP9MDH5MBPbIqV92AaeXatLxBI9gBaebbnrfifHhDYfgasaacH8akY=wiFfYdH8Gipec8Eeeu0xXdbba9frFj0=OqFfea0dXdd9vqai=hGuQ8kuc9pgc9s8qqaq=dirpe0xb9q8qiLsFr0=vr0=vr0dc8meaabaqaciaacaGaaeqabaqabeGadaaakeaacuWG6bGEgaWcamaaBaaaleaacqWGRbWAaeqaaaaa@2FC6@ has changed:

[z˜→˙kz˜→˙n]=[g→1(z˜→k,−T2−1T1z˜→k+T2−1z˜→n,u→)g→2(z˜→k,−T2−1T1z˜→k+T2−1z˜→n,u→)].
 MathType@MTEF@5@5@+=feaafiart1ev1aaatCvAUfKttLearuWrP9MDH5MBPbIqV92AaeXatLxBI9gBaebbnrfifHhDYfgasaacH8akY=wiFfYdH8Gipec8Eeeu0xXdbba9frFj0=OqFfea0dXdd9vqai=hGuQ8kuc9pgc9s8qqaq=dirpe0xb9q8qiLsFr0=vr0=vr0dc8meaabaqaciaacaGaaeqabaqabeGadaaakeaadaWadaqaauaabeqaceaaaeaacuWG6bGEgaacgaWcgaGaamaaBaaaleaacqWGRbWAaeqaaaGcbaGafmOEaONbaGGbaSGbaiaadaWgaaWcbaGaemOBa4gabeaaaaaakiaawUfacaGLDbaacqGH9aqpdaWadaqaauaabeqaceaaaeaacuWGNbWzgaWcamaaBaaaleaacqaIXaqmaeqaaOGaeiikaGIafmOEaONbaGGbaSaadaWgaaWcbaGaem4AaSgabeaakiabcYcaSiabgkHiTiabdsfaunaaDaaaleaacqaIYaGmaeaacqGHsislcqaIXaqmaaGccqWGubavdaWgaaWcbaGaeGymaedabeaakiqbdQha6zaaiyaalaWaaSbaaSqaaiabdUgaRbqabaGccqGHRaWkcqWGubavdaqhaaWcbaGaeGOmaidabaGaeyOeI0IaeGymaedaaOGafmOEaONbaGGbaSaadaWgaaWcbaGaemOBa4gabeaakiabcYcaSiqbdwha1zaalaGaeiykaKcabaGafm4zaCMbaSaadaWgaaWcbaGaeGOmaidabeaakiabcIcaOiqbdQha6zaaiyaalaWaaSbaaSqaaiabdUgaRbqabaGccqGGSaalcqGHsislcqWGubavdaqhaaWcbaGaeGOmaidabaGaeyOeI0IaeGymaedaaOGaemivaq1aaSbaaSqaaiabigdaXaqabaGccuWG6bGEgaacgaWcamaaBaaaleaacqWGRbWAaeqaaOGaey4kaSIaemivaq1aa0baaSqaaiabikdaYaqaaiabgkHiTiabigdaXaaakiqbdQha6zaaiyaalaWaaSbaaSqaaiabd6gaUbqabaGccqGGSaalcuWG1bqDgaWcaiabcMcaPaaaaiaawUfacaGLDbaacqGGUaGlaaa@747C@

However, the transformed algebraic equations now also have a different form:

z˜→n=T1z˜→k+T2ψ→(z˜→k).
 MathType@MTEF@5@5@+=feaafiart1ev1aaatCvAUfKttLearuWrP9MDH5MBPbIqV92AaeXatLxBI9gBaebbnrfifHhDYfgasaacH8akY=wiFfYdH8Gipec8Eeeu0xXdbba9frFj0=OqFfea0dXdd9vqai=hGuQ8kuc9pgc9s8qqaq=dirpe0xb9q8qiLsFr0=vr0=vr0dc8meaabaqaciaacaGaaeqabaqabeGadaaakeaacuWG6bGEgaacgaWcamaaBaaaleaacqWGUbGBaeqaaOGaeyypa0Jaemivaq1aaSbaaSqaaiabigdaXaqabaGccuWG6bGEgaacgaWcamaaBaaaleaacqWGRbWAaeqaaOGaey4kaSIaemivaq1aaSbaaSqaaiabikdaYaqabaacciGccuWFipqEgaWcaiabcIcaOiqbdQha6zaaiyaalaWaaSbaaSqaaiabdUgaRbqabaGccqGGPaqkcqGGUaGlaaa@415D@

If one now replaces z˜→n
 MathType@MTEF@5@5@+=feaafiart1ev1aaatCvAUfKttLearuWrP9MDH5MBPbIqV92AaeXatLxBI9gBaebbnrfifHhDYfgasaacH8akY=wiFfYdH8Gipec8Eeeu0xXdbba9frFj0=OqFfea0dXdd9vqai=hGuQ8kuc9pgc9s8qqaq=dirpe0xb9q8qiLsFr0=vr0=vr0dc8meaabaqaciaacaGaaeqabaqabeGadaaakeaacuWG6bGEgaacgaWcamaaBaaaleaacqWGUbGBaeqaaaaa@2FDA@ in Equation 35 by what is given in Equation 36 and observes z˜→k=z→k
 MathType@MTEF@5@5@+=feaafiart1ev1aaatCvAUfKttLearuWrP9MDH5MBPbIqV92AaeXatLxBI9gBaebbnrfifHhDYfgasaacH8akY=wiFfYdH8Gipec8Eeeu0xXdbba9frFj0=OqFfea0dXdd9vqai=hGuQ8kuc9pgc9s8qqaq=dirpe0xb9q8qiLsFr0=vr0=vr0dc8meaabaqaciaacaGaaeqabaqabeGadaaakeaacuWG6bGEgaacgaWcamaaBaaaleaacqWGRbWAaeqaaOGaeyypa0JafmOEaONbaSaadaWgaaWcbaGaem4AaSgabeaaaaa@33FE@ the resulting reduced model again is z→˙k=g→1(z→k,ψ→(z→k))
 MathType@MTEF@5@5@+=feaafiart1ev1aaatCvAUfKttLearuWrP9MDH5MBPbIqV92AaeXatLxBI9gBaebbnrfifHhDYfgasaacH8akY=wiFfYdH8Gipec8Eeeu0xXdbba9frFj0=OqFfea0dXdd9vqai=hGuQ8kuc9pgc9s8qqaq=dirpe0xb9q8qiLsFr0=vr0=vr0dc8meaabaqaciaacaGaaeqabaqabeGadaaakeaacuWG6bGEgaWcgaGaamaaBaaaleaacqWGRbWAaeqaaOGaeyypa0Jafm4zaCMbaSaadaWgaaWcbaGaeGymaedabeaakiabcIcaOiqbdQha6zaalaWaaSbaaSqaaiabdUgaRbqabaGccqGGSaaliiGacuWFipqEgaWcaiabcIcaOiqbdQha6zaalaWaaSbaaSqaaiabdUgaRbqabaGccqGGPaqkcqGGPaqkaaa@3FE0@. This means that the reduced model structure is completely determined by defining the states z→k
 MathType@MTEF@5@5@+=feaafiart1ev1aaatCvAUfKttLearuWrP9MDH5MBPbIqV92AaeXatLxBI9gBaebbnrfifHhDYfgasaacH8akY=wiFfYdH8Gipec8Eeeu0xXdbba9frFj0=OqFfea0dXdd9vqai=hGuQ8kuc9pgc9s8qqaq=dirpe0xb9q8qiLsFr0=vr0=vr0dc8meaabaqaciaacaGaaeqabaqabeGadaaakeaacuWG6bGEgaWcamaaBaaaleaacqWGRbWAaeqaaaaa@2FC6@ plus the definition of *dim*(z→n
 MathType@MTEF@5@5@+=feaafiart1ev1aaatCvAUfKttLearuWrP9MDH5MBPbIqV92AaeXatLxBI9gBaebbnrfifHhDYfgasaacH8akY=wiFfYdH8Gipec8Eeeu0xXdbba9frFj0=OqFfea0dXdd9vqai=hGuQ8kuc9pgc9s8qqaq=dirpe0xb9q8qiLsFr0=vr0=vr0dc8meaabaqaciaacaGaaeqabaqabeGadaaakeaacuWG6bGEgaWcamaaBaaaleaacqWGUbGBaeqaaaaa@2FCC@) algebraic equations in order to approximate the neglected states. These equations are the ratio equations like Equation 27, transformed to z→
 MathType@MTEF@5@5@+=feaafiart1ev1aaatCvAUfKttLearuWrP9MDH5MBPbIqV92AaeXatLxBI9gBaebbnrfifHhDYfgasaacH8akY=wiFfYdH8Gipec8Eeeu0xXdbba9frFj0=OqFfea0dXdd9vqai=hGuQ8kuc9pgc9s8qqaq=dirpe0xb9q8qiLsFr0=vr0=vr0dc8meaabaqaciaacaGaaeqabaqabeGadaaakeaacuWG6bGEgaWcaaaa@2E3B@-coordinates. They are necessary to reconstruct the states of the detailed model. Alternatively, for building the reduced model directly it is not necessary to postulate these equations explicitly. They are already contained implicitly in the reduced model equations (confer Equation 25).

#### Approximation of neglected states

After having defined the new coordinates z→k
 MathType@MTEF@5@5@+=feaafiart1ev1aaatCvAUfKttLearuWrP9MDH5MBPbIqV92AaeXatLxBI9gBaebbnrfifHhDYfgasaacH8akY=wiFfYdH8Gipec8Eeeu0xXdbba9frFj0=OqFfea0dXdd9vqai=hGuQ8kuc9pgc9s8qqaq=dirpe0xb9q8qiLsFr0=vr0=vr0dc8meaabaqaciaacaGaaeqabaqabeGadaaakeaacuWG6bGEgaWcamaaBaaaleaacqWGRbWAaeqaaaaa@2FC6@ and z→n
 MathType@MTEF@5@5@+=feaafiart1ev1aaatCvAUfKttLearuWrP9MDH5MBPbIqV92AaeXatLxBI9gBaebbnrfifHhDYfgasaacH8akY=wiFfYdH8Gipec8Eeeu0xXdbba9frFj0=OqFfea0dXdd9vqai=hGuQ8kuc9pgc9s8qqaq=dirpe0xb9q8qiLsFr0=vr0=vr0dc8meaabaqaciaacaGaaeqabaqabeGadaaakeaacuWG6bGEgaWcamaaBaaaleaacqWGUbGBaeqaaaaa@2FCC@ of the detailed system, one has to find algebraic equations Ψ→(x→)=0
 MathType@MTEF@5@5@+=feaafiart1ev1aaatCvAUfKttLearuWrP9MDH5MBPbIqV92AaeXatLxBI9gBaebbnrfifHhDYfgasaacH8akY=wiFfYdH8Gipec8Eeeu0xXdbba9frFj0=OqFfea0dXdd9vqai=hGuQ8kuc9pgc9s8qqaq=dirpe0xb9q8qiLsFr0=vr0=vr0dc8meaabaqaciaacaGaaeqabaqabeGadaaakeaacuqHOoqwgaWcaiabcIcaOiqbdIha4zaalaGaeiykaKIaeyypa0JaeGimaadaaa@337E@, which have to be transformed to z→
 MathType@MTEF@5@5@+=feaafiart1ev1aaatCvAUfKttLearuWrP9MDH5MBPbIqV92AaeXatLxBI9gBaebbnrfifHhDYfgasaacH8akY=wiFfYdH8Gipec8Eeeu0xXdbba9frFj0=OqFfea0dXdd9vqai=hGuQ8kuc9pgc9s8qqaq=dirpe0xb9q8qiLsFr0=vr0=vr0dc8meaabaqaciaacaGaaeqabaqabeGadaaakeaacuWG6bGEgaWcaaaa@2E3B@ coordinates in order to approximate the states z→n
 MathType@MTEF@5@5@+=feaafiart1ev1aaatCvAUfKttLearuWrP9MDH5MBPbIqV92AaeXatLxBI9gBaebbnrfifHhDYfgasaacH8akY=wiFfYdH8Gipec8Eeeu0xXdbba9frFj0=OqFfea0dXdd9vqai=hGuQ8kuc9pgc9s8qqaq=dirpe0xb9q8qiLsFr0=vr0=vr0dc8meaabaqaciaacaGaaeqabaqabeGadaaakeaacuWG6bGEgaWcamaaBaaaleaacqWGUbGBaeqaaaaa@2FCC@. Our considerations shall start with the work of Borisov et al. [22,46], who discuss a special form of algebraic constraint equations in combinatorial networks. We shortly review these ideas, extend them and show that these considerations help us to evaluate and quantify the layer based method. Borisov et al. discussed reduced modeling of a scaffold protein with a large number of independent binding sites. If binding of a ligand to the scaffold changes neither binding affinity nor velocity of all the other binding sites, they will be independent. Binding events at each binding site then can be described separately in a strongly reduced model that only provides information about the levels of occupancy of the different domains. They suggested that information about detailed complex composition can be reconstructed using the calculus of probability. This can be exemplified considering a scaffold with two independent binding sites. Such a molecule can occur in four different states, namely completely unoccupied *D*[0, 0], with only the first or the second domain being occupied *D*[1, 0] and *D*[0, 1], or with both sites being occupied *D *[1, 1]. Due to independence of binding sites the calculus of probability suggests that the concentration of scaffold with both domains being occupied can be calculated as

D11=R1X⋅RX1RXX.
 MathType@MTEF@5@5@+=feaafiart1ev1aaatCvAUfKttLearuWrP9MDH5MBPbIqV92AaeXatLxBI9gBaebbnrfifHhDYfgasaacH8akY=wiFfYdH8Gipec8Eeeu0xXdbba9frFj0=OqFfea0dXdd9vqai=hGuQ8kuc9pgc9s8qqaq=dirpe0xb9q8qiLsFr0=vr0=vr0dc8meaabaqaciaacaGaaeqabaqabeGadaaakeaacqWGebarcqaIXaqmcqaIXaqmcqGH9aqpdaWcaaqaaiabdkfasjabigdaXiabdIfayjabgwSixlabdkfasjabdIfayjabigdaXaqaaiabdkfasjabdIfayjabdIfaybaacqGGUaGlaaa@3E2C@

Borisov et al. showed that if this equation is fulfilled at a point of time *t*_0 _it will be fulfilled for all times *t *> *t*_0_. This equation can be simplified by elementary transformations to

*DOO*·*D*11 - *D*1*O*·*DO*1 = 0,

which is equivalent to the ratio Equation 27.

In both the examples here and in Figure 3 the four species occurring in the ratio equations form a reaction cycle. These cycles consist of two processes in different layers, which do not influence each other directly, e.g. ligand and effector binding. It is quite obvious that each reaction network that is decomposed into layers includes such independent cycles. All pairs of processes being located in distinct layers do not interact *directly*. Indirect interactions with at least one all-or-none interaction in between are possible. For each of these cycles one can formulate a ratio equation like that described above. Note that each ratio equation lowers the number of states of the reduced model by one.

The assumption of rapid equilibrium [47] for each pair of reactions describing the same process also leads to the same ratio equations if the equilibrium constant is eliminated from the equations. From this it follows that these ratio equations are at least approximately fulfilled if the rapid equilibrium assumption is a good approximation. Therefore, the approximation error vanishes completely if the system reaches thermodynamic equilibrium [47], as illustrated below. The ratio equations are used to approximate the states z→n
 MathType@MTEF@5@5@+=feaafiart1ev1aaatCvAUfKttLearuWrP9MDH5MBPbIqV92AaeXatLxBI9gBaebbnrfifHhDYfgasaacH8akY=wiFfYdH8Gipec8Eeeu0xXdbba9frFj0=OqFfea0dXdd9vqai=hGuQ8kuc9pgc9s8qqaq=dirpe0xb9q8qiLsFr0=vr0=vr0dc8meaabaqaciaacaGaaeqabaqabeGadaaakeaacuWG6bGEgaWcamaaBaaaleaacqWGUbGBaeqaaaaa@2FCC@ as discussed above.

#### Approximation quality

**Figure 7:**
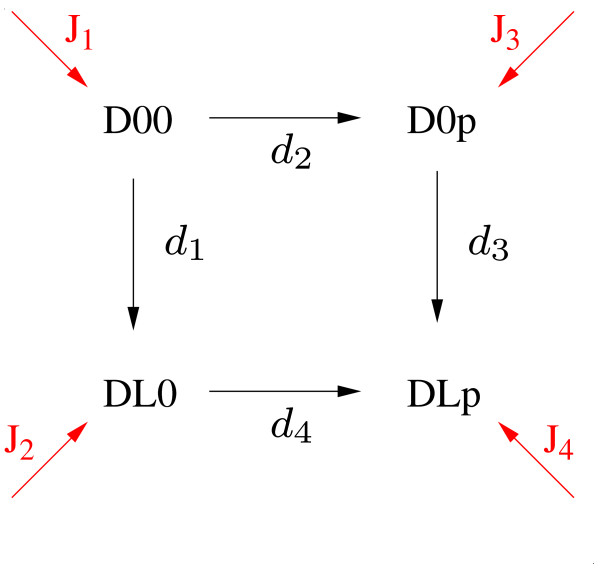
**Typical reaction cycle with input fluxes**. The in-fluxes *J*_*i *_result from indirect interactions between processes of different layers. As the processes are assumed to be independent, the rates *d*_1 _and *d*_3 _are parametrized by *k*_1 _and *k*_-1_. Additionally, the rates *d*_2 _and *d*_4 _are parametrized by *k*_2 _and *k*_-2_.

In order to mathematically analyze approximation of these ratio equations, we consider a reaction cycle with four different influxes *J*_*i *_(see Figure 7). Each reaction cycle of processes of different layers can be formulated in such a way. According to Borisov et al. [22] the proposed equations provide an exact approximation if the processes forming the cycle interact neither directly nor indirectly and if the initial conditions already fulfill the equation. We already discussed that each pair of processes located in different layers and forming a reaction cycle does not interact directly. However, the occurring all-or-none interactions between the layers may realize indirect interactions between the considered processes. Indirect interactions always result in certain external influxes *J*_*i *_as shown in Figure 7 and the relations like Equation 27 become erroneous. In this case we can define an error function *g *as

*g *= *DOO*·*DLp *- *DOp*·*DLO*.

Interestingly, this error function fulfills the linear differential equation

g˙+a g=u(t)
 MathType@MTEF@5@5@+=feaafiart1ev1aaatCvAUfKttLearuWrP9MDH5MBPbIqV92AaeXatLxBI9gBaebbnrfifHhDYfgasaacH8akY=wiFfYdH8Gipec8Eeeu0xXdbba9frFj0=OqFfea0dXdd9vqai=hGuQ8kuc9pgc9s8qqaq=dirpe0xb9q8qiLsFr0=vr0=vr0dc8meaabaqaciaacaGaaeqabaqabeGadaaakeaacuWGNbWzgaGaaiabgUcaRiabdggaHjabbccaGiabdEgaNjabg2da9iabdwha1jabcIcaOiabdsha0jabcMcaPaaa@37F3@

with

*a *= *k*_1 _+ *k*_-1 _+ *k*_2 _+ *k*_-2_

and

*u*(*t*) = *J*_1_·*DLp *- *J*_2_·*DOp *- *J*_3_·*DLO *+ *J*_4_·*DOO*

where the rates *d*_1 _and *d*_3 _are parametrized by *k*_1 _and *k*_-1 _and the rates *d*_2 _and *d*_4 _are parametrized by *k*_2 _and *k*_-2_.

It is apparent that the error will completely vanish if *u*(*t*) = 0, e.g. if the in-fluxes vanish. From a thermodynamic point of view all reaction rates have to vanish if the system reaches thermodynamic equilibrium. Thus, one can guarantee that the stationary error always is zero if the considered system ends up in thermodynamic equilibrium. This is the case if two major conditions are fulfilled. These are that the Wegscheider condition (detailed balance) [47] is satisfied in the whole network and none of the modeled concentrations is assumed to be clamped. However, most biological reaction networks operate far from thermodynamic equilibrium. Hence, it is important to discuss the error for these cases. From Equation 40 one can easily derive the stationary error as

gs=usa
 MathType@MTEF@5@5@+=feaafiart1ev1aaatCvAUfKttLearuWrP9MDH5MBPbIqV92AaeXatLxBI9gBaebbnrfifHhDYfgasaacH8akY=wiFfYdH8Gipec8Eeeu0xXdbba9frFj0=OqFfea0dXdd9vqai=hGuQ8kuc9pgc9s8qqaq=dirpe0xb9q8qiLsFr0=vr0=vr0dc8meaabaqaciaacaGaaeqabaqabeGadaaakeaacqWGNbWzdaWgaaWcbaGaem4Camhabeaakiabg2da9maalaaabaGaemyDau3aaSbaaSqaaiabdohaZbqabaaakeaacqWGHbqyaaaaaa@3521@

and the dynamic error as

g(t)=e−a t(g0+∫0tea τu(τ)dτ).
 MathType@MTEF@5@5@+=feaafiart1ev1aaatCvAUfKttLearuWrP9MDH5MBPbIqV92AaeXatLxBI9gBaebbnrfifHhDYfgasaacH8akY=wiFfYdH8Gipec8Eeeu0xXdbba9frFj0=OqFfea0dXdd9vqai=hGuQ8kuc9pgc9s8qqaq=dirpe0xb9q8qiLsFr0=vr0=vr0dc8meaabaqaciaacaGaaeqabaqabeGadaaakeaacqWGNbWzcqGGOaakcqWG0baDcqGGPaqkcqGH9aqpcqWGLbqzdaahaaWcbeqaaiabgkHiTiabdggaHjabbccaGiabdsha0baakmaabmaabaGaem4zaC2aaSbaaSqaaiabicdaWaqabaGccqGHRaWkdaWdXaqaaiabdwgaLnaaCaaaleqabaGaemyyaeMaeeiiaaccciGae8hXdqhaaOGaemyDauNaeiikaGIae8hXdqNaeiykaKIaemizaqMae8hXdqhaleaacqaIWaamaeaacqWG0baDa0Gaey4kIipaaOGaayjkaiaawMcaaiabc6caUaaa@4FE0@

In order to provide at least a rough estimation of the maximal error we assume that *u*(*t*) = *u*_*max *_= max (*u*(*t*)). With this we can give the following error bound

g(t)≤g0e−a t+umaxa(1−e−a t).
 MathType@MTEF@5@5@+=feaafiart1ev1aaatCvAUfKttLearuWrP9MDH5MBPbIqV92AaeXatLxBI9gBaebbnrfifHhDYfgasaacH8akY=wiFfYdH8Gipec8Eeeu0xXdbba9frFj0=OqFfea0dXdd9vqai=hGuQ8kuc9pgc9s8qqaq=dirpe0xb9q8qiLsFr0=vr0=vr0dc8meaabaqaciaacaGaaeqabaqabeGadaaakeaacqWGNbWzcqGGOaakcqWG0baDcqGGPaqkcqGHKjYOcqWGNbWzdaWgaaWcbaGaeGimaadabeaakiabdwgaLnaaCaaaleqabaGaeyOeI0IaemyyaeMaeeiiaaIaemiDaqhaaOGaey4kaSYaaSaaaeaacqWG1bqDdaWgaaWcbaGaemyBa0MaemyyaeMaemiEaGhabeaaaOqaaiabdggaHbaadaqadaqaaiabigdaXiabgkHiTiabdwgaLnaaCaaaleqabaGaeyOeI0IaemyyaeMaeeiiaaIaemiDaqhaaaGccaGLOaGaayzkaaGaeiOla4caaa@4DA1@

These equations show that both the steady state error as well as the maximal dynamic error decrease for increasing values of *a*, which corresponds to increasing values of the kinetic parameters *k*_1_, *k*_-1_, *k*_2 _and *k*_2_, and is zero for one of these values going to infinity. Note, that even for a large error *g *the reduced dynamic model equations may provide very good approximations for the states z→k
 MathType@MTEF@5@5@+=feaafiart1ev1aaatCvAUfKttLearuWrP9MDH5MBPbIqV92AaeXatLxBI9gBaebbnrfifHhDYfgasaacH8akY=wiFfYdH8Gipec8Eeeu0xXdbba9frFj0=OqFfea0dXdd9vqai=hGuQ8kuc9pgc9s8qqaq=dirpe0xb9q8qiLsFr0=vr0=vr0dc8meaabaqaciaacaGaaeqabaqabeGadaaakeaacuWG6bGEgaWcamaaBaaaleaacqWGRbWAaeqaaaaa@2FC6@. This is the case if the erroneous states z→n
 MathType@MTEF@5@5@+=feaafiart1ev1aaatCvAUfKttLearuWrP9MDH5MBPbIqV92AaeXatLxBI9gBaebbnrfifHhDYfgasaacH8akY=wiFfYdH8Gipec8Eeeu0xXdbba9frFj0=OqFfea0dXdd9vqai=hGuQ8kuc9pgc9s8qqaq=dirpe0xb9q8qiLsFr0=vr0=vr0dc8meaabaqaciaacaGaaeqabaqabeGadaaakeaacuWG6bGEgaWcamaaBaaaleaacqWGUbGBaeqaaaaa@2FCC@ that are approximated only have very little influence on z→k
 MathType@MTEF@5@5@+=feaafiart1ev1aaatCvAUfKttLearuWrP9MDH5MBPbIqV92AaeXatLxBI9gBaebbnrfifHhDYfgasaacH8akY=wiFfYdH8Gipec8Eeeu0xXdbba9frFj0=OqFfea0dXdd9vqai=hGuQ8kuc9pgc9s8qqaq=dirpe0xb9q8qiLsFr0=vr0=vr0dc8meaabaqaciaacaGaaeqabaqabeGadaaakeaacuWG6bGEgaWcamaaBaaaleaacqWGRbWAaeqaaaaa@2FC6@, which is closely related to the system theoretical concept of observability [48]. However, since in most relevant cases even the states z→n
 MathType@MTEF@5@5@+=feaafiart1ev1aaatCvAUfKttLearuWrP9MDH5MBPbIqV92AaeXatLxBI9gBaebbnrfifHhDYfgasaacH8akY=wiFfYdH8Gipec8Eeeu0xXdbba9frFj0=OqFfea0dXdd9vqai=hGuQ8kuc9pgc9s8qqaq=dirpe0xb9q8qiLsFr0=vr0=vr0dc8meaabaqaciaacaGaaeqabaqabeGadaaakeaacuWG6bGEgaWcamaaBaaaleaacqWGUbGBaeqaaaaa@2FCC@ are approximated quite well, this shall not be discussed in detail. Additional explanations and detailed calculations can be found in Additional files 1 and 2.

### The modeling procedure – step by step

The following procedure provides a guide to build reduced models step by step. This procedure is used to generate a large model of insulin signaling that covers all processes discussed in the introduction (Additional file 3).

1. Identify all processes (Figure 4, white boxes) and their interactions (Figure 4, green and red lines).

2. Define layers: all processes that are coupled by graded interactions are within the same layer. Layers are coupled by all-or-none interactions or do not interact (Figure 4, blue boxes).

3. Model each layer individually.

(a) Define all sums of phosphorylated binding sites *x*_*i *_(e.g. *x *= *ROP *+ *RLP*, see Equation 26b) and all sums of occupied binding sites *x*_*i*_*b *(e.g. *xb *= *RXE*). The terminal 'b' indicates that there is something bound to the binding site.

(b) Define the concentrations of all unoccupied phosphorylated binding sites that are needed as binding partners within the considered layer (e.g. *RXp *= *x *- *xb*). These species act as binding partners for binding reactions (see Figure 5).

(c) Define rules and reactions (including dephosphorylation of binding sites) as if there were no other layers and in particular as if there was no binding of effectors (see Figure 5). Use (uppercase) 'P' to indicate phosphorylation of binding sites and use another denotation (e.g.lowercase 'p') to indicate phosphorylation of regulatory sites.

(d) Translate each reaction into the corresponding rate by using the desired kinetic law. This step is analogous to detailed mechanistic modeling. For dephosphorylation reactions of binding sites multiply the expression describing dephosphorylation with (*x*_*i *_- *x*_*i*_*b*)/*x*_*i *_using the appropriate *x*_*i*_. This ensures that only unoccupied binding sites are dephosphorylated (see Equation 26a).

(e) Optional: for each molecule that is not degraded or synthesized a conservation relation can be formulated (e.g. *ROO *= *R*_0 _- *RLO *- *ROP *- *RLP*, see Equation 26b).

(f) Construct ODEs as a sum of rates for each species that is used in this layer and not defined by an algebraic equation (see Equation 26c).

4. Additional information transfer between layers is allowed, as long as no additional graded interactions are introduced (e.g. *R*_*activ *_in Additional file 4).

This procedure also outlines how automation of the modeling procedure can be achieved. Steps 1) to 3c) most probably will be performed by the user, whereas expansion of rules and generation of rates and ODEs could be automated. The modeling procedure then remains in close similarity to automated rule based building of detailed mechanistic models by BioNetGen [19].

### A larger example system

**Figure 8:**
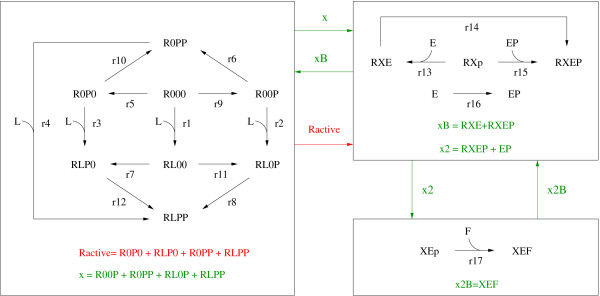
**Layer based modularization of the larger example system**. In the larger example system the left module describes the receptor layer. The output of this layer is the sum of all species which are phosphorylated on the binding site for *E*. This sum is denoted as *x*. The upper right module, which describes the layer of effector *E*, returns the sum of occupied binding sites (*xb*) and sends the sum of all species which are phosphorylated on the binding site for *F *(*x*2). The lower right module, which describes the layer of effector *F*, returns the sum of occupied binding sites *x*2*b*. The sum of catalytically active receptors is denoted as *R*_*activ*_. All reactions are reversible, arrows definne directions of positive rates. For equations see Additional file 4.

**Figure 9:**
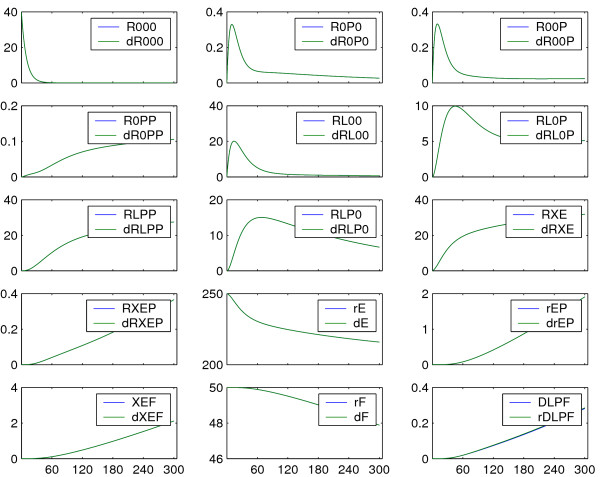
**Simulation results: comparison reduced and detailed model, larger example system**. For parameter values from literature (Additional file 4), the deviations of the lumped states from the corresponding sums of states of the detailed model are negligible. As demonstrated for *DLPF *reconstitution of states of the detailed model is possible with high accuracy. The axis of abscissae is given in *s*, the axis of ordinates in *nM*.

To demonstrate the method on a more realistic example we study an extended subsystem of insulin signaling. This subsystem was also employed to demonstrate the modularization criterion (Figure 4B). Assume that the receptor can bind *L *(insulin) and perform autophosphorylation on two sites, one being a binding site for an effector *E *(IRS) and one being a regulatory phosphorylation site which negatively affects autophosphorylation of the binding site. Effector bound to the receptor can be phosphorylated by the receptor (yielding *RXEP*). Another effector *F *(PI3K) can bind phosphorylated effector *E*. The detailed system is described by 24 equations. If there is no protein synthesis or degradation three of these 24 differential equations (Additional file 4 and 5) can be replaced by conservation relations for the receptor, *E *and *F*, yielding 21 equations for the detailed model. All reactions are visualized in Additional file 4. Reduction and modularization of the model is performed layer based (Figures 4B and 8). The receptor layer contains *L*- binding and receptor autophosphorylation, the *E*- layer contains *E*- binding and phosphorylation. Binding of *F *occurs in the *F*- layer. The receptor layer is described by 2^3 ^= 8 differential equations (2 possibilities each for insulin binding, binding site and regulatory phosphorylation), the *E *layer needs four equations (*E *can be bound and unbound, the binding site can be phosphorylated or unphosphorylated). The *F *layer is described by two equations (bound and unbound *F*). Three of these equations could be replaced by conservation relations, yielding 11 equations for the reduced model. In the layers for *E *and *F *there are lumped states that are defined by algebraic relations and need no differential equation. By convention, in each case we replace the balance of the binding partner of the effector molecule. These are *RXp *in the *E*-layer and *XEp *in the *F *- layer. *RXp *defines all receptor species with phosphorylated and unoccupied binding site for *E*, *XEp *defines all *E*-species with phosphorylated and unoccupied binding site for *F*, *E *being bound to the receptor or not. Simulation with parameters and concentrations from literature was performed and showed a very high quality of the reduction (Figure 9). For model descriptions, parameters, initial conditions and transformation equations see Additional file 4. Note that there is an additional information transfer between the layers of receptor and *E*. The sum of all activated receptor species *R*_*activ *_is transfered to the *E *layer. This macroscopic variable represents the concentration of activated receptor and acts as a catalyst to phosphorylate membrane-located (that is receptor-bound) *E*. This does not disrupt the modularity of the receptor and *E *layers because the concentration of activated receptors can be expressed as a sum of the reduced variables in the receptor layer. No additional graded interaction is introduced by this information transfer. If *E *could be phosphorylated only when bound to an activated receptor, however, this would introduce a graded interaction between the two layers requiring the merging of the receptor and *E *layers. Note that this hypothetical additional interaction in this case is no all-or-none interaction. Receptor activation is a necessary precondition for effector phosphorylation, which resembles to an all-or-none interaction. However, for an all-or-none interaction always two constraints are necessary. The second would be that receptor deactivation can only occur if the effector is unphosphorylated. This will most often not be assumed. Therefore, this hypothetical additional interaction in most cases will be a graded interaction that disrupts the modularity between the layers of receptor and *E*.

#### Optimization study of the larger example system

An optimization study was performed to analyze the worst case scenario within physiologic parameter ranges. Measures for reduction quality are the errors in *XEF *and *DXXF*. *XEF *corresponds to all species of effector *F*, that are bound to phosphorylated effector *E*, be *E *bound to the receptor or not. *DXXF *corresponds to all species of *F *that are bound to the receptor via *E*. In contrast to *XEF*, *DXXF *is not a state of the reduced model. It is the sum of all approximated states of the detailed model, where *F *is bound to the receptor via *E*. Depending on the scenario, both *XEF *and *DXXF *can correspond to activated effector *F *and therefore have physiologic relevance. Reconstruction of states (or sums of states as it is DXXF) of the detailed model is extensively discussed in Additional file 4. Starting from literature values, parameters were varied over four orders of magnitude to maximize the error in *XEF *and *DXXF*. This parameter interval should cover physiologic parameter ranges. Reduction quality is still very high and even the error of *DXXF *is within the range of measurement errors in typical experiments. Worst case parameters and simulation results are shown in Additional file 4.

### Synthesis, degradation and transport of proteins

Up to now it was assumed that there is no protein synthesis or degradation. However, synthesis and degradation of free unmodified proteins is easy to handle. The rate of synthesis or degradation just has to be included in the differential equation for the free species. Degradation or synthesis of complexes is also possible though often not being as easy to realize. If a scaffold protein or even the receptor is to be degraded one has to observe that there exist lumped states in several layers that correspond to complexes with this effector or the receptor. In this case, the rate of degradation or synthesis has to be considered in different layers to guarantee consistency. Now more communication between the layers is necessary. The same rates that are modified by different correction terms to reflect complex composition occur in distinct layers. Transport between different compartments can be handled as degradation in one compartment and synthesis of the same complex in the other. Therefore, synthesis, degradation and transport of complexes is possible with the layer based formalism. Concise modular structure of the model with a minimum of information transfer between the layers is yielded when these processes are limited to free protein species.

### Outline: application on insulin signaling

A model for the insulin signaling system which includes all events that were mentioned in the introduction can be built with 64 + 128 + 4 + 11 + 5 + 2 = 214 differential equations instead of 1.5·10^8 ^in the detailed case. The 214 equations for the reduced model are composed as follows. 2^6 ^= 64 equations in the receptor layer describe binding of two insulin molecules and phosphorylation of four binding sites (two for Shc and two for IRS). The 2^7 ^= 128 equations of the IRS- layer derive from six binding sites, each of them can be phosphorylated and unphosphorylated. IRS can be bound to the receptor and unbound. 4 equations are needed for the Shc layer (Shc binding to the receptor and becoming phosphorylated). SOS and Grb2 are merged into one layer. This allows SOS binding to Grb2 influencing Grb2 binding to IRS and Shc. The corresponding layer is described by 11 equations (six equation describing binding of complexes of Grb2 and SOS to IRS and Shc, five equations for free species, remember that SOS can be phosphorylated). The PI3K layer contains 5 differential equations (binding to four binding sites) and the SHP2 layer 2 (binding to IRS). Free species are considered. The two binding sites on the receptor for Shc and IRS in each case are assumed to be equivalent. Other underlying assumptions are specified in Table 1. Layer based reduced modeling of this system is demonstrated in Additional files 3 and 6.

### Reduction ratios and combination with domain-oriented lumping

As shown above for insulin signaling combinatorial complexity increases as the effector is subject of additional modification and binding events. The reduction potential of the layer based reduced modeling method strongly increases with increasing combinatorial complexity. For the small example system the number of equations is reduced by 20%, for the larger example system by 48%. The fraction of necessary equations for the signaling system as described in the introduction is reduced by 99.9999 %. This illustrates that even large systems can be described with a number of equations that can be handled by manual modeling. However, molecules with many binding sites or regulatory phosphorylation sites are still difficult to handle. Here combination with the domain-oriented lumping technique [18] is possible. An example scenario is analyzed, underlying assumptions are specified in Table 1. There, case 1) is a very general setting, while case 2) is one of the most simple realistic special cases. In this case it is assumed that the phosphorylation state of binding sites does not influence phosphorylation of other binding sites on the same molecule. The layer based formalism requires still 214 equation, domain-oriented lumping requires 212 equations, the combination of both methods yields a model with only 56 equations. To combine both approaches, first the layer based model is generated. Then the domain-oriented approach is applied to each layer separately (see Additional files 7 and 8).

So, combination of layer based reduced modeling with the domain-oriented approach [18] is possible. Under specific conditions, this results in an additional decrease of the fraction of necessary equations. The ideal scenario for combining both methods is the occurrence of molecules with many sites, where these sites do not or sparely interact. Then domain-oriented lumping allows further strong reduction of the layer based reduced model. Reduction quality stays the same as the domain-oriented approach provides exact lumping.

## Conclusion

We present a reduced modeling approach which allows to tackle the problem of combinatorial complexity in signal transduction and regulation networks. For physiologic systems combinatorial complexity is dramatically decreased, as demonstrated on insulin signaling. Similarly to Pawson and Nash [23], Borisov et al. [22] and Conzelmann et al. [18] our method aims at a more macroscopic description of a system using levels of occupancy and phosphorylation.

A modularization principle is introduced. There, one has to distinguish between graded interactions and all-or-none interactions. Modules, which correspond to layers of signal transduction, are chosen such that they only interact via all-or-none interactions. All processes inside layers are connected directly or indirectly via graded interactions. For each layer we formulate a detailed reaction scheme comprising all processes of the current layer but neglecting all other processes. In the subsequent modeling step one has to formulate gross reaction rates for all of these reactions. A step by step procedure for building reduced modular models is given.

A potential drawback of the method is that even small changes to the assumptions of the model may lead to merging of layers which results in lower decrease of combinatorial complexity. This is the case if additional graded interactions between processes of different layers are introduced.

Mathematical analysis as well as simulation and optimization studies using insulin signaling models show that the approximation quality is excellent for relevant parameter settings. In the considered examples it is even possible to reconstruct the eliminated micro-states with high accuracy. As we also showed the method allows an enormous reduction of the number of necessary ODEs compared with detailed combinatorial models.

The method can be combined with the domain-oriented lumping technique [18], which can result in further reduction of the model.

## Methods

### Initial conditions

The total concentration of insulin receptor in hepatocytes was reported to be 10^5 ^receptors per cell [49]. A hepatocyte is assumed to be a ball with diameter 20 *μ*m. This ball has a volume of 4.2·10^-12 ^*l*. With one mol standing for 6.0221415·10^23 ^molecules there are 1.66·10^-19 ^*mol *receptor per cell. This is a concentration of 40 nM. The total concentration for IRS (*E*) was assumed to be the same as for Shc, which is 250 *nM *[9]. The total concentration for PI3K (*F*) was taken from literature [9]. It is assumed that the signalling system is totally inactive at the beginning. Initial conditions other than zero but close to zero (10^-20 ^*nM*) for the states which should start with an initial condition of zero were chosen. This prevents division by zero in correction terms of the rates of the reduced models and in the computations for consistent initial values. The states of the reduced model were chosen consistently such that transformation equations hold.

### Parameter values

Insulin receptor autophosphorylation *in vitro *has a half-life of about 0.5 *min *(Figure 1 in [50]). Assuming first order kinetics, this corresponds to a rate constant of 0.0231 *s*^-1^. Insulin receptor dephosphorylation on the plasma membrane in vitro has a half-life of about 3 *min *(Figure 2 in [51]). Assuming first order kinetics, this corresponds to a rate constant of 0.00385 *s*^-1^. Parameters describing binding of IRS to the insulin receptor were originally reported to describe the binding of the p85 subunit to IRS [50].

## Abbreviations

nM, nano molar (10^-9 ^*mol*·*l*^-1^); ass., assumption; ODE, ordinary differential equation

## Competing interests

The author(s) declares that there are no competing interests.

## Authors' contributions

MK developed the method, provided the example systems and performed the optimization study. HC performed the mathematical analysis and participated in development of the method. ME took part in mathematical analysis and development of the method. SE took part in development of the method. EDG initiated and supervised the study. All authors approved the final manuscript.

## Supplementary Material

Additional file 1 Transformation and stationary error Click here for file

Additional file 2 Transformation and stationary error Click here for file

Additional file 3 Layer based reduced modeling of insulin signaling Click here for file

Additional file 4 Documentation and analysis of the larger example system Click here for file

Additional file 5 Layer based reduced model of the larger example system Click here for file

Additional file 6 Layer based reduced model of insulin signaling (214 ODEs) Click here for file

Additional file 7 Application of the domain oriented approach to the insulin model with 214 ODEs Click here for file

Additional file 8 Reduced model resulting from combination of both approaches (56 ODEs) Click here for file

## References

[B1] Sackmann A, Heiner M, Koch I (2006). Application of Petri net based analysis techniques to signal transduction pathways. BMC Bioinformatics.

[B2] Klamt S, Saez-Rodriguez J, Lindquist JA, Simeoni L, Gilles ED (2006). A methodology for the structural and functional analysis of signaling and regulatory networks. BMC Bioinformatics.

[B3] Gillespie DT (1992). A rigorous derivation of the chemical master equation. Physica A.

[B4] Asthagiri A, Lauffenburger D (2001). A computational study of feedback effects on signal dynamics in a mitogen-activated protein kinase (MAPK) pathway model. Biotechnol Prog.

[B5] Hatakeyama M, Kimura S, Naka T, Kawasaki T, Yumoto N, Ichikawa M, Kim JH, Saito K, Saeki M, Shirouzu M, Yokoyama S, Konagaya A (2003). A computational model on the modulation of mitogen-activated protein kinase (MAPK) and Akt pathways in heregulin-induced ErbB signalling. Biochem J.

[B6] Haugh J, Schooler K, Well A, Wiley H, Lauffenburger D (1999). Effect of epidermal growth factor receptor internalization on regulation of the phosphoslipase C-gamma1 signaling pathway. J Biol Chem.

[B7] Haugh J, Well A, Lauffenburger D (2000). Mathematical modeling of epidermal growth factor receptor signaling through the phospholipase C pathway: mechanistic insights and predictions for molecular interventions. Biotechnol Bioeng.

[B8] Kholodenko BN, Demin OV, Moehren G, Hoek JB (1999). Quantification of Short Term Signaling by the Epidermal Growth Factor Receptor. J Biol Chem.

[B9] Moehren G, Markevich N, Demin O, Kiyatkin A, Goryanin I, Hoek JB, Kholodenko BN (2002). Temperature dependence of the epidermal growth factor receptor signaling network can be accounted for by a kinetic model. Biochemistry.

[B10] Schoeberl B, Eichler-Jonsson C, Gilles ED, Müller G (2002). Computational modeling of the dynamics of the MAP kinase cascade activated by surface and internalized EGF receptors. Nat Biotechnol.

[B11] Sedaghat AR, Sherman A, Quon MJ (2002). A mathematical model of metabolic insulin signaling pathways. Am J Physiol Endocrinol Metab.

[B12] Faeder JR, Hlavacek WS, Reischl I, Blinov ML, Metzger H, Redondo A, Wofsy C, Goldstein B (2003). Investigation of early events in Fc(epsilon) RI-mediated signaling using a detailed mathematical model. J Immunol.

[B13] Blinov ML, Faeder JR, Goldstein B, Hlavacek WS (2006). A network model of early events in epidermal growth factor receptor signaling that accounts for combinatorial complexity. Biosystems.

[B14] Hlavacek WS, Faeder JR, Blinov ML, Perelson AS, Goldstein B (2004). The complexity of complexes in signal transduction. Biotechnol Bioeng.

[B15] Morton-Firth CJ, Bray D (1998). Predicting temporal fluctuations in an intracellular signalling pathway. J Theor Biol.

[B16] Shimizu TS, Novere NL, Levin MD, Beavil AJ, Sutton BJ, Bray D (2000). Molecular model of a lattice of signalling proteins involved in bacterial chemotaxis. Nat Cell Biol.

[B17] Faeder JR, Blinov ML, Goldstein B, Hlavacek WS (2005). Combinatorial complexity and dynamical restriction of network flows in signal transduction. IEE Systems Biology.

[B18] Conzelmann H, Saez-Rodriguez J, Sauter T, Kholodenko B, Gilles E (2006). A domain-oriented approach to the reduction of combinatorial complexity in signal transduction networks. BMC Bioinformatics.

[B19] Blinov ML, Faeder JR, Goldstein B, Hlavacek WS (2004). BioNetGen: software for rule-based modeling of signal transduction based on the interactions of molecular domains. Bioinformatics.

[B20] Faeder JR, Blinov ML, Hlavacek WS (2005). Graphical rule-based representation of signal transduction networks. Proc ACM Symp Appl Computing.

[B21] Blinov ML, Yang J, Faeder JR, Hlavacek WS (2005). Graph theory for rule-based modeling of biochemical networks. Proceedings of BioCONCUR 2005, A workshop on concurrent models in molecular biology.

[B22] Borisov NM, Markevich NI, Hoek JB, Kholodenko BN (2005). Signaling through receptors and scaffolds: independent interactions reduce combinatorial complexity. Biophys J.

[B23] Pawson T, Nash P (2003). Assembly of cell regulatory systems through protein interaction domains. Science.

[B24] Khan A, Pessin J (2002). Insulin regulation of glucose uptake: a complex interplay of intracellular signalling pathways. Diabetologia.

[B25] Saltiel AR, Pessin JE (2002). Insulin signaling pathways in time and space. Trends Cell Biol.

[B26] Saltiel A, Kahn C (2001). Insulin signalling and the regulation of glucose and lipid metabolism. Nature.

[B27] Taniguchi CM, Emanuelli B, Kahn CR (2006). Critical nodes in signalling pathways: insights into insulin action. Nat Rev Mol Cell Biol.

[B28] Chang L, Chiang SH, Saltiel AR (2004). Insulin signaling and the regulation of glucose transport. Mol Med.

[B29] Plum L, Belgardt BF, Brüning JC (2006). Central insulin action in energy and glucose homeostasis. J Clin Invest.

[B30] Mounier C, Posner BI (2006). Transcriptional regulation by insulin: from the receptor to the gene. Can J Physiol Pharmacol.

[B31] Leng Y, Karlsson HKR, Zierath JR (2004). Insulin signaling defects in type 2 diabetes. Rev Endocr Metab Disord.

[B32] Chakraborty C (2006). Biochemical and molecular basis of insulin resistance. Curr Protein Pept Sci.

[B33] Ludvigsson J (2006). Why diabetes incidence increases – a unifying theory. Ann N Y Acad Sci.

[B34] Musi N, Goodyear LJ (2006). Insulin resistance and improvements in signal transduction. Endocrine.

[B35] Salsali A, Nathan M (2006). A review of types 1 and 2 diabetes mellitus and their treatment with insulin. Am J Ther.

[B36] Stumvoll M, Goldstein BJ, van Haeften TW (2005). Type 2 diabetes: principles of pathogenesis and therapy. Lancet.

[B37] Hirsch IB (2005). Insulin analogues. N Engl J Med.

[B38] Luo R, Beniac D, Fernandes A, Yip C, Ottensmeyer F (1999). Quaternary structure of the insulin-insulin receptor complex. Science.

[B39] White M (1998). The IRS-signalling system: a network of docking proteins that mediate insulin action. Mol Cell Biochem.

[B40] Gual P, Marchand-Brustel YL, Tanti JF (2005). Positive and negative regulation of insulin signaling through IRS-1 phosphorylation. Biochimie.

[B41] Pirola L, Johnston A, Obberghen EV (2004). Modulation of insulin action. Diabetologia.

[B42] Hartwell L, Hopfield J, Leibler S, Murray A (1999). From molecular to modular cell biology. Nature.

[B43] Saez-Rodriguez J, Kremling A, Conzelmann H, Bettenbrock K, Gilles ED (2004). Modular Analysis of Signal Transduction Networks. IEEE Contr Syst Mag.

[B44] Saez-Rodriguez J, Kremling A, Gilles ED (2005). Dissecting the puzzle of life: Modularization of signal transduction networks. Comput Chem Eng.

[B45] Ederer M, Sauter T, Bullinger E, Gilles ED, Allgöwer F (2003). An Approach for Dividing Models of Biological Reaction Networks into Functional Units. Simulation.

[B46] Borisov NM, Markevich NI, Hoek JB, Kholodenko BN (2006). Trading the micro-world of combinatorial complexity for the macro-world of protein interaction domains. Biosystems.

[B47] Heinrich R, Schuster S (1996). The Regulation of Cellular Systems.

[B48] Isidori A Nonlinear control systems, 1995 chap Global decomposition of control systems.

[B49] Rother K, Imai Y, Caruso M, Beguinot F, Formisano P, Accili D (1998). Evidence that IRS-2 phosphorylation is required for insulin action in hepatocytes. J Biol Chem.

[B50] Faure R, Baquiran G, Bergeron J, Posner B (1992). The dephosphorylation of insulin and epidermal growth factor receptors. Role of endosome-associated phosphotyrosine phosphatase(s). J Biol Chem.

[B51] Drake P, Bevan A, Burgess J, Bergeron J, Posner B (1996). A role for tyrosine phosphorylation in both activation and inhibition of the insulin receptor tyrosine kinase in vivo. Endocrinology.

[B52] Wanant S, Quon M (2000). Insulin receptor binding kinetics: modeling and simulation studies. J Theor Biol.

[B53] Felder S, Zhou M, Hu P, Ureña J, Ullrich A, Chaudhuri M, White M, Shoelson S, Schlessinger J (1993). SH2 domains exhibit high-affinity binding to tyrosine-phosphorylated peptides yet also exhibit rapid dissociation and exchange. Mol Cell Biol.

