# Supplementary material for: Reduced modeling of signal transduction – a modular approach
Source: BMC Bioinformatics. 2007 Sep 13;8:336. doi: 10.1186/1471-2105-8-336 (PMC2216040; doi:10.1186/1471-2105-8-336)
Supplement: Additional file 2 — Transformation and stationary error [file 1471-2105-8-336-S2.pdf]

Level−based model reduction

1. Retrieving microstates from a macro−description

Borisov et al. introduced a new viewpoint of modeling signaling networks, namely a domain−oriented approach, which has been formalized in Conzelmann et al. In addition Borisov et al. have shown that in the case of completely independent binding domains all micro−states can be retrieved from a macro−description using probability calculations. Here we want to review the finding of Borisov et al and show how this concept can be extend and used for model reduction.

1.1 Simple Example / Definitions

We start considering the most simple example of a signaling network. A transmembrane receptor D can be activated by an extracellular ligand L and in turn is phosphorylated at another intracellular domain. In a first step we assume that ligand binding and phosphorylation are completely independent.

In[310]:=

```
(*Definition of all microstates*)Vars1stExpMicro = {D00[t], DL0[t], D0P[t], DLP[t]};

(*Definition of reaction rates using the mass action law*)

ReactionRates1stExp = {d1 → k1 * D00[t] - kd1 * DL0[t], d2 → k2 * D00[t] - kd2 * D0P[t],
   d3 → k3 * D0P[t] - kd3 * DLP[t], d4 → k4 * DL0[t] - kd4 * DLP[t]};

(*Parameter definition for completely independent domains*)

Parameters1stExpIndep = {k3 → k1, kd3 → kd1, k4 → k2, kd4 → kd2};
(*Parameter definition for unidirectional
  influence between the two binding domains*)
Parameters1stExpUni = {k3 → k1, kd3 → kd1};

(*1 st Output:Differential equantions for the microstates D00[t],
 DL0[t],D0P[t] und DLP[t]*)
ODE1stExp = {D00'[t] → -d1 - d2, DL0'[t] → d1 - d4, D0P'[t] → d2 - d3, DLP'[t] → d3 + d4};
ODE1stExpIndep = ODE1stExp /. ReactionRates1stExp /. Parameters1stExpIndep;
ODE1stExpIndep // MatrixForm

(*Definition of transformed system states according to Conzelmann
  et al.These states are required because the probability calculations
  in Borisov et al.are formulated using these lumped states.*)

Vars1stExpMacro = {DXX[t], DLX[t], DXP[t], DLP[t]};
Transformation1stExp = {DXX[t] → D00[t] + DL0[t] + D0P[t] + DLP[t],
   DLX[t] → DL0[t] + DLP[t], DXP[t] → D0P[t] + DLP[t], DLP[t] → DLP[t]};
TransformationMatrix = Outer[D, Vars1stExpMacro /. Transformation1stExp,
   Vars1stExpMicro];
InvTransformation = (Vars1stExpMicro[[#]] →
      (Inverse[TransformationMatrix].Vars1stExpMacro)[[#]]) & /@ Range[4];
ODE1stExpTransformed = D[Vars1stExpMacro[[#]], t] →
     FullSimplify[(TransformationMatrix.D[Vars1stExpMicro, t] /. ODE1stExpIndep) /.
        InvTransformation][[#]] & /@ Range[4];

(*2 nd Output:Transformed model equations*)
ODE1stExpTransformed // MatrixForm
```

Out[316]//MatrixForm=

$$\begin{pmatrix} D00'[t] \to -k1\,D00[t] - k2\,D00[t] + kd2\,D0P[t] + kd1\,DL0[t] \\ DL0'[t] \to k1\,D00[t] - k2\,DL0[t] - kd1\,DL0[t] + kd2\,DLP[t] \\ D0P'[t] \to k2\,D00[t] - k1\,D0P[t] - kd2\,D0P[t] + kd1\,DLP[t] \\ DLP'[t] \to k1\,D0P[t] + k2\,DL0[t] - kd1\,DLP[t] - kd2\,DLP[t] \end{pmatrix}$$

Out[322]//MatrixForm=

$$\begin{pmatrix} DXX'[t] \to 0 \\ DLX'[t] \to -(k1 + kd1)\,DLX[t] + k1\,DXX[t] \\ DXP'[t] \to -(k2 + kd2)\,DXP[t] + k2\,DXX[t] \\ DLP'[t] \to -(k1 + k2 + kd1 + kd2)\,DLP[t] + k2\,DLX[t] + k1\,DXP[t] \end{pmatrix}$$

1.2 Error Equation

Borisov et al. formulated the following algebraic interrelationship between macro - states and micro - states : $DLP[t] = \frac{DLX[t] * DXP[t]}{DXX[t]}$. They also showed that if the initial conditions fulfill this equation, it holds for all times $t > 0$. We rewrite this equation in a slightly modified form as $DLP[t] * DXX[t] - DLX[t] * DXP[t] = 0$, and we translate it into a formulation using the original micro - states. If we assume that the initial conditions may not fulfill this equation, an error function g can be defined $g = DLP[t] * DXX[t] - DLX[t] * DXP[t]$. It can be shown that this function g fulfills a linear ordinary differential equation.

In[323]:=

```
(*Transformation of the error function.1
  st Output:Transformed error function*)TransformedG[t] =
 FullSimplify[g[t] → DXX[t] * DLP[t] - DLX[t] * DXP[t] /. Transformation1stExp]

(*Ordinary differential equation for g[t].g[t] is differentiated
   and the time derivatives of the micro-states are replaced by their
   differential equations.2 nd/3 rd Output:Differential equation for g[t].*)

ODEforG[t] = FullSimplify[D[TransformedG[t], t] /. ODE1stExpIndep]
ODEforG[t] /. {DLP[t] → (g[t] + D0P[t] * DL0[t]) / D00[t]}
```

Out[323]=

```
g[t] → -D0P[t] DL0[t] + D00[t] DLP[t]
```

Out[324]=

```
g′[t] → (k1 + k2 + kd1 + kd2) (D0P[t] DL0[t] - D00[t] DLP[t])
```

Out[325]=

```
g′[t] → -(k1 + k2 + kd1 + kd2) g[t]
```

This ordinary differential equation for g[t] is homogene and stable. This is the proof that if the initial conditions do fulfill g[t]=0, the equation of Borisov et al. holds for all t>0. Even if the initial conditions do not fulfill g[t]=0 Borisov’s equation will give a very good approximation for all t>t0, since the system tends exponentially to g[t]=0.
Importantly, if the two binding domains are not completely independent, no such global conclusions can be made. However, Borisov et al. have shown that g[t]=0 is a quite good approximation in a number of relevant cases.

2. Model reduction

Now we want to extend the considerations mode in Borisov et al. In the following we again consider a receptor with 2 binding sites. However, in addition to ligand binding and receptor phosphorylation of the intracellular domain also effector binding to the phosphorylated domain shall be incorporated. Importantly, we first again assume that all binding events are completely independent.

In[326]:=
```
(*Definition of extended model 1*)
ReactionRatesExtModel = {d1 → k1 * D00[t] - kd1 * DL0[t],
   d2 → k2 * D00[t] - kd2 * D0P[t], d3 → k3 * D0P[t] - kd3 * DLP[t],
   d4 → k4 * DL0[t] - kd4 * DLP[t], d5 → k5 * D0P[t] - kd5 * D0E[t],
   d6 → k6 * DLP[t] - kd6 * DLE[t], d7 → k7 * D0E[t] - kd7 * DLE[t]};

ODEListExtModel = {D00'[t] → -d1 - d2, DL0'[t] → d1 - d4,
   D0P'[t] → d2 - d5 - d3, DLP'[t] → d3 + d4 - d6, D0E'[t] → d5 - d7, DLE'[t] → d6 + d7};

(*Parameter values for completely independent binding events*)

ParametersExtModelIndep =
  {k3 → k1, kd3 → kd1, k7 → k1, kd7 → kd1, k4 → k2, kd4 → kd2, k6 → k5, kd6 → kd5};
(*Parameter values for ligand induced phosphorylation.Effector
  binding is independent of ligand binding.*)

ParametersExtModelUni = {k3 → k1, kd3 → kd1, k7 → k1, kd7 → kd1, k6 → k5, kd6 → kd5};

VarsExtModel = {D00[t], DL0[t], D0P[t], DLP[t], D0E[t], DLE[t]};
ODEExtModelRates = D[VarsExtModel, t] /. ODEListExtModel;
ODEExtModelEqn = ODEExtModelRates /. ReactionRatesExtModel;
ODEExtModelList = D[VarsExtModel, t][[#]] → ODEExtModelEqn[[#]] & /@ Range[6];

(*Model equations for simulation*)

ODEExtModelSolve = D[VarsExtModel, t][[#]] == ODEExtModelEqn[[#]] & /@ Range[6];
ic = {D00[0] == 1, D0P[0] == 0, DL0[0] == 0, DLP[0] == 0, D0E[0] == 0, DLE[0] == 0};
ODEExtModelSim = Join[ODEExtModelSolve, ic];

(*Steady state solution of extended model*)

SteadyStateExtModel = FullSimplify[Solve[Join[(ODEExtModelEqn[[#]] == 0 & /@ Range[5]),
     {(Plus @@ VarsDetailed) == D0}], VarsDetailed]];
```

In the case of completely independent domains, one can formulate three error equations:

```
    g1[t]=D00[t]*DLP[t]-D0P[t]*DL0[t]
    g2[t]=D00[t]*DLE[t]-D0E[t]*DL0[t]
    g3[t]=D0P[t]*DLE[t]-DLP[t]*D0E[t]
```

g1[t], g2[t] and g3[t] are error functions describing how much the assumptions g1[t]=g2[t]=g3[t]=0 is violated. Interestingly, g1, g2 and g3 are independent (that means that non of the three errors can be calculated if the other two errors are known. However, if two of the equations g1[t]=0, g2[t]=0 and g3[t]=0 are fulfilled the third equation is also fulfilled!
Now we will show that these three error functions fulfill a homogene linear ordinary differential equation.

In[338]:=

```
(*Definition of the three error functions*)
ErrorFunctions = {g1[t] == D00[t] * DLP[t] - D0P[t] * DL0[t],
   g2[t] == D0P[t] * DLE[t] - DLP[t] * D0E[t], g3[t] == D00[t] * DLE[t] - D0E[t] * DL0[t]};

ErrorFunctionsReplList = Flatten[Solve[ErrorFunctions, {g1[t], g2[t], g3[t]}]];
ReplVars = Flatten[Solve[ErrorFunctions, {DLE[t], D00[t], D0P[t]}]];
(*Formulation of the linear error ODEs.Same procedure as shown
  above.1 st Output:Linear error ODEs in g1[t],g2[t] and g3[t]*)
ErrorODEs = FullSimplify[D[ErrorFunctionsReplList, t] /. ODEExtModelList /.
    ParametersExtModelIndep];

ErrorODEsG = FullSimplify[ErrorODEs /. ReplVars];

ErrorODEsG // MatrixForm
```

Out[342]//MatrixForm=

$$\begin{pmatrix} g1'[t] \to -(k1 + k2 + k5 + kd1 + kd2)\, g1[t] + kd5\, g3[t] \\ g2'[t] \to -(k1 + k5 + kd1 + kd2 + kd5)\, g2[t] + k2\, g3[t] \\ g3'[t] \to k5\, g1[t] + kd2\, g2[t] - (k1 + k2 + kd1 + kd5)\, g3[t] \end{pmatrix}$$

In[343]:=

```
(*Eigenvalues of the linear ODE-system.The Error-
  ODEs are always stable.2 nd Output:Eigenvalues of the error ODEs.*)
 A = Outer[D, {g1'[t], g2'[t], g3'[t]} /. ErrorODEsG, {g1[t], g2[t], g3[t]}];

Eig = Eigenvalues[A]

(*Obviously the biggest eigenvalue is the third one in the list below.However,
 it is always smaller than-k1-kd1! Hence,
 the error always decays exponentially with an exponent smaller than-
  k1-kd1! 3 rd Output:Inequality λ3<-k1-kd1*)

FullSimplify[Eig[[3]] < -k1 - kd1]
```

Out[344]=

$$\left\{-k1-k2-k5-kd1-kd2-kd5,\ \frac{1}{2}\left(-2\,k1-k2-k5-2\,kd1-kd2-kd5-\sqrt{k2^2-2\,k2\,k5+k5^2+2\,k2\,kd2+2\,k5\,kd2+kd2^2-2\,k2\,kd5+2\,k5\,kd5-2\,kd2\,kd5+kd5^2}\right),\ \frac{1}{2}\left(-2\,k1-k2-k5-2\,kd1-kd2-kd5+\sqrt{k2^2-2\,k2\,k5+k5^2+2\,k2\,kd2+2\,k5\,kd2+kd2^2-2\,k2\,kd5+2\,k5\,kd5-2\,kd2\,kd5+kd5^2}\right)\right\}$$

Out[345]=

$$\sqrt{k2^2+(k5+kd2)^2+2\,(k5-kd2)\,kd5+kd5^2-2\,k2\,(k5-kd2+kd5)} < k2+k5+kd2+kd5$$

In[346]:=

```
(* If the resulting inequality is squared and simplified,
 it can be shown that it is always fulfilled if k2,k5,
 kd2 and kd5 are bigger than zero (4 th Output)! *)
FullSimplify[(√(k2^2 + k5^2 + (kd2 - kd5)^2 - 2 k2 (k5 - kd2 + kd5) + 2 k5 (kd2 + kd5)))^2 <
  (k2 + k5 + kd2 + kd5)^2, k2 > 0 && kd2 > 0 && k5 > 0 && kd5 > 0]
```

Out[346]=
True

In[347]:=

We can show (see above) that for independent binding events the error functions fulfill the linear ODE−system $\frac{d\vec{g}}{dt}=A\vec{g}$. Now we want to consider the case in which ligand binding induces receptor phoshorylation. Here we want to show that in this case the ODE−system looks like $\frac{d\vec{g}}{dt}=A\vec{g}+Bu$.

In[348]:=
```
ErrorODEsUniModel = FullSimplify[
   D[ErrorFunctionsReplList, t] /. ODEExtModelList /. ParametersExtModelUni];

(* The term B*u can be calculated by subtracting the old ODEs from the independent
  case (A*g) from the new ODEs here. 1 st Output: The term B*u. *)
FullSimplify[({g1'[t], g2'[t], g3'[t]} /. ErrorODEsUniModel) -
   ({g1'[t], g2'[t], g3'[t]} /. ErrorODEs)] // MatrixForm
```

Out[349]//MatrixForm=

$$\begin{pmatrix} -(D00[t] + D0P[t])\,((k2 - k4)\,DL0[t] + (-kd2 + kd4)\,DLP[t]) \\ D0E[t]\,((k2 - k4)\,DL0[t] + (-kd2 + kd4)\,DLP[t]) \\ D0E[t]\,((-k2 + k4)\,DL0[t] + (kd2 - kd4)\,DLP[t]) \end{pmatrix}$$

Interestingly, this input vector can be written as
$(D00[t] + D0P[t], -D0E[t], D0E[t])^T * (k2 - k4)\,DL0[t] - (kd2 - kd4) * DLP[t]$.
The term $(k2 - k4)\,DL0[t] - (kd2 - kd4) * DLP[t]$ can be interpreted as a flux difference. The difference between the flux which would occur in a completely independent reaction network and this reaction network. Additionally, it is obvious that if the ligand has a high affinity to the receptor, the norm of the vector $(D00[t] + D0P[t], -D0E[t], D0E[t])^T$ will become very small.

2.1 The stationary error

In the following we will consider the stationary error of the example considered above, and we will show that the stationary error vanishes if the model fullfils the detailed balance constraint. Additionally, we show that in other cases the stationary error shrinks with growing values of parameters.

In[350]:=
```
(*1 st Assumption:ligand binding and effector binding are independent.*)

Parameters1stAssump = {k7 -> k3, kd7 -> kd3, k6 -> k5, kd6 -> kd5};

(*Calculation of the stationary solutions*)

StationaryEqn = Join[(ODEExtModelEqn /. Parameters1stAssump)[[#]] == 0 & /@ Range[5],
   {D00[t] + DL0[t] + D0P[t] + DLP[t] + D0E[t] + DLE[t] == D0}];
SteadyStateSol = FullSimplify[Solve[StationaryEqn,
    {D00[t], DL0[t], D0P[t], DLP[t], D0E[t], DLE[t]}]];

(*1 st Output:Stationary error of g2[t].This error function is chosen since it is
    the only relation that is used in the level based reduction approach.*)
StationaryErrorEqn = FullSimplify[D0P[t] * DLE[t] - DLP[t] * D0E[t] /. SteadyStateSol]
```

Out[353]=

$$\{-(D0^2\,k5\,(k1\,k4\,kd2\,kd3 - k2\,k3\,kd1\,kd4)\,kd5\,((k3 + kd3)\,(k2\,(k4 + kd1)\,(k3 + k5 + kd3) + k1\,k4\,(k3 + k5 + kd2 + kd3) + k2\,kd1\,kd4) + (k2\,(k4 + kd1)\,(k3 + kd3) + k1\,k4\,(k3 + kd2 + kd3) + k2\,kd1\,kd4)\,kd5)) \Big/ (k5\,(k3 + kd3)\,(k2\,(k4 + kd1)\,(k3 + k5 + kd3) + k1\,k4\,(k3 + k5 + kd2 + kd3) + k2\,kd1\,kd4) + ((k4 + kd1)\,(kd2\,kd3\,(k3 + k5 + kd3) + k2\,(k3 + kd3)\,(k3 + 2\,k5 + kd3)) + (k3\,kd1\,(k3 + k5 + kd2) + kd1\,(k3 + kd2)\,kd3 + k2\,(k3 + kd1)\,(k3 + k5 + kd3))\,kd4 + k1\,(k4\,(kd3\,(kd2 + kd3) + k5\,(kd2 + 2\,kd3)) + k3^2\,(k4 + kd4) + kd2\,kd3\,(k5 + kd3 + kd4) + k3\,(kd2\,kd3 + k4\,(2\,k5 + kd2 + 2\,kd3) + (k5 + kd2 + kd3)\,kd4)))\,kd5 + ((k4 + kd1)\,kd2\,kd3 + k2\,(k4 + kd1)\,(k3 + kd3) + k2\,(k3 + kd1)\,kd4 + kd1\,(k3 + kd2)\,kd4 + k1\,(k4\,(kd2 + kd3) + k3\,(k4 + kd4) + kd2\,(kd3 + kd4)))\,kd5^2)^2\}$$

In[354]:=
```
(*
  2 nd Assumption: Detailed balance is fullfiled in the
     network! In the network there are 2 detailed balace relations:
```
$\frac{k1*kd2*kd3*k4}{kd1*k2*k3*kd4}=1$ und $\frac{k3*kd5*k6*kd7}{kd3*k5*kd6*k7}=1$.
```
    With the 1 st Assumption (above) the
    second detailed balance relation is already fullfiled.
*)
```

In[355]:=

```
Parameters2ndAssump =
  {k1 → kd1 * k2 * k3 * kd4 / (kd2 * kd3 * k4), k7 → k3, kd7 → kd3, k6 → k5, kd6 → kd5};

(* 1 st Output: Stationary error under the 2 nd Assumption *)
StationaryErrorEqn /. Parameters2ndAssump
```

Out[356]=

```
{0}
```

In[357]:=

```
(*
 In a last scenario we want to reconsider the case that ligand binding
  has an unidirectional influence on receptor phosphorylation ,
 but no direct influence on effector binding. In this scenario
  the 1 st detailed balance constraint is not fullfiled. However,
 the stationary error quadratically decays with growing values of k1,
 kd1, k5 and kd5 . In the following we consider the stationary error,
 which is a fraction. We calculate the order of numerator
  and denominator in the different kinetic parameters.
*)
```

In[358]:=

```
(* 1 st Output: Order of numerator considering
    k1. 2 nd Output: Order of denominator considering k1.
       3 rd Output: Order of numerator considering kd1. 4 th
        Output: Order of denominator considering kd1. *)
Length[CoefficientList[Numerator[StationaryErrorEqn[[1]] /. ParametersExtModelUni],
  k1]]
Length[CoefficientList[Denominator[
   StationaryErrorEqn[[1]] /. ParametersExtModelUni], k1]]
Length[CoefficientList[Numerator[StationaryErrorEqn[[1]] /. ParametersExtModelUni],
  kd1]]
Length[CoefficientList[Denominator[
   StationaryErrorEqn[[1]] /. ParametersExtModelUni], kd1]]
```

Out[358]=

```
5
```

Out[359]=

```
7
```

Out[360]=

```
5
```

Out[361]=

```
7
```

In[362]:=

```
(* 1 st Output: Order of numerator considering
   k2. 2 nd Output: Order of denominator considering k2.
      3 rd Output: Order of numerator considering kd2. 4 th
       Output: Order of denominator considering kd2. *)
Length[CoefficientList[Numerator[StationaryErrorEqn[[1]] /. ParametersExtModelUni],
  k2]]
Length[CoefficientList[Denominator[
   StationaryErrorEqn[[1]] /. ParametersExtModelUni], k2]]
Length[CoefficientList[Numerator[StationaryErrorEqn[[1]] /. ParametersExtModelUni],
  kd2]]
Length[CoefficientList[Denominator[
   StationaryErrorEqn[[1]] /. ParametersExtModelUni], kd2]]
```

Out[362]=
3

Out[363]=
3

Out[364]=
3

Out[365]=
3

In[366]:=

```
(* 1 st Output: Order of numerator considering
   k4. 2 nd Output: Order of denominator considering k4.
      3 rd Output: Order of numerator considering kd4. 4 th
       Output: Order of denominator considering kd4. *)
Length[CoefficientList[Numerator[StationaryErrorEqn[[1]] /. ParametersExtModelUni],
  k4]]
Length[CoefficientList[Denominator[
   StationaryErrorEqn[[1]] /. ParametersExtModelUni], k4]]
Length[CoefficientList[Numerator[StationaryErrorEqn[[1]] /. ParametersExtModelUni],
  kd4]]
Length[CoefficientList[Denominator[
   StationaryErrorEqn[[1]] /. ParametersExtModelUni], kd4]]
```

Out[366]=
3

Out[367]=
3

Out[368]=
3

Out[369]=
3

In[370]:=

```
(* 1 st Output: Order of numerator considering
   k5. 2 nd Output: Order of denominator considering k5.
     3 rd Output: Order of numerator considering kd5. 4 th
      Output: Order of denominator considering kd5. *)
Length[CoefficientList[Numerator[StationaryErrorEqn[[1]] /. ParametersExtModelUni],
  k5]]
Length[CoefficientList[Denominator[
   StationaryErrorEqn[[1]] /. ParametersExtModelUni], k5]]
Length[CoefficientList[Numerator[StationaryErrorEqn[[1]] /. ParametersExtModelUni],
  kd5]]
Length[CoefficientList[Denominator[
   StationaryErrorEqn[[1]] /. ParametersExtModelUni], kd5]]
```

Out[370]=
3

Out[371]=
5

Out[372]=
3

Out[373]=
5

3. General considerations

In the following we make some general considerations of a reaction cycle in which two independent processes are coupled (e.g. ligand binding and phosphorylation). Additionally, we assume that the reaction cycle is stimulated by influxes J1 to J4.

In[374]:=
```
(*Differential equantions for the micro-states D00[t],DL0[t],
 D0P[t] und DLP[t] in the general case (i.e.with in-or out-fluxes)*)
 ODEGeneral = {D00'[t] → -d1 - d2 + J1, DL0'[t] → d1 - d4 + J2,
   D0P'[t] → d2 - d3 + J3, DLP'[t] → d3 + d4 + J4};
ODEGeneralIndep = ODEGeneral /. ReactionRates1stExp /. Parameters1stExpIndep;

(*1 st Output:ODEs for the micro-states in this general case.*)
ODEGeneralIndep // MatrixForm

(*Formulation of the error equation,which can be written as a linear ODE g'[t]=
  -(k1+kd1+k2+kd2)*g[t]+u[t].2 nd Output:Stimulation term u[t].*)

ErrorEqn = DLP[t] * D00[t] - DL0[t] * D0P[t];
ODEforG[t] = FullSimplify[D[ErrorEqn, t] /. ODEGeneralIndep];
FullSimplify[ODEforG[t] + (k1 + kd1 + k2 + kd2) * (D00[t] * DLP[t] - D0P[t] * DL0[t])]
```

Out[376]//MatrixForm=

$$\begin{pmatrix} D00'[t] \to J1 - k1\,D00[t] - k2\,D00[t] + kd2\,D0P[t] + kd1\,DL0[t] \\ DL0'[t] \to J2 + k1\,D00[t] - k2\,DL0[t] - kd1\,DL0[t] + kd2\,DLP[t] \\ D0P'[t] \to J3 + k2\,D00[t] - k1\,D0P[t] - kd2\,D0P[t] + kd1\,DLP[t] \\ DLP'[t] \to J4 + k1\,D0P[t] + k2\,DL0[t] - kd1\,DLP[t] - kd2\,DLP[t] \end{pmatrix}$$

Out[379]=

$$J4\,D00[t] - J2\,D0P[t] - J3\,DL0[t] + J1\,DLP[t]$$

4. Transformation

In the following we show how the states of the detailed model can be calculated from the lumped states of the reduced model using the ratio equations. First, we will consider the simple example discussed in the text. Afterwards, we consider the more complicated example also described in the manuscript.

In[380]:=
```
(*Definition of reduced states as
  described in the main text for the simple example.*)
 Eqn1 = {R00 == D00, RL0 == DL0, R0P == D0P + D0E, RLP == DLP + DLE, RXE == D0E + DLE};
(*In order to calculate the 6 detailed states from the 5 reduced ones,
 one has to add an additional equation*)

Eqn2 = {D0P * DLE - D0E * DLP == 0};

(*1 st Output:Transformation of the reduced model states to the detailed ones*)

Transpose[FullSimplify[Solve[Join[Eqn1, Eqn2], {D00, D0P, DL0, DLP, D0E, DLE}]]] //
 MatrixForm
```

Out[382]//MatrixForm=

$$\begin{pmatrix} D00 \to R00 \\ DL0 \to RL0 \\ D0P \to R0P - \frac{R0P\,RXE}{R0P+RLP} \\ DLP \to RLP - \frac{RLP\,RXE}{R0P+RLP} \\ D0E \to \frac{R0P\,RXE}{R0P+RLP} \\ DLE \to \frac{RLP\,RXE}{R0P+RLP} \end{pmatrix}$$

In[383]:=

```
(*Definition of reduced states for the more complicated example.*)
Eqn1 = {ROOO == DOOO, ROPO == DOPO, RLOO == DLOO,
   RLPO == DLPO, rE == dE, rF == dF, ROOP == DOOP + DOOE + DOOEp + DOOF,
   ROPP == DOPP + DOPE + DOPEp + DOPF, RLOP == DLOP + DLOE + DLOEp + DLOF,
   RLPP == DLPP + DLPE + DLPEp + DLPF, RXE == DOOE + DOPE + DLOE + DLPE,
   RXEp == DOOEp + DOPEp + DLOEp + DLPEp + DOOF + DOPF + DLOF + DLPF,
   XEpF == DOOF + DOPF + DLOF + DLPF + dEpF, rEp == dEp + dEpF};

(*In this case one requires 10 ratio equations in order
  to reconstruct the detailed states from the reduced ones*)

Eqn2 = {DOOP / DOOE == DLOP / DLOE, DOOP / DOOE == DOPP / DOPE,
   DOOP / DOOE == DLPP / DLPE, DOOP / DOOEp == DLOP / DLOEp,
   DOOP / DOOEp == DOPP / DOPEp, DOOP / DOOEp == DLPP / DLPEp, DOOP / DOOF == DLOP / DLOF,
   DOOP / DOOF == DOPP / DOPF, DOOP / DOOF == DLPP / DLPF, DOOEp / DOOF == dEp / dEpF};

(*Calculation of the detailed model states*)

erg1 = Solve[Eqn2, {DOPEp, DLOEp, DOOEp, DOPE, DOOE, DOPP, DLOP, DLOE, DOOP, DLPF}];
erg2 = FullSimplify[Solve[Flatten[Eqn1 /. erg1],
    {dEpF, dEp, DLPE, DLPEp, DLPP, DOOF, DOPF, DLOF, DOOO, DOPO, DLOO, DLPO, dE, dF}]];

(*1 st Output:Transformation of the reduced model states to the detailed ones*)

FullSimplify[Join[Flatten[erg1 /. erg2], Flatten[erg2]]] // MatrixForm
```

Out[387]//MatrixForm=

$$
\begin{pmatrix}
\text{DOPEp} \to \frac{\text{ROPP RXEp (rEp+RXEp-XEpF)}}{\text{(RLOP+RLPP+ROOP+ROPP) (rEp+RXEp)}} \\
\text{DLOEp} \to \frac{\text{RLOP RXEp (rEp+RXEp-XEpF)}}{\text{(RLOP+RLPP+ROOP+ROPP) (rEp+RXEp)}} \\
\text{DOPE} \to \frac{\text{ROPP RXE}}{\text{RLOP+RLPP+ROOP+ROPP}} \\
\text{DLOE} \to \frac{\text{RLOP RXE}}{\text{RLOP+RLPP+ROOP+ROPP}} \\
\text{DLPF} \to \frac{\text{RLPP RXEp XEpF}}{\text{(RLOP+RLPP+ROOP+ROPP) (rEp+RXEp)}} \\
\text{DOPP} \to \frac{\text{ROPP (RLOP+RLPP+ROOP+ROPP-RXE-RXEp)}}{\text{RLOP+RLPP+ROOP+ROPP}} \\
\text{DLOP} \to \frac{\text{RLOP (RLOP+RLPP+ROOP+ROPP-RXE-RXEp)}}{\text{RLOP+RLPP+ROOP+ROPP}} \\
\text{DOOE} \to \frac{\text{ROOP RXE}}{\text{RLOP+RLPP+ROOP+ROPP}} \\
\text{DOOEp} \to \frac{\text{ROOP RXEp (rEp+RXEp-XEpF)}}{\text{(RLOP+RLPP+ROOP+ROPP) (rEp+RXEp)}} \\
\text{DOOP} \to \frac{\text{ROOP (RLOP+RLPP+ROOP+ROPP-RXE-RXEp)}}{\text{RLOP+RLPP+ROOP+ROPP}} \\
\text{DOOO} \to \text{ROOO} \\
\text{DOPO} \to \text{ROPO} \\
\text{DLOO} \to \text{RLOO} \\
\text{DLPO} \to \text{RLPO} \\
\text{dE} \to \text{rE} \\
\text{dF} \to \text{rF} \\
\text{DLPP} \to \frac{\text{RLPP (RLOP+RLPP+ROOP+ROPP-RXE-RXEp)}}{\text{RLOP+RLPP+ROOP+ROPP}} \\
\text{DLPE} \to \frac{\text{RLPP RXE}}{\text{RLOP+RLPP+ROOP+ROPP}} \\
\text{DOOF} \to \frac{\text{ROOP RXEp XEpF}}{\text{(RLOP+RLPP+ROOP+ROPP) (rEp+RXEp)}} \\
\text{DOPF} \to \frac{\text{ROPP RXEp XEpF}}{\text{(RLOP+RLPP+ROOP+ROPP) (rEp+RXEp)}} \\
\text{DLOF} \to \frac{\text{RLOP RXEp XEpF}}{\text{(RLOP+RLPP+ROOP+ROPP) (rEp+RXEp)}} \\
\text{DLPEp} \to \frac{\text{RLPP RXEp (rEp+RXEp-XEpF)}}{\text{(RLOP+RLPP+ROOP+ROPP) (rEp+RXEp)}} \\
\text{dEpF} \to \frac{\text{rEp XEpF}}{\text{rEp+RXEp}} \\
\text{dEp} \to \text{rEp} - \frac{\text{rEp XEpF}}{\text{rEp+RXEp}}
\end{pmatrix}
$$
