# Supplementary material for: Reduced modeling of signal transduction – a modular approach
Source: BMC Bioinformatics. 2007 Sep 13;8:336. doi: 10.1186/1471-2105-8-336 (PMC2216040; doi:10.1186/1471-2105-8-336)
Supplement: Additional file 3 — Layer based reduced modeling of insulin signaling [file 1471-2105-8-336-S3.pdf]

A large model of insulin signaling - step by step

In the following a model of insulin signaling as discussed in the manuscript is given. A very general and simple parameterization for reactions was chosen. The modeling procedure is performed according to the step by step protocol given in the manuscript. An executable model file is given in another additional file. In another additional file there is also demonstrated how the domain-oriented approach can be applied to each layer where reduction is possible. An executable model file where the layer based method and the domain-oriented approach are used is also given.

General considerations

The general format for a reaction is given as

$$A \leftrightharpoons B \quad k1 \quad k1d,$$

the left part specifies the reaction equation itself, the right part specifies the parameterization of the reaction rate. $k1$ is the kinetic parameter for the forward reaction, $k1d$ the kinetic parameter for the backward reaction. Using the law of mass action, the reaction rate then is given as

$$r_i = k1 * A - k1d * B.$$

Note that for dephosphorylation reactions of binding sites an additional factor $(x_i - x_i b)/x_i$ is needed to guarantee that only unoccupied binding sites are dephosphorylated. This factor is not included in the parameter part of the reaction and has to be considered when formulating the reaction rate.
Denotation of x_i (sum of phosphorylated binding sites), $x_i b$ (sum of occupied binding sites) and their difference, which is the sum of unoccupied phosphorylated binding sites is as follows. If a molecule A has only one binding site, the sum of phosphorylated binding sites is denoted as xA. If A has more than one binding site, the sum of phosphorylated binding sites on A for C is denoted as xAC. The corresponding sums of occupied binding sites follow the same notation, however followed by a lowercase 'b'. The denotation of the difference $x_i - x_i b$ (sum of unoccupied phosphorylated binding sites) starts with the name of the molecule that provides the binding sites, followed by 'Xp'. If this molecule binds more than one molecule, the name of the molecule that binds to this binding sites is appended. In this file, we denote species and their concentrations by the same symbol. In the executable Mathematica files, however, the concentration of a species is denoted by enclosing the species denotation by c["..."].

Step 1 and 2: Interactions and layers

The signaling process is divided into processes, interactions between processes are indicated by lines (step 1). All processes that are coupled by graded interactions are within the same layer (step 2). Layers are coupled by all-or-none interactions or do not interact. In Figure 1, processes are symbolized by black bordered boxes, all-or-none interactions by green lines, graded interactions by red lines. Layers are symbolized by blue boxes.

Step 3: Separate sub-models for each layer

As each layer can be modeled independently we start with the smaller layers (less reactions and ODEs) to get familiar with the procedure. The numbering of rates, however, is in the order the modeling process was performed, starting with the receptor layer.

SHP2 layer

The sole process in this layer is binding of SHP2 to its binding site on IRS. Free SHP2 is denoted as $SHP[0]$, SHP bound to IRS is denoted as $SHP[IRS]$

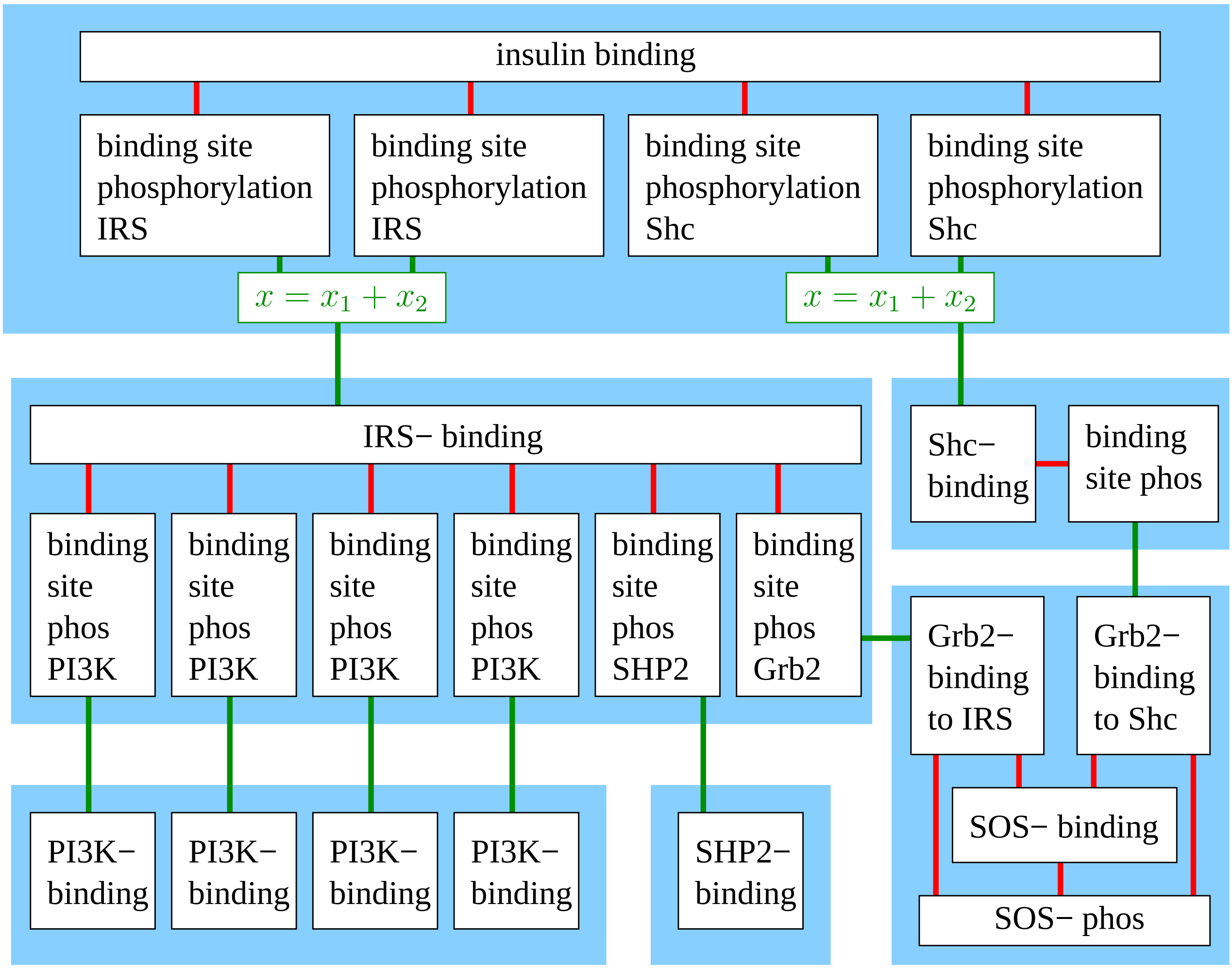

Figure 1: Interactions and layers in insulin signaling
Graded interactions between processes are indicated by red lines, all-or-none interactions by green lines. The boxes $x = x_1 + x_2$ indicate summation of sums of concentrations of phosphorylated binding sites. The modification factors $(x_i - x_ib)/x_i$ for dephosphorylation rates are computed using the sums $x_1 + x_2$ as x_i.

a) Define all sums of phosphorylated binding sites x_i and occupied binding sites x_ib

SHP2 has no binding site that becomes phosphorylated, therefore no x_i is to be defined. The sum of occupied binding sites on IRS by SHP is

$$xIrsShp2b = SHP2[IRS]$$

b) Define the concentrations of all unoccupied phosphorylated binding sites that are needed as binding partners within the considered layer

$$IrsXpShp2 = xIrsShp2 - xIrsShp2b$$

$xIrsShp2$ is the sum of phosphorylated binding sites for SHP2 on IRS, $IrsXpShp2$ is the sum of unoccupied phosphorylated binding sites for SHP2 on IRS.

c) Define rules and reactions (including dephosphorylation of binding sites) as if there were no other layers

$$IrsXpShp2 + SHP2[0] \leftrightharpoons SHP2[IRS] \quad kbshp2 \quad kbshp2d$$

d) translate each reaction into the corresponding rate

$$r664 = kbshp2 * IrsXpShp2 * SHP2[0] - kbshp2d * SHP2[IRS]$$

e) construct ODEs

$$\frac{d}{dt}SHP2[0] = -r664$$
$$\frac{d}{dt}SHP2[IRS] = r664$$

Shc layer

Processes of the Shc layer are Shc binding to the insulin receptor and binding site phosphorylation.

a) Define all sums of phosphorylated binding sites x_i and occupied binding sites x_ib

$$xRShcb = Shc[R, X]$$
$$xShc = Shc[X, P]$$

$xRShcb$ is the sum of occupied Shc binding sites on the receptor, $xShc$ is the sum of phosphorylated binding sites on Shc for Grb2.

b) Define the concentrations of all unoccupied phosphorylated binding sites that are needed as binding partners within the considered layer

$$RXpShc = xRShc - xRShcb$$

$RXpShc$ is the sum of unoccupied phosphorylated binding sites for Shc on the receptor.

c) Define rules and reactions (including dephosphorylation of binding sites) as if there were no other layers

Shc binding to the receptor

$$RXpShc + Shc[0, 0] \leftrightharpoons Shc[R, 0] \quad kbshc \quad kbshcd$$
$$RXpShc + Shc[0, P] \leftrightharpoons Shc[R, P] \quad kbshc \quad kbshcd$$

Shc phosphorylation

$$Shc[0, 0] \leftrightharpoons Shc[0, P] \quad kshcp \quad kshcpd$$
$$Shc[R, 0] \leftrightharpoons Shc[R, P] \quad kshcbp \quad kshcbpd$$

d) translate each reaction into the corresponding rate

$$r640 = kbshc * RXpShc * Shc[0, 0] - kbshcd * Shc[R, 0]$$
$$r641 = kbshc * RXpShc * Shc[0, P] - kbshcd * Shc[R, P]$$
$$r642 = kshcp * Shc[0, 0] - kshcpd * ((xShc - xShcb)/xShc) * Shc[0, P]$$
$$r643 = kshcbp * Shc[R, 0] - kshcbpd * ((xShc - xShcb)/xShc) * Shc[R, P]$$

In rates $r642$ and $r643$ the factor $(xShc - xShcb)/xShc$ is necessary, as only the fraction of $Shc[X, P]$ with unoccupied binding site is subject to dephosphorylation.

e) construct ODEs

$$\frac{d}{dt}Shc[0, 0] = -r640 - r642$$
$$\frac{d}{dt}Shc[0, P] = -r641 + r642$$
$$\frac{d}{dt}Shc[R, 0] = r640 - r643$$
$$\frac{d}{dt}Shc[R, P] = r641 + r643$$

Grb2/SOS layer

The reactions of Grb2 and SOS are within the same layer, as SOS binding to Grb2 and SOS phosphorylation influence Grb2 binding to Shc, which are graded interactions (Figure 1). Processes within this layer are binding of Grb2 to IRS and Shc, SOS binding to Grb2 and SOS phosphorylation.

a) Define all sums of phosphorylated binding sites x_i and occupied binding sites x_ib

$$xShcb = Grb2[Shc, X]$$
$$xIrsGrb2b = Grb2[IRS, X]$$

xShcb is the sum of occupied binding sites on Shc, xIrsGrb2b is the sum of occupied binding sites on IRS.

b) Define the concentrations of all unoccupied phosphorylated binding sites that are needed as binding partners within the considered layer

$$ShcXp = xShc - xShcb$$
$$IrsXpGrb2 = xIrsGrb2 - xIrsGrb2b$$

ShcXp is the sum of unoccupied phosphorylated binding sites for Grb2 on Shc, IrsXpGrb2 is the sum of unoccupied phosphorylated binding sites for Grb2 on IRS.

c) Define rules and reactions (including dephosphorylation of binding sites) as if there were no other layers

binding of Grb2 to IRS

$$IrsXpGrb2 + Grb2[0, 0] \leftrightharpoons Grb2[IRS, 0] \quad kbirsgrb2 \quad kbirsgrb2d$$
$$IrsXpGrb2 + Grb2[0, SOS] \leftrightharpoons Grb2[IRS, SOS] \quad kbirsgrb2sos \quad kbirsgrb2sosd$$
$$IrsXpGrb2 + Grb2[0, SOSp] \leftrightharpoons Grb2[IRS, SOSp] \quad kbirsgrb2sosp \quad kbirsgrb2sospd$$

binding of SOS to Grb2

$$Grb2[0, 0] + SOS[0] \leftrightharpoons Grb2[0, SOS] \quad kbsos \quad kbsosd$$
$$Grb2[IRS, 0] + SOS[0] \leftrightharpoons Grb2[IRS, SOS] \quad kbsos \quad kbsosd$$
$$Grb2[Shc, 0] + SOS[0] \leftrightharpoons Grb2[Shc, SOS] \quad kbsos \quad kbsosd$$
$$Grb2[0, 0] + SOS[p] \leftrightharpoons Grb2[0, SOSp] \quad kbsosp \quad kbsosdp$$
$$Grb2[IRS, 0] + SOS[p] \leftrightharpoons Grb2[IRS, SOSp] \quad kbsosp \quad kbsosdp$$
$$Grb2[Shc, 0] + SOS[p] \leftrightharpoons Grb2[Shc, SOSp] \quad kbsosp \quad kbsosdp$$

binding of Grb2 to Shc

$$ShcXp + Grb2[0, 0] \leftrightharpoons Grb2[Shc, 0] \quad kbshcgrb2 \quad kbshcgrb2d$$
$$ShcXp + Grb2[0, SOS] \leftrightharpoons Grb2[Shc, SOS] \quad kbshcgrb2sos \quad kbshcgrb2sosd$$
$$ShcXp + Grb2[0, SOSp] \leftrightharpoons Grb2[Shc, SOSp] \quad kbshcgrbsosp2 \quad kbshcgrb2sospd$$

SOS phosphorylation

$$Grb2[0, SOS] \leftrightharpoons Grb2[0, SOSp] \quad kpgrb2sos \quad kpgrb2sosd$$
$$Grb2[IRS, SOS] \leftrightharpoons Grb2[IRS, SOSp] \quad kpirsgrb2sos \quad pirsgrb2sosd$$
$$Grb2[Shc, SOS] \leftrightharpoons Grb2[Shc, SOSp] \quad kpshcgrb2sos \quad kpshcgrb2sosd$$
$$SOS[0] \leftrightharpoons SOS[p] \quad kpsos \quad kpsosd$$

d) translate each reaction into the corresponding rate

$$r644 = kbirsgrb2 * IrsXpGrb2 * Grb2[0,0] - kbirsgrb2d * Grb2[IRS,0]$$
$$r645 = kbirsgrb2sos * IrsXpGrb2 * Grb2[0,SOS] - kbirsgrb2sosd * Grb2[IRS,SOS]$$
$$r646 = kbirsgrb2sosp * IrsXpGrb2 * Grb2[0,SOSp] - kbirsgrb2sospd * Grb2[IRS,SOSp]$$
$$r647 = kbsos * Grb2[0,0] * SOS[0] - kbsosd * Grb2[0,SOS]$$
$$r648 = kbsos * Grb2[IRS,0] * SOS[0] - kbsosd * Grb2[IRS,SOS]$$
$$r649 = kbsos * Grb2[Shc,0] * SOS[0] - kbsosd * Grb2[Shc,SOS]$$
$$r650 = kbsosp * Grb2[0,0] * SOS[p] - kbsosdp * Grb2[0,SOSp]$$
$$r651 = kbsosp * Grb2[IRS,0] * SOS[p] - kbsosdp * Grb2[IRS,SOSp]$$
$$r652 = kbsosp * Grb2[Shc,0] * SOS[p] - kbsosdp * Grb2[Shc,SOSp]$$
$$r653 = kbshcgrb2 * ShcXp * Grb2[0,0] - kbshcgrb2d * Grb2[Shc,0]$$
$$r654 = kbshcgrb2sos * ShcXp * Grb2[0,SOS] - kbshcgrb2sosd * Grb2[Shc,SOS]$$
$$r655 = kbshcgrbsosp2 * ShcXp * Grb2[0,SOSp] - kbshcgrb2sospd * Grb2[Shc,SOSp]$$
$$r656 = kpgrb2sos * Grb2[0,SOS] - kpgrb2sosd * Grb2[0,SOSp]$$
$$r657 = kpirsgrb2sos * Grb2[IRS,SOS] - pirsgrb2sosd * Grb2[IRS,SOSp]$$
$$r658 = kpshcgrb2sos * Grb2[Shc,SOS] - kpshcgrb2sosd * Grb2[Shc,SOSp]$$
$$r659 = kpsos * SOS[0] - kpsosd * SOS[p]$$

e) construct ODEs

$$\frac{d}{dt}Grb2[0,0] = -r644 - r647 - r650 - r653$$
$$\frac{d}{dt}Grb2[0,SOS] = -r645 + r647 - r654 - r656$$
$$\frac{d}{dt}Grb2[0,SOSp] = -r646 + r650 - r655 + r656$$
$$\frac{d}{dt}Grb2[IRS,0] = r644 - r648 - r651$$
$$\frac{d}{dt}Grb2[IRS,SOS] = r645 + r648 - r657$$
$$\frac{d}{dt}Grb2[IRS,SOSp] = r646 + r651 + r657$$
$$\frac{d}{dt}Grb2[Shc,0] = -r649 - r652 + r653$$
$$\frac{d}{dt}Grb2[Shc,SOS] = r649 + r654 - r658$$
$$\frac{d}{dt}Grb2[Shc,SOSp] = r652 + r655 + r658$$
$$\frac{d}{dt}SOS[0] = -r647 - r648 - r649 - r659$$
$$\frac{d}{dt}SOS[p] = -r650 - r651 - r652 + r659$$

PI3K layer

Within the PI3K layer there are four binding processes, one for each binding site on IRS.

a) Define all sums of phosphorylated binding sites x_i and occupied binding sites x_ib

$$xIrsPi3k1b = PI3K[IRS1]$$
$$xIrsPi3k2b = PI3K[IRS2]$$
$$xIrsPi3k3b = PI3K[IRS3]$$
$$xIrsPi3k4b = PI3K[IRS4]$$

$xIrsPi3k_ib$ is the sum of occupied binding sites on the binding site of IRS that is indexed by i.

b) Define the concentrations of all unoccupied phosphorylated binding sites that are needed as binding partners within the considered layer

$$IrsXpPi3k1 = xIrsPi3k1 - xIrsPi3k1b$$
$$IrsXpPi3k2 = xIrsPi3k2 - xIrsPi3k2b$$
$$IrsXpPi3k3 = xIrsPi3k3 - xIrsPi3k3b$$
$$IrsXpPi3k4 = xIrsPi3k4 - xIrsPi3k4b$$

$IrsXpPi3k_i$ is the sum of unoccupied phosphorylated binding sites for PI3K on the binding site of IRS that is indexed by i.

c) Define rules and reactions (including dephosphorylation of binding sites) as if there were no other layers

PI3K binding to four binding sites on IRS

$$IrsXpPi3k1 + PI3K[0] \leftrightharpoons PI3K[IRS1] \quad kbpi3k1 \quad kbpi3k1d$$
$$IrsXpPi3k2 + PI3K[0] \leftrightharpoons PI3K[IRS2] \quad kbpi3k2 \quad kbpi3k2d$$
$$IrsXpPi3k3 + PI3K[0] \leftrightharpoons PI3K[IRS3] \quad kbpi3k3 \quad kbpi3k3d$$
$$IrsXpPi3k4 + PI3K[0] \leftrightharpoons PI3K[IRS4] \quad kbpi3k4 \quad kbpi3k4d$$

d) translate each reaction into the corresponding rate

$$r660 = kbpi3k1 * IrsXpPi3k1 * PI3K[0] - kbpi3k1d * PI3K[IRS1]$$
$$r661 = kbpi3k2 * IrsXpPi3k2 * PI3K[0] - kbpi3k2d * PI3K[IRS2]$$
$$r662 = kbpi3k3 * IrsXpPi3k3 * PI3K[0] - kbpi3k3d * PI3K[IRS3]$$
$$r663 = kbpi3k4 * IrsXpPi3k4 * PI3K[0] - kbpi3k4d * PI3K[IRS4]$$

e) construct ODEs

$$\frac{d}{dt}PI3K[0] = -r660 - r661 - r662 - r663$$
$$\frac{d}{dt}PI3K[IRS1] = r660$$
$$\frac{d}{dt}PI3K[IRS2] = r661$$
$$\frac{d}{dt}PI3K[IRS3] = r662$$
$$\frac{d}{dt}PI3K[IRS4] = r663$$

receptor layer

Processes in the receptor layer are binding of insulin and phosphorylation of two binding sites for IRS and Shc each. The receptor has six sites. The first two sites are binding sites for insulin, they can be unoccupied (0) or occupied by insulin (I). The next two sites are binding sites for IRS, the last two sites are binding sites for Shc. All binding sites for IRS and Shc can be phosphorylated (P) or unphosphorylated (0). Insulin concentration is assumed to be constant.

$$Ins = 100$$

a) Define all sums of phosphorylated binding sites x_i and occupied binding sites x_ib

$$xRIrs = R[X, X, P, X, X, X] + R[X, X, X, P, X, X]$$
$$xRShc = R[X, X, X, X, P, X] + R[X, X, X, X, X, P]$$

$xRIrs$ is the sum of phosphorylated binding sites for IRS, $xRShc$ is the sum of phosphorylated binding sites for Shc. As no x_i is passed to the receptor layer, there is no x_ib in this layer.

b) Define the concentrations of all unoccupied phosphorylated binding sites that are needed as binding partners within the considered layer

As there is no binding process that is described in this way, this is not necessary here.

c) Define rules and reactions (including dephosphorylation of binding sites) as if there were no other layers

insulin binding

$$R[0, 0, X, X, X, X] + Ins \leftrightharpoons R[I, 0, X, X, X, X] \quad ki1 \quad ki1d$$
$$R[0, 0, X, X, X, X] + Ins \leftrightharpoons R[0, I, X, X, X, X] \quad ki1 \quad ki1d$$
$$R[I, 0, X, X, X, X] + Ins \leftrightharpoons R[I, I, X, X, X, X] \quad ki2 \quad ki2d$$
$$R[0, I, X, X, X, X] + Ins \leftrightharpoons R[I, I, X, X, X, X] \quad ki2 \quad ki2d$$

phosphorylation of first IRS binding site

$$R[0, 0, 0, X, X, X] \leftrightharpoons R[0, 0, P, X, X, X] \quad kp10 \quad kp10d$$
$$R[I, 0, 0, X, X, X] \leftrightharpoons R[I, 0, P, X, X, X] \quad kp11 \quad kp11d$$
$$R[0, I, 0, X, X, X] \leftrightharpoons R[0, I, P, X, X, X] \quad kp11 \quad kp11d$$
$$R[I, I, 0, X, X, X] \leftrightharpoons R[I, I, P, X, X, X] \quad kp12 \quad kp12d$$

phosphorylation of second IRS binding site

$$R[0, 0, X, 0, X, X] \leftrightharpoons R[0, 0, X, P, X, X] \quad kp20 \quad kp20d$$
$$R[I, 0, X, 0, X, X] \leftrightharpoons R[I, 0, X, P, X, X] \quad kp21 \quad kp21d$$
$$R[0, I, X, 0, X, X] \leftrightharpoons R[0, I, X, P, X, X] \quad kp21 \quad kp21d$$
$$R[I, I, X, 0, X, X] \leftrightharpoons R[I, I, X, P, X, X] \quad kp22 \quad kp22d$$

phosphorylation of first Shc binding site

$$R[0, 0, X, X, 0, X] \leftrightharpoons R[0, 0, X, X, P, X] \quad kp30 \quad kp30d$$
$$R[I, 0, X, X, 0, X] \leftrightharpoons R[I, 0, X, X, P, X] \quad kp31 \quad kp31d$$
$$R[0, I, X, X, 0, X] \leftrightharpoons R[0, I, X, X, P, X] \quad kp31 \quad kp31d$$
$$R[I, I, X, X, 0, X] \leftrightharpoons R[I, I, X, X, P, X] \quad kp32 \quad kp32d$$

phosphorylation of second Shc binding site

$$R[0, 0, X, X, X, 0] \leftrightharpoons R[0, 0, X, X, X, P] \quad kp40 \quad kp40d$$
$$R[I, 0, X, X, X, 0] \leftrightharpoons R[I, 0, X, X, X, P] \quad kp41 \quad kp41d$$
$$R[0, I, X, X, X, 0] \leftrightharpoons R[0, I, X, X, X, P] \quad kp41 \quad kp41d$$
$$R[I, I, X, X, X, 0] \leftrightharpoons R[I, I, X, X, X, P] \quad kp42 \quad kp42d$$

these rules correspond to the following reactions

$$R[0,0,0,0,0,0] + Ins \leftrightharpoons R[I,0,0,0,0,0] \quad ki1 \quad ki1d$$
$$R[0,0,0,0,0,P] + Ins \leftrightharpoons R[I,0,0,0,0,P] \quad ki1 \quad ki1d$$
$$R[0,0,0,0,P,0] + Ins \leftrightharpoons R[I,0,0,0,P,0] \quad ki1 \quad ki1d$$
$$R[0,0,0,0,P,P] + Ins \leftrightharpoons R[I,0,0,0,P,P] \quad ki1 \quad ki1d$$
$$R[0,0,0,P,0,0] + Ins \leftrightharpoons R[I,0,0,P,0,0] \quad ki1 \quad ki1d$$
$$R[0,0,0,P,0,P] + Ins \leftrightharpoons R[I,0,0,P,0,P] \quad ki1 \quad ki1d$$
$$R[0,0,0,P,P,0] + Ins \leftrightharpoons R[I,0,0,P,P,0] \quad ki1 \quad ki1d$$
$$R[0,0,0,P,P,P] + Ins \leftrightharpoons R[I,0,0,P,P,P] \quad ki1 \quad ki1d$$
$$R[0,0,P,0,0,0] + Ins \leftrightharpoons R[I,0,P,0,0,0] \quad ki1 \quad ki1d$$
$$R[0,0,P,0,0,P] + Ins \leftrightharpoons R[I,0,P,0,0,P] \quad ki1 \quad ki1d$$
$$R[0,0,P,0,P,0] + Ins \leftrightharpoons R[I,0,P,0,P,0] \quad ki1 \quad ki1d$$
$$R[0,0,P,0,P,P] + Ins \leftrightharpoons R[I,0,P,0,P,P] \quad ki1 \quad ki1d$$
$$R[0,0,P,P,0,0] + Ins \leftrightharpoons R[I,0,P,P,0,0] \quad ki1 \quad ki1d$$
$$R[0,0,P,P,0,P] + Ins \leftrightharpoons R[I,0,P,P,0,P] \quad ki1 \quad ki1d$$
$$R[0,0,P,P,P,0] + Ins \leftrightharpoons R[I,0,P,P,P,0] \quad ki1 \quad ki1d$$
$$R[0,0,P,P,P,P] + Ins \leftrightharpoons R[I,0,P,P,P,P] \quad ki1 \quad ki1d$$
$$R[0,0,0,0,0,0] + Ins \leftrightharpoons R[0,I,0,0,0,0] \quad ki1 \quad ki1d$$
$$R[0,0,0,0,0,P] + Ins \leftrightharpoons R[0,I,0,0,0,P] \quad ki1 \quad ki1d$$
$$R[0,0,0,0,P,0] + Ins \leftrightharpoons R[0,I,0,0,P,0] \quad ki1 \quad ki1d$$
$$R[0,0,0,0,P,P] + Ins \leftrightharpoons R[0,I,0,0,P,P] \quad ki1 \quad ki1d$$
$$R[0,0,0,P,0,0] + Ins \leftrightharpoons R[0,I,0,P,0,0] \quad ki1 \quad ki1d$$
$$R[0,0,0,P,0,P] + Ins \leftrightharpoons R[0,I,0,P,0,P] \quad ki1 \quad ki1d$$
$$R[0,0,0,P,P,0] + Ins \leftrightharpoons R[0,I,0,P,P,0] \quad ki1 \quad ki1d$$
$$R[0,0,0,P,P,P] + Ins \leftrightharpoons R[0,I,0,P,P,P] \quad ki1 \quad ki1d$$
$$R[0,0,P,0,0,0] + Ins \leftrightharpoons R[0,I,P,0,0,0] \quad ki1 \quad ki1d$$
$$R[0,0,P,0,0,P] + Ins \leftrightharpoons R[0,I,P,0,0,P] \quad ki1 \quad ki1d$$
$$R[0,0,P,0,P,0] + Ins \leftrightharpoons R[0,I,P,0,P,0] \quad ki1 \quad ki1d$$
$$R[0,0,P,0,P,P] + Ins \leftrightharpoons R[0,I,P,0,P,P] \quad ki1 \quad ki1d$$
$$R[0,0,P,P,0,0] + Ins \leftrightharpoons R[0,I,P,P,0,0] \quad ki1 \quad ki1d$$
$$R[0,0,P,P,0,P] + Ins \leftrightharpoons R[0,I,P,P,0,P] \quad ki1 \quad ki1d$$
$$R[0,0,P,P,P,0] + Ins \leftrightharpoons R[0,I,P,P,P,0] \quad ki1 \quad ki1d$$
$$R[0,0,P,P,P,P] + Ins \leftrightharpoons R[0,I,P,P,P,P] \quad ki1 \quad ki1d$$
$$R[I,0,0,0,0,0] + Ins \leftrightharpoons R[I,I,0,0,0,0] \quad ki2 \quad ki2d$$
$$R[I,0,0,0,0,P] + Ins \leftrightharpoons R[I,I,0,0,0,P] \quad ki2 \quad ki2d$$
$$R[I,0,0,0,P,0] + Ins \leftrightharpoons R[I,I,0,0,P,0] \quad ki2 \quad ki2d$$
$$R[I,0,0,0,P,P] + Ins \leftrightharpoons R[I,I,0,0,P,P] \quad ki2 \quad ki2d$$
$$R[I,0,0,P,0,0] + Ins \leftrightharpoons R[I,I,0,P,0,0] \quad ki2 \quad ki2d$$
$$R[I,0,0,P,0,P] + Ins \leftrightharpoons R[I,I,0,P,0,P] \quad ki2 \quad ki2d$$
$$R[I,0,0,P,P,0] + Ins \leftrightharpoons R[I,I,0,P,P,0] \quad ki2 \quad ki2d$$
$$R[I,0,0,P,P,P] + Ins \leftrightharpoons R[I,I,0,P,P,P] \quad ki2 \quad ki2d$$
$$R[I,0,P,0,0,0] + Ins \leftrightharpoons R[I,I,P,0,0,0] \quad ki2 \quad ki2d$$
$$R[I,0,P,0,0,P] + Ins \leftrightharpoons R[I,I,P,0,0,P] \quad ki2 \quad ki2d$$
$$R[I,0,P,0,P,0] + Ins \leftrightharpoons R[I,I,P,0,P,0] \quad ki2 \quad ki2d$$
$$R[I,0,P,0,P,P] + Ins \leftrightharpoons R[I,I,P,0,P,P] \quad ki2 \quad ki2d$$
$$R[I,0,P,P,0,0] + Ins \leftrightharpoons R[I,I,P,P,0,0] \quad ki2 \quad ki2d$$

$$R[I,0,P,P,0,P] + Ins \leftrightharpoons R[I,I,P,P,0,P] \quad ki2 \quad ki2d$$
$$R[I,0,P,P,P,0] + Ins \leftrightharpoons R[I,I,P,P,P,0] \quad ki2 \quad ki2d$$
$$R[I,0,P,P,P,P] + Ins \leftrightharpoons R[I,I,P,P,P,P] \quad ki2 \quad ki2d$$
$$R[0,I,0,0,0,0] + Ins \leftrightharpoons R[I,I,0,0,0,0] \quad ki2 \quad ki2d$$
$$R[0,I,0,0,0,P] + Ins \leftrightharpoons R[I,I,0,0,0,P] \quad ki2 \quad ki2d$$
$$R[0,I,0,0,P,0] + Ins \leftrightharpoons R[I,I,0,0,P,0] \quad ki2 \quad ki2d$$
$$R[0,I,0,0,P,P] + Ins \leftrightharpoons R[I,I,0,0,P,P] \quad ki2 \quad ki2d$$
$$R[0,I,0,P,0,0] + Ins \leftrightharpoons R[I,I,0,P,0,0] \quad ki2 \quad ki2d$$
$$R[0,I,0,P,0,P] + Ins \leftrightharpoons R[I,I,0,P,0,P] \quad ki2 \quad ki2d$$
$$R[0,I,0,P,P,0] + Ins \leftrightharpoons R[I,I,0,P,P,0] \quad ki2 \quad ki2d$$
$$R[0,I,0,P,P,P] + Ins \leftrightharpoons R[I,I,0,P,P,P] \quad ki2 \quad ki2d$$
$$R[0,I,P,0,0,0] + Ins \leftrightharpoons R[I,I,P,0,0,0] \quad ki2 \quad ki2d$$
$$R[0,I,P,0,0,P] + Ins \leftrightharpoons R[I,I,P,0,0,P] \quad ki2 \quad ki2d$$
$$R[0,I,P,0,P,0] + Ins \leftrightharpoons R[I,I,P,0,P,0] \quad ki2 \quad ki2d$$
$$R[0,I,P,0,P,P] + Ins \leftrightharpoons R[I,I,P,0,P,P] \quad ki2 \quad ki2d$$
$$R[0,I,P,P,0,0] + Ins \leftrightharpoons R[I,I,P,P,0,0] \quad ki2 \quad ki2d$$
$$R[0,I,P,P,0,P] + Ins \leftrightharpoons R[I,I,P,P,0,P] \quad ki2 \quad ki2d$$
$$R[0,I,P,P,P,0] + Ins \leftrightharpoons R[I,I,P,P,P,0] \quad ki2 \quad ki2d$$
$$R[0,I,P,P,P,P] + Ins \leftrightharpoons R[I,I,P,P,P,P] \quad ki2 \quad ki2d$$
$$R[0,0,0,0,0,0] \leftrightharpoons R[0,0,P,0,0,0] \quad kp10 \quad kp10d$$
$$R[0,0,0,0,0,P] \leftrightharpoons R[0,0,P,0,0,P] \quad kp10 \quad kp10d$$
$$R[0,0,0,0,P,0] \leftrightharpoons R[0,0,P,0,P,0] \quad kp10 \quad kp10d$$
$$R[0,0,0,0,P,P] \leftrightharpoons R[0,0,P,0,P,P] \quad kp10 \quad kp10d$$
$$R[0,0,0,P,0,0] \leftrightharpoons R[0,0,P,P,0,0] \quad kp10 \quad kp10d$$
$$R[0,0,0,P,0,P] \leftrightharpoons R[0,0,P,P,0,P] \quad kp10 \quad kp10d$$
$$R[0,0,0,P,P,0] \leftrightharpoons R[0,0,P,P,P,0] \quad kp10 \quad kp10d$$
$$R[0,0,0,P,P,P] \leftrightharpoons R[0,0,P,P,P,P] \quad kp10 \quad kp10d$$
$$R[I,0,0,0,0,0] \leftrightharpoons R[I,0,P,0,0,0] \quad kp11 \quad kp11d$$
$$R[I,0,0,0,0,P] \leftrightharpoons R[I,0,P,0,0,P] \quad kp11 \quad kp11d$$
$$R[I,0,0,0,P,0] \leftrightharpoons R[I,0,P,0,P,0] \quad kp11 \quad kp11d$$
$$R[I,0,0,0,P,P] \leftrightharpoons R[I,0,P,0,P,P] \quad kp11 \quad kp11d$$
$$R[I,0,0,P,0,0] \leftrightharpoons R[I,0,P,P,0,0] \quad kp11 \quad kp11d$$
$$R[I,0,0,P,0,P] \leftrightharpoons R[I,0,P,P,0,P] \quad kp11 \quad kp11d$$
$$R[I,0,0,P,P,0] \leftrightharpoons R[I,0,P,P,P,0] \quad kp11 \quad kp11d$$
$$R[I,0,0,P,P,P] \leftrightharpoons R[I,0,P,P,P,P] \quad kp11 \quad kp11d$$
$$R[0,I,0,0,0,0] \leftrightharpoons R[0,I,P,0,0,0] \quad kp11 \quad kp11d$$
$$R[0,I,0,0,0,P] \leftrightharpoons R[0,I,P,0,0,P] \quad kp11 \quad kp11d$$
$$R[0,I,0,0,P,0] \leftrightharpoons R[0,I,P,0,P,0] \quad kp11 \quad kp11d$$
$$R[0,I,0,0,P,P] \leftrightharpoons R[0,I,P,0,P,P] \quad kp11 \quad kp11d$$
$$R[0,I,0,P,0,0] \leftrightharpoons R[0,I,P,P,0,0] \quad kp11 \quad kp11d$$
$$R[0,I,0,P,0,P] \leftrightharpoons R[0,I,P,P,0,P] \quad kp11 \quad kp11d$$
$$R[0,I,0,P,P,0] \leftrightharpoons R[0,I,P,P,P,0] \quad kp11 \quad kp11d$$
$$R[0,I,0,P,P,P] \leftrightharpoons R[0,I,P,P,P,P] \quad kp11 \quad kp11d$$
$$R[I,I,0,0,0,0] \leftrightharpoons R[I,I,P,0,0,0] \quad kp12 \quad kp12d$$
$$R[I,I,0,0,0,P] \leftrightharpoons R[I,I,P,0,0,P] \quad kp12 \quad kp12d$$

$$R[I,I,0,0,P,0] \leftrightharpoons R[I,I,P,0,P,0] \quad kp12 \quad kp12d$$
$$R[I,I,0,0,P,P] \leftrightharpoons R[I,I,P,0,P,P] \quad kp12 \quad kp12d$$
$$R[I,I,0,P,0,0] \leftrightharpoons R[I,I,P,P,0,0] \quad kp12 \quad kp12d$$
$$R[I,I,0,P,0,P] \leftrightharpoons R[I,I,P,P,0,P] \quad kp12 \quad kp12d$$
$$R[I,I,0,P,P,0] \leftrightharpoons R[I,I,P,P,P,0] \quad kp12 \quad kp12d$$
$$R[I,I,0,P,P,P] \leftrightharpoons R[I,I,P,P,P,P] \quad kp12 \quad kp12d$$
$$R[0,0,0,0,0,0] \leftrightharpoons R[0,0,0,P,0,0] \quad kp20 \quad kp20d$$
$$R[0,0,0,0,0,P] \leftrightharpoons R[0,0,0,P,0,P] \quad kp20 \quad kp20d$$
$$R[0,0,0,0,P,0] \leftrightharpoons R[0,0,0,P,P,0] \quad kp20 \quad kp20d$$
$$R[0,0,0,0,P,P] \leftrightharpoons R[0,0,0,P,P,P] \quad kp20 \quad kp20d$$
$$R[0,0,P,0,0,0] \leftrightharpoons R[0,0,P,P,0,0] \quad kp20 \quad kp20d$$
$$R[0,0,P,0,0,P] \leftrightharpoons R[0,0,P,P,0,P] \quad kp20 \quad kp20d$$
$$R[0,0,P,0,P,0] \leftrightharpoons R[0,0,P,P,P,0] \quad kp20 \quad kp20d$$
$$R[0,0,P,0,P,P] \leftrightharpoons R[0,0,P,P,P,P] \quad kp20 \quad kp20d$$
$$R[I,0,0,0,0,0] \leftrightharpoons R[I,0,0,P,0,0] \quad kp21 \quad kp21d$$
$$R[I,0,0,0,0,P] \leftrightharpoons R[I,0,0,P,0,P] \quad kp21 \quad kp21d$$
$$R[I,0,0,0,P,0] \leftrightharpoons R[I,0,0,P,P,0] \quad kp21 \quad kp21d$$
$$R[I,0,0,0,P,P] \leftrightharpoons R[I,0,0,P,P,P] \quad kp21 \quad kp21d$$
$$R[I,0,P,0,0,0] \leftrightharpoons R[I,0,P,P,0,0] \quad kp21 \quad kp21d$$
$$R[I,0,P,0,0,P] \leftrightharpoons R[I,0,P,P,0,P] \quad kp21 \quad kp21d$$
$$R[I,0,P,0,P,0] \leftrightharpoons R[I,0,P,P,P,0] \quad kp21 \quad kp21d$$
$$R[I,0,P,0,P,P] \leftrightharpoons R[I,0,P,P,P,P] \quad kp21 \quad kp21d$$
$$R[0,I,0,0,0,0] \leftrightharpoons R[0,I,0,P,0,0] \quad kp21 \quad kp21d$$
$$R[0,I,0,0,0,P] \leftrightharpoons R[0,I,0,P,0,P] \quad kp21 \quad kp21d$$
$$R[0,I,0,0,P,0] \leftrightharpoons R[0,I,0,P,P,0] \quad kp21 \quad kp21d$$
$$R[0,I,0,0,P,P] \leftrightharpoons R[0,I,0,P,P,P] \quad kp21 \quad kp21d$$
$$R[0,I,P,0,0,0] \leftrightharpoons R[0,I,P,P,0,0] \quad kp21 \quad kp21d$$
$$R[0,I,P,0,0,P] \leftrightharpoons R[0,I,P,P,0,P] \quad kp21 \quad kp21d$$
$$R[0,I,P,0,P,0] \leftrightharpoons R[0,I,P,P,P,0] \quad kp21 \quad kp21d$$
$$R[0,I,P,0,P,P] \leftrightharpoons R[0,I,P,P,P,P] \quad kp21 \quad kp21d$$
$$R[I,I,0,0,0,0] \leftrightharpoons R[I,I,0,P,0,0] \quad kp22 \quad kp22d$$
$$R[I,I,0,0,0,P] \leftrightharpoons R[I,I,0,P,0,P] \quad kp22 \quad kp22d$$
$$R[I,I,0,0,P,0] \leftrightharpoons R[I,I,0,P,P,0] \quad kp22 \quad kp22d$$
$$R[I,I,0,0,P,P] \leftrightharpoons R[I,I,0,P,P,P] \quad kp22 \quad kp22d$$
$$R[I,I,P,0,0,0] \leftrightharpoons R[I,I,P,P,0,0] \quad kp22 \quad kp22d$$
$$R[I,I,P,0,0,P] \leftrightharpoons R[I,I,P,P,0,P] \quad kp22 \quad kp22d$$
$$R[I,I,P,0,P,0] \leftrightharpoons R[I,I,P,P,P,0] \quad kp22 \quad kp22d$$
$$R[I,I,P,0,P,P] \leftrightharpoons R[I,I,P,P,P,P] \quad kp22 \quad kp22d$$
$$R[0,0,0,0,0,0] \leftrightharpoons R[0,0,0,0,P,0] \quad kp30 \quad kp30d$$
$$R[0,0,0,0,0,P] \leftrightharpoons R[0,0,0,0,P,P] \quad kp30 \quad kp30d$$
$$R[0,0,0,P,0,0] \leftrightharpoons R[0,0,0,P,P,0] \quad kp30 \quad kp30d$$
$$R[0,0,0,P,0,P] \leftrightharpoons R[0,0,0,P,P,P] \quad kp30 \quad kp30d$$
$$R[0,0,P,0,0,0] \leftrightharpoons R[0,0,P,0,P,0] \quad kp30 \quad kp30d$$
$$R[0,0,P,0,0,P] \leftrightharpoons R[0,0,P,0,P,P] \quad kp30 \quad kp30d$$
$$R[0,0,P,P,0,0] \leftrightharpoons R[0,0,P,P,P,0] \quad kp30 \quad kp30d$$

$$R[0,0,P,P,0,P] \leftrightharpoons R[0,0,P,P,P,P] \quad kp30 \quad kp30d$$
$$R[I,0,0,0,0,0] \leftrightharpoons R[I,0,0,0,P,0] \quad kp31 \quad kp31d$$
$$R[I,0,0,0,0,P] \leftrightharpoons R[I,0,0,0,P,P] \quad kp31 \quad kp31d$$
$$R[I,0,0,P,0,0] \leftrightharpoons R[I,0,0,P,P,0] \quad kp31 \quad kp31d$$
$$R[I,0,0,P,0,P] \leftrightharpoons R[I,0,0,P,P,P] \quad kp31 \quad kp31d$$
$$R[I,0,P,0,0,0] \leftrightharpoons R[I,0,P,0,P,0] \quad kp31 \quad kp31d$$
$$R[I,0,P,0,0,P] \leftrightharpoons R[I,0,P,0,P,P] \quad kp31 \quad kp31d$$
$$R[I,0,P,P,0,0] \leftrightharpoons R[I,0,P,P,P,0] \quad kp31 \quad kp31d$$
$$R[I,0,P,P,0,P] \leftrightharpoons R[I,0,P,P,P,P] \quad kp31 \quad kp31d$$
$$R[0,I,0,0,0,0] \leftrightharpoons R[0,I,0,0,P,0] \quad kp31 \quad kp31d$$
$$R[0,I,0,0,0,P] \leftrightharpoons R[0,I,0,0,P,P] \quad kp31 \quad kp31d$$
$$R[0,I,0,P,0,0] \leftrightharpoons R[0,I,0,P,P,0] \quad kp31 \quad kp31d$$
$$R[0,I,0,P,0,P] \leftrightharpoons R[0,I,0,P,P,P] \quad kp31 \quad kp31d$$
$$R[0,I,P,0,0,0] \leftrightharpoons R[0,I,P,0,P,0] \quad kp31 \quad kp31d$$
$$R[0,I,P,0,0,P] \leftrightharpoons R[0,I,P,0,P,P] \quad kp31 \quad kp31d$$
$$R[0,I,P,P,0,0] \leftrightharpoons R[0,I,P,P,P,0] \quad kp31 \quad kp31d$$
$$R[0,I,P,P,0,P] \leftrightharpoons R[0,I,P,P,P,P] \quad kp31 \quad kp31d$$
$$R[I,I,0,0,0,0] \leftrightharpoons R[I,I,0,0,P,0] \quad kp32 \quad kp32d$$
$$R[I,I,0,0,0,P] \leftrightharpoons R[I,I,0,0,P,P] \quad kp32 \quad kp32d$$
$$R[I,I,0,P,0,0] \leftrightharpoons R[I,I,0,P,P,0] \quad kp32 \quad kp32d$$
$$R[I,I,0,P,0,P] \leftrightharpoons R[I,I,0,P,P,P] \quad kp32 \quad kp32d$$
$$R[I,I,P,0,0,0] \leftrightharpoons R[I,I,P,0,P,0] \quad kp32 \quad kp32d$$
$$R[I,I,P,0,0,P] \leftrightharpoons R[I,I,P,0,P,P] \quad kp32 \quad kp32d$$
$$R[I,I,P,P,0,0] \leftrightharpoons R[I,I,P,P,P,0] \quad kp32 \quad kp32d$$
$$R[I,I,P,P,0,P] \leftrightharpoons R[I,I,P,P,P,P] \quad kp32 \quad kp32d$$
$$R[0,0,0,0,0,0] \leftrightharpoons R[0,0,0,0,0,P] \quad kp40 \quad kp40d$$
$$R[0,0,0,0,P,0] \leftrightharpoons R[0,0,0,0,P,P] \quad kp40 \quad kp40d$$
$$R[0,0,0,P,0,0] \leftrightharpoons R[0,0,0,P,0,P] \quad kp40 \quad kp40d$$
$$R[0,0,0,P,P,0] \leftrightharpoons R[0,0,0,P,P,P] \quad kp40 \quad kp40d$$
$$R[0,0,P,0,0,0] \leftrightharpoons R[0,0,P,0,0,P] \quad kp40 \quad kp40d$$
$$R[0,0,P,0,P,0] \leftrightharpoons R[0,0,P,0,P,P] \quad kp40 \quad kp40d$$
$$R[0,0,P,P,0,0] \leftrightharpoons R[0,0,P,P,0,P] \quad kp40 \quad kp40d$$
$$R[0,0,P,P,P,0] \leftrightharpoons R[0,0,P,P,P,P] \quad kp40 \quad kp40d$$
$$R[I,0,0,0,0,0] \leftrightharpoons R[I,0,0,0,0,P] \quad kp41 \quad kp41d$$
$$R[I,0,0,0,P,0] \leftrightharpoons R[I,0,0,0,P,P] \quad kp41 \quad kp41d$$
$$R[I,0,0,P,0,0] \leftrightharpoons R[I,0,0,P,0,P] \quad kp41 \quad kp41d$$
$$R[I,0,0,P,P,0] \leftrightharpoons R[I,0,0,P,P,P] \quad kp41 \quad kp41d$$
$$R[I,0,P,0,0,0] \leftrightharpoons R[I,0,P,0,0,P] \quad kp41 \quad kp41d$$
$$R[I,0,P,0,P,0] \leftrightharpoons R[I,0,P,0,P,P] \quad kp41 \quad kp41d$$
$$R[I,0,P,P,0,0] \leftrightharpoons R[I,0,P,P,0,P] \quad kp41 \quad kp41d$$
$$R[I,0,P,P,P,0] \leftrightharpoons R[I,0,P,P,P,P] \quad kp41 \quad kp41d$$
$$R[0,I,0,0,0,0] \leftrightharpoons R[0,I,0,0,0,P] \quad kp41 \quad kp41d$$
$$R[0,I,0,0,P,0] \leftrightharpoons R[0,I,0,0,P,P] \quad kp41 \quad kp41d$$
$$R[0,I,0,P,0,0] \leftrightharpoons R[0,I,0,P,0,P] \quad kp41 \quad kp41d$$
$$R[0,I,0,P,P,0] \leftrightharpoons R[0,I,0,P,P,P] \quad kp41 \quad kp41d$$

$$R[0,I,P,0,0,0] \leftrightharpoons R[0,I,P,0,0,P] \quad kp41 \quad kp41d$$
$$R[0,I,P,0,P,0] \leftrightharpoons R[0,I,P,0,P,P] \quad kp41 \quad kp41d$$
$$R[0,I,P,P,0,0] \leftrightharpoons R[0,I,P,P,0,P] \quad kp41 \quad kp41d$$
$$R[0,I,P,P,P,0] \leftrightharpoons R[0,I,P,P,P,P] \quad kp41 \quad kp41d$$
$$R[I,I,0,0,0,0] \leftrightharpoons R[I,I,0,0,0,P] \quad kp42 \quad kp42d$$
$$R[I,I,0,0,P,0] \leftrightharpoons R[I,I,0,0,P,P] \quad kp42 \quad kp42d$$
$$R[I,I,0,P,0,0] \leftrightharpoons R[I,I,0,P,0,P] \quad kp42 \quad kp42d$$
$$R[I,I,0,P,P,0] \leftrightharpoons R[I,I,0,P,P,P] \quad kp42 \quad kp42d$$
$$R[I,I,P,0,0,0] \leftrightharpoons R[I,I,P,0,0,P] \quad kp42 \quad kp42d$$
$$R[I,I,P,0,P,0] \leftrightharpoons R[I,I,P,0,P,P] \quad kp42 \quad kp42d$$
$$R[I,I,P,P,0,0] \leftrightharpoons R[I,I,P,P,0,P] \quad kp42 \quad kp42d$$
$$R[I,I,P,P,P,0] \leftrightharpoons R[I,I,P,P,P,P] \quad kp42 \quad kp42d$$

d) translate each reaction into the corresponding rate

$$r0 = ki1 * R[0, 0, 0, 0, 0, 0] * Ins - ki1d * R[I, 0, 0, 0, 0, 0]$$
$$r1 = ki1 * R[0, 0, 0, 0, 0, P] * Ins - ki1d * R[I, 0, 0, 0, 0, P]$$
$$r2 = ki1 * R[0, 0, 0, 0, P, 0] * Ins - ki1d * R[I, 0, 0, 0, P, 0]$$
$$r3 = ki1 * R[0, 0, 0, 0, P, P] * Ins - ki1d * R[I, 0, 0, 0, P, P]$$
$$r4 = ki1 * R[0, 0, 0, P, 0, 0] * Ins - ki1d * R[I, 0, 0, P, 0, 0]$$
$$r5 = ki1 * R[0, 0, 0, P, 0, P] * Ins - ki1d * R[I, 0, 0, P, 0, P]$$
$$r6 = ki1 * R[0, 0, 0, P, P, 0] * Ins - ki1d * R[I, 0, 0, P, P, 0]$$
$$r7 = ki1 * R[0, 0, 0, P, P, P] * Ins - ki1d * R[I, 0, 0, P, P, P]$$
$$r8 = ki1 * R[0, 0, P, 0, 0, 0] * Ins - ki1d * R[I, 0, P, 0, 0, 0]$$
$$r9 = ki1 * R[0, 0, P, 0, 0, P] * Ins - ki1d * R[I, 0, P, 0, 0, P]$$
$$r10 = ki1 * R[0, 0, P, 0, P, 0] * Ins - ki1d * R[I, 0, P, 0, P, 0]$$
$$r11 = ki1 * R[0, 0, P, 0, P, P] * Ins - ki1d * R[I, 0, P, 0, P, P]$$
$$r12 = ki1 * R[0, 0, P, P, 0, 0] * Ins - ki1d * R[I, 0, P, P, 0, 0]$$
$$r13 = ki1 * R[0, 0, P, P, 0, P] * Ins - ki1d * R[I, 0, P, P, 0, P]$$
$$r14 = ki1 * R[0, 0, P, P, P, 0] * Ins - ki1d * R[I, 0, P, P, P, 0]$$
$$r15 = ki1 * R[0, 0, P, P, P, P] * Ins - ki1d * R[I, 0, P, P, P, P]$$
$$r16 = ki1 * R[0, 0, 0, 0, 0, 0] * Ins - ki1d * R[0, I, 0, 0, 0, 0]$$
$$r17 = ki1 * R[0, 0, 0, 0, 0, P] * Ins - ki1d * R[0, I, 0, 0, 0, P]$$
$$r18 = ki1 * R[0, 0, 0, 0, P, 0] * Ins - ki1d * R[0, I, 0, 0, P, 0]$$
$$r19 = ki1 * R[0, 0, 0, 0, P, P] * Ins - ki1d * R[0, I, 0, 0, P, P]$$
$$r20 = ki1 * R[0, 0, 0, P, 0, 0] * Ins - ki1d * R[0, I, 0, P, 0, 0]$$
$$r21 = ki1 * R[0, 0, 0, P, 0, P] * Ins - ki1d * R[0, I, 0, P, 0, P]$$
$$r22 = ki1 * R[0, 0, 0, P, P, 0] * Ins - ki1d * R[0, I, 0, P, P, 0]$$
$$r23 = ki1 * R[0, 0, 0, P, P, P] * Ins - ki1d * R[0, I, 0, P, P, P]$$
$$r24 = ki1 * R[0, 0, P, 0, 0, 0] * Ins - ki1d * R[0, I, P, 0, 0, 0]$$
$$r25 = ki1 * R[0, 0, P, 0, 0, P] * Ins - ki1d * R[0, I, P, 0, 0, P]$$
$$r26 = ki1 * R[0, 0, P, 0, P, 0] * Ins - ki1d * R[0, I, P, 0, P, 0]$$
$$r27 = ki1 * R[0, 0, P, 0, P, P] * Ins - ki1d * R[0, I, P, 0, P, P]$$
$$r28 = ki1 * R[0, 0, P, P, 0, 0] * Ins - ki1d * R[0, I, P, P, 0, 0]$$
$$r29 = ki1 * R[0, 0, P, P, 0, P] * Ins - ki1d * R[0, I, P, P, 0, P]$$
$$r30 = ki1 * R[0, 0, P, P, P, 0] * Ins - ki1d * R[0, I, P, P, P, 0]$$
$$r31 = ki1 * R[0, 0, P, P, P, P] * Ins - ki1d * R[0, I, P, P, P, P]$$
$$r32 = ki2 * R[I, 0, 0, 0, 0, 0] * Ins - ki2d * R[I, I, 0, 0, 0, 0]$$
$$r33 = ki2 * R[I, 0, 0, 0, 0, P] * Ins - ki2d * R[I, I, 0, 0, 0, P]$$
$$r34 = ki2 * R[I, 0, 0, 0, P, 0] * Ins - ki2d * R[I, I, 0, 0, P, 0]$$
$$r35 = ki2 * R[I, 0, 0, 0, P, P] * Ins - ki2d * R[I, I, 0, 0, P, P]$$
$$r36 = ki2 * R[I, 0, 0, P, 0, 0] * Ins - ki2d * R[I, I, 0, P, 0, 0]$$
$$r37 = ki2 * R[I, 0, 0, P, 0, P] * Ins - ki2d * R[I, I, 0, P, 0, P]$$
$$r38 = ki2 * R[I, 0, 0, P, P, 0] * Ins - ki2d * R[I, I, 0, P, P, 0]$$
$$r39 = ki2 * R[I, 0, 0, P, P, P] * Ins - ki2d * R[I, I, 0, P, P, P]$$
$$r40 = ki2 * R[I, 0, P, 0, 0, 0] * Ins - ki2d * R[I, I, P, 0, 0, 0]$$
$$r41 = ki2 * R[I, 0, P, 0, 0, P] * Ins - ki2d * R[I, I, P, 0, 0, P]$$
$$r42 = ki2 * R[I, 0, P, 0, P, 0] * Ins - ki2d * R[I, I, P, 0, P, 0]$$
$$r43 = ki2 * R[I, 0, P, 0, P, P] * Ins - ki2d * R[I, I, P, 0, P, P]$$
$$r44 = ki2 * R[I, 0, P, P, 0, 0] * Ins - ki2d * R[I, I, P, P, 0, 0]$$

$$r45 = ki2 * R[I,0,P,P,0,P] * Ins - ki2d * R[I,I,P,P,0,P]$$
$$r46 = ki2 * R[I,0,P,P,P,0] * Ins - ki2d * R[I,I,P,P,P,0]$$
$$r47 = ki2 * R[I,0,P,P,P,P] * Ins - ki2d * R[I,I,P,P,P,P]$$
$$r48 = ki2 * R[0,I,0,0,0,0] * Ins - ki2d * R[I,I,0,0,0,0]$$
$$r49 = ki2 * R[0,I,0,0,0,P] * Ins - ki2d * R[I,I,0,0,0,P]$$
$$r50 = ki2 * R[0,I,0,0,P,0] * Ins - ki2d * R[I,I,0,0,P,0]$$
$$r51 = ki2 * R[0,I,0,0,P,P] * Ins - ki2d * R[I,I,0,0,P,P]$$
$$r52 = ki2 * R[0,I,0,P,0,0] * Ins - ki2d * R[I,I,0,P,0,0]$$
$$r53 = ki2 * R[0,I,0,P,0,P] * Ins - ki2d * R[I,I,0,P,0,P]$$
$$r54 = ki2 * R[0,I,0,P,P,0] * Ins - ki2d * R[I,I,0,P,P,0]$$
$$r55 = ki2 * R[0,I,0,P,P,P] * Ins - ki2d * R[I,I,0,P,P,P]$$
$$r56 = ki2 * R[0,I,P,0,0,0] * Ins - ki2d * R[I,I,P,0,0,0]$$
$$r57 = ki2 * R[0,I,P,0,0,P] * Ins - ki2d * R[I,I,P,0,0,P]$$
$$r58 = ki2 * R[0,I,P,0,P,0] * Ins - ki2d * R[I,I,P,0,P,0]$$
$$r59 = ki2 * R[0,I,P,0,P,P] * Ins - ki2d * R[I,I,P,0,P,P]$$
$$r60 = ki2 * R[0,I,P,P,0,0] * Ins - ki2d * R[I,I,P,P,0,0]$$
$$r61 = ki2 * R[0,I,P,P,0,P] * Ins - ki2d * R[I,I,P,P,0,P]$$
$$r62 = ki2 * R[0,I,P,P,P,0] * Ins - ki2d * R[I,I,P,P,P,0]$$
$$r63 = ki2 * R[0,I,P,P,P,P] * Ins - ki2d * R[I,I,P,P,P,P]$$
$$r64 = kp10 * R[0,0,0,0,0,0] - kp10d * ((xRIrs - xRIrsb)/xRIrs) * R[0,0,P,0,0,0]$$
$$r65 = kp10 * R[0,0,0,0,0,P] - kp10d * ((xRIrs - xRIrsb)/xRIrs) * R[0,0,P,0,0,P]$$
$$r66 = kp10 * R[0,0,0,0,P,0] - kp10d * ((xRIrs - xRIrsb)/xRIrs) * R[0,0,P,0,P,0]$$
$$r67 = kp10 * R[0,0,0,0,P,P] - kp10d * ((xRIrs - xRIrsb)/xRIrs) * R[0,0,P,0,P,P]$$
$$r68 = kp10 * R[0,0,0,P,0,0] - kp10d * ((xRIrs - xRIrsb)/xRIrs) * R[0,0,P,P,0,0]$$
$$r69 = kp10 * R[0,0,0,P,0,P] - kp10d * ((xRIrs - xRIrsb)/xRIrs) * R[0,0,P,P,0,P]$$
$$r70 = kp10 * R[0,0,0,P,P,0] - kp10d * ((xRIrs - xRIrsb)/xRIrs) * R[0,0,P,P,P,0]$$
$$r71 = kp10 * R[0,0,0,P,P,P] - kp10d * ((xRIrs - xRIrsb)/xRIrs) * R[0,0,P,P,P,P]$$
$$r72 = kp11 * R[I,0,0,0,0,0] - kp11d * ((xRIrs - xRIrsb)/xRIrs) * R[I,0,P,0,0,0]$$
$$r73 = kp11 * R[I,0,0,0,0,P] - kp11d * ((xRIrs - xRIrsb)/xRIrs) * R[I,0,P,0,0,P]$$
$$r74 = kp11 * R[I,0,0,0,P,0] - kp11d * ((xRIrs - xRIrsb)/xRIrs) * R[I,0,P,0,P,0]$$
$$r75 = kp11 * R[I,0,0,0,P,P] - kp11d * ((xRIrs - xRIrsb)/xRIrs) * R[I,0,P,0,P,P]$$
$$r76 = kp11 * R[I,0,0,P,0,0] - kp11d * ((xRIrs - xRIrsb)/xRIrs) * R[I,0,P,P,0,0]$$
$$r77 = kp11 * R[I,0,0,P,0,P] - kp11d * ((xRIrs - xRIrsb)/xRIrs) * R[I,0,P,P,0,P]$$
$$r78 = kp11 * R[I,0,0,P,P,0] - kp11d * ((xRIrs - xRIrsb)/xRIrs) * R[I,0,P,P,P,0]$$
$$r79 = kp11 * R[I,0,0,P,P,P] - kp11d * ((xRIrs - xRIrsb)/xRIrs) * R[I,0,P,P,P,P]$$
$$r80 = kp11 * R[0,I,0,0,0,0] - kp11d * ((xRIrs - xRIrsb)/xRIrs) * R[0,I,P,0,0,0]$$
$$r81 = kp11 * R[0,I,0,0,0,P] - kp11d * ((xRIrs - xRIrsb)/xRIrs) * R[0,I,P,0,0,P]$$
$$r82 = kp11 * R[0,I,0,0,P,0] - kp11d * ((xRIrs - xRIrsb)/xRIrs) * R[0,I,P,0,P,0]$$
$$r83 = kp11 * R[0,I,0,0,P,P] - kp11d * ((xRIrs - xRIrsb)/xRIrs) * R[0,I,P,0,P,P]$$
$$r84 = kp11 * R[0,I,0,P,0,0] - kp11d * ((xRIrs - xRIrsb)/xRIrs) * R[0,I,P,P,0,0]$$
$$r85 = kp11 * R[0,I,0,P,0,P] - kp11d * ((xRIrs - xRIrsb)/xRIrs) * R[0,I,P,P,0,P]$$
$$r86 = kp11 * R[0,I,0,P,P,0] - kp11d * ((xRIrs - xRIrsb)/xRIrs) * R[0,I,P,P,P,0]$$
$$r87 = kp11 * R[0,I,0,P,P,P] - kp11d * ((xRIrs - xRIrsb)/xRIrs) * R[0,I,P,P,P,P]$$
$$r88 = kp12 * R[I,I,0,0,0,0] - kp12d * ((xRIrs - xRIrsb)/xRIrs) * R[I,I,P,0,0,0]$$
$$r89 = kp12 * R[I,I,0,0,0,P] - kp12d * ((xRIrs - xRIrsb)/xRIrs) * R[I,I,P,0,0,P]$$

$$r90 = kp12 * R[I, I, 0, 0, P, 0] - kp12d * ((xRIrs - xRIrsb)/xRIrs) * R[I, I, P, 0, P, 0]$$
$$r91 = kp12 * R[I, I, 0, 0, P, P] - kp12d * ((xRIrs - xRIrsb)/xRIrs) * R[I, I, P, 0, P, P]$$
$$r92 = kp12 * R[I, I, 0, P, 0, 0] - kp12d * ((xRIrs - xRIrsb)/xRIrs) * R[I, I, P, P, 0, 0]$$
$$r93 = kp12 * R[I, I, 0, P, 0, P] - kp12d * ((xRIrs - xRIrsb)/xRIrs) * R[I, I, P, P, 0, P]$$
$$r94 = kp12 * R[I, I, 0, P, P, 0] - kp12d * ((xRIrs - xRIrsb)/xRIrs) * R[I, I, P, P, P, 0]$$
$$r95 = kp12 * R[I, I, 0, P, P, P] - kp12d * ((xRIrs - xRIrsb)/xRIrs) * R[I, I, P, P, P, P]$$
$$r96 = kp20 * R[0, 0, 0, 0, 0, 0] - kp20d * ((xRIrs - xRIrsb)/xRIrs) * R[0, 0, 0, P, 0, 0]$$
$$r97 = kp20 * R[0, 0, 0, 0, 0, P] - kp20d * ((xRIrs - xRIrsb)/xRIrs) * R[0, 0, 0, P, 0, P]$$
$$r98 = kp20 * R[0, 0, 0, 0, P, 0] - kp20d * ((xRIrs - xRIrsb)/xRIrs) * R[0, 0, 0, P, P, 0]$$
$$r99 = kp20 * R[0, 0, 0, 0, P, P] - kp20d * ((xRIrs - xRIrsb)/xRIrs) * R[0, 0, 0, P, P, P]$$
$$r100 = kp20 * R[0, 0, P, 0, 0, 0] - kp20d * ((xRIrs - xRIrsb)/xRIrs) * R[0, 0, P, P, 0, 0]$$
$$r101 = kp20 * R[0, 0, P, 0, 0, P] - kp20d * ((xRIrs - xRIrsb)/xRIrs) * R[0, 0, P, P, 0, P]$$
$$r102 = kp20 * R[0, 0, P, 0, P, 0] - kp20d * ((xRIrs - xRIrsb)/xRIrs) * R[0, 0, P, P, P, 0]$$
$$r103 = kp20 * R[0, 0, P, 0, P, P] - kp20d * ((xRIrs - xRIrsb)/xRIrs) * R[0, 0, P, P, P, P]$$
$$r104 = kp21 * R[I, 0, 0, 0, 0, 0] - kp21d * ((xRIrs - xRIrsb)/xRIrs) * R[I, 0, 0, P, 0, 0]$$
$$r105 = kp21 * R[I, 0, 0, 0, 0, P] - kp21d * ((xRIrs - xRIrsb)/xRIrs) * R[I, 0, 0, P, 0, P]$$
$$r106 = kp21 * R[I, 0, 0, 0, P, 0] - kp21d * ((xRIrs - xRIrsb)/xRIrs) * R[I, 0, 0, P, P, 0]$$
$$r107 = kp21 * R[I, 0, 0, 0, P, P] - kp21d * ((xRIrs - xRIrsb)/xRIrs) * R[I, 0, 0, P, P, P]$$
$$r108 = kp21 * R[I, 0, P, 0, 0, 0] - kp21d * ((xRIrs - xRIrsb)/xRIrs) * R[I, 0, P, P, 0, 0]$$
$$r109 = kp21 * R[I, 0, P, 0, 0, P] - kp21d * ((xRIrs - xRIrsb)/xRIrs) * R[I, 0, P, P, 0, P]$$
$$r110 = kp21 * R[I, 0, P, 0, P, 0] - kp21d * ((xRIrs - xRIrsb)/xRIrs) * R[I, 0, P, P, P, 0]$$
$$r111 = kp21 * R[I, 0, P, 0, P, P] - kp21d * ((xRIrs - xRIrsb)/xRIrs) * R[I, 0, P, P, P, P]$$
$$r112 = kp21 * R[0, I, 0, 0, 0, 0] - kp21d * ((xRIrs - xRIrsb)/xRIrs) * R[0, I, 0, P, 0, 0]$$
$$r113 = kp21 * R[0, I, 0, 0, 0, P] - kp21d * ((xRIrs - xRIrsb)/xRIrs) * R[0, I, 0, P, 0, P]$$
$$r114 = kp21 * R[0, I, 0, 0, P, 0] - kp21d * ((xRIrs - xRIrsb)/xRIrs) * R[0, I, 0, P, P, 0]$$
$$r115 = kp21 * R[0, I, 0, 0, P, P] - kp21d * ((xRIrs - xRIrsb)/xRIrs) * R[0, I, 0, P, P, P]$$
$$r116 = kp21 * R[0, I, P, 0, 0, 0] - kp21d * ((xRIrs - xRIrsb)/xRIrs) * R[0, I, P, P, 0, 0]$$
$$r117 = kp21 * R[0, I, P, 0, 0, P] - kp21d * ((xRIrs - xRIrsb)/xRIrs) * R[0, I, P, P, 0, P]$$
$$r118 = kp21 * R[0, I, P, 0, P, 0] - kp21d * ((xRIrs - xRIrsb)/xRIrs) * R[0, I, P, P, P, 0]$$
$$r119 = kp21 * R[0, I, P, 0, P, P] - kp21d * ((xRIrs - xRIrsb)/xRIrs) * R[0, I, P, P, P, P]$$
$$r120 = kp22 * R[I, I, 0, 0, 0, 0] - kp22d * ((xRIrs - xRIrsb)/xRIrs) * R[I, I, 0, P, 0, 0]$$
$$r121 = kp22 * R[I, I, 0, 0, 0, P] - kp22d * ((xRIrs - xRIrsb)/xRIrs) * R[I, I, 0, P, 0, P]$$
$$r122 = kp22 * R[I, I, 0, 0, P, 0] - kp22d * ((xRIrs - xRIrsb)/xRIrs) * R[I, I, 0, P, P, 0]$$
$$r123 = kp22 * R[I, I, 0, 0, P, P] - kp22d * ((xRIrs - xRIrsb)/xRIrs) * R[I, I, 0, P, P, P]$$
$$r124 = kp22 * R[I, I, P, 0, 0, 0] - kp22d * ((xRIrs - xRIrsb)/xRIrs) * R[I, I, P, P, 0, 0]$$
$$r125 = kp22 * R[I, I, P, 0, 0, P] - kp22d * ((xRIrs - xRIrsb)/xRIrs) * R[I, I, P, P, 0, P]$$
$$r126 = kp22 * R[I, I, P, 0, P, 0] - kp22d * ((xRIrs - xRIrsb)/xRIrs) * R[I, I, P, P, P, 0]$$
$$r127 = kp22 * R[I, I, P, 0, P, P] - kp22d * ((xRIrs - xRIrsb)/xRIrs) * R[I, I, P, P, P, P]$$
$$r128 = kp30 * R[0, 0, 0, 0, 0, 0] - kp30d * ((xRShc - xRShcb)/xRShc) * R[0, 0, 0, 0, P, 0]$$
$$r129 = kp30 * R[0, 0, 0, 0, 0, P] - kp30d * ((xRShc - xRShcb)/xRShc) * R[0, 0, 0, 0, P, P]$$
$$r130 = kp30 * R[0, 0, 0, P, 0, 0] - kp30d * ((xRShc - xRShcb)/xRShc) * R[0, 0, 0, P, P, 0]$$
$$r131 = kp30 * R[0, 0, 0, P, 0, P] - kp30d * ((xRShc - xRShcb)/xRShc) * R[0, 0, 0, P, P, P]$$
$$r132 = kp30 * R[0, 0, P, 0, 0, 0] - kp30d * ((xRShc - xRShcb)/xRShc) * R[0, 0, P, 0, P, 0]$$
$$r133 = kp30 * R[0, 0, P, 0, 0, P] - kp30d * ((xRShc - xRShcb)/xRShc) * R[0, 0, P, 0, P, P]$$
$$r134 = kp30 * R[0, 0, P, P, 0, 0] - kp30d * ((xRShc - xRShcb)/xRShc) * R[0, 0, P, P, P, 0]$$

$$r135 = kp30 * R[0, 0, P, P, 0, P] - kp30d * ((xRShc - xRShcb)/xRShc) * R[0, 0, P, P, P, P]$$
$$r136 = kp31 * R[I, 0, 0, 0, 0, 0] - kp31d * ((xRShc - xRShcb)/xRShc) * R[I, 0, 0, 0, P, 0]$$
$$r137 = kp31 * R[I, 0, 0, 0, 0, P] - kp31d * ((xRShc - xRShcb)/xRShc) * R[I, 0, 0, 0, P, P]$$
$$r138 = kp31 * R[I, 0, 0, P, 0, 0] - kp31d * ((xRShc - xRShcb)/xRShc) * R[I, 0, 0, P, P, 0]$$
$$r139 = kp31 * R[I, 0, 0, P, 0, P] - kp31d * ((xRShc - xRShcb)/xRShc) * R[I, 0, 0, P, P, P]$$
$$r140 = kp31 * R[I, 0, P, 0, 0, 0] - kp31d * ((xRShc - xRShcb)/xRShc) * R[I, 0, P, 0, P, 0]$$
$$r141 = kp31 * R[I, 0, P, 0, 0, P] - kp31d * ((xRShc - xRShcb)/xRShc) * R[I, 0, P, 0, P, P]$$
$$r142 = kp31 * R[I, 0, P, P, 0, 0] - kp31d * ((xRShc - xRShcb)/xRShc) * R[I, 0, P, P, P, 0]$$
$$r143 = kp31 * R[I, 0, P, P, 0, P] - kp31d * ((xRShc - xRShcb)/xRShc) * R[I, 0, P, P, P, P]$$
$$r144 = kp31 * R[0, I, 0, 0, 0, 0] - kp31d * ((xRShc - xRShcb)/xRShc) * R[0, I, 0, 0, P, 0]$$
$$r145 = kp31 * R[0, I, 0, 0, 0, P] - kp31d * ((xRShc - xRShcb)/xRShc) * R[0, I, 0, 0, P, P]$$
$$r146 = kp31 * R[0, I, 0, P, 0, 0] - kp31d * ((xRShc - xRShcb)/xRShc) * R[0, I, 0, P, P, 0]$$
$$r147 = kp31 * R[0, I, 0, P, 0, P] - kp31d * ((xRShc - xRShcb)/xRShc) * R[0, I, 0, P, P, P]$$
$$r148 = kp31 * R[0, I, P, 0, 0, 0] - kp31d * ((xRShc - xRShcb)/xRShc) * R[0, I, P, 0, P, 0]$$
$$r149 = kp31 * R[0, I, P, 0, 0, P] - kp31d * ((xRShc - xRShcb)/xRShc) * R[0, I, P, 0, P, P]$$
$$r150 = kp31 * R[0, I, P, P, 0, 0] - kp31d * ((xRShc - xRShcb)/xRShc) * R[0, I, P, P, P, 0]$$
$$r151 = kp31 * R[0, I, P, P, 0, P] - kp31d * ((xRShc - xRShcb)/xRShc) * R[0, I, P, P, P, P]$$
$$r152 = kp32 * R[I, I, 0, 0, 0, 0] - kp32d * ((xRShc - xRShcb)/xRShc) * R[I, I, 0, 0, P, 0]$$
$$r153 = kp32 * R[I, I, 0, 0, 0, P] - kp32d * ((xRShc - xRShcb)/xRShc) * R[I, I, 0, 0, P, P]$$
$$r154 = kp32 * R[I, I, 0, P, 0, 0] - kp32d * ((xRShc - xRShcb)/xRShc) * R[I, I, 0, P, P, 0]$$
$$r155 = kp32 * R[I, I, 0, P, 0, P] - kp32d * ((xRShc - xRShcb)/xRShc) * R[I, I, 0, P, P, P]$$
$$r156 = kp32 * R[I, I, P, 0, 0, 0] - kp32d * ((xRShc - xRShcb)/xRShc) * R[I, I, P, 0, P, 0]$$
$$r157 = kp32 * R[I, I, P, 0, 0, P] - kp32d * ((xRShc - xRShcb)/xRShc) * R[I, I, P, 0, P, P]$$
$$r158 = kp32 * R[I, I, P, P, 0, 0] - kp32d * ((xRShc - xRShcb)/xRShc) * R[I, I, P, P, P, 0]$$
$$r159 = kp32 * R[I, I, P, P, 0, P] - kp32d * ((xRShc - xRShcb)/xRShc) * R[I, I, P, P, P, P]$$
$$r160 = kp40 * R[0, 0, 0, 0, 0, 0] - kp40d * ((xRShc - xRShcb)/xRShc) * R[0, 0, 0, 0, 0, P]$$
$$r161 = kp40 * R[0, 0, 0, 0, P, 0] - kp40d * ((xRShc - xRShcb)/xRShc) * R[0, 0, 0, 0, P, P]$$
$$r162 = kp40 * R[0, 0, 0, P, 0, 0] - kp40d * ((xRShc - xRShcb)/xRShc) * R[0, 0, 0, P, 0, P]$$
$$r163 = kp40 * R[0, 0, 0, P, P, 0] - kp40d * ((xRShc - xRShcb)/xRShc) * R[0, 0, 0, P, P, P]$$
$$r164 = kp40 * R[0, 0, P, 0, 0, 0] - kp40d * ((xRShc - xRShcb)/xRShc) * R[0, 0, P, 0, 0, P]$$
$$r165 = kp40 * R[0, 0, P, 0, P, 0] - kp40d * ((xRShc - xRShcb)/xRShc) * R[0, 0, P, 0, P, P]$$
$$r166 = kp40 * R[0, 0, P, P, 0, 0] - kp40d * ((xRShc - xRShcb)/xRShc) * R[0, 0, P, P, 0, P]$$
$$r167 = kp40 * R[0, 0, P, P, P, 0] - kp40d * ((xRShc - xRShcb)/xRShc) * R[0, 0, P, P, P, P]$$
$$r168 = kp41 * R[I, 0, 0, 0, 0, 0] - kp41d * ((xRShc - xRShcb)/xRShc) * R[I, 0, 0, 0, 0, P]$$
$$r169 = kp41 * R[I, 0, 0, 0, P, 0] - kp41d * ((xRShc - xRShcb)/xRShc) * R[I, 0, 0, 0, P, P]$$
$$r170 = kp41 * R[I, 0, 0, P, 0, 0] - kp41d * ((xRShc - xRShcb)/xRShc) * R[I, 0, 0, P, 0, P]$$
$$r171 = kp41 * R[I, 0, 0, P, P, 0] - kp41d * ((xRShc - xRShcb)/xRShc) * R[I, 0, 0, P, P, P]$$
$$r172 = kp41 * R[I, 0, P, 0, 0, 0] - kp41d * ((xRShc - xRShcb)/xRShc) * R[I, 0, P, 0, 0, P]$$
$$r173 = kp41 * R[I, 0, P, 0, P, 0] - kp41d * ((xRShc - xRShcb)/xRShc) * R[I, 0, P, 0, P, P]$$
$$r174 = kp41 * R[I, 0, P, P, 0, 0] - kp41d * ((xRShc - xRShcb)/xRShc) * R[I, 0, P, P, 0, P]$$
$$r175 = kp41 * R[I, 0, P, P, P, 0] - kp41d * ((xRShc - xRShcb)/xRShc) * R[I, 0, P, P, P, P]$$
$$r176 = kp41 * R[0, I, 0, 0, 0, 0] - kp41d * ((xRShc - xRShcb)/xRShc) * R[0, I, 0, 0, 0, P]$$
$$r177 = kp41 * R[0, I, 0, 0, P, 0] - kp41d * ((xRShc - xRShcb)/xRShc) * R[0, I, 0, 0, P, P]$$
$$r178 = kp41 * R[0, I, 0, P, 0, 0] - kp41d * ((xRShc - xRShcb)/xRShc) * R[0, I, 0, P, 0, P]$$
$$r179 = kp41 * R[0, I, 0, P, P, 0] - kp41d * ((xRShc - xRShcb)/xRShc) * R[0, I, 0, P, P, P]$$

$$r180 = kp41 * R[0, I, P, 0, 0, 0] - kp41d * ((xRShc - xRShcb)/xRShc) * R[0, I, P, 0, 0, P]$$
$$r181 = kp41 * R[0, I, P, 0, P, 0] - kp41d * ((xRShc - xRShcb)/xRShc) * R[0, I, P, 0, P, P]$$
$$r182 = kp41 * R[0, I, P, P, 0, 0] - kp41d * ((xRShc - xRShcb)/xRShc) * R[0, I, P, P, 0, P]$$
$$r183 = kp41 * R[0, I, P, P, P, 0] - kp41d * ((xRShc - xRShcb)/xRShc) * R[0, I, P, P, P, P]$$
$$r184 = kp42 * R[I, I, 0, 0, 0, 0] - kp42d * ((xRShc - xRShcb)/xRShc) * R[I, I, 0, 0, 0, P]$$
$$r185 = kp42 * R[I, I, 0, 0, P, 0] - kp42d * ((xRShc - xRShcb)/xRShc) * R[I, I, 0, 0, P, P]$$
$$r186 = kp42 * R[I, I, 0, P, 0, 0] - kp42d * ((xRShc - xRShcb)/xRShc) * R[I, I, 0, P, 0, P]$$
$$r187 = kp42 * R[I, I, 0, P, P, 0] - kp42d * ((xRShc - xRShcb)/xRShc) * R[I, I, 0, P, P, P]$$
$$r188 = kp42 * R[I, I, P, 0, 0, 0] - kp42d * ((xRShc - xRShcb)/xRShc) * R[I, I, P, 0, 0, P]$$
$$r189 = kp42 * R[I, I, P, 0, P, 0] - kp42d * ((xRShc - xRShcb)/xRShc) * R[I, I, P, 0, P, P]$$
$$r190 = kp42 * R[I, I, P, P, 0, 0] - kp42d * ((xRShc - xRShcb)/xRShc) * R[I, I, P, P, 0, P]$$
$$r191 = kp42 * R[I, I, P, P, P, 0] - kp42d * ((xRShc - xRShcb)/xRShc) * R[I, I, P, P, P, P]$$

e) construct ODEs

$$\frac{d}{dt}R[0,0,0,0,0,0] = -r0 - r16 - r64 - r96 - r128 - r160$$
$$\frac{d}{dt}R[0,0,0,0,0,P] = -r1 - r17 - r65 - r97 - r129 + r160$$
$$\frac{d}{dt}R[0,0,0,0,P,0] = -r2 - r18 - r66 - r98 + r128 - r161$$
$$\frac{d}{dt}R[0,0,0,0,P,P] = -r3 - r19 - r67 - r99 + r129 + r161$$
$$\frac{d}{dt}R[0,0,0,P,0,0] = -r4 - r20 - r68 + r96 - r130 - r162$$
$$\frac{d}{dt}R[0,0,0,P,0,P] = -r5 - r21 - r69 + r97 - r131 + r162$$
$$\frac{d}{dt}R[0,0,0,P,P,0] = -r6 - r22 - r70 + r98 + r130 - r163$$
$$\frac{d}{dt}R[0,0,0,P,P,P] = -r7 - r23 - r71 + r99 + r131 + r163$$
$$\frac{d}{dt}R[0,0,P,0,0,0] = -r8 - r24 + r64 - r100 - r132 - r164$$
$$\frac{d}{dt}R[0,0,P,0,0,P] = -r9 - r25 + r65 - r101 - r133 + r164$$
$$\frac{d}{dt}R[0,0,P,0,P,0] = -r10 - r26 + r66 - r102 + r132 - r165$$
$$\frac{d}{dt}R[0,0,P,0,P,P] = -r11 - r27 + r67 - r103 + r133 + r165$$
$$\frac{d}{dt}R[0,0,P,P,0,0] = -r12 - r28 + r68 + r100 - r134 - r166$$
$$\frac{d}{dt}R[0,0,P,P,0,P] = -r13 - r29 + r69 + r101 - r135 + r166$$
$$\frac{d}{dt}R[0,0,P,P,P,0] = -r14 - r30 + r70 + r102 + r134 - r167$$
$$\frac{d}{dt}R[0,0,P,P,P,P] = -r15 - r31 + r71 + r103 + r135 + r167$$
$$\frac{d}{dt}R[0,I,0,0,0,0] = r16 - r48 - r80 - r112 - r144 - r176$$

$$\frac{d}{dt}R[0,I,0,0,0,P] = r17 - r49 - r81 - r113 - r145 + r176$$
$$\frac{d}{dt}R[0,I,0,0,P,0] = r18 - r50 - r82 - r114 + r144 - r177$$
$$\frac{d}{dt}R[0,I,0,0,P,P] = r19 - r51 - r83 - r115 + r145 + r177$$
$$\frac{d}{dt}R[0,I,0,P,0,0] = r20 - r52 - r84 + r112 - r146 - r178$$
$$\frac{d}{dt}R[0,I,0,P,0,P] = r21 - r53 - r85 + r113 - r147 + r178$$
$$\frac{d}{dt}R[0,I,0,P,P,0] = r22 - r54 - r86 + r114 + r146 - r179$$
$$\frac{d}{dt}R[0,I,0,P,P,P] = r23 - r55 - r87 + r115 + r147 + r179$$
$$\frac{d}{dt}R[0,I,P,0,0,0] = r24 - r56 + r80 - r116 - r148 - r180$$
$$\frac{d}{dt}R[0,I,P,0,0,P] = r25 - r57 + r81 - r117 - r149 + r180$$
$$\frac{d}{dt}R[0,I,P,0,P,0] = r26 - r58 + r82 - r118 + r148 - r181$$
$$\frac{d}{dt}R[0,I,P,0,P,P] = r27 - r59 + r83 - r119 + r149 + r181$$
$$\frac{d}{dt}R[0,I,P,P,0,0] = r28 - r60 + r84 + r116 - r150 - r182$$
$$\frac{d}{dt}R[0,I,P,P,0,P] = r29 - r61 + r85 + r117 - r151 + r182$$
$$\frac{d}{dt}R[0,I,P,P,P,0] = r30 - r62 + r86 + r118 + r150 - r183$$
$$\frac{d}{dt}R[0,I,P,P,P,P] = r31 - r63 + r87 + r119 + r151 + r183$$
$$\frac{d}{dt}R[I,0,0,0,0,0] = r0 - r32 - r72 - r104 - r136 - r168$$
$$\frac{d}{dt}R[I,0,0,0,0,P] = r1 - r33 - r73 - r105 - r137 + r168$$
$$\frac{d}{dt}R[I,0,0,0,P,0] = r2 - r34 - r74 - r106 + r136 - r169$$
$$\frac{d}{dt}R[I,0,0,0,P,P] = r3 - r35 - r75 - r107 + r137 + r169$$
$$\frac{d}{dt}R[I,0,0,P,0,0] = r4 - r36 - r76 + r104 - r138 - r170$$
$$\frac{d}{dt}R[I,0,0,P,0,P] = r5 - r37 - r77 + r105 - r139 + r170$$

$$\frac{d}{dt}R[I,0,0,P,P,0] = r6 - r38 - r78 + r106 + r138 - r171$$
$$\frac{d}{dt}R[I,0,0,P,P,P] = r7 - r39 - r79 + r107 + r139 + r171$$
$$\frac{d}{dt}R[I,0,P,0,0,0] = r8 - r40 + r72 - r108 - r140 - r172$$
$$\frac{d}{dt}R[I,0,P,0,0,P] = r9 - r41 + r73 - r109 - r141 + r172$$
$$\frac{d}{dt}R[I,0,P,0,P,0] = r10 - r42 + r74 - r110 + r140 - r173$$
$$\frac{d}{dt}R[I,0,P,0,P,P] = r11 - r43 + r75 - r111 + r141 + r173$$
$$\frac{d}{dt}R[I,0,P,P,0,0] = r12 - r44 + r76 + r108 - r142 - r174$$
$$\frac{d}{dt}R[I,0,P,P,0,P] = r13 - r45 + r77 + r109 - r143 + r174$$
$$\frac{d}{dt}R[I,0,P,P,P,0] = r14 - r46 + r78 + r110 + r142 - r175$$
$$\frac{d}{dt}R[I,0,P,P,P,P] = r15 - r47 + r79 + r111 + r143 + r175$$
$$\frac{d}{dt}R[I,I,0,0,0,0] = r32 + r48 - r88 - r120 - r152 - r184$$
$$\frac{d}{dt}R[I,I,0,0,0,P] = r33 + r49 - r89 - r121 - r153 + r184$$
$$\frac{d}{dt}R[I,I,0,0,P,0] = r34 + r50 - r90 - r122 + r152 - r185$$
$$\frac{d}{dt}R[I,I,0,0,P,P] = r35 + r51 - r91 - r123 + r153 + r185$$
$$\frac{d}{dt}R[I,I,0,P,0,0] = r36 + r52 - r92 + r120 - r154 - r186$$
$$\frac{d}{dt}R[I,I,0,P,0,P] = r37 + r53 - r93 + r121 - r155 + r186$$
$$\frac{d}{dt}R[I,I,0,P,P,0] = r38 + r54 - r94 + r122 + r154 - r187$$
$$\frac{d}{dt}R[I,I,0,P,P,P] = r39 + r55 - r95 + r123 + r155 + r187$$
$$\frac{d}{dt}R[I,I,P,0,0,0] = r40 + r56 + r88 - r124 - r156 - r188$$
$$\frac{d}{dt}R[I,I,P,0,0,P] = r41 + r57 + r89 - r125 - r157 + r188$$
$$\frac{d}{dt}R[I,I,P,0,P,0] = r42 + r58 + r90 - r126 + r156 - r189$$
$$\frac{d}{dt}R[I,I,P,0,P,P] = r43 + r59 + r91 - r127 + r157 + r189$$
$$\frac{d}{dt}R[I,I,P,P,0,0] = r44 + r60 + r92 + r124 - r158 - r190$$
$$\frac{d}{dt}R[I,I,P,P,0,P] = r45 + r61 + r93 + r125 - r159 + r190$$
$$\frac{d}{dt}R[I,I,P,P,P,0] = r46 + r62 + r94 + r126 + r158 - r191$$
$$\frac{d}{dt}R[I,I,P,P,P,P] = r47 + r63 + r95 + r127 + r159 + r191$$

IRS layer

Processes of the IRS layer are IRS binding to the receptor and phosphorylation of six binding sites. The first four binding sites that can become phosphorylated are for PI3K, the next is for SHP2 and the last for Grb2. All binding sites can be phosphorylated (P) or unphosphorylated (0). The first site on IRS indicates if IRS is free (0) or bound to the receptor (R).

a) Define all sums of phosphorylated binding sites x_i and occupied binding sites x_ib

$$\begin{aligned} xIrsPi3k1 &= IRS[X, P, X, X, X, X, X] \\ xIrsPi3k2 &= IRS[X, X, P, X, X, X, X] \\ xIrsPi3k3 &= IRS[X, X, X, P, X, X, X] \\ xIrsPi3k4 &= IRS[X, X, X, X, P, X, X] \\ xIrsShp2 &= IRS[X, X, X, X, X, P, X] \\ xIrsGrb2 &= IRS[X, X, X, X, X, X, P] \\ xRIrsb &= IRS[R, X, X, X, X, X, X] \end{aligned}$$

$xIrsPi3k_i$ is the sum of phosphorylated binding sites for PI3K, for the binding site that is indexed by i. $xIrsShp2$ and $xIrsGrb2$ are the sums of phosphorylated binding sites on IRS for SHP2 and Grb2, respectively. xRIrsb is the sum of occupied binding sites for IRS on the receptor.

b) Define the concentrations of all unoccupied phosphorylated binding sites that are needed as binding partners within the considered layer

$$RXpIrs = xRIrs - xRIrsb$$

$RXpIrs$ is the sum of unoccupied phosphorylated binding sites for IRS on the receptor.

c) Define reaction rules and reactions (including dephosphorylation of binding sites) as if there were no other layers

IRS binding to the receptor

$$RXpIrs + IRS[0, X, X, X, X, X, X] \leftrightharpoons IRS[R, X, X, X, X, X, X] \quad kbirs \quad kbirsd$$

phosphorylation of binding sites on free IRS

$$\begin{aligned} IRS[0, 0, X, X, X, X, X] &\leftrightharpoons IRS[0, P, X, X, X, X, X] & kirsp1 \quad & kirsp1d \\ IRS[0, X, 0, X, X, X, X] &\leftrightharpoons IRS[0, X, P, X, X, X, X] & kirsp2 \quad & kirsp2d \\ IRS[0, X, X, 0, X, X, X] &\leftrightharpoons IRS[0, X, X, P, X, X, X] & kirsp3 \quad & kirsp3d \\ IRS[0, X, X, X, 0, X, X] &\leftrightharpoons IRS[0, X, X, X, P, X, X] & kirsp4 \quad & kirsp4d \\ IRS[0, X, X, X, X, 0, X] &\leftrightharpoons IRS[0, X, X, X, X, P, X] & kirsp5 \quad & kirsp5d \\ IRS[0, X, X, X, X, X, 0] &\leftrightharpoons IRS[0, X, X, X, X, X, P] & kirsp6 \quad & kirsp6d \end{aligned}$$

phosphorylation of binding sites on IRS bound to the receptor

$$\begin{aligned} IRS[R, 0, X, X, X, X, X] &\leftrightharpoons IRS[R, P, X, X, X, X, X] & kirsbp1 \quad & kirsbp1d \\ IRS[R, X, 0, X, X, X, X] &\leftrightharpoons IRS[R, X, P, X, X, X, X] & kirsbp2 \quad & kirsbp2d \\ IRS[R, X, X, 0, X, X, X] &\leftrightharpoons IRS[R, X, X, P, X, X, X] & kirsbp3 \quad & kirsbp3d \\ IRS[R, X, X, X, 0, X, X] &\leftrightharpoons IRS[R, X, X, X, P, X, X] & kirsbp4 \quad & kirsbp4d \\ IRS[R, X, X, X, X, 0, X] &\leftrightharpoons IRS[R, X, X, X, X, P, X] & kirsbp5 \quad & kirsbp5d \\ IRS[R, X, X, X, X, X, 0] &\leftrightharpoons IRS[R, X, X, X, X, X, P] & kirsbp6 \quad & kirsbp6d \end{aligned}$$

these rules correspond to the following reactions

$$RXpIrs + IRS[0,0,0,0,0,0,0] \rightleftharpoons IRS[R,0,0,0,0,0,0] \quad kbirs \quad kbirsd$$
$$RXpIrs + IRS[0,0,0,0,0,0,P] \rightleftharpoons IRS[R,0,0,0,0,0,P] \quad kbirs \quad kbirsd$$
$$RXpIrs + IRS[0,0,0,0,0,P,0] \rightleftharpoons IRS[R,0,0,0,0,P,0] \quad kbirs \quad kbirsd$$
$$RXpIrs + IRS[0,0,0,0,0,P,P] \rightleftharpoons IRS[R,0,0,0,0,P,P] \quad kbirs \quad kbirsd$$
$$RXpIrs + IRS[0,0,0,0,P,0,0] \rightleftharpoons IRS[R,0,0,0,P,0,0] \quad kbirs \quad kbirsd$$
$$RXpIrs + IRS[0,0,0,0,P,0,P] \rightleftharpoons IRS[R,0,0,0,P,0,P] \quad kbirs \quad kbirsd$$
$$RXpIrs + IRS[0,0,0,0,P,P,0] \rightleftharpoons IRS[R,0,0,0,P,P,0] \quad kbirs \quad kbirsd$$
$$RXpIrs + IRS[0,0,0,0,P,P,P] \rightleftharpoons IRS[R,0,0,0,P,P,P] \quad kbirs \quad kbirsd$$
$$RXpIrs + IRS[0,0,0,P,0,0,0] \rightleftharpoons IRS[R,0,0,P,0,0,0] \quad kbirs \quad kbirsd$$
$$RXpIrs + IRS[0,0,0,P,0,0,P] \rightleftharpoons IRS[R,0,0,P,0,0,P] \quad kbirs \quad kbirsd$$
$$RXpIrs + IRS[0,0,0,P,0,P,0] \rightleftharpoons IRS[R,0,0,P,0,P,0] \quad kbirs \quad kbirsd$$
$$RXpIrs + IRS[0,0,0,P,0,P,P] \rightleftharpoons IRS[R,0,0,P,0,P,P] \quad kbirs \quad kbirsd$$
$$RXpIrs + IRS[0,0,0,P,P,0,0] \rightleftharpoons IRS[R,0,0,P,P,0,0] \quad kbirs \quad kbirsd$$
$$RXpIrs + IRS[0,0,0,P,P,0,P] \rightleftharpoons IRS[R,0,0,P,P,0,P] \quad kbirs \quad kbirsd$$
$$RXpIrs + IRS[0,0,0,P,P,P,0] \rightleftharpoons IRS[R,0,0,P,P,P,0] \quad kbirs \quad kbirsd$$
$$RXpIrs + IRS[0,0,0,P,P,P,P] \rightleftharpoons IRS[R,0,0,P,P,P,P] \quad kbirs \quad kbirsd$$
$$RXpIrs + IRS[0,0,P,0,0,0,0] \rightleftharpoons IRS[R,0,P,0,0,0,0] \quad kbirs \quad kbirsd$$
$$RXpIrs + IRS[0,0,P,0,0,0,P] \rightleftharpoons IRS[R,0,P,0,0,0,P] \quad kbirs \quad kbirsd$$
$$RXpIrs + IRS[0,0,P,0,0,P,0] \rightleftharpoons IRS[R,0,P,0,0,P,0] \quad kbirs \quad kbirsd$$
$$RXpIrs + IRS[0,0,P,0,0,P,P] \rightleftharpoons IRS[R,0,P,0,0,P,P] \quad kbirs \quad kbirsd$$
$$RXpIrs + IRS[0,0,P,0,P,0,0] \rightleftharpoons IRS[R,0,P,0,P,0,0] \quad kbirs \quad kbirsd$$
$$RXpIrs + IRS[0,0,P,0,P,0,P] \rightleftharpoons IRS[R,0,P,0,P,0,P] \quad kbirs \quad kbirsd$$
$$RXpIrs + IRS[0,0,P,0,P,P,0] \rightleftharpoons IRS[R,0,P,0,P,P,0] \quad kbirs \quad kbirsd$$
$$RXpIrs + IRS[0,0,P,0,P,P,P] \rightleftharpoons IRS[R,0,P,0,P,P,P] \quad kbirs \quad kbirsd$$
$$RXpIrs + IRS[0,0,P,P,0,0,0] \rightleftharpoons IRS[R,0,P,P,0,0,0] \quad kbirs \quad kbirsd$$
$$RXpIrs + IRS[0,0,P,P,0,0,P] \rightleftharpoons IRS[R,0,P,P,0,0,P] \quad kbirs \quad kbirsd$$
$$RXpIrs + IRS[0,0,P,P,0,P,0] \rightleftharpoons IRS[R,0,P,P,0,P,0] \quad kbirs \quad kbirsd$$
$$RXpIrs + IRS[0,0,P,P,0,P,P] \rightleftharpoons IRS[R,0,P,P,0,P,P] \quad kbirs \quad kbirsd$$
$$RXpIrs + IRS[0,0,P,P,P,0,0] \rightleftharpoons IRS[R,0,P,P,P,0,0] \quad kbirs \quad kbirsd$$
$$RXpIrs + IRS[0,0,P,P,P,0,P] \rightleftharpoons IRS[R,0,P,P,P,0,P] \quad kbirs \quad kbirsd$$
$$RXpIrs + IRS[0,0,P,P,P,P,0] \rightleftharpoons IRS[R,0,P,P,P,P,0] \quad kbirs \quad kbirsd$$
$$RXpIrs + IRS[0,0,P,P,P,P,P] \rightleftharpoons IRS[R,0,P,P,P,P,P] \quad kbirs \quad kbirsd$$
$$RXpIrs + IRS[0,P,0,0,0,0,0] \rightleftharpoons IRS[R,P,0,0,0,0,0] \quad kbirs \quad kbirsd$$

$$RXpIrs + IRS[0,P,0,0,0,0,P] \leftrightharpoons IRS[R,P,0,0,0,0,P] \quad kbirs \quad kbirsd$$
$$RXpIrs + IRS[0,P,0,0,0,P,0] \leftrightharpoons IRS[R,P,0,0,0,P,0] \quad kbirs \quad kbirsd$$
$$RXpIrs + IRS[0,P,0,0,0,P,P] \leftrightharpoons IRS[R,P,0,0,0,P,P] \quad kbirs \quad kbirsd$$
$$RXpIrs + IRS[0,P,0,0,P,0,0] \leftrightharpoons IRS[R,P,0,0,P,0,0] \quad kbirs \quad kbirsd$$
$$RXpIrs + IRS[0,P,0,0,P,0,P] \leftrightharpoons IRS[R,P,0,0,P,0,P] \quad kbirs \quad kbirsd$$
$$RXpIrs + IRS[0,P,0,0,P,P,0] \leftrightharpoons IRS[R,P,0,0,P,P,0] \quad kbirs \quad kbirsd$$
$$RXpIrs + IRS[0,P,0,0,P,P,P] \leftrightharpoons IRS[R,P,0,0,P,P,P] \quad kbirs \quad kbirsd$$
$$RXpIrs + IRS[0,P,0,P,0,0,0] \leftrightharpoons IRS[R,P,0,P,0,0,0] \quad kbirs \quad kbirsd$$
$$RXpIrs + IRS[0,P,0,P,0,0,P] \leftrightharpoons IRS[R,P,0,P,0,0,P] \quad kbirs \quad kbirsd$$
$$RXpIrs + IRS[0,P,0,P,0,P,0] \leftrightharpoons IRS[R,P,0,P,0,P,0] \quad kbirs \quad kbirsd$$
$$RXpIrs + IRS[0,P,0,P,0,P,P] \leftrightharpoons IRS[R,P,0,P,0,P,P] \quad kbirs \quad kbirsd$$
$$RXpIrs + IRS[0,P,0,P,P,0,0] \leftrightharpoons IRS[R,P,0,P,P,0,0] \quad kbirs \quad kbirsd$$
$$RXpIrs + IRS[0,P,0,P,P,0,P] \leftrightharpoons IRS[R,P,0,P,P,0,P] \quad kbirs \quad kbirsd$$
$$RXpIrs + IRS[0,P,0,P,P,P,0] \leftrightharpoons IRS[R,P,0,P,P,P,0] \quad kbirs \quad kbirsd$$
$$RXpIrs + IRS[0,P,0,P,P,P,P] \leftrightharpoons IRS[R,P,0,P,P,P,P] \quad kbirs \quad kbirsd$$
$$RXpIrs + IRS[0,P,P,0,0,0,0] \leftrightharpoons IRS[R,P,P,0,0,0,0] \quad kbirs \quad kbirsd$$
$$RXpIrs + IRS[0,P,P,0,0,0,P] \leftrightharpoons IRS[R,P,P,0,0,0,P] \quad kbirs \quad kbirsd$$
$$RXpIrs + IRS[0,P,P,0,0,P,0] \leftrightharpoons IRS[R,P,P,0,0,P,0] \quad kbirs \quad kbirsd$$
$$RXpIrs + IRS[0,P,P,0,0,P,P] \leftrightharpoons IRS[R,P,P,0,0,P,P] \quad kbirs \quad kbirsd$$
$$RXpIrs + IRS[0,P,P,0,P,0,0] \leftrightharpoons IRS[R,P,P,0,P,0,0] \quad kbirs \quad kbirsd$$
$$RXpIrs + IRS[0,P,P,0,P,0,P] \leftrightharpoons IRS[R,P,P,0,P,0,P] \quad kbirs \quad kbirsd$$
$$RXpIrs + IRS[0,P,P,0,P,P,0] \leftrightharpoons IRS[R,P,P,0,P,P,0] \quad kbirs \quad kbirsd$$
$$RXpIrs + IRS[0,P,P,0,P,P,P] \leftrightharpoons IRS[R,P,P,0,P,P,P] \quad kbirs \quad kbirsd$$
$$RXpIrs + IRS[0,P,P,P,0,0,0] \leftrightharpoons IRS[R,P,P,P,0,0,0] \quad kbirs \quad kbirsd$$
$$RXpIrs + IRS[0,P,P,P,0,0,P] \leftrightharpoons IRS[R,P,P,P,0,0,P] \quad kbirs \quad kbirsd$$
$$RXpIrs + IRS[0,P,P,P,0,P,0] \leftrightharpoons IRS[R,P,P,P,0,P,0] \quad kbirs \quad kbirsd$$
$$RXpIrs + IRS[0,P,P,P,0,P,P] \leftrightharpoons IRS[R,P,P,P,0,P,P] \quad kbirs \quad kbirsd$$
$$RXpIrs + IRS[0,P,P,P,P,0,0] \leftrightharpoons IRS[R,P,P,P,P,0,0] \quad kbirs \quad kbirsd$$
$$RXpIrs + IRS[0,P,P,P,P,0,P] \leftrightharpoons IRS[R,P,P,P,P,0,P] \quad kbirs \quad kbirsd$$
$$RXpIrs + IRS[0,P,P,P,P,P,0] \leftrightharpoons IRS[R,P,P,P,P,P,0] \quad kbirs \quad kbirsd$$
$$RXpIrs + IRS[0,P,P,P,P,P,P] \leftrightharpoons IRS[R,P,P,P,P,P,P] \quad kbirs \quad kbirsd$$
$$IRS[0,0,0,0,0,0,0] \leftrightharpoons IRS[0,P,0,0,0,0,0] \quad kirsp1 \quad kirsp1d$$
$$IRS[0,0,0,0,0,0,P] \leftrightharpoons IRS[0,P,0,0,0,0,P] \quad kirsp1 \quad kirsp1d$$
$$IRS[0,0,0,0,0,P,0] \leftrightharpoons IRS[0,P,0,0,0,P,0] \quad kirsp1 \quad kirsp1d$$
$$IRS[0,0,0,0,0,P,P] \leftrightharpoons IRS[0,P,0,0,0,P,P] \quad kirsp1 \quad kirsp1d$$
$$IRS[0,0,0,0,P,0,0] \leftrightharpoons IRS[0,P,0,0,P,0,0] \quad kirsp1 \quad kirsp1d$$
$$IRS[0,0,0,0,P,0,P] \leftrightharpoons IRS[0,P,0,0,P,0,P] \quad kirsp1 \quad kirsp1d$$
$$IRS[0,0,0,0,P,P,0] \leftrightharpoons IRS[0,P,0,0,P,P,0] \quad kirsp1 \quad kirsp1d$$
$$IRS[0,0,0,0,P,P,P] \leftrightharpoons IRS[0,P,0,0,P,P,P] \quad kirsp1 \quad kirsp1d$$
$$IRS[0,0,0,P,0,0,0] \leftrightharpoons IRS[0,P,0,P,0,0,0] \quad kirsp1 \quad kirsp1d$$
$$IRS[0,0,0,P,0,0,P] \leftrightharpoons IRS[0,P,0,P,0,0,P] \quad kirsp1 \quad kirsp1d$$
$$IRS[0,0,0,P,0,P,0] \leftrightharpoons IRS[0,P,0,P,0,P,0] \quad kirsp1 \quad kirsp1d$$
$$IRS[0,0,0,P,0,P,P] \leftrightharpoons IRS[0,P,0,P,0,P,P] \quad kirsp1 \quad kirsp1d$$
$$IRS[0,0,0,P,P,0,0] \leftrightharpoons IRS[0,P,0,P,P,0,0] \quad kirsp1 \quad kirsp1d$$
$$IRS[0,0,0,P,P,0,P] \leftrightharpoons IRS[0,P,0,P,P,0,P] \quad kirsp1 \quad kirsp1d$$

$$IRS[0,0,0,P,P,P,0] \rightleftharpoons IRS[0,P,0,P,P,P,0] \quad kirsp1 \quad kirsp1d$$
$$IRS[0,0,0,P,P,P,P] \rightleftharpoons IRS[0,P,0,P,P,P,P] \quad kirsp1 \quad kirsp1d$$
$$IRS[0,0,P,0,0,0,0] \rightleftharpoons IRS[0,P,P,0,0,0,0] \quad kirsp1 \quad kirsp1d$$
$$IRS[0,0,P,0,0,0,P] \rightleftharpoons IRS[0,P,P,0,0,0,P] \quad kirsp1 \quad kirsp1d$$
$$IRS[0,0,P,0,0,P,0] \rightleftharpoons IRS[0,P,P,0,0,P,0] \quad kirsp1 \quad kirsp1d$$
$$IRS[0,0,P,0,0,P,P] \rightleftharpoons IRS[0,P,P,0,0,P,P] \quad kirsp1 \quad kirsp1d$$
$$IRS[0,0,P,0,P,0,0] \rightleftharpoons IRS[0,P,P,0,P,0,0] \quad kirsp1 \quad kirsp1d$$
$$IRS[0,0,P,0,P,0,P] \rightleftharpoons IRS[0,P,P,0,P,0,P] \quad kirsp1 \quad kirsp1d$$
$$IRS[0,0,P,0,P,P,0] \rightleftharpoons IRS[0,P,P,0,P,P,0] \quad kirsp1 \quad kirsp1d$$
$$IRS[0,0,P,0,P,P,P] \rightleftharpoons IRS[0,P,P,0,P,P,P] \quad kirsp1 \quad kirsp1d$$
$$IRS[0,0,P,P,0,0,0] \rightleftharpoons IRS[0,P,P,P,0,0,0] \quad kirsp1 \quad kirsp1d$$
$$IRS[0,0,P,P,0,0,P] \rightleftharpoons IRS[0,P,P,P,0,0,P] \quad kirsp1 \quad kirsp1d$$
$$IRS[0,0,P,P,0,P,0] \rightleftharpoons IRS[0,P,P,P,0,P,0] \quad kirsp1 \quad kirsp1d$$
$$IRS[0,0,P,P,0,P,P] \rightleftharpoons IRS[0,P,P,P,0,P,P] \quad kirsp1 \quad kirsp1d$$
$$IRS[0,0,P,P,P,0,0] \rightleftharpoons IRS[0,P,P,P,P,0,0] \quad kirsp1 \quad kirsp1d$$
$$IRS[0,0,P,P,P,0,P] \rightleftharpoons IRS[0,P,P,P,P,0,P] \quad kirsp1 \quad kirsp1d$$
$$IRS[0,0,P,P,P,P,0] \rightleftharpoons IRS[0,P,P,P,P,P,0] \quad kirsp1 \quad kirsp1d$$
$$IRS[0,0,P,P,P,P,P] \rightleftharpoons IRS[0,P,P,P,P,P,P] \quad kirsp1 \quad kirsp1d$$
$$IRS[0,0,0,0,0,0,0] \rightleftharpoons IRS[0,0,P,0,0,0,0] \quad kirsp2 \quad kirsp2d$$
$$IRS[0,0,0,0,0,0,P] \rightleftharpoons IRS[0,0,P,0,0,0,P] \quad kirsp2 \quad kirsp2d$$
$$IRS[0,0,0,0,0,P,0] \rightleftharpoons IRS[0,0,P,0,0,P,0] \quad kirsp2 \quad kirsp2d$$
$$IRS[0,0,0,0,0,P,P] \rightleftharpoons IRS[0,0,P,0,0,P,P] \quad kirsp2 \quad kirsp2d$$
$$IRS[0,0,0,0,P,0,0] \rightleftharpoons IRS[0,0,P,0,P,0,0] \quad kirsp2 \quad kirsp2d$$
$$IRS[0,0,0,0,P,0,P] \rightleftharpoons IRS[0,0,P,0,P,0,P] \quad kirsp2 \quad kirsp2d$$
$$IRS[0,0,0,0,P,P,0] \rightleftharpoons IRS[0,0,P,0,P,P,0] \quad kirsp2 \quad kirsp2d$$
$$IRS[0,0,0,0,P,P,P] \rightleftharpoons IRS[0,0,P,0,P,P,P] \quad kirsp2 \quad kirsp2d$$
$$IRS[0,0,0,P,0,0,0] \rightleftharpoons IRS[0,0,P,P,0,0,0] \quad kirsp2 \quad kirsp2d$$
$$IRS[0,0,0,P,0,0,P] \rightleftharpoons IRS[0,0,P,P,0,0,P] \quad kirsp2 \quad kirsp2d$$
$$IRS[0,0,0,P,0,P,0] \rightleftharpoons IRS[0,0,P,P,0,P,0] \quad kirsp2 \quad kirsp2d$$
$$IRS[0,0,0,P,0,P,P] \rightleftharpoons IRS[0,0,P,P,0,P,P] \quad kirsp2 \quad kirsp2d$$
$$IRS[0,0,0,P,P,0,0] \rightleftharpoons IRS[0,0,P,P,P,0,0] \quad kirsp2 \quad kirsp2d$$
$$IRS[0,0,0,P,P,0,P] \rightleftharpoons IRS[0,0,P,P,P,0,P] \quad kirsp2 \quad kirsp2d$$
$$IRS[0,0,0,P,P,P,0] \rightleftharpoons IRS[0,0,P,P,P,P,0] \quad kirsp2 \quad kirsp2d$$
$$IRS[0,0,0,P,P,P,P] \rightleftharpoons IRS[0,0,P,P,P,P,P] \quad kirsp2 \quad kirsp2d$$
$$IRS[0,P,0,0,0,0,0] \rightleftharpoons IRS[0,P,P,0,0,0,0] \quad kirsp2 \quad kirsp2d$$
$$IRS[0,P,0,0,0,0,P] \rightleftharpoons IRS[0,P,P,0,0,0,P] \quad kirsp2 \quad kirsp2d$$
$$IRS[0,P,0,0,0,P,0] \rightleftharpoons IRS[0,P,P,0,0,P,0] \quad kirsp2 \quad kirsp2d$$
$$IRS[0,P,0,0,0,P,P] \rightleftharpoons IRS[0,P,P,0,0,P,P] \quad kirsp2 \quad kirsp2d$$
$$IRS[0,P,0,0,P,0,0] \rightleftharpoons IRS[0,P,P,0,P,0,0] \quad kirsp2 \quad kirsp2d$$
$$IRS[0,P,0,0,P,0,P] \rightleftharpoons IRS[0,P,P,0,P,0,P] \quad kirsp2 \quad kirsp2d$$
$$IRS[0,P,0,0,P,P,0] \rightleftharpoons IRS[0,P,P,0,P,P,0] \quad kirsp2 \quad kirsp2d$$
$$IRS[0,P,0,0,P,P,P] \rightleftharpoons IRS[0,P,P,0,P,P,P] \quad kirsp2 \quad kirsp2d$$
$$IRS[0,P,0,P,0,0,0] \rightleftharpoons IRS[0,P,P,P,0,0,0] \quad kirsp2 \quad kirsp2d$$
$$IRS[0,P,0,P,0,0,P] \rightleftharpoons IRS[0,P,P,P,0,0,P] \quad kirsp2 \quad kirsp2d$$
$$IRS[0,P,0,P,0,P,0] \rightleftharpoons IRS[0,P,P,P,0,P,0] \quad kirsp2 \quad kirsp2d$$

$$IRS[0,P,0,P,0,P,P] \risingdotseq IRS[0,P,P,P,0,P,P] \quad kirsp2 \quad kirsp2d$$
$$IRS[0,P,0,P,P,0,0] \risingdotseq IRS[0,P,P,P,P,0,0] \quad kirsp2 \quad kirsp2d$$
$$IRS[0,P,0,P,P,0,P] \risingdotseq IRS[0,P,P,P,P,0,P] \quad kirsp2 \quad kirsp2d$$
$$IRS[0,P,0,P,P,P,0] \risingdotseq IRS[0,P,P,P,P,P,0] \quad kirsp2 \quad kirsp2d$$
$$IRS[0,P,0,P,P,P,P] \risingdotseq IRS[0,P,P,P,P,P,P] \quad kirsp2 \quad kirsp2d$$
$$IRS[0,0,0,0,0,0,0] \risingdotseq IRS[0,0,0,P,0,0,0] \quad kirsp3 \quad kirsp3d$$
$$IRS[0,0,0,0,0,0,P] \risingdotseq IRS[0,0,0,P,0,0,P] \quad kirsp3 \quad kirsp3d$$
$$IRS[0,0,0,0,0,P,0] \risingdotseq IRS[0,0,0,P,0,P,0] \quad kirsp3 \quad kirsp3d$$
$$IRS[0,0,0,0,0,P,P] \risingdotseq IRS[0,0,0,P,0,P,P] \quad kirsp3 \quad kirsp3d$$
$$IRS[0,0,0,0,P,0,0] \risingdotseq IRS[0,0,0,P,P,0,0] \quad kirsp3 \quad kirsp3d$$
$$IRS[0,0,0,0,P,0,P] \risingdotseq IRS[0,0,0,P,P,0,P] \quad kirsp3 \quad kirsp3d$$
$$IRS[0,0,0,0,P,P,0] \risingdotseq IRS[0,0,0,P,P,P,0] \quad kirsp3 \quad kirsp3d$$
$$IRS[0,0,0,0,P,P,P] \risingdotseq IRS[0,0,0,P,P,P,P] \quad kirsp3 \quad kirsp3d$$
$$IRS[0,0,P,0,0,0,0] \risingdotseq IRS[0,0,P,P,0,0,0] \quad kirsp3 \quad kirsp3d$$
$$IRS[0,0,P,0,0,0,P] \risingdotseq IRS[0,0,P,P,0,0,P] \quad kirsp3 \quad kirsp3d$$
$$IRS[0,0,P,0,0,P,0] \risingdotseq IRS[0,0,P,P,0,P,0] \quad kirsp3 \quad kirsp3d$$
$$IRS[0,0,P,0,0,P,P] \risingdotseq IRS[0,0,P,P,0,P,P] \quad kirsp3 \quad kirsp3d$$
$$IRS[0,0,P,0,P,0,0] \risingdotseq IRS[0,0,P,P,P,0,0] \quad kirsp3 \quad kirsp3d$$
$$IRS[0,0,P,0,P,0,P] \risingdotseq IRS[0,0,P,P,P,0,P] \quad kirsp3 \quad kirsp3d$$
$$IRS[0,0,P,0,P,P,0] \risingdotseq IRS[0,0,P,P,P,P,0] \quad kirsp3 \quad kirsp3d$$
$$IRS[0,0,P,0,P,P,P] \risingdotseq IRS[0,0,P,P,P,P,P] \quad kirsp3 \quad kirsp3d$$
$$IRS[0,P,0,0,0,0,0] \risingdotseq IRS[0,P,0,P,0,0,0] \quad kirsp3 \quad kirsp3d$$
$$IRS[0,P,0,0,0,0,P] \risingdotseq IRS[0,P,0,P,0,0,P] \quad kirsp3 \quad kirsp3d$$
$$IRS[0,P,0,0,0,P,0] \risingdotseq IRS[0,P,0,P,0,P,0] \quad kirsp3 \quad kirsp3d$$
$$IRS[0,P,0,0,0,P,P] \risingdotseq IRS[0,P,0,P,0,P,P] \quad kirsp3 \quad kirsp3d$$
$$IRS[0,P,0,0,P,0,0] \risingdotseq IRS[0,P,0,P,P,0,0] \quad kirsp3 \quad kirsp3d$$
$$IRS[0,P,0,0,P,0,P] \risingdotseq IRS[0,P,0,P,P,0,P] \quad kirsp3 \quad kirsp3d$$
$$IRS[0,P,0,0,P,P,0] \risingdotseq IRS[0,P,0,P,P,P,0] \quad kirsp3 \quad kirsp3d$$
$$IRS[0,P,0,0,P,P,P] \risingdotseq IRS[0,P,0,P,P,P,P] \quad kirsp3 \quad kirsp3d$$
$$IRS[0,P,P,0,0,0,0] \risingdotseq IRS[0,P,P,P,0,0,0] \quad kirsp3 \quad kirsp3d$$
$$IRS[0,P,P,0,0,0,P] \risingdotseq IRS[0,P,P,P,0,0,P] \quad kirsp3 \quad kirsp3d$$
$$IRS[0,P,P,0,0,P,0] \risingdotseq IRS[0,P,P,P,0,P,0] \quad kirsp3 \quad kirsp3d$$
$$IRS[0,P,P,0,0,P,P] \risingdotseq IRS[0,P,P,P,0,P,P] \quad kirsp3 \quad kirsp3d$$
$$IRS[0,P,P,0,P,0,0] \risingdotseq IRS[0,P,P,P,P,0,0] \quad kirsp3 \quad kirsp3d$$
$$IRS[0,P,P,0,P,0,P] \risingdotseq IRS[0,P,P,P,P,0,P] \quad kirsp3 \quad kirsp3d$$
$$IRS[0,P,P,0,P,P,0] \risingdotseq IRS[0,P,P,P,P,P,0] \quad kirsp3 \quad kirsp3d$$
$$IRS[0,P,P,0,P,P,P] \risingdotseq IRS[0,P,P,P,P,P,P] \quad kirsp3 \quad kirsp3d$$
$$IRS[0,0,0,0,0,0,0] \risingdotseq IRS[0,0,0,0,P,0,0] \quad kirsp4 \quad kirsp4d$$
$$IRS[0,0,0,0,0,0,P] \risingdotseq IRS[0,0,0,0,P,0,P] \quad kirsp4 \quad kirsp4d$$
$$IRS[0,0,0,0,0,P,0] \risingdotseq IRS[0,0,0,0,P,P,0] \quad kirsp4 \quad kirsp4d$$
$$IRS[0,0,0,0,0,P,P] \risingdotseq IRS[0,0,0,0,P,P,P] \quad kirsp4 \quad kirsp4d$$
$$IRS[0,0,0,P,0,0,0] \risingdotseq IRS[0,0,0,P,P,0,0] \quad kirsp4 \quad kirsp4d$$
$$IRS[0,0,0,P,0,0,P] \risingdotseq IRS[0,0,0,P,P,0,P] \quad kirsp4 \quad kirsp4d$$
$$IRS[0,0,0,P,0,P,0] \risingdotseq IRS[0,0,0,P,P,P,0] \quad kirsp4 \quad kirsp4d$$
$$IRS[0,0,0,P,0,P,P] \risingdotseq IRS[0,0,0,P,P,P,P] \quad kirsp4 \quad kirsp4d$$

$$IRS[0,0,P,0,0,0,0] \leftrightharpoons IRS[0,0,P,0,P,0,0] \quad kirsp4 \quad kirsp4d$$
$$IRS[0,0,P,0,0,0,P] \leftrightharpoons IRS[0,0,P,0,P,0,P] \quad kirsp4 \quad kirsp4d$$
$$IRS[0,0,P,0,0,P,0] \leftrightharpoons IRS[0,0,P,0,P,P,0] \quad kirsp4 \quad kirsp4d$$
$$IRS[0,0,P,0,0,P,P] \leftrightharpoons IRS[0,0,P,0,P,P,P] \quad kirsp4 \quad kirsp4d$$
$$IRS[0,0,P,P,0,0,0] \leftrightharpoons IRS[0,0,P,P,P,0,0] \quad kirsp4 \quad kirsp4d$$
$$IRS[0,0,P,P,0,0,P] \leftrightharpoons IRS[0,0,P,P,P,0,P] \quad kirsp4 \quad kirsp4d$$
$$IRS[0,0,P,P,0,P,0] \leftrightharpoons IRS[0,0,P,P,P,P,0] \quad kirsp4 \quad kirsp4d$$
$$IRS[0,0,P,P,0,P,P] \leftrightharpoons IRS[0,0,P,P,P,P,P] \quad kirsp4 \quad kirsp4d$$
$$IRS[0,P,0,0,0,0,0] \leftrightharpoons IRS[0,P,0,0,P,0,0] \quad kirsp4 \quad kirsp4d$$
$$IRS[0,P,0,0,0,0,P] \leftrightharpoons IRS[0,P,0,0,P,0,P] \quad kirsp4 \quad kirsp4d$$
$$IRS[0,P,0,0,0,P,0] \leftrightharpoons IRS[0,P,0,0,P,P,0] \quad kirsp4 \quad kirsp4d$$
$$IRS[0,P,0,0,0,P,P] \leftrightharpoons IRS[0,P,0,0,P,P,P] \quad kirsp4 \quad kirsp4d$$
$$IRS[0,P,0,P,0,0,0] \leftrightharpoons IRS[0,P,0,P,P,0,0] \quad kirsp4 \quad kirsp4d$$
$$IRS[0,P,0,P,0,0,P] \leftrightharpoons IRS[0,P,0,P,P,0,P] \quad kirsp4 \quad kirsp4d$$
$$IRS[0,P,0,P,0,P,0] \leftrightharpoons IRS[0,P,0,P,P,P,0] \quad kirsp4 \quad kirsp4d$$
$$IRS[0,P,0,P,0,P,P] \leftrightharpoons IRS[0,P,0,P,P,P,P] \quad kirsp4 \quad kirsp4d$$
$$IRS[0,P,P,0,0,0,0] \leftrightharpoons IRS[0,P,P,0,P,0,0] \quad kirsp4 \quad kirsp4d$$
$$IRS[0,P,P,0,0,0,P] \leftrightharpoons IRS[0,P,P,0,P,0,P] \quad kirsp4 \quad kirsp4d$$
$$IRS[0,P,P,0,0,P,0] \leftrightharpoons IRS[0,P,P,0,P,P,0] \quad kirsp4 \quad kirsp4d$$
$$IRS[0,P,P,0,0,P,P] \leftrightharpoons IRS[0,P,P,0,P,P,P] \quad kirsp4 \quad kirsp4d$$
$$IRS[0,P,P,P,0,0,0] \leftrightharpoons IRS[0,P,P,P,P,0,0] \quad kirsp4 \quad kirsp4d$$
$$IRS[0,P,P,P,0,0,P] \leftrightharpoons IRS[0,P,P,P,P,0,P] \quad kirsp4 \quad kirsp4d$$
$$IRS[0,P,P,P,0,P,0] \leftrightharpoons IRS[0,P,P,P,P,P,0] \quad kirsp4 \quad kirsp4d$$
$$IRS[0,P,P,P,0,P,P] \leftrightharpoons IRS[0,P,P,P,P,P,P] \quad kirsp4 \quad kirsp4d$$
$$IRS[0,0,0,0,0,0,0] \leftrightharpoons IRS[0,0,0,0,0,P,0] \quad kirsp5 \quad kirsp5d$$
$$IRS[0,0,0,0,0,0,P] \leftrightharpoons IRS[0,0,0,0,0,P,P] \quad kirsp5 \quad kirsp5d$$
$$IRS[0,0,0,0,P,0,0] \leftrightharpoons IRS[0,0,0,0,P,P,0] \quad kirsp5 \quad kirsp5d$$
$$IRS[0,0,0,0,P,0,P] \leftrightharpoons IRS[0,0,0,0,P,P,P] \quad kirsp5 \quad kirsp5d$$
$$IRS[0,0,0,P,0,0,0] \leftrightharpoons IRS[0,0,0,P,0,P,0] \quad kirsp5 \quad kirsp5d$$
$$IRS[0,0,0,P,0,0,P] \leftrightharpoons IRS[0,0,0,P,0,P,P] \quad kirsp5 \quad kirsp5d$$
$$IRS[0,0,0,P,P,0,0] \leftrightharpoons IRS[0,0,0,P,P,P,0] \quad kirsp5 \quad kirsp5d$$
$$IRS[0,0,0,P,P,0,P] \leftrightharpoons IRS[0,0,0,P,P,P,P] \quad kirsp5 \quad kirsp5d$$
$$IRS[0,0,P,0,0,0,0] \leftrightharpoons IRS[0,0,P,0,0,P,0] \quad kirsp5 \quad kirsp5d$$
$$IRS[0,0,P,0,0,0,P] \leftrightharpoons IRS[0,0,P,0,0,P,P] \quad kirsp5 \quad kirsp5d$$
$$IRS[0,0,P,0,P,0,0] \leftrightharpoons IRS[0,0,P,0,P,P,0] \quad kirsp5 \quad kirsp5d$$
$$IRS[0,0,P,0,P,0,P] \leftrightharpoons IRS[0,0,P,0,P,P,P] \quad kirsp5 \quad kirsp5d$$
$$IRS[0,0,P,P,0,0,0] \leftrightharpoons IRS[0,0,P,P,0,P,0] \quad kirsp5 \quad kirsp5d$$
$$IRS[0,0,P,P,0,0,P] \leftrightharpoons IRS[0,0,P,P,0,P,P] \quad kirsp5 \quad kirsp5d$$
$$IRS[0,0,P,P,P,0,0] \leftrightharpoons IRS[0,0,P,P,P,P,0] \quad kirsp5 \quad kirsp5d$$
$$IRS[0,0,P,P,P,0,P] \leftrightharpoons IRS[0,0,P,P,P,P,P] \quad kirsp5 \quad kirsp5d$$
$$IRS[0,P,0,0,0,0,0] \leftrightharpoons IRS[0,P,0,0,0,P,0] \quad kirsp5 \quad kirsp5d$$
$$IRS[0,P,0,0,0,0,P] \leftrightharpoons IRS[0,P,0,0,0,P,P] \quad kirsp5 \quad kirsp5d$$
$$IRS[0,P,0,0,P,0,0] \leftrightharpoons IRS[0,P,0,0,P,P,0] \quad kirsp5 \quad kirsp5d$$
$$IRS[0,P,0,0,P,0,P] \leftrightharpoons IRS[0,P,0,0,P,P,P] \quad kirsp5 \quad kirsp5d$$
$$IRS[0,P,0,P,0,0,0] \leftrightharpoons IRS[0,P,0,P,0,P,0] \quad kirsp5 \quad kirsp5d$$

$$IRS[0,P,0,P,0,0,P] \rightleftharpoons IRS[0,P,0,P,0,P,P] \quad kirsp5 \quad kirsp5d$$
$$IRS[0,P,0,P,P,0,0] \rightleftharpoons IRS[0,P,0,P,P,P,0] \quad kirsp5 \quad kirsp5d$$
$$IRS[0,P,0,P,P,0,P] \rightleftharpoons IRS[0,P,0,P,P,P,P] \quad kirsp5 \quad kirsp5d$$
$$IRS[0,P,P,0,0,0,0] \rightleftharpoons IRS[0,P,P,0,0,P,0] \quad kirsp5 \quad kirsp5d$$
$$IRS[0,P,P,0,0,0,P] \rightleftharpoons IRS[0,P,P,0,0,P,P] \quad kirsp5 \quad kirsp5d$$
$$IRS[0,P,P,0,P,0,0] \rightleftharpoons IRS[0,P,P,0,P,P,0] \quad kirsp5 \quad kirsp5d$$
$$IRS[0,P,P,0,P,0,P] \rightleftharpoons IRS[0,P,P,0,P,P,P] \quad kirsp5 \quad kirsp5d$$
$$IRS[0,P,P,P,0,0,0] \rightleftharpoons IRS[0,P,P,P,0,P,0] \quad kirsp5 \quad kirsp5d$$
$$IRS[0,P,P,P,0,0,P] \rightleftharpoons IRS[0,P,P,P,0,P,P] \quad kirsp5 \quad kirsp5d$$
$$IRS[0,P,P,P,P,0,0] \rightleftharpoons IRS[0,P,P,P,P,P,0] \quad kirsp5 \quad kirsp5d$$
$$IRS[0,P,P,P,P,0,P] \rightleftharpoons IRS[0,P,P,P,P,P,P] \quad kirsp5 \quad kirsp5d$$
$$IRS[0,0,0,0,0,0,0] \rightleftharpoons IRS[0,0,0,0,0,0,P] \quad kirsp6 \quad kirsp6d$$
$$IRS[0,0,0,0,0,P,0] \rightleftharpoons IRS[0,0,0,0,0,P,P] \quad kirsp6 \quad kirsp6d$$
$$IRS[0,0,0,0,P,0,0] \rightleftharpoons IRS[0,0,0,0,P,0,P] \quad kirsp6 \quad kirsp6d$$
$$IRS[0,0,0,0,P,P,0] \rightleftharpoons IRS[0,0,0,0,P,P,P] \quad kirsp6 \quad kirsp6d$$
$$IRS[0,0,0,P,0,0,0] \rightleftharpoons IRS[0,0,0,P,0,0,P] \quad kirsp6 \quad kirsp6d$$
$$IRS[0,0,0,P,0,P,0] \rightleftharpoons IRS[0,0,0,P,0,P,P] \quad kirsp6 \quad kirsp6d$$
$$IRS[0,0,0,P,P,0,0] \rightleftharpoons IRS[0,0,0,P,P,0,P] \quad kirsp6 \quad kirsp6d$$
$$IRS[0,0,0,P,P,P,0] \rightleftharpoons IRS[0,0,0,P,P,P,P] \quad kirsp6 \quad kirsp6d$$
$$IRS[0,0,P,0,0,0,0] \rightleftharpoons IRS[0,0,P,0,0,0,P] \quad kirsp6 \quad kirsp6d$$
$$IRS[0,0,P,0,0,P,0] \rightleftharpoons IRS[0,0,P,0,0,P,P] \quad kirsp6 \quad kirsp6d$$
$$IRS[0,0,P,0,P,0,0] \rightleftharpoons IRS[0,0,P,0,P,0,P] \quad kirsp6 \quad kirsp6d$$
$$IRS[0,0,P,0,P,P,0] \rightleftharpoons IRS[0,0,P,0,P,P,P] \quad kirsp6 \quad kirsp6d$$
$$IRS[0,0,P,P,0,0,0] \rightleftharpoons IRS[0,0,P,P,0,0,P] \quad kirsp6 \quad kirsp6d$$
$$IRS[0,0,P,P,0,P,0] \rightleftharpoons IRS[0,0,P,P,0,P,P] \quad kirsp6 \quad kirsp6d$$
$$IRS[0,0,P,P,P,0,0] \rightleftharpoons IRS[0,0,P,P,P,0,P] \quad kirsp6 \quad kirsp6d$$
$$IRS[0,0,P,P,P,P,0] \rightleftharpoons IRS[0,0,P,P,P,P,P] \quad kirsp6 \quad kirsp6d$$
$$IRS[0,P,0,0,0,0,0] \rightleftharpoons IRS[0,P,0,0,0,0,P] \quad kirsp6 \quad kirsp6d$$
$$IRS[0,P,0,0,0,P,0] \rightleftharpoons IRS[0,P,0,0,0,P,P] \quad kirsp6 \quad kirsp6d$$
$$IRS[0,P,0,0,P,0,0] \rightleftharpoons IRS[0,P,0,0,P,0,P] \quad kirsp6 \quad kirsp6d$$
$$IRS[0,P,0,0,P,P,0] \rightleftharpoons IRS[0,P,0,0,P,P,P] \quad kirsp6 \quad kirsp6d$$
$$IRS[0,P,0,P,0,0,0] \rightleftharpoons IRS[0,P,0,P,0,0,P] \quad kirsp6 \quad kirsp6d$$
$$IRS[0,P,0,P,0,P,0] \rightleftharpoons IRS[0,P,0,P,0,P,P] \quad kirsp6 \quad kirsp6d$$
$$IRS[0,P,0,P,P,0,0] \rightleftharpoons IRS[0,P,0,P,P,0,P] \quad kirsp6 \quad kirsp6d$$
$$IRS[0,P,0,P,P,P,0] \rightleftharpoons IRS[0,P,0,P,P,P,P] \quad kirsp6 \quad kirsp6d$$
$$IRS[0,P,P,0,0,0,0] \rightleftharpoons IRS[0,P,P,0,0,0,P] \quad kirsp6 \quad kirsp6d$$
$$IRS[0,P,P,0,0,P,0] \rightleftharpoons IRS[0,P,P,0,0,P,P] \quad kirsp6 \quad kirsp6d$$
$$IRS[0,P,P,0,P,0,0] \rightleftharpoons IRS[0,P,P,0,P,0,P] \quad kirsp6 \quad kirsp6d$$
$$IRS[0,P,P,0,P,P,0] \rightleftharpoons IRS[0,P,P,0,P,P,P] \quad kirsp6 \quad kirsp6d$$
$$IRS[0,P,P,P,0,0,0] \rightleftharpoons IRS[0,P,P,P,0,0,P] \quad kirsp6 \quad kirsp6d$$
$$IRS[0,P,P,P,0,P,0] \rightleftharpoons IRS[0,P,P,P,0,P,P] \quad kirsp6 \quad kirsp6d$$
$$IRS[0,P,P,P,P,0,0] \rightleftharpoons IRS[0,P,P,P,P,0,P] \quad kirsp6 \quad kirsp6d$$
$$IRS[0,P,P,P,P,P,0] \rightleftharpoons IRS[0,P,P,P,P,P,P] \quad kirsp6 \quad kirsp6d$$
$$IRS[R,0,0,0,0,0,0] \rightleftharpoons IRS[R,P,0,0,0,0,0] \quad kirsbp1 \quad kirsbp1d$$
$$IRS[R,0,0,0,0,0,P] \rightleftharpoons IRS[R,P,0,0,0,0,P] \quad kirsbp1 \quad kirsbp1d$$

$$IRS[R,0,0,0,0,P,0] \leftrightharpoons IRS[R,P,0,0,0,P,0] \quad kirsbp1 \quad kirsbp1d$$
$$IRS[R,0,0,0,0,P,P] \leftrightharpoons IRS[R,P,0,0,0,P,P] \quad kirsbp1 \quad kirsbp1d$$
$$IRS[R,0,0,0,P,0,0] \leftrightharpoons IRS[R,P,0,0,P,0,0] \quad kirsbp1 \quad kirsbp1d$$
$$IRS[R,0,0,0,P,0,P] \leftrightharpoons IRS[R,P,0,0,P,0,P] \quad kirsbp1 \quad kirsbp1d$$
$$IRS[R,0,0,0,P,P,0] \leftrightharpoons IRS[R,P,0,0,P,P,0] \quad kirsbp1 \quad kirsbp1d$$
$$IRS[R,0,0,0,P,P,P] \leftrightharpoons IRS[R,P,0,0,P,P,P] \quad kirsbp1 \quad kirsbp1d$$
$$IRS[R,0,0,P,0,0,0] \leftrightharpoons IRS[R,P,0,P,0,0,0] \quad kirsbp1 \quad kirsbp1d$$
$$IRS[R,0,0,P,0,0,P] \leftrightharpoons IRS[R,P,0,P,0,0,P] \quad kirsbp1 \quad kirsbp1d$$
$$IRS[R,0,0,P,0,P,0] \leftrightharpoons IRS[R,P,0,P,0,P,0] \quad kirsbp1 \quad kirsbp1d$$
$$IRS[R,0,0,P,0,P,P] \leftrightharpoons IRS[R,P,0,P,0,P,P] \quad kirsbp1 \quad kirsbp1d$$
$$IRS[R,0,0,P,P,0,0] \leftrightharpoons IRS[R,P,0,P,P,0,0] \quad kirsbp1 \quad kirsbp1d$$
$$IRS[R,0,0,P,P,0,P] \leftrightharpoons IRS[R,P,0,P,P,0,P] \quad kirsbp1 \quad kirsbp1d$$
$$IRS[R,0,0,P,P,P,0] \leftrightharpoons IRS[R,P,0,P,P,P,0] \quad kirsbp1 \quad kirsbp1d$$
$$IRS[R,0,0,P,P,P,P] \leftrightharpoons IRS[R,P,0,P,P,P,P] \quad kirsbp1 \quad kirsbp1d$$
$$IRS[R,0,P,0,0,0,0] \leftrightharpoons IRS[R,P,P,0,0,0,0] \quad kirsbp1 \quad kirsbp1d$$
$$IRS[R,0,P,0,0,0,P] \leftrightharpoons IRS[R,P,P,0,0,0,P] \quad kirsbp1 \quad kirsbp1d$$
$$IRS[R,0,P,0,0,P,0] \leftrightharpoons IRS[R,P,P,0,0,P,0] \quad kirsbp1 \quad kirsbp1d$$
$$IRS[R,0,P,0,0,P,P] \leftrightharpoons IRS[R,P,P,0,0,P,P] \quad kirsbp1 \quad kirsbp1d$$
$$IRS[R,0,P,0,P,0,0] \leftrightharpoons IRS[R,P,P,0,P,0,0] \quad kirsbp1 \quad kirsbp1d$$
$$IRS[R,0,P,0,P,0,P] \leftrightharpoons IRS[R,P,P,0,P,0,P] \quad kirsbp1 \quad kirsbp1d$$
$$IRS[R,0,P,0,P,P,0] \leftrightharpoons IRS[R,P,P,0,P,P,0] \quad kirsbp1 \quad kirsbp1d$$
$$IRS[R,0,P,0,P,P,P] \leftrightharpoons IRS[R,P,P,0,P,P,P] \quad kirsbp1 \quad kirsbp1d$$
$$IRS[R,0,P,P,0,0,0] \leftrightharpoons IRS[R,P,P,P,0,0,0] \quad kirsbp1 \quad kirsbp1d$$
$$IRS[R,0,P,P,0,0,P] \leftrightharpoons IRS[R,P,P,P,0,0,P] \quad kirsbp1 \quad kirsbp1d$$
$$IRS[R,0,P,P,0,P,0] \leftrightharpoons IRS[R,P,P,P,0,P,0] \quad kirsbp1 \quad kirsbp1d$$
$$IRS[R,0,P,P,0,P,P] \leftrightharpoons IRS[R,P,P,P,0,P,P] \quad kirsbp1 \quad kirsbp1d$$
$$IRS[R,0,P,P,P,0,0] \leftrightharpoons IRS[R,P,P,P,P,0,0] \quad kirsbp1 \quad kirsbp1d$$
$$IRS[R,0,P,P,P,0,P] \leftrightharpoons IRS[R,P,P,P,P,0,P] \quad kirsbp1 \quad kirsbp1d$$
$$IRS[R,0,P,P,P,P,0] \leftrightharpoons IRS[R,P,P,P,P,P,0] \quad kirsbp1 \quad kirsbp1d$$
$$IRS[R,0,P,P,P,P,P] \leftrightharpoons IRS[R,P,P,P,P,P,P] \quad kirsbp1 \quad kirsbp1d$$
$$IRS[R,0,0,0,0,0,0] \leftrightharpoons IRS[R,0,P,0,0,0,0] \quad kirsbp2 \quad kirsbp2d$$
$$IRS[R,0,0,0,0,0,P] \leftrightharpoons IRS[R,0,P,0,0,0,P] \quad kirsbp2 \quad kirsbp2d$$
$$IRS[R,0,0,0,0,P,0] \leftrightharpoons IRS[R,0,P,0,0,P,0] \quad kirsbp2 \quad kirsbp2d$$
$$IRS[R,0,0,0,0,P,P] \leftrightharpoons IRS[R,0,P,0,0,P,P] \quad kirsbp2 \quad kirsbp2d$$
$$IRS[R,0,0,0,P,0,0] \leftrightharpoons IRS[R,0,P,0,P,0,0] \quad kirsbp2 \quad kirsbp2d$$
$$IRS[R,0,0,0,P,0,P] \leftrightharpoons IRS[R,0,P,0,P,0,P] \quad kirsbp2 \quad kirsbp2d$$
$$IRS[R,0,0,0,P,P,0] \leftrightharpoons IRS[R,0,P,0,P,P,0] \quad kirsbp2 \quad kirsbp2d$$
$$IRS[R,0,0,0,P,P,P] \leftrightharpoons IRS[R,0,P,0,P,P,P] \quad kirsbp2 \quad kirsbp2d$$
$$IRS[R,0,0,P,0,0,0] \leftrightharpoons IRS[R,0,P,P,0,0,0] \quad kirsbp2 \quad kirsbp2d$$
$$IRS[R,0,0,P,0,0,P] \leftrightharpoons IRS[R,0,P,P,0,0,P] \quad kirsbp2 \quad kirsbp2d$$
$$IRS[R,0,0,P,0,P,0] \leftrightharpoons IRS[R,0,P,P,0,P,0] \quad kirsbp2 \quad kirsbp2d$$
$$IRS[R,0,0,P,0,P,P] \leftrightharpoons IRS[R,0,P,P,0,P,P] \quad kirsbp2 \quad kirsbp2d$$
$$IRS[R,0,0,P,P,0,0] \leftrightharpoons IRS[R,0,P,P,P,0,0] \quad kirsbp2 \quad kirsbp2d$$
$$IRS[R,0,0,P,P,0,P] \leftrightharpoons IRS[R,0,P,P,P,0,P] \quad kirsbp2 \quad kirsbp2d$$
$$IRS[R,0,0,P,P,P,0] \leftrightharpoons IRS[R,0,P,P,P,P,0] \quad kirsbp2 \quad kirsbp2d$$

$$IRS[R,0,0,P,P,P,P] \leftrightharpoons IRS[R,0,P,P,P,P,P] \quad kirsbp2 \quad kirsbp2d$$
$$IRS[R,P,0,0,0,0,0] \leftrightharpoons IRS[R,P,P,0,0,0,0] \quad kirsbp2 \quad kirsbp2d$$
$$IRS[R,P,0,0,0,0,P] \leftrightharpoons IRS[R,P,P,0,0,0,P] \quad kirsbp2 \quad kirsbp2d$$
$$IRS[R,P,0,0,0,P,0] \leftrightharpoons IRS[R,P,P,0,0,P,0] \quad kirsbp2 \quad kirsbp2d$$
$$IRS[R,P,0,0,0,P,P] \leftrightharpoons IRS[R,P,P,0,0,P,P] \quad kirsbp2 \quad kirsbp2d$$
$$IRS[R,P,0,0,P,0,0] \leftrightharpoons IRS[R,P,P,0,P,0,0] \quad kirsbp2 \quad kirsbp2d$$
$$IRS[R,P,0,0,P,0,P] \leftrightharpoons IRS[R,P,P,0,P,0,P] \quad kirsbp2 \quad kirsbp2d$$
$$IRS[R,P,0,0,P,P,0] \leftrightharpoons IRS[R,P,P,0,P,P,0] \quad kirsbp2 \quad kirsbp2d$$
$$IRS[R,P,0,0,P,P,P] \leftrightharpoons IRS[R,P,P,0,P,P,P] \quad kirsbp2 \quad kirsbp2d$$
$$IRS[R,P,0,P,0,0,0] \leftrightharpoons IRS[R,P,P,P,0,0,0] \quad kirsbp2 \quad kirsbp2d$$
$$IRS[R,P,0,P,0,0,P] \leftrightharpoons IRS[R,P,P,P,0,0,P] \quad kirsbp2 \quad kirsbp2d$$
$$IRS[R,P,0,P,0,P,0] \leftrightharpoons IRS[R,P,P,P,0,P,0] \quad kirsbp2 \quad kirsbp2d$$
$$IRS[R,P,0,P,0,P,P] \leftrightharpoons IRS[R,P,P,P,0,P,P] \quad kirsbp2 \quad kirsbp2d$$
$$IRS[R,P,0,P,P,0,0] \leftrightharpoons IRS[R,P,P,P,P,0,0] \quad kirsbp2 \quad kirsbp2d$$
$$IRS[R,P,0,P,P,0,P] \leftrightharpoons IRS[R,P,P,P,P,0,P] \quad kirsbp2 \quad kirsbp2d$$
$$IRS[R,P,0,P,P,P,0] \leftrightharpoons IRS[R,P,P,P,P,P,0] \quad kirsbp2 \quad kirsbp2d$$
$$IRS[R,P,0,P,P,P,P] \leftrightharpoons IRS[R,P,P,P,P,P,P] \quad kirsbp2 \quad kirsbp2d$$
$$IRS[R,0,0,0,0,0,0] \leftrightharpoons IRS[R,0,0,P,0,0,0] \quad kirsbp3 \quad kirsbp3d$$
$$IRS[R,0,0,0,0,0,P] \leftrightharpoons IRS[R,0,0,P,0,0,P] \quad kirsbp3 \quad kirsbp3d$$
$$IRS[R,0,0,0,0,P,0] \leftrightharpoons IRS[R,0,0,P,0,P,0] \quad kirsbp3 \quad kirsbp3d$$
$$IRS[R,0,0,0,0,P,P] \leftrightharpoons IRS[R,0,0,P,0,P,P] \quad kirsbp3 \quad kirsbp3d$$
$$IRS[R,0,0,0,P,0,0] \leftrightharpoons IRS[R,0,0,P,P,0,0] \quad kirsbp3 \quad kirsbp3d$$
$$IRS[R,0,0,0,P,0,P] \leftrightharpoons IRS[R,0,0,P,P,0,P] \quad kirsbp3 \quad kirsbp3d$$
$$IRS[R,0,0,0,P,P,0] \leftrightharpoons IRS[R,0,0,P,P,P,0] \quad kirsbp3 \quad kirsbp3d$$
$$IRS[R,0,0,0,P,P,P] \leftrightharpoons IRS[R,0,0,P,P,P,P] \quad kirsbp3 \quad kirsbp3d$$
$$IRS[R,0,P,0,0,0,0] \leftrightharpoons IRS[R,0,P,P,0,0,0] \quad kirsbp3 \quad kirsbp3d$$
$$IRS[R,0,P,0,0,0,P] \leftrightharpoons IRS[R,0,P,P,0,0,P] \quad kirsbp3 \quad kirsbp3d$$
$$IRS[R,0,P,0,0,P,0] \leftrightharpoons IRS[R,0,P,P,0,P,0] \quad kirsbp3 \quad kirsbp3d$$
$$IRS[R,0,P,0,0,P,P] \leftrightharpoons IRS[R,0,P,P,0,P,P] \quad kirsbp3 \quad kirsbp3d$$
$$IRS[R,0,P,0,P,0,0] \leftrightharpoons IRS[R,0,P,P,P,0,0] \quad kirsbp3 \quad kirsbp3d$$
$$IRS[R,0,P,0,P,0,P] \leftrightharpoons IRS[R,0,P,P,P,0,P] \quad kirsbp3 \quad kirsbp3d$$
$$IRS[R,0,P,0,P,P,0] \leftrightharpoons IRS[R,0,P,P,P,P,0] \quad kirsbp3 \quad kirsbp3d$$
$$IRS[R,0,P,0,P,P,P] \leftrightharpoons IRS[R,0,P,P,P,P,P] \quad kirsbp3 \quad kirsbp3d$$
$$IRS[R,P,0,0,0,0,0] \leftrightharpoons IRS[R,P,0,P,0,0,0] \quad kirsbp3 \quad kirsbp3d$$
$$IRS[R,P,0,0,0,0,P] \leftrightharpoons IRS[R,P,0,P,0,0,P] \quad kirsbp3 \quad kirsbp3d$$
$$IRS[R,P,0,0,0,P,0] \leftrightharpoons IRS[R,P,0,P,0,P,0] \quad kirsbp3 \quad kirsbp3d$$
$$IRS[R,P,0,0,0,P,P] \leftrightharpoons IRS[R,P,0,P,0,P,P] \quad kirsbp3 \quad kirsbp3d$$
$$IRS[R,P,0,0,P,0,0] \leftrightharpoons IRS[R,P,0,P,P,0,0] \quad kirsbp3 \quad kirsbp3d$$
$$IRS[R,P,0,0,P,0,P] \leftrightharpoons IRS[R,P,0,P,P,0,P] \quad kirsbp3 \quad kirsbp3d$$
$$IRS[R,P,0,0,P,P,0] \leftrightharpoons IRS[R,P,0,P,P,P,0] \quad kirsbp3 \quad kirsbp3d$$
$$IRS[R,P,0,0,P,P,P] \leftrightharpoons IRS[R,P,0,P,P,P,P] \quad kirsbp3 \quad kirsbp3d$$
$$IRS[R,P,P,0,0,0,0] \leftrightharpoons IRS[R,P,P,P,0,0,0] \quad kirsbp3 \quad kirsbp3d$$
$$IRS[R,P,P,0,0,0,P] \leftrightharpoons IRS[R,P,P,P,0,0,P] \quad kirsbp3 \quad kirsbp3d$$
$$IRS[R,P,P,0,0,P,0] \leftrightharpoons IRS[R,P,P,P,0,P,0] \quad kirsbp3 \quad kirsbp3d$$
$$IRS[R,P,P,0,0,P,P] \leftrightharpoons IRS[R,P,P,P,0,P,P] \quad kirsbp3 \quad kirsbp3d$$

$$IRS[R,P,P,0,P,0,0] \leftrightharpoons IRS[R,P,P,P,P,0,0] \quad kirsbp3 \quad kirsbp3d$$
$$IRS[R,P,P,0,P,0,P] \leftrightharpoons IRS[R,P,P,P,P,0,P] \quad kirsbp3 \quad kirsbp3d$$
$$IRS[R,P,P,0,P,P,0] \leftrightharpoons IRS[R,P,P,P,P,P,0] \quad kirsbp3 \quad kirsbp3d$$
$$IRS[R,P,P,0,P,P,P] \leftrightharpoons IRS[R,P,P,P,P,P,P] \quad kirsbp3 \quad kirsbp3d$$
$$IRS[R,0,0,0,0,0,0] \leftrightharpoons IRS[R,0,0,0,P,0,0] \quad kirsbp4 \quad kirsbp4d$$
$$IRS[R,0,0,0,0,0,P] \leftrightharpoons IRS[R,0,0,0,P,0,P] \quad kirsbp4 \quad kirsbp4d$$
$$IRS[R,0,0,0,0,P,0] \leftrightharpoons IRS[R,0,0,0,P,P,0] \quad kirsbp4 \quad kirsbp4d$$
$$IRS[R,0,0,0,0,P,P] \leftrightharpoons IRS[R,0,0,0,P,P,P] \quad kirsbp4 \quad kirsbp4d$$
$$IRS[R,0,0,P,0,0,0] \leftrightharpoons IRS[R,0,0,P,P,0,0] \quad kirsbp4 \quad kirsbp4d$$
$$IRS[R,0,0,P,0,0,P] \leftrightharpoons IRS[R,0,0,P,P,0,P] \quad kirsbp4 \quad kirsbp4d$$
$$IRS[R,0,0,P,0,P,0] \leftrightharpoons IRS[R,0,0,P,P,P,0] \quad kirsbp4 \quad kirsbp4d$$
$$IRS[R,0,0,P,0,P,P] \leftrightharpoons IRS[R,0,0,P,P,P,P] \quad kirsbp4 \quad kirsbp4d$$
$$IRS[R,0,P,0,0,0,0] \leftrightharpoons IRS[R,0,P,0,P,0,0] \quad kirsbp4 \quad kirsbp4d$$
$$IRS[R,0,P,0,0,0,P] \leftrightharpoons IRS[R,0,P,0,P,0,P] \quad kirsbp4 \quad kirsbp4d$$
$$IRS[R,0,P,0,0,P,0] \leftrightharpoons IRS[R,0,P,0,P,P,0] \quad kirsbp4 \quad kirsbp4d$$
$$IRS[R,0,P,0,0,P,P] \leftrightharpoons IRS[R,0,P,0,P,P,P] \quad kirsbp4 \quad kirsbp4d$$
$$IRS[R,0,P,P,0,0,0] \leftrightharpoons IRS[R,0,P,P,P,0,0] \quad kirsbp4 \quad kirsbp4d$$
$$IRS[R,0,P,P,0,0,P] \leftrightharpoons IRS[R,0,P,P,P,0,P] \quad kirsbp4 \quad kirsbp4d$$
$$IRS[R,0,P,P,0,P,0] \leftrightharpoons IRS[R,0,P,P,P,P,0] \quad kirsbp4 \quad kirsbp4d$$
$$IRS[R,0,P,P,0,P,P] \leftrightharpoons IRS[R,0,P,P,P,P,P] \quad kirsbp4 \quad kirsbp4d$$
$$IRS[R,P,0,0,0,0,0] \leftrightharpoons IRS[R,P,0,0,P,0,0] \quad kirsbp4 \quad kirsbp4d$$
$$IRS[R,P,0,0,0,0,P] \leftrightharpoons IRS[R,P,0,0,P,0,P] \quad kirsbp4 \quad kirsbp4d$$
$$IRS[R,P,0,0,0,P,0] \leftrightharpoons IRS[R,P,0,0,P,P,0] \quad kirsbp4 \quad kirsbp4d$$
$$IRS[R,P,0,0,0,P,P] \leftrightharpoons IRS[R,P,0,0,P,P,P] \quad kirsbp4 \quad kirsbp4d$$
$$IRS[R,P,0,P,0,0,0] \leftrightharpoons IRS[R,P,0,P,P,0,0] \quad kirsbp4 \quad kirsbp4d$$
$$IRS[R,P,0,P,0,0,P] \leftrightharpoons IRS[R,P,0,P,P,0,P] \quad kirsbp4 \quad kirsbp4d$$
$$IRS[R,P,0,P,0,P,0] \leftrightharpoons IRS[R,P,0,P,P,P,0] \quad kirsbp4 \quad kirsbp4d$$
$$IRS[R,P,0,P,0,P,P] \leftrightharpoons IRS[R,P,0,P,P,P,P] \quad kirsbp4 \quad kirsbp4d$$
$$IRS[R,P,P,0,0,0,0] \leftrightharpoons IRS[R,P,P,0,P,0,0] \quad kirsbp4 \quad kirsbp4d$$
$$IRS[R,P,P,0,0,0,P] \leftrightharpoons IRS[R,P,P,0,P,0,P] \quad kirsbp4 \quad kirsbp4d$$
$$IRS[R,P,P,0,0,P,0] \leftrightharpoons IRS[R,P,P,0,P,P,0] \quad kirsbp4 \quad kirsbp4d$$
$$IRS[R,P,P,0,0,P,P] \leftrightharpoons IRS[R,P,P,0,P,P,P] \quad kirsbp4 \quad kirsbp4d$$
$$IRS[R,P,P,P,0,0,0] \leftrightharpoons IRS[R,P,P,P,P,0,0] \quad kirsbp4 \quad kirsbp4d$$
$$IRS[R,P,P,P,0,0,P] \leftrightharpoons IRS[R,P,P,P,P,0,P] \quad kirsbp4 \quad kirsbp4d$$
$$IRS[R,P,P,P,0,P,0] \leftrightharpoons IRS[R,P,P,P,P,P,0] \quad kirsbp4 \quad kirsbp4d$$
$$IRS[R,P,P,P,0,P,P] \leftrightharpoons IRS[R,P,P,P,P,P,P] \quad kirsbp4 \quad kirsbp4d$$
$$IRS[R,0,0,0,0,0,0] \leftrightharpoons IRS[R,0,0,0,0,P,0] \quad kirsbp5 \quad kirsbp5d$$
$$IRS[R,0,0,0,0,0,P] \leftrightharpoons IRS[R,0,0,0,0,P,P] \quad kirsbp5 \quad kirsbp5d$$
$$IRS[R,0,0,0,P,0,0] \leftrightharpoons IRS[R,0,0,0,P,P,0] \quad kirsbp5 \quad kirsbp5d$$
$$IRS[R,0,0,0,P,0,P] \leftrightharpoons IRS[R,0,0,0,P,P,P] \quad kirsbp5 \quad kirsbp5d$$
$$IRS[R,0,0,P,0,0,0] \leftrightharpoons IRS[R,0,0,P,0,P,0] \quad kirsbp5 \quad kirsbp5d$$
$$IRS[R,0,0,P,0,0,P] \leftrightharpoons IRS[R,0,0,P,0,P,P] \quad kirsbp5 \quad kirsbp5d$$
$$IRS[R,0,0,P,P,0,0] \leftrightharpoons IRS[R,0,0,P,P,P,0] \quad kirsbp5 \quad kirsbp5d$$
$$IRS[R,0,0,P,P,0,P] \leftrightharpoons IRS[R,0,0,P,P,P,P] \quad kirsbp5 \quad kirsbp5d$$
$$IRS[R,0,P,0,0,0,0] \leftrightharpoons IRS[R,0,P,0,0,P,0] \quad kirsbp5 \quad kirsbp5d$$

$IRS[R,0,P,0,0,0,P] \leftrightharpoons IRS[R,0,P,0,0,P,P]$ $kirsbp5$ $kirsbp5d$

$IRS[R,0,P,0,P,0,0] \leftrightharpoons IRS[R,0,P,0,P,P,0]$ $kirsbp5$ $kirsbp5d$

$IRS[R,0,P,0,P,0,P] \leftrightharpoons IRS[R,0,P,0,P,P,P]$ $kirsbp5$ $kirsbp5d$

$IRS[R,0,P,P,0,0,0] \leftrightharpoons IRS[R,0,P,P,0,P,0]$ $kirsbp5$ $kirsbp5d$

$IRS[R,0,P,P,0,0,P] \leftrightharpoons IRS[R,0,P,P,0,P,P]$ $kirsbp5$ $kirsbp5d$

$IRS[R,0,P,P,P,0,0] \leftrightharpoons IRS[R,0,P,P,P,P,0]$ $kirsbp5$ $kirsbp5d$

$IRS[R,0,P,P,P,0,P] \leftrightharpoons IRS[R,0,P,P,P,P,P]$ $kirsbp5$ $kirsbp5d$

$IRS[R,P,0,0,0,0,0] \leftrightharpoons IRS[R,P,0,0,0,P,0]$ $kirsbp5$ $kirsbp5d$

$IRS[R,P,0,0,0,0,P] \leftrightharpoons IRS[R,P,0,0,0,P,P]$ $kirsbp5$ $kirsbp5d$

$IRS[R,P,0,0,P,0,0] \leftrightharpoons IRS[R,P,0,0,P,P,0]$ $kirsbp5$ $kirsbp5d$

$IRS[R,P,0,0,P,0,P] \leftrightharpoons IRS[R,P,0,0,P,P,P]$ $kirsbp5$ $kirsbp5d$

$IRS[R,P,0,P,0,0,0] \leftrightharpoons IRS[R,P,0,P,0,P,0]$ $kirsbp5$ $kirsbp5d$

$IRS[R,P,0,P,0,0,P] \leftrightharpoons IRS[R,P,0,P,0,P,P]$ $kirsbp5$ $kirsbp5d$

$IRS[R,P,0,P,P,0,0] \leftrightharpoons IRS[R,P,0,P,P,P,0]$ $kirsbp5$ $kirsbp5d$

$IRS[R,P,0,P,P,0,P] \leftrightharpoons IRS[R,P,0,P,P,P,P]$ $kirsbp5$ $kirsbp5d$

$IRS[R,P,P,0,0,0,0] \leftrightharpoons IRS[R,P,P,0,0,P,0]$ $kirsbp5$ $kirsbp5d$

$IRS[R,P,P,0,0,0,P] \leftrightharpoons IRS[R,P,P,0,0,P,P]$ $kirsbp5$ $kirsbp5d$

$IRS[R,P,P,0,P,0,0] \leftrightharpoons IRS[R,P,P,0,P,P,0]$ $kirsbp5$ $kirsbp5d$

$IRS[R,P,P,0,P,0,P] \leftrightharpoons IRS[R,P,P,0,P,P,P]$ $kirsbp5$ $kirsbp5d$

$IRS[R,P,P,P,0,0,0] \leftrightharpoons IRS[R,P,P,P,0,P,0]$ $kirsbp5$ $kirsbp5d$

$IRS[R,P,P,P,0,0,P] \leftrightharpoons IRS[R,P,P,P,0,P,P]$ $kirsbp5$ $kirsbp5d$

$IRS[R,P,P,P,P,0,0] \leftrightharpoons IRS[R,P,P,P,P,P,0]$ $kirsbp5$ $kirsbp5d$

$IRS[R,P,P,P,P,0,P] \leftrightharpoons IRS[R,P,P,P,P,P,P]$ $kirsbp5$ $kirsbp5d$

$IRS[R,0,0,0,0,0,0] \leftrightharpoons IRS[R,0,0,0,0,0,P]$ $kirsbp6$ $kirsbp6d$

$IRS[R,0,0,0,0,P,0] \leftrightharpoons IRS[R,0,0,0,0,P,P]$ $kirsbp6$ $kirsbp6d$

$IRS[R,0,0,0,P,0,0] \leftrightharpoons IRS[R,0,0,0,P,0,P]$ $kirsbp6$ $kirsbp6d$

$IRS[R,0,0,0,P,P,0] \leftrightharpoons IRS[R,0,0,0,P,P,P]$ $kirsbp6$ $kirsbp6d$

$IRS[R,0,0,P,0,0,0] \leftrightharpoons IRS[R,0,0,P,0,0,P]$ $kirsbp6$ $kirsbp6d$

$IRS[R,0,0,P,0,P,0] \leftrightharpoons IRS[R,0,0,P,0,P,P]$ $kirsbp6$ $kirsbp6d$

$IRS[R,0,0,P,P,0,0] \leftrightharpoons IRS[R,0,0,P,P,0,P]$ $kirsbp6$ $kirsbp6d$

$IRS[R,0,0,P,P,P,0] \leftrightharpoons IRS[R,0,0,P,P,P,P]$ $kirsbp6$ $kirsbp6d$

$IRS[R,0,P,0,0,0,0] \leftrightharpoons IRS[R,0,P,0,0,0,P]$ $kirsbp6$ $kirsbp6d$

$IRS[R,0,P,0,0,P,0] \leftrightharpoons IRS[R,0,P,0,0,P,P]$ $kirsbp6$ $kirsbp6d$

$IRS[R,0,P,0,P,0,0] \leftrightharpoons IRS[R,0,P,0,P,0,P]$ $kirsbp6$ $kirsbp6d$

$IRS[R,0,P,0,P,P,0] \leftrightharpoons IRS[R,0,P,0,P,P,P]$ $kirsbp6$ $kirsbp6d$

$IRS[R,0,P,P,0,0,0] \leftrightharpoons IRS[R,0,P,P,0,0,P]$ $kirsbp6$ $kirsbp6d$

$IRS[R,0,P,P,0,P,0] \leftrightharpoons IRS[R,0,P,P,0,P,P]$ $kirsbp6$ $kirsbp6d$

$IRS[R,0,P,P,P,0,0] \leftrightharpoons IRS[R,0,P,P,P,0,P]$ $kirsbp6$ $kirsbp6d$

$IRS[R,0,P,P,P,P,0] \leftrightharpoons IRS[R,0,P,P,P,P,P]$ $kirsbp6$ $kirsbp6d$

$IRS[R,P,0,0,0,0,0] \leftrightharpoons IRS[R,P,0,0,0,0,P]$ $kirsbp6$ $kirsbp6d$

$IRS[R,P,0,0,0,P,0] \leftrightharpoons IRS[R,P,0,0,0,P,P]$ $kirsbp6$ $kirsbp6d$

$IRS[R,P,0,0,P,0,0] \leftrightharpoons IRS[R,P,0,0,P,0,P]$ $kirsbp6$ $kirsbp6d$

$IRS[R,P,0,0,P,P,0] \leftrightharpoons IRS[R,P,0,0,P,P,P]$ $kirsbp6$ $kirsbp6d$

$IRS[R,P,0,P,0,0,0] \leftrightharpoons IRS[R,P,0,P,0,0,P]$ $kirsbp6$ $kirsbp6d$

$IRS[R,P,0,P,0,P,0] \leftrightharpoons IRS[R,P,0,P,0,P,P]$ $kirsbp6$ $kirsbp6d$

$$IRS[R,P,0,P,P,0,0] \leftrightharpoons IRS[R,P,0,P,P,0,P] \quad kirsbp6 \quad kirsbp6d$$
$$IRS[R,P,0,P,P,P,0] \leftrightharpoons IRS[R,P,0,P,P,P,P] \quad kirsbp6 \quad kirsbp6d$$
$$IRS[R,P,P,0,0,0,0] \leftrightharpoons IRS[R,P,P,0,0,0,P] \quad kirsbp6 \quad kirsbp6d$$
$$IRS[R,P,P,0,0,P,0] \leftrightharpoons IRS[R,P,P,0,0,P,P] \quad kirsbp6 \quad kirsbp6d$$
$$IRS[R,P,P,0,P,0,0] \leftrightharpoons IRS[R,P,P,0,P,0,P] \quad kirsbp6 \quad kirsbp6d$$
$$IRS[R,P,P,0,P,P,0] \leftrightharpoons IRS[R,P,P,0,P,P,P] \quad kirsbp6 \quad kirsbp6d$$
$$IRS[R,P,P,P,0,0,0] \leftrightharpoons IRS[R,P,P,P,0,0,P] \quad kirsbp6 \quad kirsbp6d$$
$$IRS[R,P,P,P,0,P,0] \leftrightharpoons IRS[R,P,P,P,0,P,P] \quad kirsbp6 \quad kirsbp6d$$
$$IRS[R,P,P,P,P,0,0] \leftrightharpoons IRS[R,P,P,P,P,0,P] \quad kirsbp6 \quad kirsbp6d$$
$$IRS[R,P,P,P,P,P,0] \leftrightharpoons IRS[R,P,P,P,P,P,P] \quad kirsbp6 \quad kirsbp6d$$

d) translate each reaction into the corresponding rate

$$r192 = kbirs * RXpIrs * IRS[0,0,0,0,0,0,0] - kbirsd * IRS[R,0,0,0,0,0,0]$$
$$r193 = kbirs * RXpIrs * IRS[0,0,0,0,0,0,P] - kbirsd * IRS[R,0,0,0,0,0,P]$$
$$r194 = kbirs * RXpIrs * IRS[0,0,0,0,0,P,0] - kbirsd * IRS[R,0,0,0,0,P,0]$$
$$r195 = kbirs * RXpIrs * IRS[0,0,0,0,0,P,P] - kbirsd * IRS[R,0,0,0,0,P,P]$$
$$r196 = kbirs * RXpIrs * IRS[0,0,0,0,P,0,0] - kbirsd * IRS[R,0,0,0,P,0,0]$$
$$r197 = kbirs * RXpIrs * IRS[0,0,0,0,P,0,P] - kbirsd * IRS[R,0,0,0,P,0,P]$$
$$r198 = kbirs * RXpIrs * IRS[0,0,0,0,P,P,0] - kbirsd * IRS[R,0,0,0,P,P,0]$$
$$r199 = kbirs * RXpIrs * IRS[0,0,0,0,P,P,P] - kbirsd * IRS[R,0,0,0,P,P,P]$$
$$r200 = kbirs * RXpIrs * IRS[0,0,0,P,0,0,0] - kbirsd * IRS[R,0,0,P,0,0,0]$$
$$r201 = kbirs * RXpIrs * IRS[0,0,0,P,0,0,P] - kbirsd * IRS[R,0,0,P,0,0,P]$$
$$r202 = kbirs * RXpIrs * IRS[0,0,0,P,0,P,0] - kbirsd * IRS[R,0,0,P,0,P,0]$$
$$r203 = kbirs * RXpIrs * IRS[0,0,0,P,0,P,P] - kbirsd * IRS[R,0,0,P,0,P,P]$$
$$r204 = kbirs * RXpIrs * IRS[0,0,0,P,P,0,0] - kbirsd * IRS[R,0,0,P,P,0,0]$$
$$r205 = kbirs * RXpIrs * IRS[0,0,0,P,P,0,P] - kbirsd * IRS[R,0,0,P,P,0,P]$$
$$r206 = kbirs * RXpIrs * IRS[0,0,0,P,P,P,0] - kbirsd * IRS[R,0,0,P,P,P,0]$$
$$r207 = kbirs * RXpIrs * IRS[0,0,0,P,P,P,P] - kbirsd * IRS[R,0,0,P,P,P,P]$$
$$r208 = kbirs * RXpIrs * IRS[0,0,P,0,0,0,0] - kbirsd * IRS[R,0,P,0,0,0,0]$$
$$r209 = kbirs * RXpIrs * IRS[0,0,P,0,0,0,P] - kbirsd * IRS[R,0,P,0,0,0,P]$$
$$r210 = kbirs * RXpIrs * IRS[0,0,P,0,0,P,0] - kbirsd * IRS[R,0,P,0,0,P,0]$$
$$r211 = kbirs * RXpIrs * IRS[0,0,P,0,0,P,P] - kbirsd * IRS[R,0,P,0,0,P,P]$$
$$r212 = kbirs * RXpIrs * IRS[0,0,P,0,P,0,0] - kbirsd * IRS[R,0,P,0,P,0,0]$$
$$r213 = kbirs * RXpIrs * IRS[0,0,P,0,P,0,P] - kbirsd * IRS[R,0,P,0,P,0,P]$$
$$r214 = kbirs * RXpIrs * IRS[0,0,P,0,P,P,0] - kbirsd * IRS[R,0,P,0,P,P,0]$$
$$r215 = kbirs * RXpIrs * IRS[0,0,P,0,P,P,P] - kbirsd * IRS[R,0,P,0,P,P,P]$$
$$r216 = kbirs * RXpIrs * IRS[0,0,P,P,0,0,0] - kbirsd * IRS[R,0,P,P,0,0,0]$$
$$r217 = kbirs * RXpIrs * IRS[0,0,P,P,0,0,P] - kbirsd * IRS[R,0,P,P,0,0,P]$$
$$r218 = kbirs * RXpIrs * IRS[0,0,P,P,0,P,0] - kbirsd * IRS[R,0,P,P,0,P,0]$$
$$r219 = kbirs * RXpIrs * IRS[0,0,P,P,0,P,P] - kbirsd * IRS[R,0,P,P,0,P,P]$$
$$r220 = kbirs * RXpIrs * IRS[0,0,P,P,P,0,0] - kbirsd * IRS[R,0,P,P,P,0,0]$$
$$r221 = kbirs * RXpIrs * IRS[0,0,P,P,P,0,P] - kbirsd * IRS[R,0,P,P,P,0,P]$$
$$r222 = kbirs * RXpIrs * IRS[0,0,P,P,P,P,0] - kbirsd * IRS[R,0,P,P,P,P,0]$$
$$r223 = kbirs * RXpIrs * IRS[0,0,P,P,P,P,P] - kbirsd * IRS[R,0,P,P,P,P,P]$$
$$r224 = kbirs * RXpIrs * IRS[0,P,0,0,0,0,0] - kbirsd * IRS[R,P,0,0,0,0,0]$$

$$r225 = kbirs * RXpIrs * IRS[0, P, 0, 0, 0, 0, P] - kbirsd * IRS[R, P, 0, 0, 0, 0, P]$$
$$r226 = kbirs * RXpIrs * IRS[0, P, 0, 0, 0, P, 0] - kbirsd * IRS[R, P, 0, 0, 0, P, 0]$$
$$r227 = kbirs * RXpIrs * IRS[0, P, 0, 0, 0, P, P] - kbirsd * IRS[R, P, 0, 0, 0, P, P]$$
$$r228 = kbirs * RXpIrs * IRS[0, P, 0, 0, P, 0, 0] - kbirsd * IRS[R, P, 0, 0, P, 0, 0]$$
$$r229 = kbirs * RXpIrs * IRS[0, P, 0, 0, P, 0, P] - kbirsd * IRS[R, P, 0, 0, P, 0, P]$$
$$r230 = kbirs * RXpIrs * IRS[0, P, 0, 0, P, P, 0] - kbirsd * IRS[R, P, 0, 0, P, P, 0]$$
$$r231 = kbirs * RXpIrs * IRS[0, P, 0, 0, P, P, P] - kbirsd * IRS[R, P, 0, 0, P, P, P]$$
$$r232 = kbirs * RXpIrs * IRS[0, P, 0, P, 0, 0, 0] - kbirsd * IRS[R, P, 0, P, 0, 0, 0]$$
$$r233 = kbirs * RXpIrs * IRS[0, P, 0, P, 0, 0, P] - kbirsd * IRS[R, P, 0, P, 0, 0, P]$$
$$r234 = kbirs * RXpIrs * IRS[0, P, 0, P, 0, P, 0] - kbirsd * IRS[R, P, 0, P, 0, P, 0]$$
$$r235 = kbirs * RXpIrs * IRS[0, P, 0, P, 0, P, P] - kbirsd * IRS[R, P, 0, P, 0, P, P]$$
$$r236 = kbirs * RXpIrs * IRS[0, P, 0, P, P, 0, 0] - kbirsd * IRS[R, P, 0, P, P, 0, 0]$$
$$r237 = kbirs * RXpIrs * IRS[0, P, 0, P, P, 0, P] - kbirsd * IRS[R, P, 0, P, P, 0, P]$$
$$r238 = kbirs * RXpIrs * IRS[0, P, 0, P, P, P, 0] - kbirsd * IRS[R, P, 0, P, P, P, 0]$$
$$r239 = kbirs * RXpIrs * IRS[0, P, 0, P, P, P, P] - kbirsd * IRS[R, P, 0, P, P, P, P]$$
$$r240 = kbirs * RXpIrs * IRS[0, P, P, 0, 0, 0, 0] - kbirsd * IRS[R, P, P, 0, 0, 0, 0]$$
$$r241 = kbirs * RXpIrs * IRS[0, P, P, 0, 0, 0, P] - kbirsd * IRS[R, P, P, 0, 0, 0, P]$$
$$r242 = kbirs * RXpIrs * IRS[0, P, P, 0, 0, P, 0] - kbirsd * IRS[R, P, P, 0, 0, P, 0]$$
$$r243 = kbirs * RXpIrs * IRS[0, P, P, 0, 0, P, P] - kbirsd * IRS[R, P, P, 0, 0, P, P]$$
$$r244 = kbirs * RXpIrs * IRS[0, P, P, 0, P, 0, 0] - kbirsd * IRS[R, P, P, 0, P, 0, 0]$$
$$r245 = kbirs * RXpIrs * IRS[0, P, P, 0, P, 0, P] - kbirsd * IRS[R, P, P, 0, P, 0, P]$$
$$r246 = kbirs * RXpIrs * IRS[0, P, P, 0, P, P, 0] - kbirsd * IRS[R, P, P, 0, P, P, 0]$$
$$r247 = kbirs * RXpIrs * IRS[0, P, P, 0, P, P, P] - kbirsd * IRS[R, P, P, 0, P, P, P]$$
$$r248 = kbirs * RXpIrs * IRS[0, P, P, P, 0, 0, 0] - kbirsd * IRS[R, P, P, P, 0, 0, 0]$$
$$r249 = kbirs * RXpIrs * IRS[0, P, P, P, 0, 0, P] - kbirsd * IRS[R, P, P, P, 0, 0, P]$$
$$r250 = kbirs * RXpIrs * IRS[0, P, P, P, 0, P, 0] - kbirsd * IRS[R, P, P, P, 0, P, 0]$$
$$r251 = kbirs * RXpIrs * IRS[0, P, P, P, 0, P, P] - kbirsd * IRS[R, P, P, P, 0, P, P]$$
$$r252 = kbirs * RXpIrs * IRS[0, P, P, P, P, 0, 0] - kbirsd * IRS[R, P, P, P, P, 0, 0]$$
$$r253 = kbirs * RXpIrs * IRS[0, P, P, P, P, 0, P] - kbirsd * IRS[R, P, P, P, P, 0, P]$$
$$r254 = kbirs * RXpIrs * IRS[0, P, P, P, P, P, 0] - kbirsd * IRS[R, P, P, P, P, P, 0]$$
$$r255 = kbirs * RXpIrs * IRS[0, P, P, P, P, P, P] - kbirsd * IRS[R, P, P, P, P, P, P]$$
$$r256 = kirsp1 * IRS[0, 0, 0, 0, 0, 0, 0] - kirsp1d * ((xIrsPi3k1 - xIrsPi3k1b)/xIrsPi3k1) * IRS[0, P, 0, 0, 0, 0, 0]$$
$$r257 = kirsp1 * IRS[0, 0, 0, 0, 0, 0, P] - kirsp1d * ((xIrsPi3k1 - xIrsPi3k1b)/xIrsPi3k1) * IRS[0, P, 0, 0, 0, 0, P]$$
$$r258 = kirsp1 * IRS[0, 0, 0, 0, 0, P, 0] - kirsp1d * ((xIrsPi3k1 - xIrsPi3k1b)/xIrsPi3k1) * IRS[0, P, 0, 0, 0, P, 0]$$
$$r259 = kirsp1 * IRS[0, 0, 0, 0, 0, P, P] - kirsp1d * ((xIrsPi3k1 - xIrsPi3k1b)/xIrsPi3k1) * IRS[0, P, 0, 0, 0, P, P]$$
$$r260 = kirsp1 * IRS[0, 0, 0, 0, P, 0, 0] - kirsp1d * ((xIrsPi3k1 - xIrsPi3k1b)/xIrsPi3k1) * IRS[0, P, 0, 0, P, 0, 0]$$
$$r261 = kirsp1 * IRS[0, 0, 0, 0, P, 0, P] - kirsp1d * ((xIrsPi3k1 - xIrsPi3k1b)/xIrsPi3k1) * IRS[0, P, 0, 0, P, 0, P]$$
$$r262 = kirsp1 * IRS[0, 0, 0, 0, P, P, 0] - kirsp1d * ((xIrsPi3k1 - xIrsPi3k1b)/xIrsPi3k1) * IRS[0, P, 0, 0, P, P, 0]$$
$$r263 = kirsp1 * IRS[0, 0, 0, 0, P, P, P] - kirsp1d * ((xIrsPi3k1 - xIrsPi3k1b)/xIrsPi3k1) * IRS[0, P, 0, 0, P, P, P]$$
$$r264 = kirsp1 * IRS[0, 0, 0, P, 0, 0, 0] - kirsp1d * ((xIrsPi3k1 - xIrsPi3k1b)/xIrsPi3k1) * IRS[0, P, 0, P, 0, 0, 0]$$
$$r265 = kirsp1 * IRS[0, 0, 0, P, 0, 0, P] - kirsp1d * ((xIrsPi3k1 - xIrsPi3k1b)/xIrsPi3k1) * IRS[0, P, 0, P, 0, 0, P]$$
$$r266 = kirsp1 * IRS[0, 0, 0, P, 0, P, 0] - kirsp1d * ((xIrsPi3k1 - xIrsPi3k1b)/xIrsPi3k1) * IRS[0, P, 0, P, 0, P, 0]$$
$$r267 = kirsp1 * IRS[0, 0, 0, P, 0, P, P] - kirsp1d * ((xIrsPi3k1 - xIrsPi3k1b)/xIrsPi3k1) * IRS[0, P, 0, P, 0, P, P]$$
$$r268 = kirsp1 * IRS[0, 0, 0, P, P, 0, 0] - kirsp1d * ((xIrsPi3k1 - xIrsPi3k1b)/xIrsPi3k1) * IRS[0, P, 0, P, P, 0, 0]$$
$$r269 = kirsp1 * IRS[0, 0, 0, P, P, 0, P] - kirsp1d * ((xIrsPi3k1 - xIrsPi3k1b)/xIrsPi3k1) * IRS[0, P, 0, P, P, 0, P]$$

$$r270 = kirsp1 * IRS[0,0,0,P,P,P,0] - kirsp1d * ((xIrsPi3k1 - xIrsPi3k1b)/xIrsPi3k1) * IRS[0,P,0,P,P,P,0]$$
$$r271 = kirsp1 * IRS[0,0,0,P,P,P,P] - kirsp1d * ((xIrsPi3k1 - xIrsPi3k1b)/xIrsPi3k1) * IRS[0,P,0,P,P,P,P]$$
$$r272 = kirsp1 * IRS[0,0,P,0,0,0,0] - kirsp1d * ((xIrsPi3k1 - xIrsPi3k1b)/xIrsPi3k1) * IRS[0,P,P,0,0,0,0]$$
$$r273 = kirsp1 * IRS[0,0,P,0,0,0,P] - kirsp1d * ((xIrsPi3k1 - xIrsPi3k1b)/xIrsPi3k1) * IRS[0,P,P,0,0,0,P]$$
$$r274 = kirsp1 * IRS[0,0,P,0,0,P,0] - kirsp1d * ((xIrsPi3k1 - xIrsPi3k1b)/xIrsPi3k1) * IRS[0,P,P,0,0,P,0]$$
$$r275 = kirsp1 * IRS[0,0,P,0,0,P,P] - kirsp1d * ((xIrsPi3k1 - xIrsPi3k1b)/xIrsPi3k1) * IRS[0,P,P,0,0,P,P]$$
$$r276 = kirsp1 * IRS[0,0,P,0,P,0,0] - kirsp1d * ((xIrsPi3k1 - xIrsPi3k1b)/xIrsPi3k1) * IRS[0,P,P,0,P,0,0]$$
$$r277 = kirsp1 * IRS[0,0,P,0,P,0,P] - kirsp1d * ((xIrsPi3k1 - xIrsPi3k1b)/xIrsPi3k1) * IRS[0,P,P,0,P,0,P]$$
$$r278 = kirsp1 * IRS[0,0,P,0,P,P,0] - kirsp1d * ((xIrsPi3k1 - xIrsPi3k1b)/xIrsPi3k1) * IRS[0,P,P,0,P,P,0]$$
$$r279 = kirsp1 * IRS[0,0,P,0,P,P,P] - kirsp1d * ((xIrsPi3k1 - xIrsPi3k1b)/xIrsPi3k1) * IRS[0,P,P,0,P,P,P]$$
$$r280 = kirsp1 * IRS[0,0,P,P,0,0,0] - kirsp1d * ((xIrsPi3k1 - xIrsPi3k1b)/xIrsPi3k1) * IRS[0,P,P,P,0,0,0]$$
$$r281 = kirsp1 * IRS[0,0,P,P,0,0,P] - kirsp1d * ((xIrsPi3k1 - xIrsPi3k1b)/xIrsPi3k1) * IRS[0,P,P,P,0,0,P]$$
$$r282 = kirsp1 * IRS[0,0,P,P,0,P,0] - kirsp1d * ((xIrsPi3k1 - xIrsPi3k1b)/xIrsPi3k1) * IRS[0,P,P,P,0,P,0]$$
$$r283 = kirsp1 * IRS[0,0,P,P,0,P,P] - kirsp1d * ((xIrsPi3k1 - xIrsPi3k1b)/xIrsPi3k1) * IRS[0,P,P,P,0,P,P]$$
$$r284 = kirsp1 * IRS[0,0,P,P,P,0,0] - kirsp1d * ((xIrsPi3k1 - xIrsPi3k1b)/xIrsPi3k1) * IRS[0,P,P,P,P,0,0]$$
$$r285 = kirsp1 * IRS[0,0,P,P,P,0,P] - kirsp1d * ((xIrsPi3k1 - xIrsPi3k1b)/xIrsPi3k1) * IRS[0,P,P,P,P,0,P]$$
$$r286 = kirsp1 * IRS[0,0,P,P,P,P,0] - kirsp1d * ((xIrsPi3k1 - xIrsPi3k1b)/xIrsPi3k1) * IRS[0,P,P,P,P,P,0]$$
$$r287 = kirsp1 * IRS[0,0,P,P,P,P,P] - kirsp1d * ((xIrsPi3k1 - xIrsPi3k1b)/xIrsPi3k1) * IRS[0,P,P,P,P,P,P]$$
$$r288 = kirsp2 * IRS[0,0,0,0,0,0,0] - kirsp2d * ((xIrsPi3k2 - xIrsPi3k2b)/xIrsPi3k2) * IRS[0,0,P,0,0,0,0]$$
$$r289 = kirsp2 * IRS[0,0,0,0,0,0,P] - kirsp2d * ((xIrsPi3k2 - xIrsPi3k2b)/xIrsPi3k2) * IRS[0,0,P,0,0,0,P]$$
$$r290 = kirsp2 * IRS[0,0,0,0,0,P,0] - kirsp2d * ((xIrsPi3k2 - xIrsPi3k2b)/xIrsPi3k2) * IRS[0,0,P,0,0,P,0]$$
$$r291 = kirsp2 * IRS[0,0,0,0,0,P,P] - kirsp2d * ((xIrsPi3k2 - xIrsPi3k2b)/xIrsPi3k2) * IRS[0,0,P,0,0,P,P]$$
$$r292 = kirsp2 * IRS[0,0,0,0,P,0,0] - kirsp2d * ((xIrsPi3k2 - xIrsPi3k2b)/xIrsPi3k2) * IRS[0,0,P,0,P,0,0]$$
$$r293 = kirsp2 * IRS[0,0,0,0,P,0,P] - kirsp2d * ((xIrsPi3k2 - xIrsPi3k2b)/xIrsPi3k2) * IRS[0,0,P,0,P,0,P]$$
$$r294 = kirsp2 * IRS[0,0,0,0,P,P,0] - kirsp2d * ((xIrsPi3k2 - xIrsPi3k2b)/xIrsPi3k2) * IRS[0,0,P,0,P,P,0]$$
$$r295 = kirsp2 * IRS[0,0,0,0,P,P,P] - kirsp2d * ((xIrsPi3k2 - xIrsPi3k2b)/xIrsPi3k2) * IRS[0,0,P,0,P,P,P]$$
$$r296 = kirsp2 * IRS[0,0,0,P,0,0,0] - kirsp2d * ((xIrsPi3k2 - xIrsPi3k2b)/xIrsPi3k2) * IRS[0,0,P,P,0,0,0]$$
$$r297 = kirsp2 * IRS[0,0,0,P,0,0,P] - kirsp2d * ((xIrsPi3k2 - xIrsPi3k2b)/xIrsPi3k2) * IRS[0,0,P,P,0,0,P]$$
$$r298 = kirsp2 * IRS[0,0,0,P,0,P,0] - kirsp2d * ((xIrsPi3k2 - xIrsPi3k2b)/xIrsPi3k2) * IRS[0,0,P,P,0,P,0]$$
$$r299 = kirsp2 * IRS[0,0,0,P,0,P,P] - kirsp2d * ((xIrsPi3k2 - xIrsPi3k2b)/xIrsPi3k2) * IRS[0,0,P,P,0,P,P]$$
$$r300 = kirsp2 * IRS[0,0,0,P,P,0,0] - kirsp2d * ((xIrsPi3k2 - xIrsPi3k2b)/xIrsPi3k2) * IRS[0,0,P,P,P,0,0]$$
$$r301 = kirsp2 * IRS[0,0,0,P,P,0,P] - kirsp2d * ((xIrsPi3k2 - xIrsPi3k2b)/xIrsPi3k2) * IRS[0,0,P,P,P,0,P]$$
$$r302 = kirsp2 * IRS[0,0,0,P,P,P,0] - kirsp2d * ((xIrsPi3k2 - xIrsPi3k2b)/xIrsPi3k2) * IRS[0,0,P,P,P,P,0]$$
$$r303 = kirsp2 * IRS[0,0,0,P,P,P,P] - kirsp2d * ((xIrsPi3k2 - xIrsPi3k2b)/xIrsPi3k2) * IRS[0,0,P,P,P,P,P]$$
$$r304 = kirsp2 * IRS[0,P,0,0,0,0,0] - kirsp2d * ((xIrsPi3k2 - xIrsPi3k2b)/xIrsPi3k2) * IRS[0,P,P,0,0,0,0]$$
$$r305 = kirsp2 * IRS[0,P,0,0,0,0,P] - kirsp2d * ((xIrsPi3k2 - xIrsPi3k2b)/xIrsPi3k2) * IRS[0,P,P,0,0,0,P]$$
$$r306 = kirsp2 * IRS[0,P,0,0,0,P,0] - kirsp2d * ((xIrsPi3k2 - xIrsPi3k2b)/xIrsPi3k2) * IRS[0,P,P,0,0,P,0]$$
$$r307 = kirsp2 * IRS[0,P,0,0,0,P,P] - kirsp2d * ((xIrsPi3k2 - xIrsPi3k2b)/xIrsPi3k2) * IRS[0,P,P,0,0,P,P]$$
$$r308 = kirsp2 * IRS[0,P,0,0,P,0,0] - kirsp2d * ((xIrsPi3k2 - xIrsPi3k2b)/xIrsPi3k2) * IRS[0,P,P,0,P,0,0]$$
$$r309 = kirsp2 * IRS[0,P,0,0,P,0,P] - kirsp2d * ((xIrsPi3k2 - xIrsPi3k2b)/xIrsPi3k2) * IRS[0,P,P,0,P,0,P]$$
$$r310 = kirsp2 * IRS[0,P,0,0,P,P,0] - kirsp2d * ((xIrsPi3k2 - xIrsPi3k2b)/xIrsPi3k2) * IRS[0,P,P,0,P,P,0]$$
$$r311 = kirsp2 * IRS[0,P,0,0,P,P,P] - kirsp2d * ((xIrsPi3k2 - xIrsPi3k2b)/xIrsPi3k2) * IRS[0,P,P,0,P,P,P]$$
$$r312 = kirsp2 * IRS[0,P,0,P,0,0,0] - kirsp2d * ((xIrsPi3k2 - xIrsPi3k2b)/xIrsPi3k2) * IRS[0,P,P,P,0,0,0]$$
$$r313 = kirsp2 * IRS[0,P,0,P,0,0,P] - kirsp2d * ((xIrsPi3k2 - xIrsPi3k2b)/xIrsPi3k2) * IRS[0,P,P,P,0,0,P]$$
$$r314 = kirsp2 * IRS[0,P,0,P,0,P,0] - kirsp2d * ((xIrsPi3k2 - xIrsPi3k2b)/xIrsPi3k2) * IRS[0,P,P,P,0,P,0]$$

$$r315 = kirsp2 * IRS[0,P,0,P,0,P,P] - kirsp2d * ((xIrsPi3k2 - xIrsPi3k2b)/xIrsPi3k2) * IRS[0,P,P,P,0,P,P]$$
$$r316 = kirsp2 * IRS[0,P,0,P,P,0,0] - kirsp2d * ((xIrsPi3k2 - xIrsPi3k2b)/xIrsPi3k2) * IRS[0,P,P,P,P,0,0]$$
$$r317 = kirsp2 * IRS[0,P,0,P,P,0,P] - kirsp2d * ((xIrsPi3k2 - xIrsPi3k2b)/xIrsPi3k2) * IRS[0,P,P,P,P,0,P]$$
$$r318 = kirsp2 * IRS[0,P,0,P,P,P,0] - kirsp2d * ((xIrsPi3k2 - xIrsPi3k2b)/xIrsPi3k2) * IRS[0,P,P,P,P,P,0]$$
$$r319 = kirsp2 * IRS[0,P,0,P,P,P,P] - kirsp2d * ((xIrsPi3k2 - xIrsPi3k2b)/xIrsPi3k2) * IRS[0,P,P,P,P,P,P]$$
$$r320 = kirsp3 * IRS[0,0,0,0,0,0,0] - kirsp3d * ((xIrsPi3k3 - xIrsPi3k3b)/xIrsPi3k3) * IRS[0,0,0,P,0,0,0]$$
$$r321 = kirsp3 * IRS[0,0,0,0,0,0,P] - kirsp3d * ((xIrsPi3k3 - xIrsPi3k3b)/xIrsPi3k3) * IRS[0,0,0,P,0,0,P]$$
$$r322 = kirsp3 * IRS[0,0,0,0,0,P,0] - kirsp3d * ((xIrsPi3k3 - xIrsPi3k3b)/xIrsPi3k3) * IRS[0,0,0,P,0,P,0]$$
$$r323 = kirsp3 * IRS[0,0,0,0,0,P,P] - kirsp3d * ((xIrsPi3k3 - xIrsPi3k3b)/xIrsPi3k3) * IRS[0,0,0,P,0,P,P]$$
$$r324 = kirsp3 * IRS[0,0,0,0,P,0,0] - kirsp3d * ((xIrsPi3k3 - xIrsPi3k3b)/xIrsPi3k3) * IRS[0,0,0,P,P,0,0]$$
$$r325 = kirsp3 * IRS[0,0,0,0,P,0,P] - kirsp3d * ((xIrsPi3k3 - xIrsPi3k3b)/xIrsPi3k3) * IRS[0,0,0,P,P,0,P]$$
$$r326 = kirsp3 * IRS[0,0,0,0,P,P,0] - kirsp3d * ((xIrsPi3k3 - xIrsPi3k3b)/xIrsPi3k3) * IRS[0,0,0,P,P,P,0]$$
$$r327 = kirsp3 * IRS[0,0,0,0,P,P,P] - kirsp3d * ((xIrsPi3k3 - xIrsPi3k3b)/xIrsPi3k3) * IRS[0,0,0,P,P,P,P]$$
$$r328 = kirsp3 * IRS[0,0,P,0,0,0,0] - kirsp3d * ((xIrsPi3k3 - xIrsPi3k3b)/xIrsPi3k3) * IRS[0,0,P,P,0,0,0]$$
$$r329 = kirsp3 * IRS[0,0,P,0,0,0,P] - kirsp3d * ((xIrsPi3k3 - xIrsPi3k3b)/xIrsPi3k3) * IRS[0,0,P,P,0,0,P]$$
$$r330 = kirsp3 * IRS[0,0,P,0,0,P,0] - kirsp3d * ((xIrsPi3k3 - xIrsPi3k3b)/xIrsPi3k3) * IRS[0,0,P,P,0,P,0]$$
$$r331 = kirsp3 * IRS[0,0,P,0,0,P,P] - kirsp3d * ((xIrsPi3k3 - xIrsPi3k3b)/xIrsPi3k3) * IRS[0,0,P,P,0,P,P]$$
$$r332 = kirsp3 * IRS[0,0,P,0,P,0,0] - kirsp3d * ((xIrsPi3k3 - xIrsPi3k3b)/xIrsPi3k3) * IRS[0,0,P,P,P,0,0]$$
$$r333 = kirsp3 * IRS[0,0,P,0,P,0,P] - kirsp3d * ((xIrsPi3k3 - xIrsPi3k3b)/xIrsPi3k3) * IRS[0,0,P,P,P,0,P]$$
$$r334 = kirsp3 * IRS[0,0,P,0,P,P,0] - kirsp3d * ((xIrsPi3k3 - xIrsPi3k3b)/xIrsPi3k3) * IRS[0,0,P,P,P,P,0]$$
$$r335 = kirsp3 * IRS[0,0,P,0,P,P,P] - kirsp3d * ((xIrsPi3k3 - xIrsPi3k3b)/xIrsPi3k3) * IRS[0,0,P,P,P,P,P]$$
$$r336 = kirsp3 * IRS[0,P,0,0,0,0,0] - kirsp3d * ((xIrsPi3k3 - xIrsPi3k3b)/xIrsPi3k3) * IRS[0,P,0,P,0,0,0]$$
$$r337 = kirsp3 * IRS[0,P,0,0,0,0,P] - kirsp3d * ((xIrsPi3k3 - xIrsPi3k3b)/xIrsPi3k3) * IRS[0,P,0,P,0,0,P]$$
$$r338 = kirsp3 * IRS[0,P,0,0,0,P,0] - kirsp3d * ((xIrsPi3k3 - xIrsPi3k3b)/xIrsPi3k3) * IRS[0,P,0,P,0,P,0]$$
$$r339 = kirsp3 * IRS[0,P,0,0,0,P,P] - kirsp3d * ((xIrsPi3k3 - xIrsPi3k3b)/xIrsPi3k3) * IRS[0,P,0,P,0,P,P]$$
$$r340 = kirsp3 * IRS[0,P,0,0,P,0,0] - kirsp3d * ((xIrsPi3k3 - xIrsPi3k3b)/xIrsPi3k3) * IRS[0,P,0,P,P,0,0]$$
$$r341 = kirsp3 * IRS[0,P,0,0,P,0,P] - kirsp3d * ((xIrsPi3k3 - xIrsPi3k3b)/xIrsPi3k3) * IRS[0,P,0,P,P,0,P]$$
$$r342 = kirsp3 * IRS[0,P,0,0,P,P,0] - kirsp3d * ((xIrsPi3k3 - xIrsPi3k3b)/xIrsPi3k3) * IRS[0,P,0,P,P,P,0]$$
$$r343 = kirsp3 * IRS[0,P,0,0,P,P,P] - kirsp3d * ((xIrsPi3k3 - xIrsPi3k3b)/xIrsPi3k3) * IRS[0,P,0,P,P,P,P]$$
$$r344 = kirsp3 * IRS[0,P,P,0,0,0,0] - kirsp3d * ((xIrsPi3k3 - xIrsPi3k3b)/xIrsPi3k3) * IRS[0,P,P,P,0,0,0]$$
$$r345 = kirsp3 * IRS[0,P,P,0,0,0,P] - kirsp3d * ((xIrsPi3k3 - xIrsPi3k3b)/xIrsPi3k3) * IRS[0,P,P,P,0,0,P]$$
$$r346 = kirsp3 * IRS[0,P,P,0,0,P,0] - kirsp3d * ((xIrsPi3k3 - xIrsPi3k3b)/xIrsPi3k3) * IRS[0,P,P,P,0,P,0]$$
$$r347 = kirsp3 * IRS[0,P,P,0,0,P,P] - kirsp3d * ((xIrsPi3k3 - xIrsPi3k3b)/xIrsPi3k3) * IRS[0,P,P,P,0,P,P]$$
$$r348 = kirsp3 * IRS[0,P,P,0,P,0,0] - kirsp3d * ((xIrsPi3k3 - xIrsPi3k3b)/xIrsPi3k3) * IRS[0,P,P,P,P,0,0]$$
$$r349 = kirsp3 * IRS[0,P,P,0,P,0,P] - kirsp3d * ((xIrsPi3k3 - xIrsPi3k3b)/xIrsPi3k3) * IRS[0,P,P,P,P,0,P]$$
$$r350 = kirsp3 * IRS[0,P,P,0,P,P,0] - kirsp3d * ((xIrsPi3k3 - xIrsPi3k3b)/xIrsPi3k3) * IRS[0,P,P,P,P,P,0]$$
$$r351 = kirsp3 * IRS[0,P,P,0,P,P,P] - kirsp3d * ((xIrsPi3k3 - xIrsPi3k3b)/xIrsPi3k3) * IRS[0,P,P,P,P,P,P]$$
$$r352 = kirsp4 * IRS[0,0,0,0,0,0,0] - kirsp4d * ((xIrsPi3k4 - xIrsPi3k4b)/xIrsPi3k4) * IRS[0,0,0,0,P,0,0]$$
$$r353 = kirsp4 * IRS[0,0,0,0,0,0,P] - kirsp4d * ((xIrsPi3k4 - xIrsPi3k4b)/xIrsPi3k4) * IRS[0,0,0,0,P,0,P]$$
$$r354 = kirsp4 * IRS[0,0,0,0,0,P,0] - kirsp4d * ((xIrsPi3k4 - xIrsPi3k4b)/xIrsPi3k4) * IRS[0,0,0,0,P,P,0]$$
$$r355 = kirsp4 * IRS[0,0,0,0,0,P,P] - kirsp4d * ((xIrsPi3k4 - xIrsPi3k4b)/xIrsPi3k4) * IRS[0,0,0,0,P,P,P]$$
$$r356 = kirsp4 * IRS[0,0,0,P,0,0,0] - kirsp4d * ((xIrsPi3k4 - xIrsPi3k4b)/xIrsPi3k4) * IRS[0,0,0,P,P,0,0]$$
$$r357 = kirsp4 * IRS[0,0,0,P,0,0,P] - kirsp4d * ((xIrsPi3k4 - xIrsPi3k4b)/xIrsPi3k4) * IRS[0,0,0,P,P,0,P]$$
$$r358 = kirsp4 * IRS[0,0,0,P,0,P,0] - kirsp4d * ((xIrsPi3k4 - xIrsPi3k4b)/xIrsPi3k4) * IRS[0,0,0,P,P,P,0]$$
$$r359 = kirsp4 * IRS[0,0,0,P,0,P,P] - kirsp4d * ((xIrsPi3k4 - xIrsPi3k4b)/xIrsPi3k4) * IRS[0,0,0,P,P,P,P]$$

$$r360 = kirsp4 * IRS[0,0,P,0,0,0,0] - kirsp4d * ((xIrsPi3k4 - xIrsPi3k4b)/xIrsPi3k4) * IRS[0,0,P,0,P,0,0]$$
$$r361 = kirsp4 * IRS[0,0,P,0,0,0,P] - kirsp4d * ((xIrsPi3k4 - xIrsPi3k4b)/xIrsPi3k4) * IRS[0,0,P,0,P,0,P]$$
$$r362 = kirsp4 * IRS[0,0,P,0,0,P,0] - kirsp4d * ((xIrsPi3k4 - xIrsPi3k4b)/xIrsPi3k4) * IRS[0,0,P,0,P,P,0]$$
$$r363 = kirsp4 * IRS[0,0,P,0,0,P,P] - kirsp4d * ((xIrsPi3k4 - xIrsPi3k4b)/xIrsPi3k4) * IRS[0,0,P,0,P,P,P]$$
$$r364 = kirsp4 * IRS[0,0,P,P,0,0,0] - kirsp4d * ((xIrsPi3k4 - xIrsPi3k4b)/xIrsPi3k4) * IRS[0,0,P,P,P,0,0]$$
$$r365 = kirsp4 * IRS[0,0,P,P,0,0,P] - kirsp4d * ((xIrsPi3k4 - xIrsPi3k4b)/xIrsPi3k4) * IRS[0,0,P,P,P,0,P]$$
$$r366 = kirsp4 * IRS[0,0,P,P,0,P,0] - kirsp4d * ((xIrsPi3k4 - xIrsPi3k4b)/xIrsPi3k4) * IRS[0,0,P,P,P,P,0]$$
$$r367 = kirsp4 * IRS[0,0,P,P,0,P,P] - kirsp4d * ((xIrsPi3k4 - xIrsPi3k4b)/xIrsPi3k4) * IRS[0,0,P,P,P,P,P]$$
$$r368 = kirsp4 * IRS[0,P,0,0,0,0,0] - kirsp4d * ((xIrsPi3k4 - xIrsPi3k4b)/xIrsPi3k4) * IRS[0,P,0,0,P,0,0]$$
$$r369 = kirsp4 * IRS[0,P,0,0,0,0,P] - kirsp4d * ((xIrsPi3k4 - xIrsPi3k4b)/xIrsPi3k4) * IRS[0,P,0,0,P,0,P]$$
$$r370 = kirsp4 * IRS[0,P,0,0,0,P,0] - kirsp4d * ((xIrsPi3k4 - xIrsPi3k4b)/xIrsPi3k4) * IRS[0,P,0,0,P,P,0]$$
$$r371 = kirsp4 * IRS[0,P,0,0,0,P,P] - kirsp4d * ((xIrsPi3k4 - xIrsPi3k4b)/xIrsPi3k4) * IRS[0,P,0,0,P,P,P]$$
$$r372 = kirsp4 * IRS[0,P,0,P,0,0,0] - kirsp4d * ((xIrsPi3k4 - xIrsPi3k4b)/xIrsPi3k4) * IRS[0,P,0,P,P,0,0]$$
$$r373 = kirsp4 * IRS[0,P,0,P,0,0,P] - kirsp4d * ((xIrsPi3k4 - xIrsPi3k4b)/xIrsPi3k4) * IRS[0,P,0,P,P,0,P]$$
$$r374 = kirsp4 * IRS[0,P,0,P,0,P,0] - kirsp4d * ((xIrsPi3k4 - xIrsPi3k4b)/xIrsPi3k4) * IRS[0,P,0,P,P,P,0]$$
$$r375 = kirsp4 * IRS[0,P,0,P,0,P,P] - kirsp4d * ((xIrsPi3k4 - xIrsPi3k4b)/xIrsPi3k4) * IRS[0,P,0,P,P,P,P]$$
$$r376 = kirsp4 * IRS[0,P,P,0,0,0,0] - kirsp4d * ((xIrsPi3k4 - xIrsPi3k4b)/xIrsPi3k4) * IRS[0,P,P,0,P,0,0]$$
$$r377 = kirsp4 * IRS[0,P,P,0,0,0,P] - kirsp4d * ((xIrsPi3k4 - xIrsPi3k4b)/xIrsPi3k4) * IRS[0,P,P,0,P,0,P]$$
$$r378 = kirsp4 * IRS[0,P,P,0,0,P,0] - kirsp4d * ((xIrsPi3k4 - xIrsPi3k4b)/xIrsPi3k4) * IRS[0,P,P,0,P,P,0]$$
$$r379 = kirsp4 * IRS[0,P,P,0,0,P,P] - kirsp4d * ((xIrsPi3k4 - xIrsPi3k4b)/xIrsPi3k4) * IRS[0,P,P,0,P,P,P]$$
$$r380 = kirsp4 * IRS[0,P,P,P,0,0,0] - kirsp4d * ((xIrsPi3k4 - xIrsPi3k4b)/xIrsPi3k4) * IRS[0,P,P,P,P,0,0]$$
$$r381 = kirsp4 * IRS[0,P,P,P,0,0,P] - kirsp4d * ((xIrsPi3k4 - xIrsPi3k4b)/xIrsPi3k4) * IRS[0,P,P,P,P,0,P]$$
$$r382 = kirsp4 * IRS[0,P,P,P,0,P,0] - kirsp4d * ((xIrsPi3k4 - xIrsPi3k4b)/xIrsPi3k4) * IRS[0,P,P,P,P,P,0]$$
$$r383 = kirsp4 * IRS[0,P,P,P,0,P,P] - kirsp4d * ((xIrsPi3k4 - xIrsPi3k4b)/xIrsPi3k4) * IRS[0,P,P,P,P,P,P]$$
$$r384 = kirsp5 * IRS[0,0,0,0,0,0,0] - kirsp5d * ((xIrsShp2 - xIrsShp2b)/xIrsShp2) * IRS[0,0,0,0,0,P,0]$$
$$r385 = kirsp5 * IRS[0,0,0,0,0,0,P] - kirsp5d * ((xIrsShp2 - xIrsShp2b)/xIrsShp2) * IRS[0,0,0,0,0,P,P]$$
$$r386 = kirsp5 * IRS[0,0,0,0,P,0,0] - kirsp5d * ((xIrsShp2 - xIrsShp2b)/xIrsShp2) * IRS[0,0,0,0,P,P,0]$$
$$r387 = kirsp5 * IRS[0,0,0,0,P,0,P] - kirsp5d * ((xIrsShp2 - xIrsShp2b)/xIrsShp2) * IRS[0,0,0,0,P,P,P]$$
$$r388 = kirsp5 * IRS[0,0,0,P,0,0,0] - kirsp5d * ((xIrsShp2 - xIrsShp2b)/xIrsShp2) * IRS[0,0,0,P,0,P,0]$$
$$r389 = kirsp5 * IRS[0,0,0,P,0,0,P] - kirsp5d * ((xIrsShp2 - xIrsShp2b)/xIrsShp2) * IRS[0,0,0,P,0,P,P]$$
$$r390 = kirsp5 * IRS[0,0,0,P,P,0,0] - kirsp5d * ((xIrsShp2 - xIrsShp2b)/xIrsShp2) * IRS[0,0,0,P,P,P,0]$$
$$r391 = kirsp5 * IRS[0,0,0,P,P,0,P] - kirsp5d * ((xIrsShp2 - xIrsShp2b)/xIrsShp2) * IRS[0,0,0,P,P,P,P]$$
$$r392 = kirsp5 * IRS[0,0,P,0,0,0,0] - kirsp5d * ((xIrsShp2 - xIrsShp2b)/xIrsShp2) * IRS[0,0,P,0,0,P,0]$$
$$r393 = kirsp5 * IRS[0,0,P,0,0,0,P] - kirsp5d * ((xIrsShp2 - xIrsShp2b)/xIrsShp2) * IRS[0,0,P,0,0,P,P]$$
$$r394 = kirsp5 * IRS[0,0,P,0,P,0,0] - kirsp5d * ((xIrsShp2 - xIrsShp2b)/xIrsShp2) * IRS[0,0,P,0,P,P,0]$$
$$r395 = kirsp5 * IRS[0,0,P,0,P,0,P] - kirsp5d * ((xIrsShp2 - xIrsShp2b)/xIrsShp2) * IRS[0,0,P,0,P,P,P]$$
$$r396 = kirsp5 * IRS[0,0,P,P,0,0,0] - kirsp5d * ((xIrsShp2 - xIrsShp2b)/xIrsShp2) * IRS[0,0,P,P,0,P,0]$$
$$r397 = kirsp5 * IRS[0,0,P,P,0,0,P] - kirsp5d * ((xIrsShp2 - xIrsShp2b)/xIrsShp2) * IRS[0,0,P,P,0,P,P]$$
$$r398 = kirsp5 * IRS[0,0,P,P,P,0,0] - kirsp5d * ((xIrsShp2 - xIrsShp2b)/xIrsShp2) * IRS[0,0,P,P,P,P,0]$$
$$r399 = kirsp5 * IRS[0,0,P,P,P,0,P] - kirsp5d * ((xIrsShp2 - xIrsShp2b)/xIrsShp2) * IRS[0,0,P,P,P,P,P]$$
$$r400 = kirsp5 * IRS[0,P,0,0,0,0,0] - kirsp5d * ((xIrsShp2 - xIrsShp2b)/xIrsShp2) * IRS[0,P,0,0,0,P,0]$$
$$r401 = kirsp5 * IRS[0,P,0,0,0,0,P] - kirsp5d * ((xIrsShp2 - xIrsShp2b)/xIrsShp2) * IRS[0,P,0,0,0,P,P]$$
$$r402 = kirsp5 * IRS[0,P,0,0,P,0,0] - kirsp5d * ((xIrsShp2 - xIrsShp2b)/xIrsShp2) * IRS[0,P,0,0,P,P,0]$$
$$r403 = kirsp5 * IRS[0,P,0,0,P,0,P] - kirsp5d * ((xIrsShp2 - xIrsShp2b)/xIrsShp2) * IRS[0,P,0,0,P,P,P]$$
$$r404 = kirsp5 * IRS[0,P,0,P,0,0,0] - kirsp5d * ((xIrsShp2 - xIrsShp2b)/xIrsShp2) * IRS[0,P,0,P,0,P,0]$$

$$r405 = kirsp5 * IRS[0, P, 0, P, 0, 0, P] - kirsp5d * ((xIrsShp2 - xIrsShp2b)/xIrsShp2) * IRS[0, P, 0, P, 0, P, P]$$
$$r406 = kirsp5 * IRS[0, P, 0, P, P, 0, 0] - kirsp5d * ((xIrsShp2 - xIrsShp2b)/xIrsShp2) * IRS[0, P, 0, P, P, P, 0]$$
$$r407 = kirsp5 * IRS[0, P, 0, P, P, 0, P] - kirsp5d * ((xIrsShp2 - xIrsShp2b)/xIrsShp2) * IRS[0, P, 0, P, P, P, P]$$
$$r408 = kirsp5 * IRS[0, P, P, 0, 0, 0, 0] - kirsp5d * ((xIrsShp2 - xIrsShp2b)/xIrsShp2) * IRS[0, P, P, 0, 0, P, 0]$$
$$r409 = kirsp5 * IRS[0, P, P, 0, 0, 0, P] - kirsp5d * ((xIrsShp2 - xIrsShp2b)/xIrsShp2) * IRS[0, P, P, 0, 0, P, P]$$
$$r410 = kirsp5 * IRS[0, P, P, 0, P, 0, 0] - kirsp5d * ((xIrsShp2 - xIrsShp2b)/xIrsShp2) * IRS[0, P, P, 0, P, P, 0]$$
$$r411 = kirsp5 * IRS[0, P, P, 0, P, 0, P] - kirsp5d * ((xIrsShp2 - xIrsShp2b)/xIrsShp2) * IRS[0, P, P, 0, P, P, P]$$
$$r412 = kirsp5 * IRS[0, P, P, P, 0, 0, 0] - kirsp5d * ((xIrsShp2 - xIrsShp2b)/xIrsShp2) * IRS[0, P, P, P, 0, P, 0]$$
$$r413 = kirsp5 * IRS[0, P, P, P, 0, 0, P] - kirsp5d * ((xIrsShp2 - xIrsShp2b)/xIrsShp2) * IRS[0, P, P, P, 0, P, P]$$
$$r414 = kirsp5 * IRS[0, P, P, P, P, 0, 0] - kirsp5d * ((xIrsShp2 - xIrsShp2b)/xIrsShp2) * IRS[0, P, P, P, P, P, 0]$$
$$r415 = kirsp5 * IRS[0, P, P, P, P, 0, P] - kirsp5d * ((xIrsShp2 - xIrsShp2b)/xIrsShp2) * IRS[0, P, P, P, P, P, P]$$
$$r416 = kirsp6 * IRS[0, 0, 0, 0, 0, 0, 0] - kirsp6d * ((xIrsGrb2 - xIrsGrb2b)/xIrsGrb2) * IRS[0, 0, 0, 0, 0, 0, P]$$
$$r417 = kirsp6 * IRS[0, 0, 0, 0, 0, P, 0] - kirsp6d * ((xIrsGrb2 - xIrsGrb2b)/xIrsGrb2) * IRS[0, 0, 0, 0, 0, P, P]$$
$$r418 = kirsp6 * IRS[0, 0, 0, 0, P, 0, 0] - kirsp6d * ((xIrsGrb2 - xIrsGrb2b)/xIrsGrb2) * IRS[0, 0, 0, 0, P, 0, P]$$
$$r419 = kirsp6 * IRS[0, 0, 0, 0, P, P, 0] - kirsp6d * ((xIrsGrb2 - xIrsGrb2b)/xIrsGrb2) * IRS[0, 0, 0, 0, P, P, P]$$
$$r420 = kirsp6 * IRS[0, 0, 0, P, 0, 0, 0] - kirsp6d * ((xIrsGrb2 - xIrsGrb2b)/xIrsGrb2) * IRS[0, 0, 0, P, 0, 0, P]$$
$$r421 = kirsp6 * IRS[0, 0, 0, P, 0, P, 0] - kirsp6d * ((xIrsGrb2 - xIrsGrb2b)/xIrsGrb2) * IRS[0, 0, 0, P, 0, P, P]$$
$$r422 = kirsp6 * IRS[0, 0, 0, P, P, 0, 0] - kirsp6d * ((xIrsGrb2 - xIrsGrb2b)/xIrsGrb2) * IRS[0, 0, 0, P, P, 0, P]$$
$$r423 = kirsp6 * IRS[0, 0, 0, P, P, P, 0] - kirsp6d * ((xIrsGrb2 - xIrsGrb2b)/xIrsGrb2) * IRS[0, 0, 0, P, P, P, P]$$
$$r424 = kirsp6 * IRS[0, 0, P, 0, 0, 0, 0] - kirsp6d * ((xIrsGrb2 - xIrsGrb2b)/xIrsGrb2) * IRS[0, 0, P, 0, 0, 0, P]$$
$$r425 = kirsp6 * IRS[0, 0, P, 0, 0, P, 0] - kirsp6d * ((xIrsGrb2 - xIrsGrb2b)/xIrsGrb2) * IRS[0, 0, P, 0, 0, P, P]$$
$$r426 = kirsp6 * IRS[0, 0, P, 0, P, 0, 0] - kirsp6d * ((xIrsGrb2 - xIrsGrb2b)/xIrsGrb2) * IRS[0, 0, P, 0, P, 0, P]$$
$$r427 = kirsp6 * IRS[0, 0, P, 0, P, P, 0] - kirsp6d * ((xIrsGrb2 - xIrsGrb2b)/xIrsGrb2) * IRS[0, 0, P, 0, P, P, P]$$
$$r428 = kirsp6 * IRS[0, 0, P, P, 0, 0, 0] - kirsp6d * ((xIrsGrb2 - xIrsGrb2b)/xIrsGrb2) * IRS[0, 0, P, P, 0, 0, P]$$
$$r429 = kirsp6 * IRS[0, 0, P, P, 0, P, 0] - kirsp6d * ((xIrsGrb2 - xIrsGrb2b)/xIrsGrb2) * IRS[0, 0, P, P, 0, P, P]$$
$$r430 = kirsp6 * IRS[0, 0, P, P, P, 0, 0] - kirsp6d * ((xIrsGrb2 - xIrsGrb2b)/xIrsGrb2) * IRS[0, 0, P, P, P, 0, P]$$
$$r431 = kirsp6 * IRS[0, 0, P, P, P, P, 0] - kirsp6d * ((xIrsGrb2 - xIrsGrb2b)/xIrsGrb2) * IRS[0, 0, P, P, P, P, P]$$
$$r432 = kirsp6 * IRS[0, P, 0, 0, 0, 0, 0] - kirsp6d * ((xIrsGrb2 - xIrsGrb2b)/xIrsGrb2) * IRS[0, P, 0, 0, 0, 0, P]$$
$$r433 = kirsp6 * IRS[0, P, 0, 0, 0, P, 0] - kirsp6d * ((xIrsGrb2 - xIrsGrb2b)/xIrsGrb2) * IRS[0, P, 0, 0, 0, P, P]$$
$$r434 = kirsp6 * IRS[0, P, 0, 0, P, 0, 0] - kirsp6d * ((xIrsGrb2 - xIrsGrb2b)/xIrsGrb2) * IRS[0, P, 0, 0, P, 0, P]$$
$$r435 = kirsp6 * IRS[0, P, 0, 0, P, P, 0] - kirsp6d * ((xIrsGrb2 - xIrsGrb2b)/xIrsGrb2) * IRS[0, P, 0, 0, P, P, P]$$
$$r436 = kirsp6 * IRS[0, P, 0, P, 0, 0, 0] - kirsp6d * ((xIrsGrb2 - xIrsGrb2b)/xIrsGrb2) * IRS[0, P, 0, P, 0, 0, P]$$
$$r437 = kirsp6 * IRS[0, P, 0, P, 0, P, 0] - kirsp6d * ((xIrsGrb2 - xIrsGrb2b)/xIrsGrb2) * IRS[0, P, 0, P, 0, P, P]$$
$$r438 = kirsp6 * IRS[0, P, 0, P, P, 0, 0] - kirsp6d * ((xIrsGrb2 - xIrsGrb2b)/xIrsGrb2) * IRS[0, P, 0, P, P, 0, P]$$
$$r439 = kirsp6 * IRS[0, P, 0, P, P, P, 0] - kirsp6d * ((xIrsGrb2 - xIrsGrb2b)/xIrsGrb2) * IRS[0, P, 0, P, P, P, P]$$
$$r440 = kirsp6 * IRS[0, P, P, 0, 0, 0, 0] - kirsp6d * ((xIrsGrb2 - xIrsGrb2b)/xIrsGrb2) * IRS[0, P, P, 0, 0, 0, P]$$
$$r441 = kirsp6 * IRS[0, P, P, 0, 0, P, 0] - kirsp6d * ((xIrsGrb2 - xIrsGrb2b)/xIrsGrb2) * IRS[0, P, P, 0, 0, P, P]$$
$$r442 = kirsp6 * IRS[0, P, P, 0, P, 0, 0] - kirsp6d * ((xIrsGrb2 - xIrsGrb2b)/xIrsGrb2) * IRS[0, P, P, 0, P, 0, P]$$
$$r443 = kirsp6 * IRS[0, P, P, 0, P, P, 0] - kirsp6d * ((xIrsGrb2 - xIrsGrb2b)/xIrsGrb2) * IRS[0, P, P, 0, P, P, P]$$
$$r444 = kirsp6 * IRS[0, P, P, P, 0, 0, 0] - kirsp6d * ((xIrsGrb2 - xIrsGrb2b)/xIrsGrb2) * IRS[0, P, P, P, 0, 0, P]$$
$$r445 = kirsp6 * IRS[0, P, P, P, 0, P, 0] - kirsp6d * ((xIrsGrb2 - xIrsGrb2b)/xIrsGrb2) * IRS[0, P, P, P, 0, P, P]$$
$$r446 = kirsp6 * IRS[0, P, P, P, P, 0, 0] - kirsp6d * ((xIrsGrb2 - xIrsGrb2b)/xIrsGrb2) * IRS[0, P, P, P, P, 0, P]$$
$$r447 = kirsp6 * IRS[0, P, P, P, P, P, 0] - kirsp6d * ((xIrsGrb2 - xIrsGrb2b)/xIrsGrb2) * IRS[0, P, P, P, P, P, P]$$
$$r448 = kirsbp1 * IRS[R, 0, 0, 0, 0, 0, 0] - kirsbp1d * ((xIrsPi3k1 - xIrsPi3k1b)/xIrsPi3k1) * IRS[R, P, 0, 0, 0, 0, 0]$$
$$r449 = kirsbp1 * IRS[R, 0, 0, 0, 0, 0, P] - kirsbp1d * ((xIrsPi3k1 - xIrsPi3k1b)/xIrsPi3k1) * IRS[R, P, 0, 0, 0, 0, P]$$

$r450 = kirsbp1 * IRS[R,0,0,0,0,P,0] - kirsbp1d * ((xIrsPi3k1 - xIrsPi3k1b)/xIrsPi3k1) * IRS[R,P,0,0,0,P,0]$

$r451 = kirsbp1 * IRS[R,0,0,0,0,P,P] - kirsbp1d * ((xIrsPi3k1 - xIrsPi3k1b)/xIrsPi3k1) * IRS[R,P,0,0,0,P,P]$

$r452 = kirsbp1 * IRS[R,0,0,0,P,0,0] - kirsbp1d * ((xIrsPi3k1 - xIrsPi3k1b)/xIrsPi3k1) * IRS[R,P,0,0,P,0,0]$

$r453 = kirsbp1 * IRS[R,0,0,0,P,0,P] - kirsbp1d * ((xIrsPi3k1 - xIrsPi3k1b)/xIrsPi3k1) * IRS[R,P,0,0,P,0,P]$

$r454 = kirsbp1 * IRS[R,0,0,0,P,P,0] - kirsbp1d * ((xIrsPi3k1 - xIrsPi3k1b)/xIrsPi3k1) * IRS[R,P,0,0,P,P,0]$

$r455 = kirsbp1 * IRS[R,0,0,0,P,P,P] - kirsbp1d * ((xIrsPi3k1 - xIrsPi3k1b)/xIrsPi3k1) * IRS[R,P,0,0,P,P,P]$

$r456 = kirsbp1 * IRS[R,0,0,P,0,0,0] - kirsbp1d * ((xIrsPi3k1 - xIrsPi3k1b)/xIrsPi3k1) * IRS[R,P,0,P,0,0,0]$

$r457 = kirsbp1 * IRS[R,0,0,P,0,0,P] - kirsbp1d * ((xIrsPi3k1 - xIrsPi3k1b)/xIrsPi3k1) * IRS[R,P,0,P,0,0,P]$

$r458 = kirsbp1 * IRS[R,0,0,P,0,P,0] - kirsbp1d * ((xIrsPi3k1 - xIrsPi3k1b)/xIrsPi3k1) * IRS[R,P,0,P,0,P,0]$

$r459 = kirsbp1 * IRS[R,0,0,P,0,P,P] - kirsbp1d * ((xIrsPi3k1 - xIrsPi3k1b)/xIrsPi3k1) * IRS[R,P,0,P,0,P,P]$

$r460 = kirsbp1 * IRS[R,0,0,P,P,0,0] - kirsbp1d * ((xIrsPi3k1 - xIrsPi3k1b)/xIrsPi3k1) * IRS[R,P,0,P,P,0,0]$

$r461 = kirsbp1 * IRS[R,0,0,P,P,0,P] - kirsbp1d * ((xIrsPi3k1 - xIrsPi3k1b)/xIrsPi3k1) * IRS[R,P,0,P,P,0,P]$

$r462 = kirsbp1 * IRS[R,0,0,P,P,P,0] - kirsbp1d * ((xIrsPi3k1 - xIrsPi3k1b)/xIrsPi3k1) * IRS[R,P,0,P,P,P,0]$

$r463 = kirsbp1 * IRS[R,0,0,P,P,P,P] - kirsbp1d * ((xIrsPi3k1 - xIrsPi3k1b)/xIrsPi3k1) * IRS[R,P,0,P,P,P,P]$

$r464 = kirsbp1 * IRS[R,0,P,0,0,0,0] - kirsbp1d * ((xIrsPi3k1 - xIrsPi3k1b)/xIrsPi3k1) * IRS[R,P,P,0,0,0,0]$

$r465 = kirsbp1 * IRS[R,0,P,0,0,0,P] - kirsbp1d * ((xIrsPi3k1 - xIrsPi3k1b)/xIrsPi3k1) * IRS[R,P,P,0,0,0,P]$

$r466 = kirsbp1 * IRS[R,0,P,0,0,P,0] - kirsbp1d * ((xIrsPi3k1 - xIrsPi3k1b)/xIrsPi3k1) * IRS[R,P,P,0,0,P,0]$

$r467 = kirsbp1 * IRS[R,0,P,0,0,P,P] - kirsbp1d * ((xIrsPi3k1 - xIrsPi3k1b)/xIrsPi3k1) * IRS[R,P,P,0,0,P,P]$

$r468 = kirsbp1 * IRS[R,0,P,0,P,0,0] - kirsbp1d * ((xIrsPi3k1 - xIrsPi3k1b)/xIrsPi3k1) * IRS[R,P,P,0,P,0,0]$

$r469 = kirsbp1 * IRS[R,0,P,0,P,0,P] - kirsbp1d * ((xIrsPi3k1 - xIrsPi3k1b)/xIrsPi3k1) * IRS[R,P,P,0,P,0,P]$

$r470 = kirsbp1 * IRS[R,0,P,0,P,P,0] - kirsbp1d * ((xIrsPi3k1 - xIrsPi3k1b)/xIrsPi3k1) * IRS[R,P,P,0,P,P,0]$

$r471 = kirsbp1 * IRS[R,0,P,0,P,P,P] - kirsbp1d * ((xIrsPi3k1 - xIrsPi3k1b)/xIrsPi3k1) * IRS[R,P,P,0,P,P,P]$

$r472 = kirsbp1 * IRS[R,0,P,P,0,0,0] - kirsbp1d * ((xIrsPi3k1 - xIrsPi3k1b)/xIrsPi3k1) * IRS[R,P,P,P,0,0,0]$

$r473 = kirsbp1 * IRS[R,0,P,P,0,0,P] - kirsbp1d * ((xIrsPi3k1 - xIrsPi3k1b)/xIrsPi3k1) * IRS[R,P,P,P,0,0,P]$

$r474 = kirsbp1 * IRS[R,0,P,P,0,P,0] - kirsbp1d * ((xIrsPi3k1 - xIrsPi3k1b)/xIrsPi3k1) * IRS[R,P,P,P,0,P,0]$

$r475 = kirsbp1 * IRS[R,0,P,P,0,P,P] - kirsbp1d * ((xIrsPi3k1 - xIrsPi3k1b)/xIrsPi3k1) * IRS[R,P,P,P,0,P,P]$

$r476 = kirsbp1 * IRS[R,0,P,P,P,0,0] - kirsbp1d * ((xIrsPi3k1 - xIrsPi3k1b)/xIrsPi3k1) * IRS[R,P,P,P,P,0,0]$

$r477 = kirsbp1 * IRS[R,0,P,P,P,0,P] - kirsbp1d * ((xIrsPi3k1 - xIrsPi3k1b)/xIrsPi3k1) * IRS[R,P,P,P,P,0,P]$

$r478 = kirsbp1 * IRS[R,0,P,P,P,P,0] - kirsbp1d * ((xIrsPi3k1 - xIrsPi3k1b)/xIrsPi3k1) * IRS[R,P,P,P,P,P,0]$

$r479 = kirsbp1 * IRS[R,0,P,P,P,P,P] - kirsbp1d * ((xIrsPi3k1 - xIrsPi3k1b)/xIrsPi3k1) * IRS[R,P,P,P,P,P,P]$

$r480 = kirsbp2 * IRS[R,0,0,0,0,0,0] - kirsbp2d * ((xIrsPi3k2 - xIrsPi3k2b)/xIrsPi3k2) * IRS[R,0,P,0,0,0,0]$

$r481 = kirsbp2 * IRS[R,0,0,0,0,0,P] - kirsbp2d * ((xIrsPi3k2 - xIrsPi3k2b)/xIrsPi3k2) * IRS[R,0,P,0,0,0,P]$

$r482 = kirsbp2 * IRS[R,0,0,0,0,P,0] - kirsbp2d * ((xIrsPi3k2 - xIrsPi3k2b)/xIrsPi3k2) * IRS[R,0,P,0,0,P,0]$

$r483 = kirsbp2 * IRS[R,0,0,0,0,P,P] - kirsbp2d * ((xIrsPi3k2 - xIrsPi3k2b)/xIrsPi3k2) * IRS[R,0,P,0,0,P,P]$

$r484 = kirsbp2 * IRS[R,0,0,0,P,0,0] - kirsbp2d * ((xIrsPi3k2 - xIrsPi3k2b)/xIrsPi3k2) * IRS[R,0,P,0,P,0,0]$

$r485 = kirsbp2 * IRS[R,0,0,0,P,0,P] - kirsbp2d * ((xIrsPi3k2 - xIrsPi3k2b)/xIrsPi3k2) * IRS[R,0,P,0,P,0,P]$

$r486 = kirsbp2 * IRS[R,0,0,0,P,P,0] - kirsbp2d * ((xIrsPi3k2 - xIrsPi3k2b)/xIrsPi3k2) * IRS[R,0,P,0,P,P,0]$

$r487 = kirsbp2 * IRS[R,0,0,0,P,P,P] - kirsbp2d * ((xIrsPi3k2 - xIrsPi3k2b)/xIrsPi3k2) * IRS[R,0,P,0,P,P,P]$

$r488 = kirsbp2 * IRS[R,0,0,P,0,0,0] - kirsbp2d * ((xIrsPi3k2 - xIrsPi3k2b)/xIrsPi3k2) * IRS[R,0,P,P,0,0,0]$

$r489 = kirsbp2 * IRS[R,0,0,P,0,0,P] - kirsbp2d * ((xIrsPi3k2 - xIrsPi3k2b)/xIrsPi3k2) * IRS[R,0,P,P,0,0,P]$

$r490 = kirsbp2 * IRS[R,0,0,P,0,P,0] - kirsbp2d * ((xIrsPi3k2 - xIrsPi3k2b)/xIrsPi3k2) * IRS[R,0,P,P,0,P,0]$

$r491 = kirsbp2 * IRS[R,0,0,P,0,P,P] - kirsbp2d * ((xIrsPi3k2 - xIrsPi3k2b)/xIrsPi3k2) * IRS[R,0,P,P,0,P,P]$

$r492 = kirsbp2 * IRS[R,0,0,P,P,0,0] - kirsbp2d * ((xIrsPi3k2 - xIrsPi3k2b)/xIrsPi3k2) * IRS[R,0,P,P,P,0,0]$

$r493 = kirsbp2 * IRS[R,0,0,P,P,0,P] - kirsbp2d * ((xIrsPi3k2 - xIrsPi3k2b)/xIrsPi3k2) * IRS[R,0,P,P,P,0,P]$

$r494 = kirsbp2 * IRS[R,0,0,P,P,P,0] - kirsbp2d * ((xIrsPi3k2 - xIrsPi3k2b)/xIrsPi3k2) * IRS[R,0,P,P,P,P,0]$

$$r495 = kirsbp2 * IRS[R,0,0,P,P,P,P] - kirsbp2d * ((xIrsPi3k2 - xIrsPi3k2b)/xIrsPi3k2) * IRS[R,0,P,P,P,P,P]$$
$$r496 = kirsbp2 * IRS[R,P,0,0,0,0,0] - kirsbp2d * ((xIrsPi3k2 - xIrsPi3k2b)/xIrsPi3k2) * IRS[R,P,P,0,0,0,0]$$
$$r497 = kirsbp2 * IRS[R,P,0,0,0,0,P] - kirsbp2d * ((xIrsPi3k2 - xIrsPi3k2b)/xIrsPi3k2) * IRS[R,P,P,0,0,0,P]$$
$$r498 = kirsbp2 * IRS[R,P,0,0,0,P,0] - kirsbp2d * ((xIrsPi3k2 - xIrsPi3k2b)/xIrsPi3k2) * IRS[R,P,P,0,0,P,0]$$
$$r499 = kirsbp2 * IRS[R,P,0,0,0,P,P] - kirsbp2d * ((xIrsPi3k2 - xIrsPi3k2b)/xIrsPi3k2) * IRS[R,P,P,0,0,P,P]$$
$$r500 = kirsbp2 * IRS[R,P,0,0,P,0,0] - kirsbp2d * ((xIrsPi3k2 - xIrsPi3k2b)/xIrsPi3k2) * IRS[R,P,P,0,P,0,0]$$
$$r501 = kirsbp2 * IRS[R,P,0,0,P,0,P] - kirsbp2d * ((xIrsPi3k2 - xIrsPi3k2b)/xIrsPi3k2) * IRS[R,P,P,0,P,0,P]$$
$$r502 = kirsbp2 * IRS[R,P,0,0,P,P,0] - kirsbp2d * ((xIrsPi3k2 - xIrsPi3k2b)/xIrsPi3k2) * IRS[R,P,P,0,P,P,0]$$
$$r503 = kirsbp2 * IRS[R,P,0,0,P,P,P] - kirsbp2d * ((xIrsPi3k2 - xIrsPi3k2b)/xIrsPi3k2) * IRS[R,P,P,0,P,P,P]$$
$$r504 = kirsbp2 * IRS[R,P,0,P,0,0,0] - kirsbp2d * ((xIrsPi3k2 - xIrsPi3k2b)/xIrsPi3k2) * IRS[R,P,P,P,0,0,0]$$
$$r505 = kirsbp2 * IRS[R,P,0,P,0,0,P] - kirsbp2d * ((xIrsPi3k2 - xIrsPi3k2b)/xIrsPi3k2) * IRS[R,P,P,P,0,0,P]$$
$$r506 = kirsbp2 * IRS[R,P,0,P,0,P,0] - kirsbp2d * ((xIrsPi3k2 - xIrsPi3k2b)/xIrsPi3k2) * IRS[R,P,P,P,0,P,0]$$
$$r507 = kirsbp2 * IRS[R,P,0,P,0,P,P] - kirsbp2d * ((xIrsPi3k2 - xIrsPi3k2b)/xIrsPi3k2) * IRS[R,P,P,P,0,P,P]$$
$$r508 = kirsbp2 * IRS[R,P,0,P,P,0,0] - kirsbp2d * ((xIrsPi3k2 - xIrsPi3k2b)/xIrsPi3k2) * IRS[R,P,P,P,P,0,0]$$
$$r509 = kirsbp2 * IRS[R,P,0,P,P,0,P] - kirsbp2d * ((xIrsPi3k2 - xIrsPi3k2b)/xIrsPi3k2) * IRS[R,P,P,P,P,0,P]$$
$$r510 = kirsbp2 * IRS[R,P,0,P,P,P,0] - kirsbp2d * ((xIrsPi3k2 - xIrsPi3k2b)/xIrsPi3k2) * IRS[R,P,P,P,P,P,0]$$
$$r511 = kirsbp2 * IRS[R,P,0,P,P,P,P] - kirsbp2d * ((xIrsPi3k2 - xIrsPi3k2b)/xIrsPi3k2) * IRS[R,P,P,P,P,P,P]$$
$$r512 = kirsbp3 * IRS[R,0,0,0,0,0,0] - kirsbp3d * ((xIrsPi3k3 - xIrsPi3k3b)/xIrsPi3k3) * IRS[R,0,0,P,0,0,0]$$
$$r513 = kirsbp3 * IRS[R,0,0,0,0,0,P] - kirsbp3d * ((xIrsPi3k3 - xIrsPi3k3b)/xIrsPi3k3) * IRS[R,0,0,P,0,0,P]$$
$$r514 = kirsbp3 * IRS[R,0,0,0,0,P,0] - kirsbp3d * ((xIrsPi3k3 - xIrsPi3k3b)/xIrsPi3k3) * IRS[R,0,0,P,0,P,0]$$
$$r515 = kirsbp3 * IRS[R,0,0,0,0,P,P] - kirsbp3d * ((xIrsPi3k3 - xIrsPi3k3b)/xIrsPi3k3) * IRS[R,0,0,P,0,P,P]$$
$$r516 = kirsbp3 * IRS[R,0,0,0,P,0,0] - kirsbp3d * ((xIrsPi3k3 - xIrsPi3k3b)/xIrsPi3k3) * IRS[R,0,0,P,P,0,0]$$
$$r517 = kirsbp3 * IRS[R,0,0,0,P,0,P] - kirsbp3d * ((xIrsPi3k3 - xIrsPi3k3b)/xIrsPi3k3) * IRS[R,0,0,P,P,0,P]$$
$$r518 = kirsbp3 * IRS[R,0,0,0,P,P,0] - kirsbp3d * ((xIrsPi3k3 - xIrsPi3k3b)/xIrsPi3k3) * IRS[R,0,0,P,P,P,0]$$
$$r519 = kirsbp3 * IRS[R,0,0,0,P,P,P] - kirsbp3d * ((xIrsPi3k3 - xIrsPi3k3b)/xIrsPi3k3) * IRS[R,0,0,P,P,P,P]$$
$$r520 = kirsbp3 * IRS[R,0,P,0,0,0,0] - kirsbp3d * ((xIrsPi3k3 - xIrsPi3k3b)/xIrsPi3k3) * IRS[R,0,P,P,0,0,0]$$
$$r521 = kirsbp3 * IRS[R,0,P,0,0,0,P] - kirsbp3d * ((xIrsPi3k3 - xIrsPi3k3b)/xIrsPi3k3) * IRS[R,0,P,P,0,0,P]$$
$$r522 = kirsbp3 * IRS[R,0,P,0,0,P,0] - kirsbp3d * ((xIrsPi3k3 - xIrsPi3k3b)/xIrsPi3k3) * IRS[R,0,P,P,0,P,0]$$
$$r523 = kirsbp3 * IRS[R,0,P,0,0,P,P] - kirsbp3d * ((xIrsPi3k3 - xIrsPi3k3b)/xIrsPi3k3) * IRS[R,0,P,P,0,P,P]$$
$$r524 = kirsbp3 * IRS[R,0,P,0,P,0,0] - kirsbp3d * ((xIrsPi3k3 - xIrsPi3k3b)/xIrsPi3k3) * IRS[R,0,P,P,P,0,0]$$
$$r525 = kirsbp3 * IRS[R,0,P,0,P,0,P] - kirsbp3d * ((xIrsPi3k3 - xIrsPi3k3b)/xIrsPi3k3) * IRS[R,0,P,P,P,0,P]$$
$$r526 = kirsbp3 * IRS[R,0,P,0,P,P,0] - kirsbp3d * ((xIrsPi3k3 - xIrsPi3k3b)/xIrsPi3k3) * IRS[R,0,P,P,P,P,0]$$
$$r527 = kirsbp3 * IRS[R,0,P,0,P,P,P] - kirsbp3d * ((xIrsPi3k3 - xIrsPi3k3b)/xIrsPi3k3) * IRS[R,0,P,P,P,P,P]$$
$$r528 = kirsbp3 * IRS[R,P,0,0,0,0,0] - kirsbp3d * ((xIrsPi3k3 - xIrsPi3k3b)/xIrsPi3k3) * IRS[R,P,0,P,0,0,0]$$
$$r529 = kirsbp3 * IRS[R,P,0,0,0,0,P] - kirsbp3d * ((xIrsPi3k3 - xIrsPi3k3b)/xIrsPi3k3) * IRS[R,P,0,P,0,0,P]$$
$$r530 = kirsbp3 * IRS[R,P,0,0,0,P,0] - kirsbp3d * ((xIrsPi3k3 - xIrsPi3k3b)/xIrsPi3k3) * IRS[R,P,0,P,0,P,0]$$
$$r531 = kirsbp3 * IRS[R,P,0,0,0,P,P] - kirsbp3d * ((xIrsPi3k3 - xIrsPi3k3b)/xIrsPi3k3) * IRS[R,P,0,P,0,P,P]$$
$$r532 = kirsbp3 * IRS[R,P,0,0,P,0,0] - kirsbp3d * ((xIrsPi3k3 - xIrsPi3k3b)/xIrsPi3k3) * IRS[R,P,0,P,P,0,0]$$
$$r533 = kirsbp3 * IRS[R,P,0,0,P,0,P] - kirsbp3d * ((xIrsPi3k3 - xIrsPi3k3b)/xIrsPi3k3) * IRS[R,P,0,P,P,0,P]$$
$$r534 = kirsbp3 * IRS[R,P,0,0,P,P,0] - kirsbp3d * ((xIrsPi3k3 - xIrsPi3k3b)/xIrsPi3k3) * IRS[R,P,0,P,P,P,0]$$
$$r535 = kirsbp3 * IRS[R,P,0,0,P,P,P] - kirsbp3d * ((xIrsPi3k3 - xIrsPi3k3b)/xIrsPi3k3) * IRS[R,P,0,P,P,P,P]$$
$$r536 = kirsbp3 * IRS[R,P,P,0,0,0,0] - kirsbp3d * ((xIrsPi3k3 - xIrsPi3k3b)/xIrsPi3k3) * IRS[R,P,P,P,0,0,0]$$
$$r537 = kirsbp3 * IRS[R,P,P,0,0,0,P] - kirsbp3d * ((xIrsPi3k3 - xIrsPi3k3b)/xIrsPi3k3) * IRS[R,P,P,P,0,0,P]$$
$$r538 = kirsbp3 * IRS[R,P,P,0,0,P,0] - kirsbp3d * ((xIrsPi3k3 - xIrsPi3k3b)/xIrsPi3k3) * IRS[R,P,P,P,0,P,0]$$
$$r539 = kirsbp3 * IRS[R,P,P,0,0,P,P] - kirsbp3d * ((xIrsPi3k3 - xIrsPi3k3b)/xIrsPi3k3) * IRS[R,P,P,P,0,P,P]$$

$r540 = kirsbp3 * IRS[R, P, P, 0, P, 0, 0] - kirsbp3d * ((xIrsPi3k3 - xIrsPi3k3b)/xIrsPi3k3) * IRS[R, P, P, P, P, 0, 0]$

$r541 = kirsbp3 * IRS[R, P, P, 0, P, 0, P] - kirsbp3d * ((xIrsPi3k3 - xIrsPi3k3b)/xIrsPi3k3) * IRS[R, P, P, P, P, 0, P]$

$r542 = kirsbp3 * IRS[R, P, P, 0, P, P, 0] - kirsbp3d * ((xIrsPi3k3 - xIrsPi3k3b)/xIrsPi3k3) * IRS[R, P, P, P, P, P, 0]$

$r543 = kirsbp3 * IRS[R, P, P, 0, P, P, P] - kirsbp3d * ((xIrsPi3k3 - xIrsPi3k3b)/xIrsPi3k3) * IRS[R, P, P, P, P, P, P]$

$r544 = kirsbp4 * IRS[R, 0, 0, 0, 0, 0, 0] - kirsbp4d * ((xIrsPi3k4 - xIrsPi3k4b)/xIrsPi3k4) * IRS[R, 0, 0, 0, P, 0, 0]$

$r545 = kirsbp4 * IRS[R, 0, 0, 0, 0, 0, P] - kirsbp4d * ((xIrsPi3k4 - xIrsPi3k4b)/xIrsPi3k4) * IRS[R, 0, 0, 0, P, 0, P]$

$r546 = kirsbp4 * IRS[R, 0, 0, 0, 0, P, 0] - kirsbp4d * ((xIrsPi3k4 - xIrsPi3k4b)/xIrsPi3k4) * IRS[R, 0, 0, 0, P, P, 0]$

$r547 = kirsbp4 * IRS[R, 0, 0, 0, 0, P, P] - kirsbp4d * ((xIrsPi3k4 - xIrsPi3k4b)/xIrsPi3k4) * IRS[R, 0, 0, 0, P, P, P]$

$r548 = kirsbp4 * IRS[R, 0, 0, P, 0, 0, 0] - kirsbp4d * ((xIrsPi3k4 - xIrsPi3k4b)/xIrsPi3k4) * IRS[R, 0, 0, P, P, 0, 0]$

$r549 = kirsbp4 * IRS[R, 0, 0, P, 0, 0, P] - kirsbp4d * ((xIrsPi3k4 - xIrsPi3k4b)/xIrsPi3k4) * IRS[R, 0, 0, P, P, 0, P]$

$r550 = kirsbp4 * IRS[R, 0, 0, P, 0, P, 0] - kirsbp4d * ((xIrsPi3k4 - xIrsPi3k4b)/xIrsPi3k4) * IRS[R, 0, 0, P, P, P, 0]$

$r551 = kirsbp4 * IRS[R, 0, 0, P, 0, P, P] - kirsbp4d * ((xIrsPi3k4 - xIrsPi3k4b)/xIrsPi3k4) * IRS[R, 0, 0, P, P, P, P]$

$r552 = kirsbp4 * IRS[R, 0, P, 0, 0, 0, 0] - kirsbp4d * ((xIrsPi3k4 - xIrsPi3k4b)/xIrsPi3k4) * IRS[R, 0, P, 0, P, 0, 0]$

$r553 = kirsbp4 * IRS[R, 0, P, 0, 0, 0, P] - kirsbp4d * ((xIrsPi3k4 - xIrsPi3k4b)/xIrsPi3k4) * IRS[R, 0, P, 0, P, 0, P]$

$r554 = kirsbp4 * IRS[R, 0, P, 0, 0, P, 0] - kirsbp4d * ((xIrsPi3k4 - xIrsPi3k4b)/xIrsPi3k4) * IRS[R, 0, P, 0, P, P, 0]$

$r555 = kirsbp4 * IRS[R, 0, P, 0, 0, P, P] - kirsbp4d * ((xIrsPi3k4 - xIrsPi3k4b)/xIrsPi3k4) * IRS[R, 0, P, 0, P, P, P]$

$r556 = kirsbp4 * IRS[R, 0, P, P, 0, 0, 0] - kirsbp4d * ((xIrsPi3k4 - xIrsPi3k4b)/xIrsPi3k4) * IRS[R, 0, P, P, P, 0, 0]$

$r557 = kirsbp4 * IRS[R, 0, P, P, 0, 0, P] - kirsbp4d * ((xIrsPi3k4 - xIrsPi3k4b)/xIrsPi3k4) * IRS[R, 0, P, P, P, 0, P]$

$r558 = kirsbp4 * IRS[R, 0, P, P, 0, P, 0] - kirsbp4d * ((xIrsPi3k4 - xIrsPi3k4b)/xIrsPi3k4) * IRS[R, 0, P, P, P, P, 0]$

$r559 = kirsbp4 * IRS[R, 0, P, P, 0, P, P] - kirsbp4d * ((xIrsPi3k4 - xIrsPi3k4b)/xIrsPi3k4) * IRS[R, 0, P, P, P, P, P]$

$r560 = kirsbp4 * IRS[R, P, 0, 0, 0, 0, 0] - kirsbp4d * ((xIrsPi3k4 - xIrsPi3k4b)/xIrsPi3k4) * IRS[R, P, 0, 0, P, 0, 0]$

$r561 = kirsbp4 * IRS[R, P, 0, 0, 0, 0, P] - kirsbp4d * ((xIrsPi3k4 - xIrsPi3k4b)/xIrsPi3k4) * IRS[R, P, 0, 0, P, 0, P]$

$r562 = kirsbp4 * IRS[R, P, 0, 0, 0, P, 0] - kirsbp4d * ((xIrsPi3k4 - xIrsPi3k4b)/xIrsPi3k4) * IRS[R, P, 0, 0, P, P, 0]$

$r563 = kirsbp4 * IRS[R, P, 0, 0, 0, P, P] - kirsbp4d * ((xIrsPi3k4 - xIrsPi3k4b)/xIrsPi3k4) * IRS[R, P, 0, 0, P, P, P]$

$r564 = kirsbp4 * IRS[R, P, 0, P, 0, 0, 0] - kirsbp4d * ((xIrsPi3k4 - xIrsPi3k4b)/xIrsPi3k4) * IRS[R, P, 0, P, P, 0, 0]$

$r565 = kirsbp4 * IRS[R, P, 0, P, 0, 0, P] - kirsbp4d * ((xIrsPi3k4 - xIrsPi3k4b)/xIrsPi3k4) * IRS[R, P, 0, P, P, 0, P]$

$r566 = kirsbp4 * IRS[R, P, 0, P, 0, P, 0] - kirsbp4d * ((xIrsPi3k4 - xIrsPi3k4b)/xIrsPi3k4) * IRS[R, P, 0, P, P, P, 0]$

$r567 = kirsbp4 * IRS[R, P, 0, P, 0, P, P] - kirsbp4d * ((xIrsPi3k4 - xIrsPi3k4b)/xIrsPi3k4) * IRS[R, P, 0, P, P, P, P]$

$r568 = kirsbp4 * IRS[R, P, P, 0, 0, 0, 0] - kirsbp4d * ((xIrsPi3k4 - xIrsPi3k4b)/xIrsPi3k4) * IRS[R, P, P, 0, P, 0, 0]$

$r569 = kirsbp4 * IRS[R, P, P, 0, 0, 0, P] - kirsbp4d * ((xIrsPi3k4 - xIrsPi3k4b)/xIrsPi3k4) * IRS[R, P, P, 0, P, 0, P]$

$r570 = kirsbp4 * IRS[R, P, P, 0, 0, P, 0] - kirsbp4d * ((xIrsPi3k4 - xIrsPi3k4b)/xIrsPi3k4) * IRS[R, P, P, 0, P, P, 0]$

$r571 = kirsbp4 * IRS[R, P, P, 0, 0, P, P] - kirsbp4d * ((xIrsPi3k4 - xIrsPi3k4b)/xIrsPi3k4) * IRS[R, P, P, 0, P, P, P]$

$r572 = kirsbp4 * IRS[R, P, P, P, 0, 0, 0] - kirsbp4d * ((xIrsPi3k4 - xIrsPi3k4b)/xIrsPi3k4) * IRS[R, P, P, P, P, 0, 0]$

$r573 = kirsbp4 * IRS[R, P, P, P, 0, 0, P] - kirsbp4d * ((xIrsPi3k4 - xIrsPi3k4b)/xIrsPi3k4) * IRS[R, P, P, P, P, 0, P]$

$r574 = kirsbp4 * IRS[R, P, P, P, 0, P, 0] - kirsbp4d * ((xIrsPi3k4 - xIrsPi3k4b)/xIrsPi3k4) * IRS[R, P, P, P, P, P, 0]$

$r575 = kirsbp4 * IRS[R, P, P, P, 0, P, P] - kirsbp4d * ((xIrsPi3k4 - xIrsPi3k4b)/xIrsPi3k4) * IRS[R, P, P, P, P, P, P]$

$r576 = kirsbp5 * IRS[R, 0, 0, 0, 0, 0, 0] - kirsbp5d * ((xIrsShp2 - xIrsShp2b)/xIrsShp2) * IRS[R, 0, 0, 0, 0, P, 0]$

$r577 = kirsbp5 * IRS[R, 0, 0, 0, 0, 0, P] - kirsbp5d * ((xIrsShp2 - xIrsShp2b)/xIrsShp2) * IRS[R, 0, 0, 0, 0, P, P]$

$r578 = kirsbp5 * IRS[R, 0, 0, 0, P, 0, 0] - kirsbp5d * ((xIrsShp2 - xIrsShp2b)/xIrsShp2) * IRS[R, 0, 0, 0, P, P, 0]$

$r579 = kirsbp5 * IRS[R, 0, 0, 0, P, 0, P] - kirsbp5d * ((xIrsShp2 - xIrsShp2b)/xIrsShp2) * IRS[R, 0, 0, 0, P, P, P]$

$r580 = kirsbp5 * IRS[R, 0, 0, P, 0, 0, 0] - kirsbp5d * ((xIrsShp2 - xIrsShp2b)/xIrsShp2) * IRS[R, 0, 0, P, 0, P, 0]$

$r581 = kirsbp5 * IRS[R, 0, 0, P, 0, 0, P] - kirsbp5d * ((xIrsShp2 - xIrsShp2b)/xIrsShp2) * IRS[R, 0, 0, P, 0, P, P]$

$r582 = kirsbp5 * IRS[R, 0, 0, P, P, 0, 0] - kirsbp5d * ((xIrsShp2 - xIrsShp2b)/xIrsShp2) * IRS[R, 0, 0, P, P, P, 0]$

$r583 = kirsbp5 * IRS[R, 0, 0, P, P, 0, P] - kirsbp5d * ((xIrsShp2 - xIrsShp2b)/xIrsShp2) * IRS[R, 0, 0, P, P, P, P]$

$r584 = kirsbp5 * IRS[R, 0, P, 0, 0, 0, 0] - kirsbp5d * ((xIrsShp2 - xIrsShp2b)/xIrsShp2) * IRS[R, 0, P, 0, 0, P, 0]$

$r585 = kirsbp5 * IRS[R, 0, P, 0, 0, 0, P] - kirsbp5d * ((xIrsShp2 - xIrsShp2b)/xIrsShp2) * IRS[R, 0, P, 0, 0, P, P]$
$r586 = kirsbp5 * IRS[R, 0, P, 0, P, 0, 0] - kirsbp5d * ((xIrsShp2 - xIrsShp2b)/xIrsShp2) * IRS[R, 0, P, 0, P, P, 0]$
$r587 = kirsbp5 * IRS[R, 0, P, 0, P, 0, P] - kirsbp5d * ((xIrsShp2 - xIrsShp2b)/xIrsShp2) * IRS[R, 0, P, 0, P, P, P]$
$r588 = kirsbp5 * IRS[R, 0, P, P, 0, 0, 0] - kirsbp5d * ((xIrsShp2 - xIrsShp2b)/xIrsShp2) * IRS[R, 0, P, P, 0, P, 0]$
$r589 = kirsbp5 * IRS[R, 0, P, P, 0, 0, P] - kirsbp5d * ((xIrsShp2 - xIrsShp2b)/xIrsShp2) * IRS[R, 0, P, P, 0, P, P]$
$r590 = kirsbp5 * IRS[R, 0, P, P, P, 0, 0] - kirsbp5d * ((xIrsShp2 - xIrsShp2b)/xIrsShp2) * IRS[R, 0, P, P, P, P, 0]$
$r591 = kirsbp5 * IRS[R, 0, P, P, P, 0, P] - kirsbp5d * ((xIrsShp2 - xIrsShp2b)/xIrsShp2) * IRS[R, 0, P, P, P, P, P]$
$r592 = kirsbp5 * IRS[R, P, 0, 0, 0, 0, 0] - kirsbp5d * ((xIrsShp2 - xIrsShp2b)/xIrsShp2) * IRS[R, P, 0, 0, 0, P, 0]$
$r593 = kirsbp5 * IRS[R, P, 0, 0, 0, 0, P] - kirsbp5d * ((xIrsShp2 - xIrsShp2b)/xIrsShp2) * IRS[R, P, 0, 0, 0, P, P]$
$r594 = kirsbp5 * IRS[R, P, 0, 0, P, 0, 0] - kirsbp5d * ((xIrsShp2 - xIrsShp2b)/xIrsShp2) * IRS[R, P, 0, 0, P, P, 0]$
$r595 = kirsbp5 * IRS[R, P, 0, 0, P, 0, P] - kirsbp5d * ((xIrsShp2 - xIrsShp2b)/xIrsShp2) * IRS[R, P, 0, 0, P, P, P]$
$r596 = kirsbp5 * IRS[R, P, 0, P, 0, 0, 0] - kirsbp5d * ((xIrsShp2 - xIrsShp2b)/xIrsShp2) * IRS[R, P, 0, P, 0, P, 0]$
$r597 = kirsbp5 * IRS[R, P, 0, P, 0, 0, P] - kirsbp5d * ((xIrsShp2 - xIrsShp2b)/xIrsShp2) * IRS[R, P, 0, P, 0, P, P]$
$r598 = kirsbp5 * IRS[R, P, 0, P, P, 0, 0] - kirsbp5d * ((xIrsShp2 - xIrsShp2b)/xIrsShp2) * IRS[R, P, 0, P, P, P, 0]$
$r599 = kirsbp5 * IRS[R, P, 0, P, P, 0, P] - kirsbp5d * ((xIrsShp2 - xIrsShp2b)/xIrsShp2) * IRS[R, P, 0, P, P, P, P]$
$r600 = kirsbp5 * IRS[R, P, P, 0, 0, 0, 0] - kirsbp5d * ((xIrsShp2 - xIrsShp2b)/xIrsShp2) * IRS[R, P, P, 0, 0, P, 0]$
$r601 = kirsbp5 * IRS[R, P, P, 0, 0, 0, P] - kirsbp5d * ((xIrsShp2 - xIrsShp2b)/xIrsShp2) * IRS[R, P, P, 0, 0, P, P]$
$r602 = kirsbp5 * IRS[R, P, P, 0, P, 0, 0] - kirsbp5d * ((xIrsShp2 - xIrsShp2b)/xIrsShp2) * IRS[R, P, P, 0, P, P, 0]$
$r603 = kirsbp5 * IRS[R, P, P, 0, P, 0, P] - kirsbp5d * ((xIrsShp2 - xIrsShp2b)/xIrsShp2) * IRS[R, P, P, 0, P, P, P]$
$r604 = kirsbp5 * IRS[R, P, P, P, 0, 0, 0] - kirsbp5d * ((xIrsShp2 - xIrsShp2b)/xIrsShp2) * IRS[R, P, P, P, 0, P, 0]$
$r605 = kirsbp5 * IRS[R, P, P, P, 0, 0, P] - kirsbp5d * ((xIrsShp2 - xIrsShp2b)/xIrsShp2) * IRS[R, P, P, P, 0, P, P]$
$r606 = kirsbp5 * IRS[R, P, P, P, P, 0, 0] - kirsbp5d * ((xIrsShp2 - xIrsShp2b)/xIrsShp2) * IRS[R, P, P, P, P, P, 0]$
$r607 = kirsbp5 * IRS[R, P, P, P, P, 0, P] - kirsbp5d * ((xIrsShp2 - xIrsShp2b)/xIrsShp2) * IRS[R, P, P, P, P, P, P]$
$r608 = kirsbp6 * IRS[R, 0, 0, 0, 0, 0, 0] - kirsbp6d * ((xIrsGrb2 - xIrsGrb2b)/xIrsGrb2) * IRS[R, 0, 0, 0, 0, 0, P]$
$r609 = kirsbp6 * IRS[R, 0, 0, 0, 0, P, 0] - kirsbp6d * ((xIrsGrb2 - xIrsGrb2b)/xIrsGrb2) * IRS[R, 0, 0, 0, 0, P, P]$
$r610 = kirsbp6 * IRS[R, 0, 0, 0, P, 0, 0] - kirsbp6d * ((xIrsGrb2 - xIrsGrb2b)/xIrsGrb2) * IRS[R, 0, 0, 0, P, 0, P]$
$r611 = kirsbp6 * IRS[R, 0, 0, 0, P, P, 0] - kirsbp6d * ((xIrsGrb2 - xIrsGrb2b)/xIrsGrb2) * IRS[R, 0, 0, 0, P, P, P]$
$r612 = kirsbp6 * IRS[R, 0, 0, P, 0, 0, 0] - kirsbp6d * ((xIrsGrb2 - xIrsGrb2b)/xIrsGrb2) * IRS[R, 0, 0, P, 0, 0, P]$
$r613 = kirsbp6 * IRS[R, 0, 0, P, 0, P, 0] - kirsbp6d * ((xIrsGrb2 - xIrsGrb2b)/xIrsGrb2) * IRS[R, 0, 0, P, 0, P, P]$
$r614 = kirsbp6 * IRS[R, 0, 0, P, P, 0, 0] - kirsbp6d * ((xIrsGrb2 - xIrsGrb2b)/xIrsGrb2) * IRS[R, 0, 0, P, P, 0, P]$
$r615 = kirsbp6 * IRS[R, 0, 0, P, P, P, 0] - kirsbp6d * ((xIrsGrb2 - xIrsGrb2b)/xIrsGrb2) * IRS[R, 0, 0, P, P, P, P]$
$r616 = kirsbp6 * IRS[R, 0, P, 0, 0, 0, 0] - kirsbp6d * ((xIrsGrb2 - xIrsGrb2b)/xIrsGrb2) * IRS[R, 0, P, 0, 0, 0, P]$
$r617 = kirsbp6 * IRS[R, 0, P, 0, 0, P, 0] - kirsbp6d * ((xIrsGrb2 - xIrsGrb2b)/xIrsGrb2) * IRS[R, 0, P, 0, 0, P, P]$
$r618 = kirsbp6 * IRS[R, 0, P, 0, P, 0, 0] - kirsbp6d * ((xIrsGrb2 - xIrsGrb2b)/xIrsGrb2) * IRS[R, 0, P, 0, P, 0, P]$
$r619 = kirsbp6 * IRS[R, 0, P, 0, P, P, 0] - kirsbp6d * ((xIrsGrb2 - xIrsGrb2b)/xIrsGrb2) * IRS[R, 0, P, 0, P, P, P]$
$r620 = kirsbp6 * IRS[R, 0, P, P, 0, 0, 0] - kirsbp6d * ((xIrsGrb2 - xIrsGrb2b)/xIrsGrb2) * IRS[R, 0, P, P, 0, 0, P]$
$r621 = kirsbp6 * IRS[R, 0, P, P, 0, P, 0] - kirsbp6d * ((xIrsGrb2 - xIrsGrb2b)/xIrsGrb2) * IRS[R, 0, P, P, 0, P, P]$
$r622 = kirsbp6 * IRS[R, 0, P, P, P, 0, 0] - kirsbp6d * ((xIrsGrb2 - xIrsGrb2b)/xIrsGrb2) * IRS[R, 0, P, P, P, 0, P]$
$r623 = kirsbp6 * IRS[R, 0, P, P, P, P, 0] - kirsbp6d * ((xIrsGrb2 - xIrsGrb2b)/xIrsGrb2) * IRS[R, 0, P, P, P, P, P]$
$r624 = kirsbp6 * IRS[R, P, 0, 0, 0, 0, 0] - kirsbp6d * ((xIrsGrb2 - xIrsGrb2b)/xIrsGrb2) * IRS[R, P, 0, 0, 0, 0, P]$
$r625 = kirsbp6 * IRS[R, P, 0, 0, 0, P, 0] - kirsbp6d * ((xIrsGrb2 - xIrsGrb2b)/xIrsGrb2) * IRS[R, P, 0, 0, 0, P, P]$
$r626 = kirsbp6 * IRS[R, P, 0, 0, P, 0, 0] - kirsbp6d * ((xIrsGrb2 - xIrsGrb2b)/xIrsGrb2) * IRS[R, P, 0, 0, P, 0, P]$
$r627 = kirsbp6 * IRS[R, P, 0, 0, P, P, 0] - kirsbp6d * ((xIrsGrb2 - xIrsGrb2b)/xIrsGrb2) * IRS[R, P, 0, 0, P, P, P]$
$r628 = kirsbp6 * IRS[R, P, 0, P, 0, 0, 0] - kirsbp6d * ((xIrsGrb2 - xIrsGrb2b)/xIrsGrb2) * IRS[R, P, 0, P, 0, 0, P]$
$r629 = kirsbp6 * IRS[R, P, 0, P, 0, P, 0] - kirsbp6d * ((xIrsGrb2 - xIrsGrb2b)/xIrsGrb2) * IRS[R, P, 0, P, 0, P, P]$

$$r630 = kirsbp6 * IRS[R, P, 0, P, P, 0, 0] - kirsbp6d * ((xIrsGrb2 - xIrsGrb2b)/xIrsGrb2) * IRS[R, P, 0, P, P, 0, P]$$
$$r631 = kirsbp6 * IRS[R, P, 0, P, P, P, 0] - kirsbp6d * ((xIrsGrb2 - xIrsGrb2b)/xIrsGrb2) * IRS[R, P, 0, P, P, P, P]$$
$$r632 = kirsbp6 * IRS[R, P, P, 0, 0, 0, 0] - kirsbp6d * ((xIrsGrb2 - xIrsGrb2b)/xIrsGrb2) * IRS[R, P, P, 0, 0, 0, P]$$
$$r633 = kirsbp6 * IRS[R, P, P, 0, 0, P, 0] - kirsbp6d * ((xIrsGrb2 - xIrsGrb2b)/xIrsGrb2) * IRS[R, P, P, 0, 0, P, P]$$
$$r634 = kirsbp6 * IRS[R, P, P, 0, P, 0, 0] - kirsbp6d * ((xIrsGrb2 - xIrsGrb2b)/xIrsGrb2) * IRS[R, P, P, 0, P, 0, P]$$
$$r635 = kirsbp6 * IRS[R, P, P, 0, P, P, 0] - kirsbp6d * ((xIrsGrb2 - xIrsGrb2b)/xIrsGrb2) * IRS[R, P, P, 0, P, P, P]$$
$$r636 = kirsbp6 * IRS[R, P, P, P, 0, 0, 0] - kirsbp6d * ((xIrsGrb2 - xIrsGrb2b)/xIrsGrb2) * IRS[R, P, P, P, 0, 0, P]$$
$$r637 = kirsbp6 * IRS[R, P, P, P, 0, P, 0] - kirsbp6d * ((xIrsGrb2 - xIrsGrb2b)/xIrsGrb2) * IRS[R, P, P, P, 0, P, P]$$
$$r638 = kirsbp6 * IRS[R, P, P, P, P, 0, 0] - kirsbp6d * ((xIrsGrb2 - xIrsGrb2b)/xIrsGrb2) * IRS[R, P, P, P, P, 0, P]$$
$$r639 = kirsbp6 * IRS[R, P, P, P, P, P, 0] - kirsbp6d * ((xIrsGrb2 - xIrsGrb2b)/xIrsGrb2) * IRS[R, P, P, P, P, P, P]$$

e) construct ODEs

$$\frac{d}{dt}IRS[0,0,0,0,0,0,0] = -r192 - r256 - r288 - r320 - r352 - r384 - r416$$
$$\frac{d}{dt}IRS[0,0,0,0,0,0,P] = -r193 - r257 - r289 - r321 - r353 - r385 + r416$$
$$\frac{d}{dt}IRS[0,0,0,0,0,P,0] = -r194 - r258 - r290 - r322 - r354 + r384 - r417$$
$$\frac{d}{dt}IRS[0,0,0,0,0,P,P] = -r195 - r259 - r291 - r323 - r355 + r385 + r417$$
$$\frac{d}{dt}IRS[0,0,0,0,P,0,0] = -r196 - r260 - r292 - r324 + r352 - r386 - r418$$
$$\frac{d}{dt}IRS[0,0,0,0,P,0,P] = -r197 - r261 - r293 - r325 + r353 - r387 + r418$$
$$\frac{d}{dt}IRS[0,0,0,0,P,P,0] = -r198 - r262 - r294 - r326 + r354 + r386 - r419$$
$$\frac{d}{dt}IRS[0,0,0,0,P,P,P] = -r199 - r263 - r295 - r327 + r355 + r387 + r419$$
$$\frac{d}{dt}IRS[0,0,0,P,0,0,0] = -r200 - r264 - r296 + r320 - r356 - r388 - r420$$
$$\frac{d}{dt}IRS[0,0,0,P,0,0,P] = -r201 - r265 - r297 + r321 - r357 - r389 + r420$$
$$\frac{d}{dt}IRS[0,0,0,P,0,P,0] = -r202 - r266 - r298 + r322 - r358 + r388 - r421$$
$$\frac{d}{dt}IRS[0,0,0,P,0,P,P] = -r203 - r267 - r299 + r323 - r359 + r389 + r421$$
$$\frac{d}{dt}IRS[0,0,0,P,P,0,0] = -r204 - r268 - r300 + r324 + r356 - r390 - r422$$
$$\frac{d}{dt}IRS[0,0,0,P,P,0,P] = -r205 - r269 - r301 + r325 + r357 - r391 + r422$$
$$\frac{d}{dt}IRS[0,0,0,P,P,P,0] = -r206 - r270 - r302 + r326 + r358 + r390 - r423$$
$$\frac{d}{dt}IRS[0,0,0,P,P,P,P] = -r207 - r271 - r303 + r327 + r359 + r391 + r423$$
$$\frac{d}{dt}IRS[0,0,P,0,0,0,0] = -r208 - r272 + r288 - r328 - r360 - r392 - r424$$
$$\frac{d}{dt}IRS[0,0,P,0,0,0,P] = -r209 - r273 + r289 - r329 - r361 - r393 + r424$$
$$\frac{d}{dt}IRS[0,0,P,0,0,P,0] = -r210 - r274 + r290 - r330 - r362 + r392 - r425$$
$$\frac{d}{dt}IRS[0,0,P,0,0,P,P] = -r211 - r275 + r291 - r331 - r363 + r393 + r425$$
$$\frac{d}{dt}IRS[0,0,P,0,P,0,0] = -r212 - r276 + r292 - r332 + r360 - r394 - r426$$
$$\frac{d}{dt}IRS[0,0,P,0,P,0,P] = -r213 - r277 + r293 - r333 + r361 - r395 + r426$$
$$\frac{d}{dt}IRS[0,0,P,0,P,P,0] = -r214 - r278 + r294 - r334 + r362 + r394 - r427$$
$$\frac{d}{dt}IRS[0,0,P,0,P,P,P] = -r215 - r279 + r295 - r335 + r363 + r395 + r427$$
$$\frac{d}{dt}IRS[0,0,P,P,0,0,0] = -r216 - r280 + r296 + r328 - r364 - r396 - r428$$
$$\frac{d}{dt}IRS[0,0,P,P,0,0,P] = -r217 - r281 + r297 + r329 - r365 - r397 + r428$$

$$\frac{d}{dt}IRS[0,0,P,P,0,P,0] = -r218 - r282 + r298 + r330 - r366 + r396 - r429$$
$$\frac{d}{dt}IRS[0,0,P,P,0,P,P] = -r219 - r283 + r299 + r331 - r367 + r397 + r429$$
$$\frac{d}{dt}IRS[0,0,P,P,P,0,0] = -r220 - r284 + r300 + r332 + r364 - r398 - r430$$
$$\frac{d}{dt}IRS[0,0,P,P,P,0,P] = -r221 - r285 + r301 + r333 + r365 - r399 + r430$$
$$\frac{d}{dt}IRS[0,0,P,P,P,P,0] = -r222 - r286 + r302 + r334 + r366 + r398 - r431$$
$$\frac{d}{dt}IRS[0,0,P,P,P,P,P] = -r223 - r287 + r303 + r335 + r367 + r399 + r431$$
$$\frac{d}{dt}IRS[0,P,0,0,0,0,0] = -r224 + r256 - r304 - r336 - r368 - r400 - r432$$
$$\frac{d}{dt}IRS[0,P,0,0,0,0,P] = -r225 + r257 - r305 - r337 - r369 - r401 + r432$$
$$\frac{d}{dt}IRS[0,P,0,0,0,P,0] = -r226 + r258 - r306 - r338 - r370 + r400 - r433$$
$$\frac{d}{dt}IRS[0,P,0,0,0,P,P] = -r227 + r259 - r307 - r339 - r371 + r401 + r433$$
$$\frac{d}{dt}IRS[0,P,0,0,P,0,0] = -r228 + r260 - r308 - r340 + r368 - r402 - r434$$
$$\frac{d}{dt}IRS[0,P,0,0,P,0,P] = -r229 + r261 - r309 - r341 + r369 - r403 + r434$$
$$\frac{d}{dt}IRS[0,P,0,0,P,P,0] = -r230 + r262 - r310 - r342 + r370 + r402 - r435$$
$$\frac{d}{dt}IRS[0,P,0,0,P,P,P] = -r231 + r263 - r311 - r343 + r371 + r403 + r435$$
$$\frac{d}{dt}IRS[0,P,0,P,0,0,0] = -r232 + r264 - r312 + r336 - r372 - r404 - r436$$
$$\frac{d}{dt}IRS[0,P,0,P,0,0,P] = -r233 + r265 - r313 + r337 - r373 - r405 + r436$$
$$\frac{d}{dt}IRS[0,P,0,P,0,P,0] = -r234 + r266 - r314 + r338 - r374 + r404 - r437$$
$$\frac{d}{dt}IRS[0,P,0,P,0,P,P] = -r235 + r267 - r315 + r339 - r375 + r405 + r437$$
$$\frac{d}{dt}IRS[0,P,0,P,P,0,0] = -r236 + r268 - r316 + r340 + r372 - r406 - r438$$
$$\frac{d}{dt}IRS[0,P,0,P,P,0,P] = -r237 + r269 - r317 + r341 + r373 - r407 + r438$$
$$\frac{d}{dt}IRS[0,P,0,P,P,P,0] = -r238 + r270 - r318 + r342 + r374 + r406 - r439$$
$$\frac{d}{dt}IRS[0,P,0,P,P,P,P] = -r239 + r271 - r319 + r343 + r375 + r407 + r439$$
$$\frac{d}{dt}IRS[0,P,P,0,0,0,0] = -r240 + r272 + r304 - r344 - r376 - r408 - r440$$
$$\frac{d}{dt}IRS[0,P,P,0,0,0,P] = -r241 + r273 + r305 - r345 - r377 - r409 + r440$$
$$\frac{d}{dt}IRS[0,P,P,0,0,P,0] = -r242 + r274 + r306 - r346 - r378 + r408 - r441$$
$$\frac{d}{dt}IRS[0,P,P,0,0,P,P] = -r243 + r275 + r307 - r347 - r379 + r409 + r441$$

$$\frac{d}{dt}IRS[0,P,P,0,P,0,0] = -r244 + r276 + r308 - r348 + r376 - r410 - r442$$
$$\frac{d}{dt}IRS[0,P,P,0,P,0,P] = -r245 + r277 + r309 - r349 + r377 - r411 + r442$$
$$\frac{d}{dt}IRS[0,P,P,0,P,P,0] = -r246 + r278 + r310 - r350 + r378 + r410 - r443$$
$$\frac{d}{dt}IRS[0,P,P,0,P,P,P] = -r247 + r279 + r311 - r351 + r379 + r411 + r443$$
$$\frac{d}{dt}IRS[0,P,P,P,0,0,0] = -r248 + r280 + r312 + r344 - r380 - r412 - r444$$
$$\frac{d}{dt}IRS[0,P,P,P,0,0,P] = -r249 + r281 + r313 + r345 - r381 - r413 + r444$$
$$\frac{d}{dt}IRS[0,P,P,P,0,P,0] = -r250 + r282 + r314 + r346 - r382 + r412 - r445$$
$$\frac{d}{dt}IRS[0,P,P,P,0,P,P] = -r251 + r283 + r315 + r347 - r383 + r413 + r445$$
$$\frac{d}{dt}IRS[0,P,P,P,P,0,0] = -r252 + r284 + r316 + r348 + r380 - r414 - r446$$
$$\frac{d}{dt}IRS[0,P,P,P,P,0,P] = -r253 + r285 + r317 + r349 + r381 - r415 + r446$$
$$\frac{d}{dt}IRS[0,P,P,P,P,P,0] = -r254 + r286 + r318 + r350 + r382 + r414 - r447$$
$$\frac{d}{dt}IRS[0,P,P,P,P,P,P] = -r255 + r287 + r319 + r351 + r383 + r415 + r447$$
$$\frac{d}{dt}IRS[R,0,0,0,0,0,0] = r192 - r448 - r480 - r512 - r544 - r576 - r608$$
$$\frac{d}{dt}IRS[R,0,0,0,0,0,P] = r193 - r449 - r481 - r513 - r545 - r577 + r608$$
$$\frac{d}{dt}IRS[R,0,0,0,0,P,0] = r194 - r450 - r482 - r514 - r546 + r576 - r609$$
$$\frac{d}{dt}IRS[R,0,0,0,0,P,P] = r195 - r451 - r483 - r515 - r547 + r577 + r609$$
$$\frac{d}{dt}IRS[R,0,0,0,P,0,0] = r196 - r452 - r484 - r516 + r544 - r578 - r610$$
$$\frac{d}{dt}IRS[R,0,0,0,P,0,P] = r197 - r453 - r485 - r517 + r545 - r579 + r610$$
$$\frac{d}{dt}IRS[R,0,0,0,P,P,0] = r198 - r454 - r486 - r518 + r546 + r578 - r611$$
$$\frac{d}{dt}IRS[R,0,0,0,P,P,P] = r199 - r455 - r487 - r519 + r547 + r579 + r611$$
$$\frac{d}{dt}IRS[R,0,0,P,0,0,0] = r200 - r456 - r488 + r512 - r548 - r580 - r612$$
$$\frac{d}{dt}IRS[R,0,0,P,0,0,P] = r201 - r457 - r489 + r513 - r549 - r581 + r612$$
$$\frac{d}{dt}IRS[R,0,0,P,0,P,0] = r202 - r458 - r490 + r514 - r550 + r580 - r613$$
$$\frac{d}{dt}IRS[R,0,0,P,0,P,P] = r203 - r459 - r491 + r515 - r551 + r581 + r613$$
$$\frac{d}{dt}IRS[R,0,0,P,P,0,0] = r204 - r460 - r492 + r516 + r548 - r582 - r614$$
$$\frac{d}{dt}IRS[R,0,0,P,P,0,P] = r205 - r461 - r493 + r517 + r549 - r583 + r614$$

$$\frac{d}{dt}IRS[R,0,0,P,P,P,0] = r206 - r462 - r494 + r518 + r550 + r582 - r615$$
$$\frac{d}{dt}IRS[R,0,0,P,P,P,P] = r207 - r463 - r495 + r519 + r551 + r583 + r615$$
$$\frac{d}{dt}IRS[R,0,P,0,0,0,0] = r208 - r464 + r480 - r520 - r552 - r584 - r616$$
$$\frac{d}{dt}IRS[R,0,P,0,0,0,P] = r209 - r465 + r481 - r521 - r553 - r585 + r616$$
$$\frac{d}{dt}IRS[R,0,P,0,0,P,0] = r210 - r466 + r482 - r522 - r554 + r584 - r617$$
$$\frac{d}{dt}IRS[R,0,P,0,0,P,P] = r211 - r467 + r483 - r523 - r555 + r585 + r617$$
$$\frac{d}{dt}IRS[R,0,P,0,P,0,0] = r212 - r468 + r484 - r524 + r552 - r586 - r618$$
$$\frac{d}{dt}IRS[R,0,P,0,P,0,P] = r213 - r469 + r485 - r525 + r553 - r587 + r618$$
$$\frac{d}{dt}IRS[R,0,P,0,P,P,0] = r214 - r470 + r486 - r526 + r554 + r586 - r619$$
$$\frac{d}{dt}IRS[R,0,P,0,P,P,P] = r215 - r471 + r487 - r527 + r555 + r587 + r619$$
$$\frac{d}{dt}IRS[R,0,P,P,0,0,0] = r216 - r472 + r488 + r520 - r556 - r588 - r620$$
$$\frac{d}{dt}IRS[R,0,P,P,0,0,P] = r217 - r473 + r489 + r521 - r557 - r589 + r620$$
$$\frac{d}{dt}IRS[R,0,P,P,0,P,0] = r218 - r474 + r490 + r522 - r558 + r588 - r621$$
$$\frac{d}{dt}IRS[R,0,P,P,0,P,P] = r219 - r475 + r491 + r523 - r559 + r589 + r621$$
$$\frac{d}{dt}IRS[R,0,P,P,P,0,0] = r220 - r476 + r492 + r524 + r556 - r590 - r622$$
$$\frac{d}{dt}IRS[R,0,P,P,P,0,P] = r221 - r477 + r493 + r525 + r557 - r591 + r622$$
$$\frac{d}{dt}IRS[R,0,P,P,P,P,0] = r222 - r478 + r494 + r526 + r558 + r590 - r623$$
$$\frac{d}{dt}IRS[R,0,P,P,P,P,P] = r223 - r479 + r495 + r527 + r559 + r591 + r623$$
$$\frac{d}{dt}IRS[R,P,0,0,0,0,0] = r224 + r448 - r496 - r528 - r560 - r592 - r624$$
$$\frac{d}{dt}IRS[R,P,0,0,0,0,P] = r225 + r449 - r497 - r529 - r561 - r593 + r624$$
$$\frac{d}{dt}IRS[R,P,0,0,0,P,0] = r226 + r450 - r498 - r530 - r562 + r592 - r625$$
$$\frac{d}{dt}IRS[R,P,0,0,0,P,P] = r227 + r451 - r499 - r531 - r563 + r593 + r625$$
$$\frac{d}{dt}IRS[R,P,0,0,P,0,0] = r228 + r452 - r500 - r532 + r560 - r594 - r626$$
$$\frac{d}{dt}IRS[R,P,0,0,P,0,P] = r229 + r453 - r501 - r533 + r561 - r595 + r626$$
$$\frac{d}{dt}IRS[R,P,0,0,P,P,0] = r230 + r454 - r502 - r534 + r562 + r594 - r627$$
$$\frac{d}{dt}IRS[R,P,0,0,P,P,P] = r231 + r455 - r503 - r535 + r563 + r595 + r627$$

$$\frac{d}{dt}IRS[R,P,0,P,0,0,0] = r232 + r456 - r504 + r528 - r564 - r596 - r628$$
$$\frac{d}{dt}IRS[R,P,0,P,0,0,P] = r233 + r457 - r505 + r529 - r565 - r597 + r628$$
$$\frac{d}{dt}IRS[R,P,0,P,0,P,0] = r234 + r458 - r506 + r530 - r566 + r596 - r629$$
$$\frac{d}{dt}IRS[R,P,0,P,0,P,P] = r235 + r459 - r507 + r531 - r567 + r597 + r629$$
$$\frac{d}{dt}IRS[R,P,0,P,P,0,0] = r236 + r460 - r508 + r532 + r564 - r598 - r630$$
$$\frac{d}{dt}IRS[R,P,0,P,P,0,P] = r237 + r461 - r509 + r533 + r565 - r599 + r630$$
$$\frac{d}{dt}IRS[R,P,0,P,P,P,0] = r238 + r462 - r510 + r534 + r566 + r598 - r631$$
$$\frac{d}{dt}IRS[R,P,0,P,P,P,P] = r239 + r463 - r511 + r535 + r567 + r599 + r631$$
$$\frac{d}{dt}IRS[R,P,P,0,0,0,0] = r240 + r464 + r496 - r536 - r568 - r600 - r632$$
$$\frac{d}{dt}IRS[R,P,P,0,0,0,P] = r241 + r465 + r497 - r537 - r569 - r601 + r632$$
$$\frac{d}{dt}IRS[R,P,P,0,0,P,0] = r242 + r466 + r498 - r538 - r570 + r600 - r633$$
$$\frac{d}{dt}IRS[R,P,P,0,0,P,P] = r243 + r467 + r499 - r539 - r571 + r601 + r633$$
$$\frac{d}{dt}IRS[R,P,P,0,P,0,0] = r244 + r468 + r500 - r540 + r568 - r602 - r634$$
$$\frac{d}{dt}IRS[R,P,P,0,P,0,P] = r245 + r469 + r501 - r541 + r569 - r603 + r634$$
$$\frac{d}{dt}IRS[R,P,P,0,P,P,0] = r246 + r470 + r502 - r542 + r570 + r602 - r635$$
$$\frac{d}{dt}IRS[R,P,P,0,P,P,P] = r247 + r471 + r503 - r543 + r571 + r603 + r635$$
$$\frac{d}{dt}IRS[R,P,P,P,0,0,0] = r248 + r472 + r504 + r536 - r572 - r604 - r636$$
$$\frac{d}{dt}IRS[R,P,P,P,0,0,P] = r249 + r473 + r505 + r537 - r573 - r605 + r636$$
$$\frac{d}{dt}IRS[R,P,P,P,0,P,0] = r250 + r474 + r506 + r538 - r574 + r604 - r637$$
$$\frac{d}{dt}IRS[R,P,P,P,0,P,P] = r251 + r475 + r507 + r539 - r575 + r605 + r637$$
$$\frac{d}{dt}IRS[R,P,P,P,P,0,0] = r252 + r476 + r508 + r540 + r572 - r606 - r638$$
$$\frac{d}{dt}IRS[R,P,P,P,P,0,P] = r253 + r477 + r509 + r541 + r573 - r607 + r638$$
$$\frac{d}{dt}IRS[R,P,P,P,P,P,0] = r254 + r478 + r510 + r542 + r574 + r606 - r639$$
$$\frac{d}{dt}IRS[R,P,P,P,P,P,P] = r255 + r479 + r511 + r543 + r575 + r607 + r639$$
