# Supplementary material for: Reduced modeling of signal transduction – a modular approach
Source: BMC Bioinformatics. 2007 Sep 13;8:336. doi: 10.1186/1471-2105-8-336 (PMC2216040; doi:10.1186/1471-2105-8-336)
Supplement: Additional file 4 — Documentation and analysis of the larger example system [file 1471-2105-8-336-S4.pdf]

Detailed description of a larger example system

Detailed model

Nomenclature of state variables is analogous to the small example system and specified as follows: The unmodified receptor is denoted as $DOOO$. The first O specifies the unoccupied ligand binding site, the second one the unphosphorylated regulatory phosphorylation site and the third one the unphosphorylated effector binding site. Phosphorylation of a phosphorylation site leads to P on the corresponding position, ligand binding leads to L on the corresponding position. E, Ep, F on the last position specifies a phosphorylated binding site with bound E, Ep, EF, respectively. Free species are referred to as E, Ep, EF and F. Note that in contrast to the reduced model there is no qualitative difference between p and P. Both represent unoccupied phosphorylation sites. Phosphorylation on receptor sites is denoted by P, while phosphorylation of E is denoted by p. The sum of all catalytically active receptors is denoted as D_{activ}. All receptors with regulatory phosphorylation are assumed to be catalytically active and phosphorylate receptor bound E. Only receptor bound E is phosphorylated. Dephosphorylation of E is independent of E- binding to the receptor.
Rates d_1-d_{10} describe ligand binding, d_{11}-d_{20} regulatory phosphorylation and d_{21}-d_{24} binding site phosphorylation on the receptor. Binding of E, phosphorylation of receptor bound E and binding of F to receptor bound Ep is described by rates d_{25}-d_{28}, d_{29}-d_{32} and d_{33}-d_{36}, respectively. Binding of Ep and EF to the phosphorylated binding site of the receptor is described by rates d_{37}-d_{40} and d_{41}-d_{44}, respectively. Dephosphorylation of free Ep and binding of F to free Ep (yielding EF) is described by rates d_{45} and d_{46}. All reactions are visualized in Figure 1.

$$\begin{aligned} D_{activ} = {} & DOPO + DOPP + DOPE + DOPEp \\ & + DOPF + DLPO + DLPP + DLPE \\ & + DLPEp + DLPF \end{aligned}$$

$$\begin{aligned}
d_1 &= k_1 \cdot L \cdot DOOO - k_{-1} \cdot DLOO \\
d_2 &= k_1 \cdot L \cdot DOOP - k_{-1} \cdot DLOP \\
d_3 &= k_1 \cdot L \cdot DOPO - k_{-1} \cdot DLPO \\
d_4 &= k_1 \cdot L \cdot DOPP - k_{-1} \cdot DLPP \\
d_5 &= k_1 \cdot L \cdot DOOE - k_{-1} \cdot DLOE \\
d_6 &= k_1 \cdot L \cdot DOOEp - k_{-1} \cdot DLOEp \\
d_7 &= k_1 \cdot L \cdot DOOF - k_{-1} \cdot DLOF \\
d_8 &= k_1 \cdot L \cdot DOPE - k_{-1} \cdot DLPE \\
d_9 &= k_1 \cdot L \cdot DOPEp - k_{-1} \cdot DLPEp \\
d_{10} &= k_1 \cdot L \cdot DOPF - k_{-1} \cdot DLPF
\end{aligned}$$

$$\begin{aligned}
d_{11} &= k_2 \cdot DOOO - k_{-2} \cdot DOPO \\
d_{12} &= k_2 \cdot DOOP - k_{-2} \cdot DOPP \\
d_{13} &= k_2 \cdot DOOE - k_{-2} \cdot DOPE \\
d_{14} &= k_2 \cdot DOOEp - k_{-2} \cdot DOPEp \\
d_{15} &= k_2 \cdot DOOF - k_{-2} \cdot DOPF \\
d_{16} &= k_3 \cdot DLOO - k_{-3} \cdot DLPO \\
d_{17} &= k_3 \cdot DLOP - k_{-3} \cdot DLPP \\
d_{18} &= k_3 \cdot DLOE - k_{-3} \cdot DLPE \\
d_{19} &= k_3 \cdot DLOEp - k_{-3} \cdot DLPEp \\
d_{20} &= k_3 \cdot DLOF - k_{-3} \cdot DLPF \\
d_{21} &= k_2 \cdot DOOO - k_{-2} \cdot DOOP \\
d_{22} &= k_4 \cdot DOPO - k_{-4} \cdot DOPP \\
d_{23} &= k_3 \cdot DLOO - k_{-3} \cdot DLOP \\
d_{24} &= k_5 \cdot DLPO - k_{-5} \cdot DLPP \\
d_{25} &= k_6 \cdot E \cdot DOOP - k_{-6} \cdot DOOE \\
d_{26} &= k_6 \cdot E \cdot DOPP - k_{-6} \cdot DOPE \\
d_{27} &= k_6 \cdot E \cdot DLOP - k_{-6} \cdot DLOE \\
d_{28} &= k_6 \cdot E \cdot DLPP - k_{-6} \cdot DLPE \\
d_{29} &= k_7 \cdot D_{activ} \cdot DOOE - k_{-7} \cdot DOOEp \\
d_{30} &= k_7 \cdot D_{activ} \cdot DOPE - k_{-7} \cdot DOPEp \\
d_{31} &= k_7 \cdot D_{activ} \cdot DLOE - k_{-7} \cdot DLOEp \\
d_{32} &= k_7 \cdot D_{activ} \cdot DLPE - k_{-7} \cdot DLPEp \\
d_{33} &= k_8 \cdot F \cdot DOOEp - k_{-8} \cdot DOOF \\
d_{34} &= k_8 \cdot F \cdot DOPEp - k_{-8} \cdot DOPF \\
d_{35} &= k_8 \cdot F \cdot DLOEp - k_{-8} \cdot DLOF \\
d_{36} &= k_8 \cdot F \cdot DLPEp - k_{-8} \cdot DLPF \\
d_{37} &= k_6 \cdot Ep \cdot DOOP - k_{-6} \cdot DOOEp \\
d_{38} &= k_6 \cdot Ep \cdot DOPP - k_{-6} \cdot DOPEp \\
d_{39} &= k_6 \cdot Ep \cdot DLOP - k_{-6} \cdot DLOEp \\
d_{40} &= k_6 \cdot Ep \cdot DLPP - k_{-6} \cdot DLPEp \\
d_{41} &= k_6 \cdot EF \cdot DOOP - k_{-6} \cdot DOOF \\
d_{42} &= k_6 \cdot EF \cdot DOPP - k_{-6} \cdot DOPF \\
d_{43} &= k_6 \cdot EF \cdot DLOP - k_{-6} \cdot DLOF \\
d_{44} &= k_6 \cdot EF \cdot DLPP - k_{-6} \cdot DLPF \\
d_{45} &= -k_{-7} \cdot Ep \\
d_{46} &= k_8 \cdot Ep \cdot F - k_{-8} \cdot EF
\end{aligned}$$

$$\begin{aligned}
D\dot{O}OO &= -d_1 - d_{11} - d_{21} \\
D\dot{O}OP &= -d_2 - d_{12} + d_{21} - d_{25} - d_{37} - d_{41} \\
D\dot{O}PO &= -d_3 + d_{11} - d_{22} \\
D\dot{O}PP &= -d_4 + d_{12} + d_{22} - d_{26} - d_{38} - d_{42}
\end{aligned}$$

$$\dot{DOOE} = -d_5 - d_{13} + d_{25} - d_{29}$$
$$\dot{DOOEp} = -d_6 - d_{14} + d_{29} - d_{33} + d_{37}$$
$$\dot{DOOF} = -d_7 - d_{15} + d_{33} + d_{41}$$
$$\dot{DOPE} = -d_8 + d_{13} + d_{26} - d_{30}$$
$$\dot{DOPEp} = -d_9 + d_{14} + d_{30} - d_{34} + d_{38}$$
$$\dot{DOPF} = -d_{10} + d_{15} + d_{34} + d_{42}$$
$$\dot{DLOO} = d_1 - d_{16} - d_{23}$$
$$\dot{DLOP} = d_2 - d_{17} + d_{23} - d_{27} - d_{39} - d_{43}$$
$$\dot{DLPO} = d_3 + d_{16} - d_{24}$$
$$\dot{DLPP} = d_4 + d_{17} + d_{24} - d_{28} - d_{40} - d_{44}$$
$$\dot{DLOE} = d_5 - d_{18} + d_{27} - d_{31}$$
$$\dot{DLOEp} = d_6 - d_{19} + d_{31} - d_{35} + d_{39}$$
$$\dot{DLOF} = d_7 - d_{20} + d_{35} + d_{43}$$
$$\dot{DLPE} = d_8 + d_{18} + d_{28} - d_{32}$$
$$\dot{DLPEp} = d_9 + d_{19} + d_{32} - d_{36} + d_{40}$$
$$\dot{DLPF} = d_{10} + d_{20} + d_{36} + d_{44}$$
$$\dot{E} = -d_{25} - d_{26} - d_{27} - d_{28} - d_{45}$$
$$\dot{Ep} = -d_{37} - d_{38} - d_{39} - d_{40} + d_{45} - d_{46}$$
$$\dot{EF} = -d_{41} - d_{42} - d_{43} - d_{44} + d_{46}$$
$$\dot{F} = -d_{33} - d_{34} - d_{35} - d_{36} - d_{46}$$

As there is no synthesis or degradation of proteins there are three balance relations. Each of them could replace one differential equation.

$$\begin{aligned}
D_{total} = {} & DOOO + DOOP + DOPO + DOPP \\
& + DOOE + DOOEp + DOOF + DOPE \\
& + DOPEp + DOPF + DLOO + DLOP \\
& + DLPO + DLPP + DLOE + DLOEp \\
& + DLOF + DLPE + DLPEp + DLPF \\
E_{total} = {} & E + Ep + EF + DOOE + DOOEp \\
& + DOOF + DOPE + DOPEp + DOPF \\
& + DLOE + DLOEp + DLOF + DLPE \\
& + DLPEp + DLPF \\
F_{total} = {} & F + EF + DOOF + DOPF \\
& + DLOF + DLPF
\end{aligned}$$

Reduced model

Nomenclature of state variables is analogous to the small example system and specified as follows: The unmodified receptor is denoted as $ROOO$. The first O specifies the unoccupied ligand binding site, the second one the unphosphorylated regulatory phosphorylation site and the third one the unphosphorylated effector binding site. Phosphorylation of a phosphorylation site leads to P on the corresponding position, ligand binding leads to L on the corresponding position. Throughout the nomenclature of the reduced model P represents all phosphorylated sites, whereas p represents phosphorylated and unoccupied sites. $ROOP$, $ROPP$, $RLOP$ and $RLPP$ are the lumped states of the receptor layer. $ROPP$ for example specifies all receptor species without bound ligand, with phosphorylated regulatory site and phosphorylated binding site. $ROPP$ represents both species with and without occupied binding site. $ROOO$, $ROPO$, $RLOO$ and $RLPO$ are no lumped states, they have their direct equivalent in the detailed model. RXp, RXE and $RXEP$ are lumped receptor states of the E- layer. They specify all receptor species with phosphorylated binding site and no E, phosphorylated binding site and bound unphosphorylated E , and phosphorylated binding site and bound and phosphorylated E, respectively. Note that nomenclature is simplified. The one X in the names of the receptor species in this case represents two sites, namely the ligand binding site and the regulatory phosphorylation site. EP is also a lumped state of the E-layer. It specifies all E- species that are phosphorylated on the binding site for F and not bound to the receptor. XEp and XEF are the lumped states of the F- layer. They specify all phosphorylated (bound to the receptor and free) E- species without bound F and with bound F, respectively. x, xb, $x2$, $x2b$ are sums that are passed between the different layers. x is the sum of all receptor species with phosphorylated binding site for E, xb is the sum of all receptor species with phosphorylated binding site and bound E, $x2$ is the sum of all E- species with phosphorylated binding site for F, $x2b$ is the sum of all E- species with phosphorylated binding site and bound F (in this case only the specie XEF). Free species are referred to as E, EP and F. Note that EP is a lumped state as it represents free and phosphorylated E with bound and unbound F. The sum of all catalytically active receptors is denoted as R_{activ}. All receptors with regulatory phosphorylation are assumed to be catalytically active and phosphorylate receptor bound E. Only receptor bound E is phosphorylated. Rates r_1-r_4 describe ligand binding, r_5-r_8 regulatory phosphorylation and r_9-r_{12} binding site phosphorylation on the receptor. Binding of E, phosphorylation of receptor bound E and binding of EP is described by rates r_{13}, r_{14} and r_{15}, respectively. Dephosphorylation of free EP and binding of F to its phosphorylated binding site on E is described by rates r_{16} and r_{17}. As there is no synthesis or degradation of proteins there are three balance relations. They can replace one differential equation in each layer.

Receptor layer

$$r_1 = k_1 \cdot L \cdot ROOO - k_{-1} \cdot RLOO$$
$$r_2 = k_1 \cdot L \cdot ROOP - k_{-1} \cdot RLOP$$
$$r_3 = k_1 \cdot L \cdot ROPO - k_{-1} \cdot RLPO$$
$$r_4 = k_1 \cdot L \cdot ROPP - k_{-1} \cdot RLPP$$

$$
\begin{aligned}
r_5 &= k_2 \cdot ROOO - k_{-2} \cdot ROPO \\
r_6 &= k_2 \cdot ROOP - k_{-2} \cdot ROPP \\
r_7 &= k_3 \cdot RLOO - k_{-3} \cdot RLPO \\
r_8 &= k_3 \cdot RLOP - k_{-3} \cdot RLPP \\
r_9 &= k_2 \cdot ROOO - k_{-2} \cdot \frac{x - xb}{x} \cdot ROOP \\
r_{10} &= k_4 \cdot ROPO - k_{-4} \cdot \frac{x - xb}{x} \cdot ROPP \\
r_{11} &= k_3 \cdot RLOO - k_{-3} \cdot \frac{x - xb}{x} \cdot RLOP \\
r_{12} &= k_5 \cdot RLPO - k_{-5} \cdot \frac{x - xb}{x} \cdot RLPP
\end{aligned}
$$

$$
\begin{aligned}
RO\dot{O}O &= -r_1 - r_5 - r_9 \\
RO\dot{O}P &= -r_2 - r_6 + r_9 \\
RO\dot{P}O &= -r_3 + r_5 - r_{10} \\
RO\dot{P}P &= -r_4 + r_6 + r_{10} \\
RL\dot{O}O &= r_1 - r_7 - r_{11} \\
RL\dot{O}P &= r_2 - r_8 + r_{11} \\
RL\dot{P}O &= r_3 + r_7 - r_{12} \\
RL\dot{P}P &= r_4 + r_8 + r_{12}
\end{aligned}
$$

$$
\begin{aligned}
x &= ROOP + RLOP + ROPP + RLPP \\
R_{activ} &= ROPO + ROPP + RLPO + RLPP
\end{aligned}
$$

E- layer

$$
\begin{aligned}
r_{13} &= k_6 \cdot E \cdot RXp - k_{-6} \cdot RXE \\
r_{14} &= k_7 \cdot R_{activ} \cdot RXE - k_{-7} \cdot \frac{x2 - x2b}{x2} \cdot RXEP \\
r_{15} &= k_6 \cdot EP \cdot RXp - k_{-6} \cdot RXEP \\
r_{16} &= -k_{-7} \cdot \frac{x2 - x2b}{x2} \cdot EP
\end{aligned}
$$

$$
\begin{aligned}
R\dot{X}E &= r_{13} - r_{14} \\
RX\dot{E}P &= r_{14} + r_{15} \\
\dot{E} &= -r_{13} - r_{16} \\
\dot{EP} &= -r_{15} + r_{16} \\
xb &= RXE + RXEP \\
RXp &= x - xb \\
x2 &= RXEP + EP
\end{aligned}
$$

F- layer

$$
r_{17} = k_8 \cdot XEp \cdot F - k_{-8} \cdot XEF
$$

$$
\begin{aligned}
X\dot{E}F &= r_{17} \\
\dot{F} &= -r_{17} \\
XEp &= x2 - XEF \\
x2b &= XEF
\end{aligned}
$$

$$
\begin{aligned}
R_{total} &= ROOO + ROOP + ROPO + ROPP \\
&\quad + RLOO + RLOP + RLPO + RLPP \\
E_{total} &= E + EP + RXE + RXEP \\
F_{total} &= F + XEF
\end{aligned}
$$

Relations

The basic assumption of the layer based approach is the existence of relations between the species of the detailed model. These relations arise from independency assumptions between processes. For the larger example system there exist ten independent relations that explain the difference of ten between the detailed and reduced formalisms in the number of equations.

$$
\begin{aligned}
\frac{DOOP}{DOOE} &= \frac{DLOP}{DLOE} \\
\frac{DOOP}{DOOE} &= \frac{DOPP}{DOPE} \\
\frac{DOOP}{DOOE} &= \frac{DLPP}{DLPE} \\
\frac{DOOP}{DOOEp} &= \frac{DLOP}{DLOEp} \\
\frac{DOOP}{DOOEp} &= \frac{DOPP}{DOPEp} \\
\frac{DOOP}{DOOEp} &= \frac{DLPP}{DLPEp} \\
\frac{DOOP}{DOOF} &= \frac{DLOP}{DLOF} \\
\frac{DOOP}{DOOF} &= \frac{DOPP}{DOPF} \\
\frac{DOOP}{DOOF} &= \frac{DLPP}{DLPF} \\
\frac{DOOEp}{DOOF} &= \frac{Ep}{EF}
\end{aligned}
$$

One might wonder, why there are no equations like

$$
\frac{E}{Ep} = \frac{DOOE}{DOOEp} \quad \text{or} \quad \frac{DOOP}{DLOP} = \frac{DOOO}{DLOO}
$$

This equations would only hold if phosphorylation of E was independent of E-binding to the receptor or if autophosphorylation of the receptor was independent of ligand binding. Then additional reduction were possible.

Transformations

This chapter deals with conversion between states of detailed and reduced formalisms. The states E and F are equivalent in both formalisms. For clarity reasons the states Ep and EF of the detailed model are referred to as dEp and dEF. The state EP of the reduced model is referred to as rEP. With the relations above the transformation is invertible.

$$
\begin{aligned}
ROOO &= DOOO \\
ROPO &= DOPO \\
ROOP &= DOOP + DOOE + DOOEp + DOOF \\
ROPP &= DOPP + DOPE + DOPEp + DOPF \\
RLOO &= DLOO \\
RLPO &= DLPO \\
RLOP &= DLOP + DLOE + DLOEp + DLOF \\
RLPP &= DLPP + DLPE + DLPEp + DLPF \\
RXE &= DOOE + DOPE + DLOE + DLPE \\
RXEP &= DOOEp + DOPEp + DLOEp + DLPEp \\
&\quad + DOOF + DOPF + DLOF + DLPF \\
XEF &= DOOF + DOPF + DLOF + DLPF + dEF \\
rEP &= dEp + dEF
\end{aligned}
$$

$$
\begin{aligned}
DOOO &= ROOO \\
DOOP &= \frac{x - xb}{x} \cdot ROOP \\
DOPO &= ROPO \\
DOPP &= \frac{x - xb}{x} \cdot ROPP \\
DOOE &= \frac{ROOP}{x} \cdot RXE \\
DOOEp &= \frac{ROOP}{x} \cdot \frac{RXEP}{x2} \cdot XEp \\
DOOF &= \frac{ROOP}{x} \cdot \frac{RXEP}{x2} \cdot XEF \\
DOPE &= \frac{ROPP}{x} \cdot RXE \\
DOPEp &= \frac{ROPP}{x} \cdot \frac{x2 - x2b}{x2} \cdot RXEP \\
DOPF &= \frac{ROPP}{x} \cdot \frac{RXEP}{x2} \cdot XEF \\
DLOO &= RLOO \\
DLOP &= \frac{x - xb}{x} \cdot RLOP \\
DLPO &= RLPO \\
DLPP &= \frac{x - xb}{x} \cdot RLPP \\
DLOE &= \frac{RLOP}{x} \cdot RXE \\
DLOEp &= \frac{RLOP}{x} \cdot \frac{x2 - x2b}{x2} \cdot RXEP \\
DLOF &= \frac{RLOP}{x} \cdot \frac{RXEP}{x2} \cdot XEF \\
DLPE &= \frac{RLPP}{x} \cdot RXE \\
DLPEp &= \frac{RLPP}{x} \cdot \frac{x2 - x2b}{x2} \cdot RXEP \\
DLPF &= \frac{RLPP}{x} \cdot \frac{RXEP}{x2} \cdot XEF
\end{aligned}
$$

$$
\begin{aligned}
dEp &= \frac{rEP}{x2} \cdot XEp \\
dEF &= \frac{rEP}{x2} \cdot XEF
\end{aligned}
$$

Transformation of selected states

There are two general possibilities to approximate the states of the detailed model from the states of the reduced model. The first is to sum up the corresponding states of the detailed model for each lumped state. Micro-states have their direct counterpart in both formalisms, so there is a one-to-one relation for each micro-state. Then all relations between the species that arise from independency assumptions, as discussed in the manuscript, are included. The resulting system is analytically solved for the states of the detailed model. This is an brute force possibility, where all relations (about 145 millions for insulin signaling) have to be explicitly formulated. Additionally, automatic solving is critical for such highly nonlinear systems that can contain many equations. This method is demonstrated for the larger example system in the mathematical part of the additional material. The second possibility is a more intuitive, however systematic, one. It usually should be preferred, especially if only few states or pools of states of the detailed model are to be reconstructed. The starting point is an arbitrarily chosen lumped state that contains the state that shall be approximated. Then terms that specify characteristics of this state are multiplied with the chosen lumped state. The method is demonstrated on reconstruction of $DOOEp$ and $DXXF$. For $DOOEp$ reconstruction is performed starting from different lumped states.

$DOOEp$ is contained in $ROOP$, so we take $ROOP$ as a starting point. The fraction of $ROOP$ that has an occupied binding site is $\frac{xb}{x}$. The fraction of receptor bound E that is phosphorylated is $\frac{RXEP}{xb}$. The fraction of phosphorylated E that is not bound by F is $\frac{x2-x2b}{x2}$. Altogether :

$$
DOOEp = ROOP \cdot \frac{xb}{x} \cdot \frac{RXEP}{xb} \cdot \frac{x2 - x2b}{x2}
$$

$DOOEp$ is also contained in $RXEP$, so we take $RXEP$ as a starting point. The fraction of phosphorylated E that is not bound by F is $\frac{x2-x2b}{x2}$. The fraction of phosphorylated binding sites on the receptor with no regulatory phosphorylation or bound ligand is $\frac{ROOP}{x}$. The result is the same as above:

$$
DOOEp = RXEP \cdot \frac{x2 - x2b}{x2} \cdot \frac{ROOP}{x}
$$

A third possibility is shown in the transformation equations. All possibilities give identical results. This can be easily verified by inserting $x2 - x2b = XEp$.

$DXXF$ that is used in the optimization study represents all species with F bound to the receptor via E. $DXXF$ is contained in XEF. The fraction of phosphorylated E that is bound to the receptor is $\frac{RXEP}{x2}$.

$$DXXF = XEF \cdot \frac{RXEP}{x2}$$

Optimization studies

Optimizations studies for this system were curried out with the lsqnonlin routine from Matlab (The MathWorks, Inc.), version 7.2.0.294. This routine minimizes the sum of squares of the passed values. Optimization parameters were optimset('MaxIter', 100000, 'MaxFunEvals', 100000, 'TolFun', 1e-8, 'TolX', 1e-8), which is more stringent than the standard values. The choice of this criterion is a tradeoff between accuracy and time consumption. With the choice above, optimization took about ten CPU- days. Simulation time was 300 s. Ten data points output per simulation second were processed and passed to the minimization algorithm. The values passed to the lsqnonlin routine (out) were computed as

$$out = \frac{1}{|\delta| + \theta}\ ,\ \theta > 0$$

where δ is the difference between the value of the reduced model and the value computed from the states of the detailed model at the corresponding time. This form of scoring function was chosen as standard routines minimize sums of squares. However, in this special case maximization had to be performed. The positive constant θ had to be introduced to prevent division by zero if the values are identical. As a tradeoff between preventing too high values for small differences and a domination of the effect of θ in the minimization, θ was chosen to be 0.1 in the present study.
Start values for parameters and initial conditions were taken from Table 1. To vary inside physiologic ranges each parameter except the regulatory factors is allowed to be between hundred times the original value and a hundredth of this value. Therefore, variation within four degrees of magnitude around literature values was allowed. The regulatory factors describe the inhibition of autophosphorylation in the absence of bound insulin (f_{ins}) or in the presence of regulatory phosphorylation (f_{reg}). They are defined to be in the interval [0,1], variation was allowed in the interval [10^{-4},0.5]. It is assumed, that receptor dephosphorylation is not subject to any receptor modification: $k_{-2} = k_{-3} = k_{-4} = k_{-5}$ and that effectors E and F bind with the same kinetic constants to their phosphorylated binding sites: $k_6 = k_8$ and $k_{-6} = k_{-8}$. The resulting worst case parameters for both scenarios can be found in Table 1. Figures 2 and 3 show simulation results for both worst case scenarios. Reduction quality is still very high, the error is surely within the range of measurement errors.

References

[1] Wanant S, Quon M: **Insulin receptor binding kinetics: modeling and simulation studies.** *J Theor Biol* 2000, **205**(3):355–64.

[2] Faure R, Baquiran G, Bergeron J, Posner B: **The dephosphorylation of insulin and epidermal growth factor receptors. Role of endosome-associated phosphotyrosine phosphatase(s).** *J Biol Chem* 1992, **267**(16):11215–21.

[3] Drake P, Bevan A, Burgess J, Bergeron J, Posner B: **A role for tyrosine phosphorylation in both activation and inhibition of the insulin receptor tyrosine kinase in vivo.** *Endocrinology* 1996, **137**(11):4960–8.

[4] Felder S, Zhou M, Hu P, Urea J, Ullrich A, Chaudhuri M, White M, Shoelson S, Schlessinger J: **SH2 domains exhibit high-affinity binding to tyrosine-phosphorylated peptides yet also exhibit rapid dissociation and exchange.** *Mol Cell Biol* 1993, **13**(3):1449–55.

[5] Shoelson S, Chatterjee S, Chaudhuri M, White M: **YMXM motifs of IRS-1 define substrate specificity of the insulin receptor kinase.** *Proc Natl Acad Sci U S A* 1992, **89**(6):2027–31.

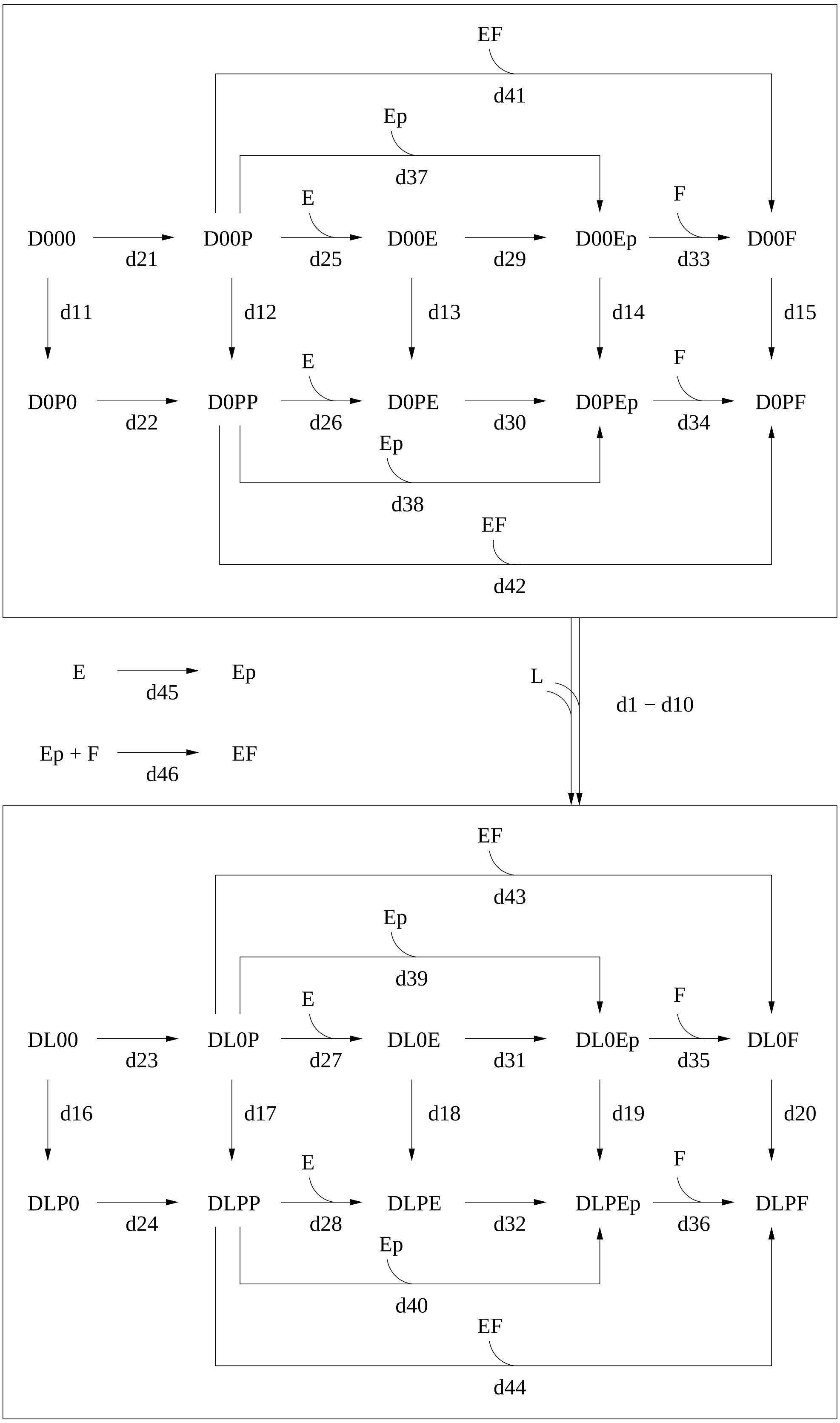

Figure 1: Reaction scheme of the detailed model

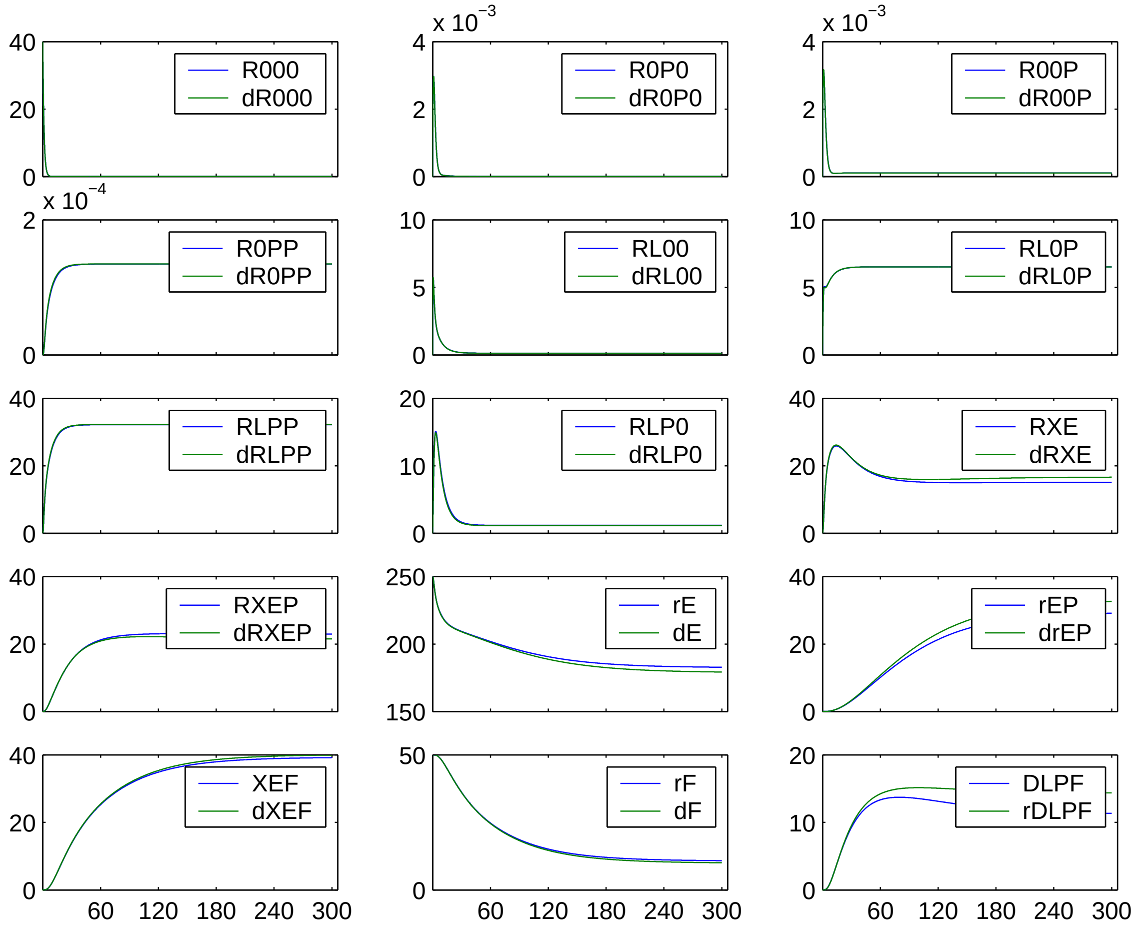

Figure 2: Worst case scenario for XEF

A simulation of the worst case scenario for XEF was performed. For Parameters and initial conditions see Table 1. The axis of abscissae is given in s, the axis of ordinates in nM.

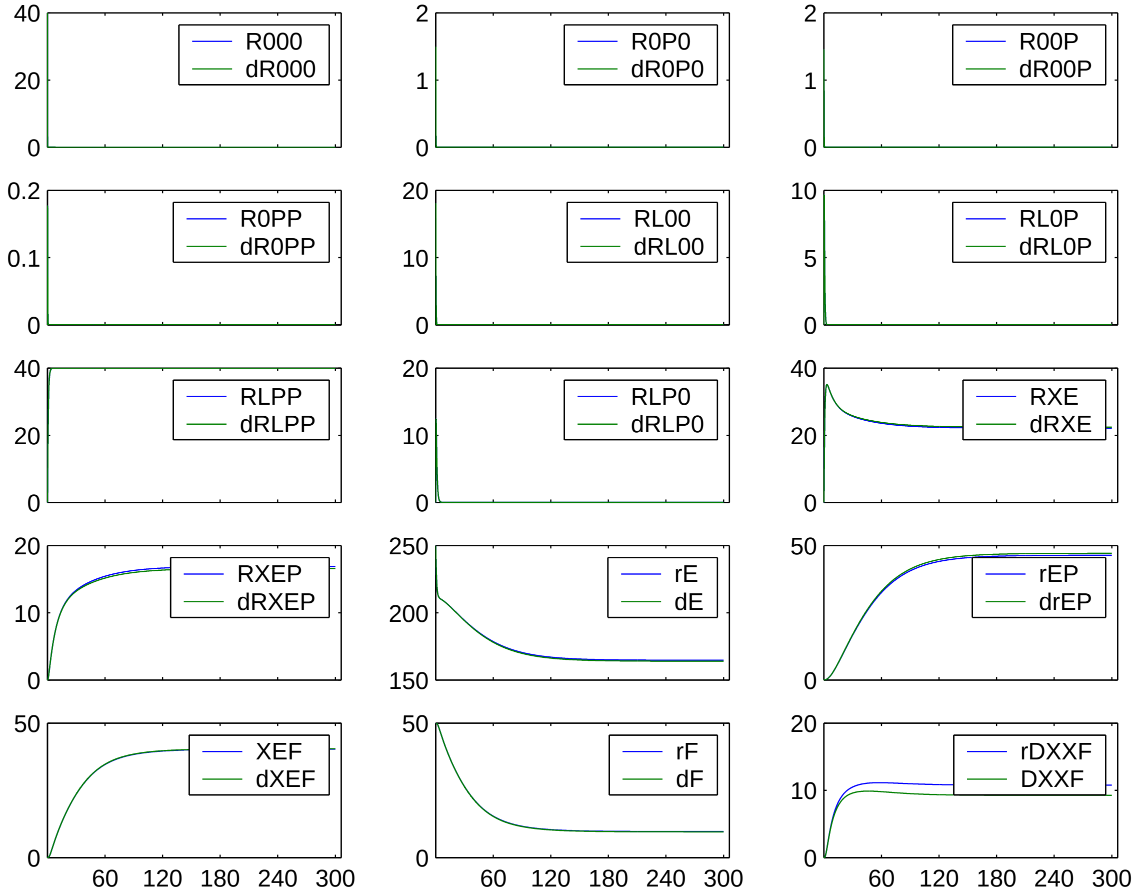

Figure 3: Worst case scenario for DXXF

A simulation of the worst case scenario for $DXXF$ was performed. For Parameters and initial conditions see Table 1. The axis of abscissae is given in s, the axis of ordinates in nM. State variables that are plotted in the upper rows show very fast dynamics. In grayscale printouts their curves may not be visible as they lie very close to coordinate axes.

Table 1: Parameters and initial conditions

Initial values were 40 nM for $DOOO$, 250 nM for E and 50 nM for F. All other initial values were 10^{-20} nM. Initial values for the reduced model were computed to be consistent. L is set to 100 nM, which is a typical insulin concentration for stimulation. k_3 defines autophosphorylation in the presence of ligand and the absence of regulatory phosphorylation. $k_2 = k_3 \cdot f_{ins}$ defines autophosphorylation in the absence of ligand and regulatory phosphorylation. $k_4 = k_3 \cdot f_{ins} \cdot f_{reg}$ defines autophosphorylation in the absence of ligand but with regulatory phosphorylation. $k_5 = k_3 \cdot f_{reg}$ defines autophosphorylation with ligand and regulatory phosphorylation. It is assumed that dephosphorylation is independent from other receptor modifications: $k_{-2} = k_{-3} = k_{-4} = k_{-5}$. It is further assumed that E and F bind with the same kinetic constants to their respective binding sites: $k_6 = k_8$ and $k_{-6} = k_{-8}$

k_i	original	source	XEF	DXXF	unit
k_1	0.001	[1]	$7.7549 \cdot 10^{-3}$	0.1	$nM^{-1}s^{-1}$
k_{-1}	$4 \cdot 10^{-4}$	[1]	$4.8735 \cdot 10^{-6}$	$4.0164 \cdot 10^{-6}$	s^{-1}
k_3	0.0231	[2]	1.9309	2.31	s^{-1}
k_{-3}	0.00385	[3]	0.38496	$3.8516 \cdot 10^{-5}$	s^{-1}
k_6	0.033	[4]	0.0044997	0.01413	$nM^{-1}s^{-1}$
k_{-6}	0.113	[4]	0.016023	0.077895	s^{-1}
k_7	10^{-5}	[5]	0.001	0.001	$nM^{-1}s^{-1}$
k_{-7}	0.000385	ass.	0.0385	0.0385	s^{-1}
f_{ins}	0.1	ass.	10^{-4}	0.5	-
f_{reg}	0.1	ass.	10^{-4}	0.5	-
